# Iron promoted end-on dinitrogen-bridging in heterobimetallic complexes of uranium and lanthanides†

**DOI:** 10.1039/d4sc01050g

**Published:** 2024-04-02

**Authors:** Nadir Jori, Juan J. Moreno, R. A. Keerthi Shivaraam, Thayalan Rajeshkumar, Rosario Scopelliti, Laurent Maron, Jesús Campos, Marinella Mazzanti

**Affiliations:** a Institut des Sciences et Ingénierie Chimiques, École Polytechnique Fédérale de Lausanne (EPFL) 1015 Lausanne Switzerland marinella.mazzanti@epfl.ch; b Laboratoire de Physique et Chimie des Nano-objets, Institut National des Sciences Appliquées 31077 Cedex 4 Toulouse France laurent.maron@irsamc.ups-tlse.fr; c Departamento de Química Inorgánica and Centro de Innovación en Química Avanzada (ORFEO-CINQA), Instituto de Investigaciones Químicas (IIQ), Consejo Superior de Investigaciones Científicas (CSIC) and Universidad de Sevilla 41092 Sevilla Spain

## Abstract

End-on binding of dinitrogen to low valent metal centres is common in transition metal chemistry but remains extremely rare in f-elements chemistry. In particular, heterobimetallic end-on N_2_ bridged complexes of lanthanides are unprecedented despite their potential relevance in catalytic reduction of dinitrogen. Here we report the synthesis and characterization of a series of N_2_ bridged heterobimetallic complexes of U(iii), Ln(iii) and Ln(ii) which were prepared by reacting the Fe dinitrogen complex [Fe(depe)_2_(N_2_)] (depe = 1,2-bis(diethylphosphino)-ethane), complex A with [M^III^{N(SiMe_3_)_2_}_3_] (M = U, Ce, Sm, Dy, Tm) and [Ln^II^{N(SiMe_3_)_2_}_2_], (Ln = Sm, Yb). Despite the lack of reactivity of the U(iii), Ln(iii) and Ln(ii) amide complexes with dinitrogen, the end-on dinitrogen bridged heterobimetallic complexes [{Fe(depe)_2_}(μ-η^1^:η^1^-N_2_)(M{N(SiMe_3_)_2_}_3_)], 1-M (M = U(iii), Ce(iii), Sm(iii), Dy(iii) and Tm(iii)), [{Fe(depe)_2_}(μ-η^1^:η^1^-N_2_)(Ln{N(SiMe_3_)_2_}_2_)], 1*-Ln (Ln = Sm(ii), Yb(ii)) and [{Fe(depe)_2_(μ-η^1^:η^1^-N_2_)}_2_{Sm^II^{N(SiMe_3_)_2_}_2_}], 3 could be prepared. The synthetic method used here allowed to isolate unprecedented end-on bridging N_2_ complexes of divalent lanthanides which provide relevant structural models for the species involved in the catalytic reduction of dinitrogen by Fe/Sm(ii) systems. Computational studies showed an essentially electrostatic interaction of the end-on bridging N_2_ with both Ln(iii) and Ln(ii) complexes with the degree of N_2_ activation correlating with their Lewis acidity. In contrast, a back-bonding covalent contribution to the U(iii)–N_2_Fe bond was identified by computational studies. Computational studies also suggest that end-on binding of N_2_ to U(iii) and Ln(ii) complexes is favoured for the iron-bound N_2_ compared to free N_2_ due to the higher N_2_ polarization.

## Abstract 2

End-on bridging dinitrogen binding to U(iii), Ln(iii) and Ln(ii) is favoured for the iron-bound N_2_ compared to free N_2_, resulting in increased N_2_ activation with increasing Ln Lewis acidity with a back-bonding contribution only found for U(iii).

## Graphical abstract

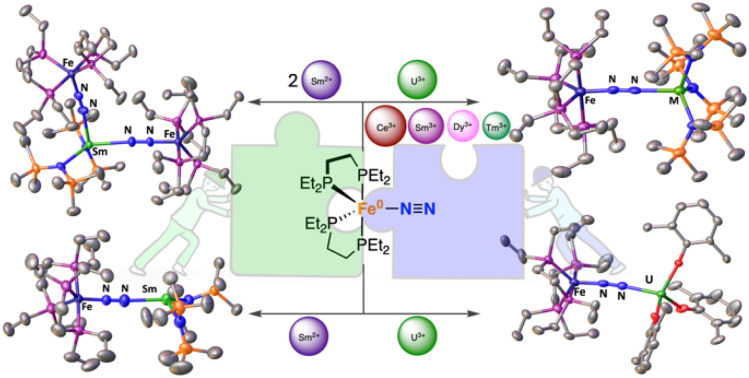

## Introduction

End-on binding of dinitrogen to low valent metal centres is common in transition metal chemistry.^1,2^ Most systems, including nitrogenase enzymes, capable of catalysing the conversion of dinitrogen to ammonia or other N-containing products, involve low valent metal intermediates binding dinitrogen in end-on fashion.^2,3^ A few homobimetallic complexes where the N_2_ molecule bridges two metals in a η^1^:η^1^ mode were also found to be active catalysts.^4,5^

N_2_ bridged heterometallic complexes were reported in seminal studies more than 50 years ago^6–15^ but the use of mid-to-late d-elements such as iron^16,17^ remains rare. Recently, N_2_ bridged heterobimetallic complexes^18–22^ have attracted increasing interest in transition metal chemistry for their potential to produce more polarized, and therefore more reactive, dinitrogen *via* a “push–pull” activation.^16,17,23–25^ However, Lewis acid–base interaction remains a difficult and underdeveloped route to the synthesis of heterobimetallic N_2_ complexes.

End-on binding of dinitrogen in f-elements is very rare compared to d-block transition metals and the seminal report of an heterobimetallic dinitrogen complex featuring N_2_ bridging uranium and molybdenum centres in a μ-η^1^:η^1^ coordination mode, [{([Ph]^*t*^BuN)_3_Mo}(μ-η^1^:η^1^-N_2_){U(N^*t*^Bu[3,5-C_6_H_3_Me_2_])_3_}], remains the only example of end-on bridging N_2_ heterobimetallic complex containing an f-element and a d block metal.^26^ The first example on an f-element capable of terminal end-on binding of dinitrogen was reported by Evans and co-workers in 2003 and led to the isolation under 80 psi N_2_ of the U(iii) complex [U^III^(C_5_Me_5_)_3_N_2_].^27^

Only very recently, the second example of a complex of uranium containing end-on bridging bound N_2_ was reported by Liddle and coworkers.^28^ Remarkably, despite the electron poor nature of the +V oxidation state of uranium, a U(v)–N_2_ backbonding interaction was demonstrated in the complex [U(BIPM^TMS^)(NAd)_2_(μ-η^1^:η^1^-N_2_){Li(2,2,2-cryptand)}] (BIPM = C(PPh_2_NSiMe_3_)_2_) that was probably enabled by cooperative binding of the Li^+^ cation to the end-on N_2_ and by the electron-rich nature of the ancillary ligands.^28^ However, most N_2_ complexes of the f-elements are dinuclear and contain side-on bridging N_2_ with different degrees of reduction.^2,29–46^

Only a few examples of homobimetallic rare earths complexes containing end-on bridging dinitrogen have been reported and they were until recently limited to bulky amide-ligated anions [{(N′′)_3_M}_2_(μ-η^1^:η^1^-N_2_)]^2−^ (N′′ = N(SiMe_3_)_2_, M = Sc^III^, Y^III^, Tb^III^).^47–49^ Amide supported μ-η^1^:η^1^-N_2_ complexes of late lanthanides were found to lose N_2_ above −35 °C while end-on bridging ligation could be observed below −90 °C for the larger Nd ion.^38^ More recently, thermally stable neutral [(Cp_2_^ttt^M)_2_(μ-η^1^:η^1^-N_2_)] (Cp^ttt^ = 1,2,4-^*t*^Bu_3_C_5_H_2_) complexes were isolated for Gd, Tb and Dy by Layfield and coworkers using the bulky Cp^ttt^ ligand.^49^

Here we explored the possibility of building N_2_ bridged heterobimetallic complexes of f-elements using the Fe dinitrogen complex [Fe(depe)_2_(N_2_)] (depe = 1,2-bis(diethylphosphino)-ethane), A,^50^ which was previously shown to act as a catalyst in the reduction of N_2_ to hydrazine.^51^ Complex A was selected because it provides a sterically accessible and nucleophilic N_2_ that was demonstrated in the seminal work of Szymczak and coworkers^16^ to form stable adducts with boranes and with a Fe(ii) complex. The binding of alkali metals was also demonstrated by spectroscopic studies, although these complexes were not crystallographically characterized. In contrast, the binding of f-elements, which can also act as strong Lewis acids, was not investigated. Besides the fundamental interest in dinitrogen reduction promoted by f-elements, which has resulted in the isolation of unusual N_2_^3−^ species^52–54^ and in rare examples of dinitrogen functionalization,^43^ there is currently high interest in probing the interaction of lanthanides with iron based dinitrogen complexes because of the high efficiency of Ln(ii) ions as electron sources in the catalytic conversion of N_2_ to ammonia or hydrazine.^55–58^ Here we isolated and characterized a range of rare end-on bridged dinitrogen complexes of uranium and lanthanides.

## Results and discussion

### Fe–U dinitrogen complexes

The U(iii) complexes [U^III^(Cp*)_3_] and [U^III^(Cp^tet^)_3_] (Cp* = C_5_Me_5_ and Cp^tet^ = C_5_Me_4_H) were reported to bind CO,^59^ but do not react with N_2_ at 1 atm and only [U^III^(Cp*)_3_] was found to bind reversibly N_2_ at 80 psi. Interested in understanding if N_2_ binding by [U^III^(Cp^tet^)_3_] could be promoted by using an iron dinitrogen complex we explored the reaction of [U^III^(Cp^tet^)_3_] with the dinitrogen complex [Fe(depe)_2_(N_2_)], A. However, no reaction occurs in toluene solution at −40 °C or 25 °C, as confirmed by ^1^H and ^31^P{^1^H} NMR spectroscopies (Fig. S1–S4†).

Since [U^III^{N(SiMe_3_)_2_}_3_] was also reported to promote CO coupling in arene non-polar solvents^60^ but did not show any reactivity with N_2_ we then investigated the possibility of promoting binding of dinitrogen to [U^III^{N(SiMe_3_)_2_}_3_] by reacting it with complex A.

**Scheme 1:**
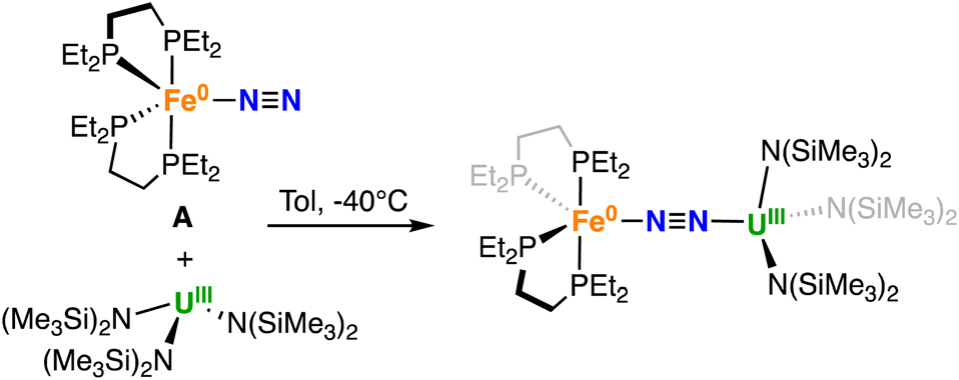
Synthesis of 1-U.

The dinitrogen complex [Fe(depe)_2_(N_2_)], A was reacted with [U^III^{N(SiMe_3_)_2_}_3_] in different solvents. The reaction of [Fe(depe)_2_(N_2_)], A^50^ with 1 equiv. of the U(iii) tris amido complex [U{N(SiMe_3_)_2_}_3_],^61,62^ at −40 °C in toluene led to the immediate full consumption of the reagents and to the formation of a new species as indicated by ^1^H and ^31^P {^1^H} NMR spectroscopy (Fig. S5 and S6†). Cooling down the concentrated reaction mixture afforded dark purple-brown crystals of the heteronuclear bridging dinitrogen complex [{Fe(depe)_2_}(μ-η^1^:η^1^-N_2_)(U{N(SiMe_3_)_2_}_3_)], 1-U in 68% yield (Scheme 1).

The ^31^P {^1^H} NMR spectrum of isolated 1-U measured at −40 °C shows a broad signal at *δ* = −25.7 ppm. Further broadening of the ^31^P{^1^H} NMR signal of 1-U was observed at higher temperatures suggesting that in toluene the Fe–U dinitrogen complex 1-U is in equilibrium with the precursors (Fig. S9†). Dissolution of crystals of 1-U in THF led to the immediate almost complete dissociation of the Fe–N_2_–U complex, with only traces (5%) of 1-U observed at −35 °C by ^31^P {^1^H} NMR spectroscopy (Fig. S7†).

**Fig. 1:**
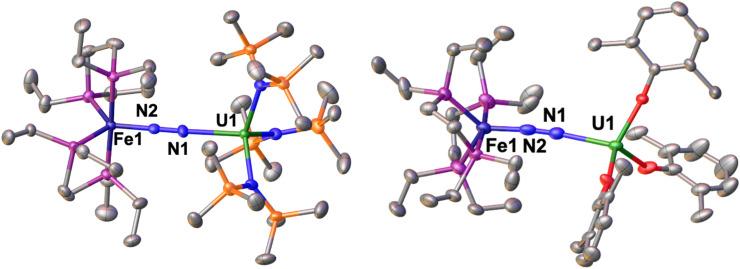
Solid-state molecular structure of 1-U (left) and 2 (right) with 50% probability ellipsoids. Colour code: uranium (green), phosphorus (purple), iron (midnight blue), oxygen (red), carbon (grey), silicon (orange). Hydrogen atoms and ^*t*^Bu groups were omitted for clarity.

The solid-state molecular structure of 1-U (Fig. 1, left) shows the presence of a heterobimetallic complex with dinitrogen bridging the uranium and iron centres in a μ-η^1^:η^1^ mode. The U centre is tetracoordinated by a N_2_ nitrogen and three amide nitrogen atoms in a pseudo tetrahedral geometry, featuring slightly longer U–N_amide_ distances (2.363(7) Å average) compared to those found in the precursor [U^III^{N(SiMe_3_)_2_}_3_] (2.320(4) Å, avg.).^62^ Furthermore, the U–N_2_ distance (2.498(4) Å) in 1-U compares well with the only terminal end-on N_2_ complex of uranium(iii), [U^III^(Cp*)_3_N_2_] (U–N distance: 2.485(9) Å).^27^

The Fe–N (1.739(4) Å) and Fe–P (2.223(3) Å) distances in 1-U are almost unaffected by the coordination to the uranium centre compared to the precursor A (Fe–N distance = 1.749(7) Å and Fe–P distance = 2.223(8) Å avg.),^50^ and the geometry of the iron centre remains trigonal bipyramidal. These geometric parameters are suggestive of Fe(0).^51^ The N–N distance in the bridging ligand (1.150(7) Å) is only slightly longer than the distance observed in complex A (1.139(2) Å) and [U^III^(Cp*)_3_N_2_] (1.120(14) Å), which were described as N^0^_2_ moieties. Overall, the structural parameters in 1-U support the formulation of the complex as a Fe(0)/U(iii)–N^0^_2_ species (Table 1).

**Table 1:** Mean values of selected bond lengths (Å) and angles (°) in the complexes 1-U, 2, and previously reported A^50^ and previously reported end-on bridged complexes [{([Ph]^*t*^BuN)_3_Mo}(μ-η^1^:η^1^-N_2_){U(N^*t*^Bu[3,5-C_6_H_3_Me_2_])_3_}],^26^ [Fe(depe)_2_(μ-η^1^:η^1^-N_2_)B(C_6_F_5_)_3_]^16^ and [Fe(depe)_2_(μ-η^1^:η^1^-N_2_)Fe(^i^Pr_2_Tp)][BArF_4_] (Tp = tris(pyrazolyl)borate)^16^

Complex	Fe–N	N–E (E = U, B, Fe)	N–N	N–N–E
1-U	1.739(4)	2.498(4)	1.150(7)	177.5(4)
2	1.752(5)	2.474(5)	1.169(7)	169.4(4)
A	1.749(7)	—	1.139(2)	—
[{([Ph]^*t*^BuN)_3_Mo}(μ-η^1^:η^1^-N_2_){U(N^*t*^Bu[3,5-C_6_H_3_Me_2_])_3_}]	—	2.220(9)	1.232(11)	173.9(8)
[U^III^(Cp*)_3_N_2_]	—	2.485(9)	1.120(14)	—
[Fe(depe)_2_(μ-η^1^:η^1^-N_2_)B(C_6_F_5_)_3_]	1.717(2)	—	1.186(3)	137.0(3)
[Fe(depe)_2_(μ-η^1^:η^1^-N_2_)Fe(^i^Pr_2_Tp)][BArF_4_]	1.744(avg.)	1.885(avg.)	1.179(avg.)	174.9(avg.)

The presence of U(iii) was corroborated by the X-band EPR spectrum of powdered 1-U at 6 K (Fig. S65†), that shows an EPR spectrum characteristic for a U(iii) centre with a pronounced narrow signal at low field around *g*_1_ = 4.40 (H_0_ = 1525 Gs) and two broader features shifted up-field to *g*_2_ = 1.70 (H_0_ = 3939 Gs) and *g*_3_ = 1.61 (H_0_ = 4150 Gs).^63,64^

The successful isolation of the rare heterobimetallic N_2_ bridging complex 1-U prompted us to investigate if other U(iii) mononuclear complexes could result in higher activation of the N–N bond. The addition of A to the U(iii) analogue with a bulkier amide, [U^III^{N(SiMe_2_Ph)_2_}_3_]^65^ showed no reaction in toluene solution at −40 °C, as confirmed by multinuclear NMR spectroscopy (Fig. S10 and S11†). Lack of reactivity of the complex [U{N(SiMe_2_Ph)_2_}_3_] with CO was also reported and was attributed to the arene–uranium interaction blocking CO binding and subsequent reductive coupling observed for [U^III^{N(SiMe_3_)_2_}_3_].^39^

We then decided to investigate how U(iii) tris–aryloxide complexes that are known to bind N_2_ in side-on mode would react with complex A. The U(iii) tris–aryloxide complex [U(O-2,6-^*t*^Bu_2_C_6_H_3_)_3_]^39^ was reported to reversibly bind and reduce N_2_ in apolar solvents leading to the isolation of a dinuclear side-on diazenido complex in low yield.^39^

**Scheme 2:**
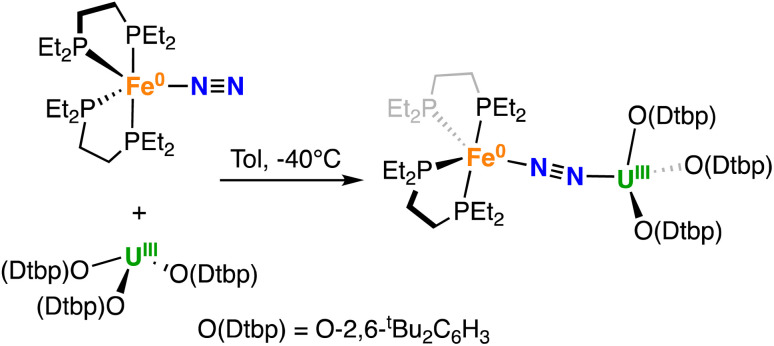
Synthesis of 2.

Gratifyingly, A reacted immediately with the U(iii) tris–aryloxide complex [U(O-2,6-^*t*^Bu_2_C_6_H_3_)_3_]^39^ yielding a new species, as confirmed by multinuclear NMR spectroscopy in d_8_-toluene. Dark brown-purple crystals of the heterobimetallic bridging dinitrogen complex [{Fe(depe)_2_}(μ-η^1^:η^1^-N_2_){U(O-2,6-^*t*^Bu_2_C_6_H_3_)_3_}], 2 were obtained in 76% yield (Scheme 2) from a concentrated Et_2_O solution at −40 °C.

The ^31^P {^1^H} NMR spectrum at −40 °C of isolated 2 shows a broad signal at *δ* = −17.9 ppm (Fig. S13†). Variable temperature ^31^P{^1^H} NMR spectroscopy (from −80 °C to 25 °C) indicates that the adduct 2 is favoured *versus* the dissociation into A and [U(O-2,6-^*t*^Bu_2_C_6_H_3_)_3_], suggesting a stronger interaction than for 1-U (Fig. S16†). Dissolution of 2 in THF results in its immediate dissociation, as confirmed by the ^31^P{^1^H} NMR spectrum, which shows immediate conversion to A at −40 °C (Fig. S14†).

The solid-state molecular structure of 2 (Fig. 1, right) is similar to that of complex 1-U. The U–O distances (2.205(6) Å average) are close to those found in the complex [U^III^(O-2,6-^*t*^Bu_2_C_6_H_3_)_3_] (U–O distance = 2.161 Å, avg.)^39^ and suggest the presence of a U centre in the +3 oxidation state. The U–N_2_ distance (2.474(5) Å) compares well with the distances reported for [U^III^(Cp*)_3_N_2_], (2.485(9) Å)^27^ and 1-U (2.498(4)Å). As observed also with 1-U, the Fe–N (1.752(5) Å) and Fe–P (2.22(2) Å) distances in 2 are almost unaffected by the coordination of the uranium centre to A (Fe–N distance = 1.749(7) Å and Fe–P distance = 2.223(8) Å avg.),^50^ in agreement with the presence of Fe(0). The N–N distance in the bridging N_2_ ligand (1.169(7) Å) is slightly longer than the one found in complex 1-U (1.150(7)Å), suggesting a similar degree of activation of the N_2_ bond. Interestingly, the reduced steric pressure exerted around the U centre by the aryloxide ligands in 2 compared to the HMDS in 1-U favours a slight bending of the N–N–U angle to 169.4(4)° (*cf.*1-U at 177.5(4)°), significant but still far from the values achieved by binding of the borane B(C_6_F_5_)_3_ to A (137.0°).^16^ Overall, the structural parameters in 2 support the formulation of the complex as a Fe(0)/U(iii)–N^0^_2_ (Table 1).

Further evidence for oxidation state is provided by the X-band EPR spectrum of powdered 2 at 6 K (Fig. S67†), that shows a complicated EPR spectrum with multiple signals that compare well with previously reported U(iii) complexes^63,64^ and with 1-U.

The solid-state IR spectra of 1-U and 2 (Fig. S73–S75†) show a *ν*(N≡N) band at 1833 cm^−1^ and 1820 cm^−1^, respectively, which indicates a higher degree of activation with respect to A (1955 cm^−1^),^50^ upon coordination to the U centre. The values observed for both complexes are lower than those previously reported for complex A upon binding to alkali metals (1903–1920 cm^−1^),^16^ and compare well with the values observed upon coordination of B(C_6_F_5_)_3_ or of [Fe^II^(^i^Pr_2_Tp)]^+^ to the N_2_ moiety from A (1830 and 1825 cm^−1^, respectively),^16^ further supporting their formulation as Fe(0)/U(iii) complexes bridged by a strongly polarized N^0^_2_ ligand.

As stronger N_2_ binding was reported for the bulkier aryloxide analogue [U^III^(O-2,4,6-^*t*^Bu_3_C_6_H_2_)_3_],^29^ its reactivity with A in toluene solution at −40 °C was also explored. Although ^31^P{^1^H} NMR of the reaction mixture at −40 °C shows partial consumption of A and the appearance of a resonance at *δ* = −15.7 ppm (Fig. S17†), similarly to what observed for 2, the very high solubility of the complexes prevented the isolation of any product.

Geometry optimization of complexes 1-U and 2 were carried out at the DFT level (B3PW91). The optimized geometry compares well with the experimental one. The U–N distance is well reproduced in both cases with a maximum deviation of 0.06 Å while the Fe–N and N–N bond distances are reproduced with a 0.01 Å accuracy. The computed U–N_2_Fe bond dissociation enthalpy for 1-U and 2 are −0.9 kcal mol^−1^ and −2.2 kcal mol^−1^, in line with the observation by ^1^H and ^31^P{^1^H} NMR spectroscopy of the products in equilibrium with the precursors. It is interesting to note that inclusion of dispersion has very little effect on the geometry (see ESI†) but leads to unrealistic bond dissociation energies (more than 30 kcal mol^−1^). This is due to the reported^66,67^ over-binding effect due to the dispersion correction. The uranium coordination to A was analysed using Natural Bonding Orbital (NBO). The Canonical Molecular Orbital (CMO) analysis (CMO tabulates the leading NBO contributions: bonding, nonbonding, or antibonding) of NBO 6.0 indicates that the HOMO-3 of complex 1-U corresponds to the interaction between the Fe–N_2_ backbonding orbital (3d on Fe and N_2_ π*), which is the HOMO of complex A (see ESI†), and the uranium 5f orbital. The Fe–N_2_–U interaction has some degree of covalency as shown by the U–N Wiberg Bond Index (WBI) of 0.52 (1-U) and 0.45 (2). This leads to a more polarized N–N bond than in complex A (−0.16/−0.08) as reflected by the natural charges of the two nitrogen of N_2_ in 1-U (N(U): −0.35 and N(Fe): −0.05) and in 2 (N(U): −0.37 and N(Fe): −0.02). This is somewhat reminiscent of the effect of boron coordination to Fe–N_2_ as reported by Szymczak^16^ and coworkers. Indeed, our CMO analysis indicates that the HOMO of complex A implies 12% contribution of N_2_ π* (18% was computed Szymczak^16^ and coworkers) and that the coordination to U results in a larger contribution of the N_2_ π* to the HOMO-3 (43% in 1-U and 48% in 2) which is somewhat similar to the 39% contribution found with the boron coordination in FeN_2_–B(C_6_F_5_)F.^16^ In the same way, the Fe contribution decreases to 30% (72% in complex A) with an uranium contribution of 26%. The unpaired spin density (see ESI†) is mostly localized at the uranium centre in line with the lack of reduction of N_2_. For the sake of comparison, the end-on coordination of N_2_ to the [U^III^{N(SiMe_3_)_2_}_3_] and [U^III^(O-2,6-^*t*^Bu_2_C_6_H_3_)_3_] complexes was investigated computationally. Some stable adducts were found with a very small coordination energy (see ESI†) for both complexes. However, in both cases, electron transfer from U(iii) to the N_2_ π*is observed to occur with concomitant formation of bimetallic complexes with a side-on coordination. This result is consistent with previous experimental reports leading to the isolation of a bimetallic side-on bridged N_2_ complex in the case of reaction with [U^III^(O-2,6-^*t*^Bu_2_C_6_H_3_)_3_], although N_2_ activation was not reported for [U^III^{N(SiMe_3_)_2_}_3_]. Therefore, these results show that the strong interaction between N_2_ and Fe led to a stronger end-on binding of N_2_ to U(iii) but end-on binding is not followed by electron transfer from U(iii) to N_2_. Dinitrogen reduction by U(iii) complexes usually involves side-on binding to two uranium centres^2,26,28,31,33,39,42–46,68–72^ but was also observed in an end-on N_2_ bridged heterobimetallic complex where two electron reduction of N_2_ was effected by combining Mo(iii) and U(iii) complexes that transfer one electron each to N_2_.^26^

### Fe–Ln(iii) dinitrogen complexes

**Scheme 3:**
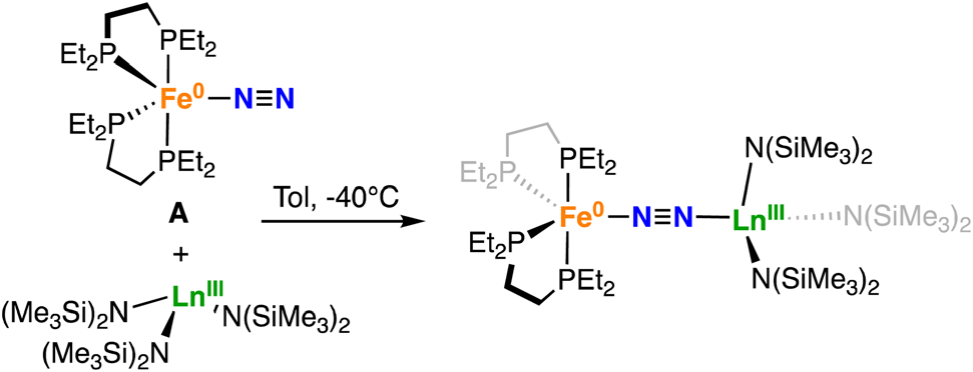
Synthesis of 1-Ln (Ln = Ce(iii), Sm(iii), Dy(iii), Tm(iii)).

The successful isolation of two heterobimetallic Fe–U complexes featuring an activated, end-on bound dinitrogen incited us to probe the possibility of end-on binding of lanthanides in different oxidation states. At first we targeted the synthesis of the analogue complex of 1-U containing Ce(iii), which is the Ln(iii) with a ionic radius closer to U(iii) (1.010 Å for Ce(iii) and 1.025 Å for U(iii))^73^ and generally shows very little covalency in its molecular complexes. The addition of a toluene solution of [Ce^III^{N(SiMe_3_)_2_}_3_] to an orange-yellow toluene solution of A at 25 °C, resulted in a dark orange-red solution. ^1^H and ^31^P{^1^H} NMR spectroscopy at low temperatures (from −80 °C to −40 °C) showed the formation of a new species. Orange-yellow crystals of the heterobimetallic dinitrogen complex [{Fe(depe)_2_}(μ-η^1^:η^1^-N_2_){Ce{N(SiMe_3_)_2_}_3_}], 1-Ce were obtained from a saturated toluene solution at −40 °C over the course of 2 days in 83% yield (Scheme 3).

In order to evaluate the effect of Ln(iii) Lewis acidity, which is reported and measured to decrease steadily from La(iii) to Lu(iii) concomitant to the ionic radii contraction,^74–79^ on the activation of the μ-η^1^:η^1^-bound dinitrogen we pursued the synthesis of heterobimetallic complexes of smaller and more Lewis acidic Ln(iii) (Scheme 3). The Sm(iii), Dy(iii) and Tm(iii) complexes (1-Sm, 1-Dy, and 1-Tm respectively) were isolated in 77%, 87% and 42% yield respectively from the reaction of A with [Ln^III^{N(SiMe_3_)_2_}_3_] (Ln = Sm, Dy) in toluene solution and in hexane solution for the Tm(iii) congener at −40 °C. Attempts to crystallise 1-Tm from toluene only led to the isolation of colourless needles of [Tm^III^{N(SiMe_3_)_2_}_3_]. This contrasts with the analogous complexes of the larger lanthanides, which crystallize readily from toluene. The more facile dissociation of the Tm complex observed in toluene compared to those of the larger lanthanides is probably due to the smaller size of Tm which increases steric repulsions between A and [Tm^III^{N(SiMe_3_)_2_}_3_].

The ^31^P{^1^H} NMR spectrum at −40 °C of a toluene solution of isolated 1-Ce shows a broad signal at *δ* = 73.8 ppm (Fig. S24†). Variable temperature ^31^P{^1^H} NMR spectroscopy (from −80 °C to 25 °C) suggests that the formation of the adduct 1-Ce is reversible at higher temperatures (Fig. S25†). Indeed, at 25 °C the ^1^H NMR spectrum shows a resonance at −3.34 ppm, which matches perfectly the signal observed for the starting material, [Ce^III^{N(SiMe_3_)_2_}_3_], while the ^31^P{^1^H} NMR spectrum at 25 °C is silent, suggesting a fast exchange between the adduct and the precursors (Fig. S22 and S23†). Dissolution of 1-Ce in THF results in the disruption of the adduct, as confirmed by the ^31^P{^1^H} NMR spectrum at −40 °C, which shows immediate conversion to A (Fig. S26†).

**Fig. 2:**
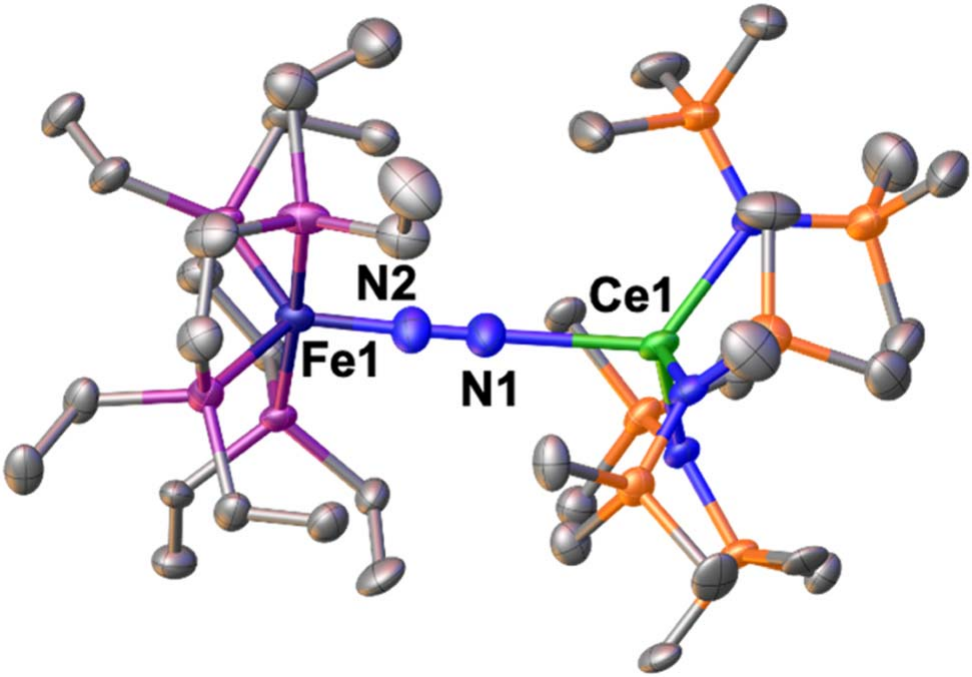
Solid-state molecular structure of 1-Ce with 50% probability ellipsoids. Colour code: cerium (light green), phosphorus (purple), iron (midnight blue), carbon (grey), silicon (orange). Hydrogen atoms were omitted for clarity.

The solid-state molecular structure of 1-Ce (Fig. 2) shows a Ce–Fe heterometallic complex, with an end-on N_2_ group bridging the Ce and the Fe centres. The Ce centre is tetracoordinated in a pseudo tetrahedral geometry by the nitrogen atoms of the amide ligands and one of the nitrogen atoms from the (μ-η^1^:η^1^-N_2_) ligand. The Ce–N_amide_ distances (2.38(1) Å average) are similar to those of the precursor [Ce^III^{N(SiMe_3_)_2_}_3_] (Ce–N_amide_ distance = 2.320(3) Å).^80^ The Fe–N (1.738(8) Å) and N–N (1.13(1) Å) distances are similar to those observed in A (Fe–N distance = 1.749(7) Å and N–N distance = 1.139(2) Å), while featuring a long Ce–N_2_ distance of 2.54(2) Å (Table 2).

**Table 2:** Mean values of selected bond lengths (Å) and angles (°) in the complexes 1-Ln, 1*-Ln, 1-U, 2, 3 and A^50^

Complex	Fe–N	Fe–P	M–N	N–N	N–N–E
A	1.749(7)	2.223(8)	—	1.139(2)	—
1-U	1.739(4)	2.223(3)	2.479(3)	1.150(7)	177.5(4)
2	1.752(5)	2.22(2)	2.474(5)	1.169(7)	169.4(4)
1-Ce	1.738(8)	2.221(5)	2.544(8)	1.13(1)	178.0(8)
1-Sm	1.756(6)	2.216(4)	2.480(6)	1.157(8)	174.9(5)
1-Dy	1.747(6)	2.227(4)	2.396(6)	1.168(7)	174.3(5)
1-Tm	1.747(3)	2.228(2)	2.345(3)	1.170(4)	175.2(3)
1*-Yb	1.759(2)	2.206(1)	2.446(2)	1.149(3)	170.6(2)
1*-Sm	1.748(3)	2.201(2)	2.565(4)	1.128(5)	174.0(4)
3	1.757(5)	2.206(4)	2.568(5)	1.140(7)	171.2(5)
	1.759(5)	2.197(4)	2.674(5)	1.141(7)	173.6(5)

The structure shows two smaller (98.2(2)° and 103.2(2)°) and one large (120.5(2)°) N1–Ce–N_amide_ angles most likely as a result of the steric bulk of the bound complex A. In contrast, the Fe–P (2.221(5) Å) distances are almost unaffected by the coordination to the cerium centre relative to those from A (Fe–P distance = 2.223(8) Å avg.),^50^ suggesting that the oxidation state of the iron centre is unchanged. The structural parameters in 1-Ce support the formulation of the complex as Fe(0)/Ce(iii) (Table 2).

1-Ce provides the first isolated example of an end-on bound dinitrogen complex of cerium, while two examples of homobimetallic cerium(iii) complexes containing side-on bridging dinitrogen (μ-η^2^:η^2^-N_2_)^2−^ were reported.^81,82^ Side-on bridged complexes with the formula [{(N′′)_3_M}_2_(μ-η^2^:η^2^-N_2_)]^2−^ (N′′=N(SiMe_3_)_2_) have also been isolated for a broad range of lanthanides ions by different synthetic routes.^83–85^ The Ce–N_amide_ distance in 1-Ce (2.38(1) Å) compares well with that found in the [{((Me_3_Si)_2_N)_2_Ce(crypt-κ^2^-O,O′)}_2_(μ-η^2^:η^2^-N_2_)] complex^82^ (2.410(1) Å), while the N–N distance in 1-Ce (1.13(1) Å) is shorter than that found in the dimetallic (μ-η^2^:η^2^-N_2_)^2−^ complex (1.233(4) Å), suggesting a lower degree of activation of the N_2_ bridging ligand in 1-Ce.

The solid-state structures of 1-Sm (Fig. S62†), 1-Dy (Fig. S63†) and 1-Tm (Fig. S64†) present overall similar coordination environments as found for 1-Ce but feature increasingly longer N–N bond distances of 1.157(8), 1.168(7), and 1.170(4) Å, respectively, that align well with their increased Lewis acidity (Table 2).

In a solid-state KBr matrix, the IR spectra of 1-Ln (Ln = Ce, Sm, Dy and Tm) (Fig. S76–S80†) showed one N–N sharp stretch with stretching frequencies ranging from 1849 cm^−1^ for Ce to 1837 cm^−1^ for Tm (Table 3). The *ν*(N≡N) band undergoes a slight bathochromic shift as the Ln(iii) ionic radius decreases and the Lewis acidity increases going from Ce(iii) to Tm(iii) (Table 3). A similar trend was also observed by Szymczak for the coordination of organic acids with increasing Lewis acidity to complex A.^16^

**Table 3:** IR *ν*(N≡N) bands and N–N bond length from XRD for 1-M, 2, 3, A^50^ and previously reported complexes^16^

Compound	*ν*(N≡N)	N–N (exp)	N–N (calc.)
A	1955 cm^−1^	1.139(2) Å	1.14 Å
1-U	1833 cm^−1^	1.150(7) Å	1.16 Å
2	1820 cm^−1^	1.169(7) Å	1.17 Å
1-Ce	1849 cm^−1^	1.13(1) Å^a^	1.16 Å
1-Sm	1842 cm^−1^	1.157(8) Å	1.16 Å
1-Dy	1839 cm^−1^	1.168(7) Å	1.16 Å
1-Tm	1837 cm^−1^	1.170(4) Å	
1*-Yb	1874 cm^−1^	1.149(3) Å	1.15 Å
1*-Sm	1888 cm^−1^	1.128(5) Å^a^	1.15 Å
3	1888 cm^−1^	1.140(7) Å	1.15 Å
	1896 cm^−1^	1.141(7) Å	1.15 Å
[Fe(depe)_2_(μ-N_2_)B(C_6_F_5_)_3_]	1825 cm^−1^	1.186(3) Å	
[Fe(depe)_2_(μ-N_2_)Fe(iPr_2_Tp)] [BArF_4_]	1830 cm^−1^	1.177(5) Å	

a The short distances measured for these compounds are not consistent with IR or calculated values and are probably the results of systematic errors associated with standard X-ray measurements.^86,87^

Overall, the N–N bond lengths and the *ν*(N≡N), suggest that a similar degree of N–N bond activation is found in the complexes of uranium(iii) and dysprosium(iii) 1-U and 1-Dy despite the difference in ionic radii. The higher degree of N–N bond activation observed for U(iii) compared to Ce(iii) is likely to be due to the presence of a small U–N_2_ back-bonding contribution, as was suggested by the calculations (see above).

Geometry optimizations were carried out on complexes for complexes 1-Ln similarly to what reported for the uranium complexes. Small core but also f-in-core relativistic pseudopotential (RECP) geometry optimization were carried out and the effects of the dispersion corrections were also investigated. Here again, the inclusion of dispersion effects modifies the geometry and leads to unrealistic strong binding of the Ln(iii) complexes to the iron complex A (see ESI†). The optimized structures of 1-Ln compare well with the experimental ones. Interestingly, a good reproduction of experimental structures is also found when using f-in-core RECPs, that are adapted to a given oxidation state and therefore do not allow any back donation from the lanthanide. The latter is further evidenced by the CMO, that clearly shows that the Fe–N_2_ backbonding orbital (HOMO) has no implication of any orbital from the lanthanide centre (see ESI†).§§In the case of complex 1-Sm, a small f-orbital contribution was found in the HOMO which could be associated with the f^6^ configuration of Sm but this is unique in the series and cannot be discussed further.

Alike the uranium complexes, the coordination to the lanthanide centre induces a larger polarization of the N–N bond (−0.30/−0.04 as an average of the 1-Ln series) than in complex A but lower than in 1-U and 2. Such reduced polarization of the bridging N_2_ in the Ln(iii) complexes can be attributed to the absence of backdonation found in the lanthanide complexes, that correlates well with the perfect match between small core and f-in-core structures. The reduced polarization is also attributed to the lower covalency in the Ln–N bond with respect to U–N, as reflected by the Ln–N WBI of 0.2 or even less.

Within the lanthanide series, the Ln–N WBI is slightly increasing from 0.22 for Ce up to 0.27 for Dy with a concomitant decrease of the N–N WBI from 2.33 to 2.28, while the Fe–N WBI remains constant to 0.75. This is in line with the increasing Lewis acidity in the series since no backdonation is found from Ln to N_2_.

The Ln–N bond is mainly ionic as expected from literature studies^88–90^ and not observed at the NBO nor CMO levels but only at the second order donor–acceptor level (donation from the nitrogen σ lone pair to empty df hybrid orbital of Ln of ≈20 kcal mol^−1^).

### Fe–Ln(ii) dinitrogen complexes

**Scheme 4:**
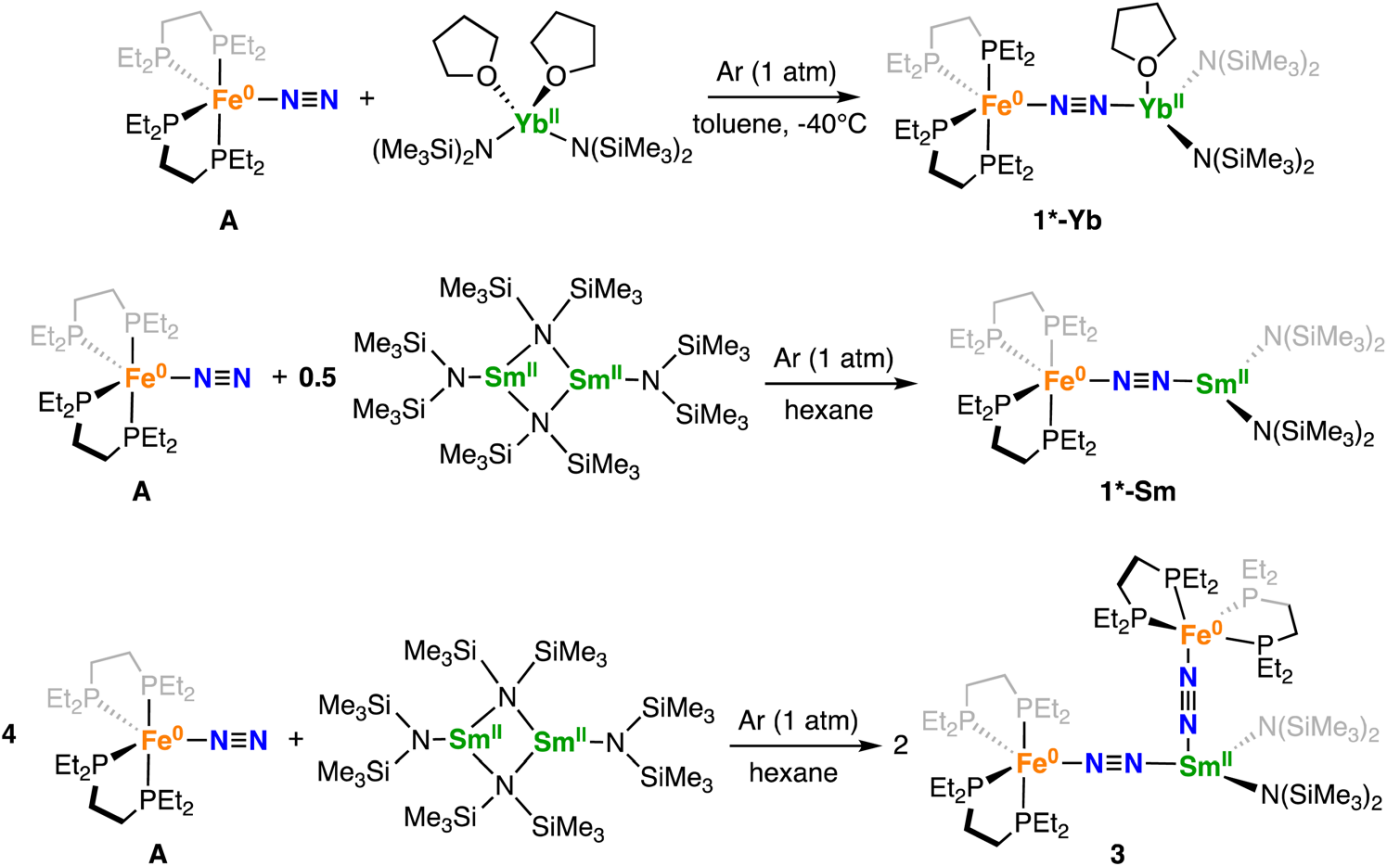
Synthesis of 1*-Ln and 3.

**Fig. 3:**
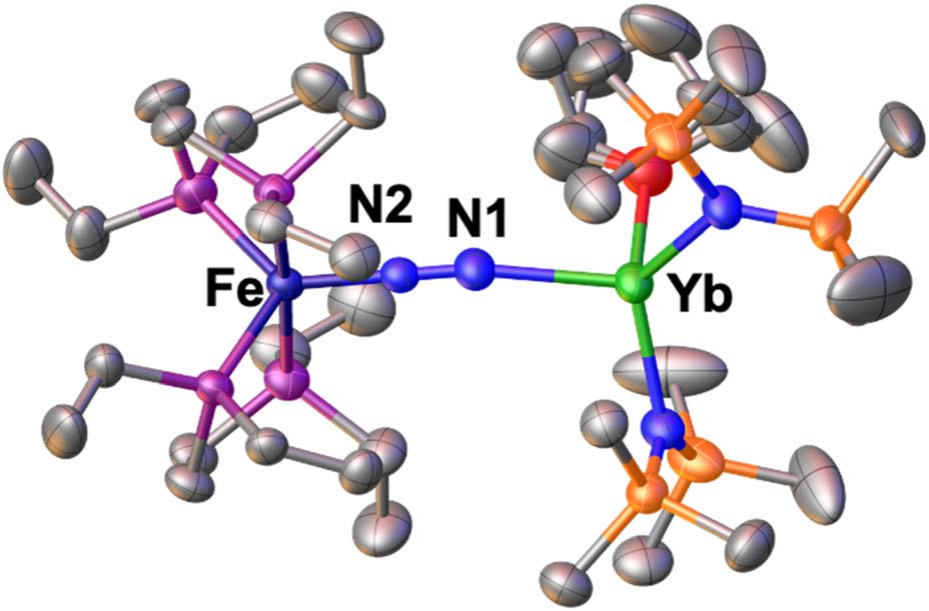
Solid-state molecular structure of 1*-Yb with 50% probability ellipsoids. Colour code: ytterbium (light green), phosphorus (purple), iron (midnight blue), carbon (grey), silicon (orange). Hydrogen atoms were omitted for clarity.

Incited by the results obtained with Ln(iii) we decided to investigate if heterobimetallic Fe–Ln(ii) complexes could be in reach. Interestingly, A reacts with [Yb^II^{N(SiMe_3_)_2_}_2_(thf)_2_] to afford the bimetallic N_2_ bridged complex [{Fe(depe)_2_}(μ-η^1^:η^1^-N_2_){Yb{N(SiMe_3_)_2_}_2_(Et_2_O)_0.4_(thf)_0.6_}], 1*-Yb as supported by a shift in the ^31^P{^1^H} NMR at 81.3 ppm in toluene solution at −40 °C (Fig. S39†). Complex 1*-Yb can be isolated in 73% yield from a concentrated Et_2_O solution at −40 °C (Scheme 4, top). The solid-state molecular structure of 1*-Yb (Fig. 3) shows a Yb–Fe heterobimetallic complex, with an end-on N_2_ group bridging the Yb and the Fe centres. The Yb centre is tetracoordinated in a pseudo tetrahedral geometry by the nitrogen atoms of the amide ligands, one of the nitrogen atoms from the (μ-η^1^:η^1^-N_2_) ligand and an oxygen atom from a solvent molecule (the position is partially occupied by 0.4 molecules of Et_2_O and 0.6 molecules of THF). The Yb–N_amide_ distances (2.352(3) Å average) are similar to those found in the precursor [Yb^II^{N(SiMe_3_)_2_}_2_(thf)_2_] (Yb–N_amide_ distance = 2.35(2) Å).^91^

The Fe–N (1.759(2)Å) and Fe–P (2.206(1) Å) distances are almost unaffected by the coordination to the ytterbium centre from A (Fe–N distance = 1.749(7) Å and Fe–P distance = 2.223(8) Å avg.),^50^ suggesting the presence of a Fe centre in the 0 oxidation state. The N–N distance (1.149(3) Å) and IR stretch (1874 cm^−1^) suggest a low degree of N_2_ activation. The reduced steric profile of the Yb fragment, arising from the presence of only two HMDS ligands, allows a comparatively shorter N–N–Yb angle of 170.6(2)°, in similar fashion to what observed for 2 compared to 1-U. A short (92.2(1)°) O–Yb–N angle is also notable. The remainder metrical parameters are consistent with an Fe(0)–Yb(ii)–N^0^_2_ formulation.

We then continued the study on Ln(ii) complexes by investigating the interaction of the [Sm^II^{N(SiMe_3_)_2_}_2_] complex with A. The use of Sm(ii) as an electron source, under the form of a simple iodide salt or in more sophisticated coordination complexes, has led to some of the highest yields and efficiencies for the catalytic transformation of N_2_ to NH_3_ or N_2_H_4_ over the last 10 years.^55–58^

Despite the growing use of Sm(ii) as an electron source for (electro)/catalytical reduction of dinitrogen, the active species in these transformations could not be unambiguously identified so far, although in a recent work Peters and collaborators proposed end-on binding of Sm(ii) to a Fe–N_2_ complex as an intermediate species for the conversion of N_2_ into N_2_H_4_.^57^ Following the isolation of 1*-Yb, we pursued the synthesis of an analogous Sm(ii) complex. The addition of an orange toluene solution of A to a dark violet toluene solution of the solvent-free [Sm{N(SiMe_3_)_2_}_2_]_2_ complex resulted in the consumption of the starting material and the formation of two different species. The ^31^P{^1^H} NMR spectrum of the reaction mixture at −40 °C shows two broad resonances at 125.1 ppm (major) and 113.2 ppm (minor) (Fig. S53†). However, crystallization attempts only resulted in the isolation of [Sm{N(SiMe_3_)_2_}_3_] and 1-Sm.

The addition of a 1 equiv. of A to 0.5 equiv. of the solvent-free [Sm{N(SiMe_3_)_2_}_2_]_2_ in cyclohexane at 25 °C resulted in a dark brown-yellow solution. Multinuclear NMR spectroscopy shows the consumption of the starting materials and the formation of a new species. The ^31^P{^1^H} NMR spectrum (Fig. S43†) at 25 °C shows a single resonance at 113.3 ppm. Brown-green crystals of [{Fe(depe)_2_}(μ-η^1^:η^1^-N_2_){Sm{N(SiMe_3_)_2_}_2_}] 1*-Sm could be obtained from slow evaporation of a saturated hexane solution over the course of 12 h at −40 °C in 83.6% yield (Scheme 4, middle).

**Fig. 4:**
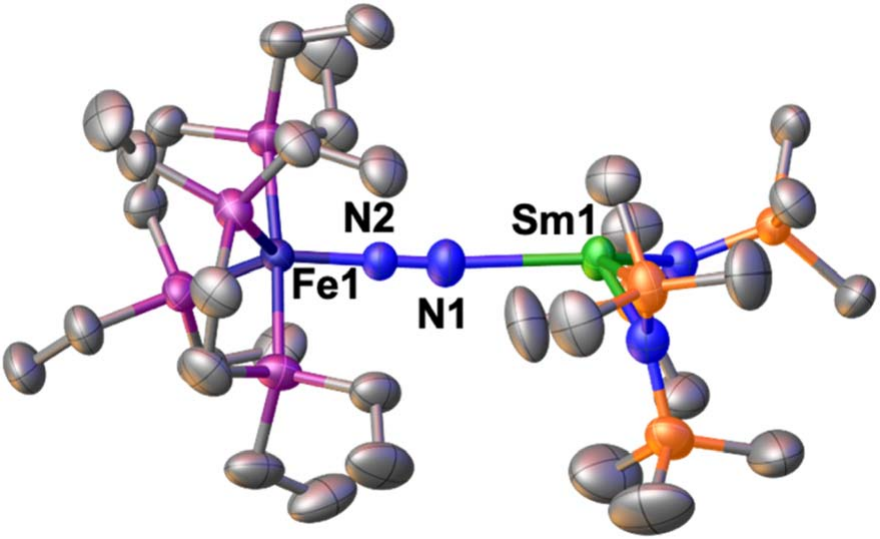
Solid-state molecular structure of 1*-Sm with 50% probability ellipsoids. Colour code: samarium (light green), phosphorus (purple), iron (midnight blue), carbon (grey), silicon (orange). Hydrogen atoms were omitted for clarity.

The solid-state molecular structure of 1*-Sm (Fig. 4) shows a Sm–Fe heterometallic complex, with an end-on N_2_ group bridging the Sm and the Fe centres. The Sm centre is tricoordinated by the nitrogen atoms of the amide ligands and one of the nitrogen atoms from the (μ-η^1^:η^1^-N_2_) ligand. The Sm–N_amide_ distances (2.421(4) Å average) are similar to those reported for the complex [Sm^II^{N(SiMe_3_)_2_}_2_(thf)_2_] (Sm–N_amide_ average distance = 2.43(1) Å).^92^ The Fe–N (1.748(3)Å) and Fe–P (2.201(2) Å) distances are almost unaffected by the coordination of the samarium centre to A (Fe–N distance = 1.749(7) Å and Fe–P distance = 2.223(8) Å avg.),^50^ suggesting that the oxidation state of iron does not vary. All metrical parameters are consistent with the Fe(0)-Sm(ii)–N^0^_2_ formulation (Table 2).

The N–N distance (1.128(5) Å) and IR stretch (1888 cm^−1^) suggest a low degree of N_2_ activation, lower than what observed for 1*-Yb, which is consistent with the lower Lewis acidity of Sm(ii) compared to Yb(ii). Although the complex [Sm^II^{N(SiMe_3_)_2_}]_2_ was not reported to react with N_2,_ examples of N_2_ activation by Sm(ii) complexes supported by cyclopentadienyl or calix-tetrapyrrole ligands were reported to yield diazenido bridged [Sm(iii)_2_-(μ-η^2^:η^2^-N_2_)]^2−^ species.^93–95^

The molecular structure of 1*-Sm (Fig. 4) shows a Sm(ii) centre with an open site in the coordination sphere, which prompted us to investigate the possibility of coordinating a second N_2_–Fe complex to the metal centre.

Thus, the addition of 1 equiv. of A to a cyclohexane solution of 1*-Sm resulted in the consumption of the starting materials and the formation of a new species with a resonance at *δ* = 125.3 ppm in the ^31^P{^1^H} NMR spectrum at 25 °C. The reaction of 4 equiv. of A with 1 equiv. of [Sm{N(SiMe_3_)_2_}_2_]_2_ afforded dark green crystals of [{Fe(depe)_2_(μ-η^1^:η^1^-N_2_)}_2_{Sm{N(SiMe_3_)_2_}_2_}], 3 in 70% yield by slow evaporation of a saturated hexane solution over the course of 12 h at −40 °C (Scheme 4, bottom).

**Fig. 5:**
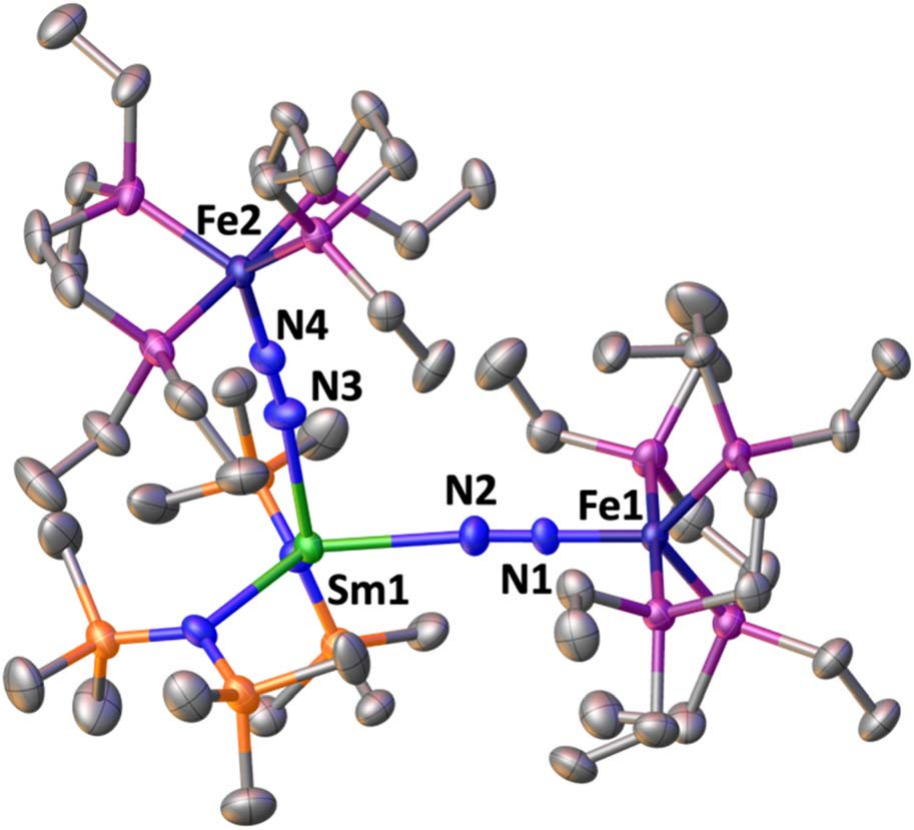
Solid-state molecular structure of 3 with 50% probability ellipsoids. Colour code: samarium (light green), phosphor (purple), iron (midnight blue), carbon (grey), silicon (orange). Hydrogen atoms were omitted for clarity.

The solid-state molecular structure of 3 (Fig. 5) shows a Sm–Fe heterometallic complex, with two end-on N_2_ groups bridging the Sm centre to two different Fe centres. The Sm centre is tetracoordinated in a distorted tetrahedral geometry by the nitrogen atoms of the amide ligands and the nitrogen atoms from the (μ-η^1^:η^1^-N_2_) ligands. The Sm–N_amide_ distances (2.452(7) Å average) are similar to those reported for the complex [Sm^II^{N(SiMe_3_)_2_}_2_(thf)_2_] (Sm–N_amide_ distance = 2.43(1) Å)^92^ and those observed for 1*-Sm (2.42(1) Å average). The Fe–N (1.757(5) and 1.759(5) Å) and Fe–P (2.206(4) and 2.197(4) Å) distances are almost unaffected by the coordination of Sm(ii) to A (Fe–N distance = 1.749(7) Å and Fe–P distance = 2.223(8) Å avg.).^50^ Overall the metrical parameters are consistent with an Fe_2_(0)Sm(ii)(N_2_)^0^_2_ formulation. The N–N distances (1.140(7) and 1.141(7) Å) and IR stretches (1888 and 1896 cm^−1^) suggest a similar low degree of N_2_ activation as found in 1*-Yb and 1*-Sm.

The isolated Fe–N_2_–Ln(ii) complexes are remarkable for two different reasons: (i) they demonstrate the possibility of end-on dinitrogen binding to lanthanides in the absence of very bulky ligands, a requirement that was believed essential to implement end-on binding;^47,49^ (ii) they provide the first examples of N_2_ binding by a Yb(ii) complex and of the binding of two N_2_ molecules by a Sm(ii) complex.

Finally, calculations were also carried out on the complexes 1*-Sm, 1*-Yb and 3 to investigate the influence of the lanthanide oxidation state on binding and activation of the end-on N_2_ in A. Once again, alike the trivalent lanthanide complexes, the optimized geometries are in good agreement with the experimental ones (see ESI†). The maximum deviation is obtained for the Yb–N distance with 0.07 Å. As for the Ln(iii) complexes, the CMO indicates that the Fe–N_2_ backbonding orbital does not involve significant contribution from the Ln(ii) centre (see ESI†). Moreover, the Ln–N interaction is even less covalent than what found for Ln(iii) species (Ln–N WBI of 0.2). Interestingly, the electrostatic Ln–N interaction is weaker for Ln(ii) than Ln(iii) since the Ln(ii) charge is around 1.4–1.5. This is in line with the lower Lewis acidity of the Ln(ii). The Ln(ii)–N bonding interaction is favoured by the polarization of the N–N when bonded to Fe, that allows an electrostatic interaction between Ln(ii) and polarized N_2_, that is not possible with free N_2_. In complex 3, the bonding appears to be quite similar to what found for 1*-Sm. Indeed, the CMO does not indicate any Sm(ii) contribution to the Fe–N_2_ backbonding (see ESI†). The two Sm–N are even slightly less covalent than that found in 1*-Sm (0.17 *vs.* 0.18). The individual electrostatic interaction between Sm(ii) and each N_2_ molecule is weaker than in 1*-Sm as reflected by the lower positive charge at Sm (1.48 *vs.* 1.51) and the lower negative charge of the nitrogens (−0.28 *vs.* −0.31). However, the presence of two electrostatic interactions in the complex induces some extra-stability for complex 3.

## Conclusions

In summary, we were able to isolate a range of end-on bridged dinitrogen complexes of uranium and lanthanides by reacting aryloxide and amide complexes of U(iii) or amide Ln(iii) and Ln(ii) complexes with the Fe dinitrogen complex [Fe(depe)_2_(N_2_)], A. All complexes were found to be stable in apolar solvents but dissociated readily in polar solvents to yield the precursor components. It is remarkable that end-on bridging dinitrogen binding is promoted by the Fe–N_2_ species for U(iii), Ln(iii) and Ln(ii) complexes which were not found previously to react with free N_2_, as a result of the higher nucleophilicity of the Fe–bound N_2_. Binding of the Ln(iii) ions results in increased activation of the N–N bond with their increasing Lewis acidity ranging from cerium to thulium. End-on bridging of dinitrogen is also observed in the absence of bulky ligands at the lanthanide(ii) centre. The synthetic method used here allowed to isolate unprecedented end-on bridged dinitrogen complexes of divalent lanthanides which provide relevant models for the species involved in the catalytical reduction of dinitrogen by Fe/Sm(ii) systems. Computational studies showed an essentially electrostatic interaction of the end-on bridging N_2_ with both Ln(iii) and Ln(ii) complexes, with the degree of N_2_ activation correlating with their Lewis acidity. In contrast, a stronger back-bonding covalent contribution to the U(iii)–N_2_Fe bond was identified by computational studies. Computational studies also suggest that end-on binding of N_2_ to U(iii) and Ln(ii) complexes is favoured for the iron–bound N_2_ compared to free N_2_, probably due to the higher polarization. Reduction of N_2_ by U(iii) and Ln(ii) is not favoured in these heterobimetallic complexes, but binding of the U(iii), Ln(iii) and Ln(ii) leads to a higher polarization of the N–N bond. It can be anticipated that higher activation of the end-on bridging N_2_ may be in reach with different combinations of d–f complexes.

## Data availability

Synthetic details, analytical data including depictions of all spectra and coordinate data of all computationally optimised species, are documented in the ESI.† Crystallographic data is made available *via* the CCDC. The data that support the findings of this study are openly available in the Zenodo repository at https://doi.org/10.5281/zenodo.10910645.

## Author contributions

N. J. and J. M. designed and carried out all the experiments and analyzed the data; R. A. K. S. prepared and characterized complex 1-Tm. M. M. designed and supervised the project; T. R. and L. M. carried out the computational study; R. S. measured and analyzed the X-ray data; J. S. provided complex A and participate to designing the project. N. J., L. M., and M. M. wrote the manuscript with contributions of all authors, and all authors have given approval for the final version of the manuscript.

## Conflicts of interest

There are no conflicts to declare.

## Supplementary Material

SC-015-D4SC01050G-s001

SC-015-D4SC01050G-s002
