# Supplementary material for: Iron promoted end-on dinitrogen-bridging in heterobimetallic complexes of uranium and lanthanides
Source: Chem Sci. 2024 Apr 2;15(18):6842–52. doi: 10.1039/d4sc01050g (PMC11077558; doi:10.1039/d4sc01050g)
Supplement: SC-015-D4SC01050G-s001 [file SC-015-D4SC01050G-s001.pdf]

Electronic Supplementary Material (ESI) for Chemical Science.
This journal is © The Royal Society of Chemistry 2024

ELECTRONIC SUPPORTING INFORMATION

Iron promoted end-on dinitrogen-bridging in heterobimetallic complexes of uranium and lanthanides

Nadir Jori,[a§] Juan J. Moreno,[a,c§] R. A. Keerthi Shivaraam,[a] Thayalan Rajeshkumar,[b] Rosario Scopelliti,[a] Laurent Maron*,[b] Jesús Campos,[c] Marinella Mazzanti*[a]

[a]Group of Coordination Chemistry Institut des Sciences et Ingénierie Chimiques, École Polytechnique Fédérale de Lausanne (EPFL), 1015 Lausanne, Switzerland. E-mail: marinella.mazzanti@epfl.ch

[b] Laboratoire de Physique et Chimie des Nano-objets, Institut National des Sciences Appliquées, 31077 Toulouse, Cedex 4, France; E-mail: laurent.maron@irsamc.ups-tlse.fr

[c] Instituto de Investigaciones Químicas (IIQ), Departamento de Química Inorgánica and Centro de Innovaciónen Química Avanzada (ORFEO−CINQA), Consejo Superior de Investigaciones Científicas (CSIC) and Universidad de Sevilla, 41092 Sevilla, Spain.

*To whom correspondence should be addressed.

Table of Contents

Table of Contents 2
Experimental Procedures 3
NMR Spectra 7
X-ray crystallography 41
EPR data 47
IR data 54
Computational Details 60
Optimized Coordinates 73
References 124

Experimental Procedures

General Considerations Unless otherwise noted, all manipulations were carried out at ambient temperature under an inert argon or nitrogen atmosphere using Schlenk techniques and an MBraun glovebox equipped with a purifier unit. The water and oxygen levels were always kept at less than 0.1 ppm. Glassware was dried overnight at 140 °C before use.

NMR experiments were carried out using NMR tubes adapted with J-Young valves. NMR spectra were recorded on a Bruker 400 MHz spectrometers. NMR chemical shifts are reported in ppm with solvent as internal reference.

Elemental analyses were performed under nitrogen using a Thermo Scientific Flash 2000 Organic Elemental Analyzer at the Institute of Chemistry and Chemical Engineering at EPFL.

Starting materials Unless otherwise noted, reagents were purchased from commercial suppliers and used without further purification. Anhydrous solvents were purchased from Aldrich and further distilled from K/benzophenone (THF, Et_2O, toluene). Deuterated solvents for NMR spectroscopy (d_8-thf, d_8-toluene) were purchased from CortecNet, freeze-degassed and distilled over K/benzophenone.
Depleted uranium was purchased from Ibilabs, Florida, USA. $[Fe(depe)_2(N_2)]$, **A** , depe = $Et_2PCH_2CH_2PEt_2$,[1] $[U^{III}(C_5Me_4H)_3]$,[2] $[U^{III}\{N(SiMe_3)_2\}_3]$,[3] $[U^{III}\{N(SiMe_2Ph)_2\}_3]$,[4] $[U(ODtbp)_3]$, ODtbp = O-2,6-$^tBu_2C_6H_3$,[5] $[U(OTtbp)_3]$, OTtbp = O-2,4,6-$^tBu_3C_6H_2$,[5] $[Ln^{III}\{N(SiMe_3)_2\}_3]$ (Ln= Ce, Sm and Dy),[6] $[Tm^{III}\{N(SiMe_3)_2\}_3]$,[7] $[Sm^{II}\{N(SiMe_3)_2\}_2]_2$,[8] and $[Yb^{II}\{N(SiMe_3)_2\}_2(thf)_2]$[9] were prepared according to the published procedure.

EPR. EPR experiments were recorded with a Bruker Elexsys E500 spectrometer working at 9.4 GHz frequency with an Oxford ESR900 cryostat for 4-300 K operations.

FT-IR spectra were recorded with a Perkin Elmer 1600 Series FTIR spectrophotometer flushed with N_2.

Syntheses
In general the synthesis of the dinitrogen complexes was performed by mixing the components at low temperature and let standing at -40°C for 12-48 hours. Carrying out the reaction (mixing the components) at room temperature leads to the same outcome, but isolation of the complex requires low temperature. In general low temperature reactions are preferred when the precursor (U(III) and Sm(II)) are thermally unstable.

Reaction of A with $[U^{III}(C_5Me_4H)_3]$ at −40 °C in toluene. A cold (−40 °C) dark brown d_8-toluene solution (0.6 mL) of **$[[U^{III}(C_5Me_4H)_3]$** (8.0 mg, 0.013 mmol, 1 equiv) in was added to orange solid **A** (6.6 mg, 0.013 mmol, 1 equiv) under Ar, resulting in a brown-reddish solution. The $^{31}P\{^1H\}$ NMR spectrum at −40 °C and 25°C of the reaction mixture (Figures S1 and S3) shows the sharp resonance at δ = 84.6 ppm (s) corresponding to **A**. The ^1H NMR spectrum shows the signals of the two starting complexes, indicating that no binding is observed (Figure S2).

Synthesis of $[\{Fe(depe)_2\}(\mu\text{-}\eta^1\text{:}\eta^1\text{-}N_2)(U\{N(SiMe_3)_2\}_3)]$, 1-U. A cold (−40 °C) orange-yellow solution of **A** (34.0 mg, 0.0684 mmol, 1 equiv) in toluene (1.0 mL) was added to cold (−40 °C) dark purple toluene solution (1.5 mL) of $[U^{III}\{N(SiMe_3)_2\}_3]$ (49.2 mg, 0.0684 mmol, 1 equiv). The reaction mixture was kept at −40 °C for 48 h. The supernatant was carefully pipetted out, affording 56.5 mg (68% yield) of analytically pure dark, crystalline material. X-ray quality crystals of **1-U** were obtained from a concentrated toluene solution at −40 °C.
Anal. Calcd for $[\{Fe(depe)_2\}(\mu\text{-}\eta^1\text{:}\eta^1\text{-}N_2)(U\{N(SiMe_3)_2\}_3)]$ $C_{38}H_{102}FeN_5P_4Si_6U$: C: 37.55%; H: 8.46%; N: 5.76%. Found: C: 37.37%; H: 8.34%; N: 5.73%.

^1H NMR (400 MHz, d_8-toluene, 233 K): δ = 2.12 (s), 1.64 (br), 1.32 (br), −4.02 (br), −5.41 (br), −6.11 (br), −7.12 (br), −11.21 (br), −12.77 (br) (Figure S5).
$^{31}P\{^1H\}$ NMR (162 MHz, d_8-toluene, 233 K): δ = −25.7 (br) (Figure S6).

IR spectrum (KBr pill): ν(N≡N) = 1833 cm^{-1} (Figure S73)

Reaction of A with [U^{III}{N(SiMe$_2$Ph)$_2$}$_3$]at −40 °C in toluene. A cold (−40 °C) dark violet solution of [U^{III}{N(SiMe$_2$Ph)$_2$}$_3$] (11.4 mg, 0.0104 mmol, 1 equiv) in d$_8$-toluene (0.6 mL) was added to orange solid **A** (5.2 mg, 0.0104 mmol, 1 equiv) under Ar at −40 °C, resulting in a dark solution. The $^{31}P\{^1H\}$ NMR spectrum at −40 °C of the reaction mixture (Figure S10) shows a sharp resonance at δ = 84.5 ppm (s) corresponding to **A**. However, increasing the temperature up to 50 °C led to gradual broadening of the ^{31}P resonance, suggestive of an interaction with the paramagnetic U center.

Synthesis of [{Fe(depe)$_2$}(μ-η^1:η^1-N$_2$)(U{ODtbp}$_3$)], 2. A cold (−40 °C) orange-yellow solution of **A** (15.0 mg, 0.0302 mmol, 1 equiv) in toluene (1.5 mL) was added to cold (−40 °C) yellow-brown solution of [U^{III}(ODtbp)$_3$] (25.2 mg, 0.0684 mmol, 1 equiv) in toluene (2.5 mL) under Ar. The resulting dark brown-red reaction mixture was left to react for 1 h under stirring at −40 °C. All volatiles were removed under vacuum and the solid was dissolved in Et_2O (0.8 mL). X-ray quality dark brown-red crystals of **2** were obtained by leaving it stand for 12 h at −40 °C, in 76.5% yield (31.2 mg).
Anal. Calcd for [{Fe(depe)$_2$}(μ-η^1:η^1-N$_2$)(U(O-2,6-^{t}Bu$_2$C$_6$H$_3$)$_3$)] $C_{62}H_{111}FeN_2O_3P_4U$: C: 55.15%; H: 8.29%; N: 2.07%. Found: C: 54.97%; H: 8.29%; N: 1.74%.

^{1}H NMR (400 MHz, d$_8$-toluene, 233 K): δ = 19.86 (s, 6H), 16.13 (s, 3H), −2.53 (s, br, 48H), −4.86 (s, 18H), −5.10 (s, 8H), −5.46 (s, 12 H), −6.37 (s, 4H), −6.63 (s, 4H), −10.93 (s, 4H), −11.9 (s, 4H). (Figure S12)
$^{31}P\{^1H\}$ NMR (162 MHz, d$_8$-toluene, 233 K): δ = −17.9 (s) (Figure S13)

IR spectrum (KBr pill): ν(N≡N) = 1820 cm^{-1} (Figure S74)

Reaction of A with [U^{III}(OTtbp)$_3$] at −40 °C in toluene. A d$_8$-toluene (0.6 mL) solution of crystalline [U(OTtbp)$_3$]$_2$(μ-η^2:η^2-N$_2$) (16.8 mg, 0.0081 mmol, 0.5 equiv) was heated to 80 °C and exposed to vacuum to remove N_2 and yield [U^{III}(OTtbp)$_3$]. Addition of orange solid **A** (8.0 mg, 0.0162 mmol, 1 equiv) to this solution under Ar at −40°C resulted in a dark brown-red solution. The $^{31}P\{^1H\}$ NMR spectrum at −40 °C of the reaction mixture (Figure S17) shows a sharp resonance at δ = 84.5 ppm (s) corresponding to **A**, and a second broad resonance at δ = −15.7 ppm, suggesting the formation of an adduct. The ^{1}H NMR spectrum of the reaction mixture at −40 °C (Figure S18) shows a pattern reminiscent of that observed for **2**, further suggesting that the formation of an adduct is occurring. The $^{31}P\{^1H\}$ NMR spectrum at 25 °C of the reaction mixture (Figure S19) shows a broad resonance at δ = 8.7 ppm, suggesting an equilibrium between the starting materials and the expected adducts, as observed for both **1-U** and **2**. However, due to the higher solubility of the complexes in toluene and hexane, nothing could be crystallized from this reaction mixture.

Synthesis of [{Fe(depe)$_2$}(μ-η^1:η^1-N$_2$)(Ce{N(SiMe$_3$)$_2$}$_3$)], 1-Ce. A yellow solution of [Ce^{III}{N(SiMe$_3$)$_2$}$_3$] (35.5 mg, 0.0571 mmol, 1 equiv) in toluene (0.9 mL) was added an orange-yellow solution of **A** (28.4 mg, 0.0572 mmol, 1 equiv) in toluene (0.6 mL) under Ar at 25 °C, resulting in a dark orange-red solution. X-ray quality orange-red crystals of **1-Ce** were obtained by leaving it stand for 12 h at −40 °C, in 83% yield (53.0 mg).
Anal. Calcd for [{Fe(depe)$_2$}(μ-η^1:η^1-N$_2$)(Ce{N(SiMe$_3$)$_2$}$_3$)] $C_{38}H_{102}FeN_5P_4Si_6Ce$: C: 40.84%; H: 9.20%; N: 6.27%. Found: C: 40.74%; H: 9.12%; N: 6.03%.

^{1}H NMR (400 MHz, d$_8$-toluene, 233 K): δ = −0.59 (s), −0.79 (s), −1.10 (s), −1.28 (s), −1.69 (br) (Figure S21).
$^{31}P\{^1H\}$ NMR (162 MHz, d$_8$-toluene, 233 K): δ = 73.8 (s) (Figure S24).

IR spectrum (KBr pill): ν(N≡N) = 1849 cm^{-1} (Figure S76).

Synthesis of [{Fe(depe)$_2$}(μ-η^1:η^1-N$_2$)(Sm{N(SiMe$_3$)$_2$}$_3$)], 1-Sm. An orange-yellow solution of **A** (33.6 mg, 0.0676 mmol, 1 equiv) in toluene (0.6 mL) was added to an off-white suspension of [Sm^{III}{N(SiMe$_3$)$_2$}$_3$] (42.7 mg, 0.0676 mmol, 1 equiv) in toluene (0.5 mL) under Ar at 25 °C, resulting in a red-orange solution. X-ray quality yellow-orange crystals of **1-Sm** were obtained by leaving it stand for 2 days at −40 °C, in 77% yield (59.0 mg).
Anal. Calcd for [{Fe(depe)$_2$}(μ-η^1:η^1-N$_2$)(Sm{N(SiMe$_3$)$_2$}$_3$)] $C_{38}H_{102}FeN_5P_4Si_6Sm$: C: 40.47%; H: 9.12%; N: 6.21%. Found: C: 40.79%; H: 9.28%; N: 6.04%.

^{1}H NMR (400 MHz, d$_8$-toluene, 233 K): δ = 8.83 (br), 1.24, 1.03 (s), 0.89 (s), 0.63 (s), −0.45 (s) (Figure S27).
$^{31}P\{^1H\}$ NMR (162 MHz, d$_8$-toluene, 233 K): δ = 81.3 (s) (Figure S29).

IR spectrum (KBr pill): ν(N≡N) = 1842 cm^{-1} (Figure S77).

Synthesis of [{Fe(depe)$_2$}(μ-η^1:η^1-N$_2$)(Dy{N(SiMe$_3$)$_2$}$_3$)], 1-Dy. An orange-yellow solution of **A** (38.0 mg, 0.0766 mmol, 1 equiv) in toluene (1.5 mL) was added to a colorless solution of [Dy^{III}{N(SiMe$_3$)$_2$}$_3$] (49.3 mg, 0.0766 mmol, 1 equiv) in toluene (1.5 mL) under Ar at 25 °C, resulting in a red-orange solution. X-ray quality yellow-orange crystals of **1-Dy** were obtained by leaving it stand for 24 h at −40 °C, in 87% yield (76.0 mg).
Anal. Calcd for [{Fe(depe)$_2$}(μ-η^1:η^1-N$_2$)(Dy{N(SiMe$_3$)$_2$}$_3$)] $C_{38}H_{102}FeN_5P_4Si_6Dy$: C: 40.04%; H: 9.02%; N: 6.14%. Found: C: 39.81%; H: 8.86%; N: 5.73%.

^{1}H NMR (400 MHz, d$_8$-toluene, 298 K): 0.53 (s) ppm, −99.90 (br) (Figure S31).
^{31}P{^{1}H} NMR (162 MHz, d$_8$-toluene, 298 K): δ = 82.1 (s) (Figure S33).

IR spectrum (KBr pill): ν(N≡N) = 1839 cm^{-1} (Figure S79).

Synthesis of [{Fe(depe)$_2$}(μ-η^1:η^1-N$_2$)(Tm{N(SiMe$_3$)$_2$}$_3$)], 1-Tm. An orange-yellow solution of **A** (32.8 mg, 0.07 mmol, 1 equiv) in toluene (0.6 mL) was added to an off-white suspension of [Tm^{III}{N(SiMe$_3$)$_2$}$_3$] (42.9 mg, 0.07 mmol, 1 equiv) in toluene (0.6 mL) under Ar at 25 °C, resulting in a red-orange solution. The reaction mixture was swirled/agitated a few times to ensure proper mixing of reactants. The volatiles were then removed under vacuum and the reaction mixture was redissolved in hexane (5 mL) and placed at −40 °C for 1 day. A red crystalline solid formed that was collected and washed with cold (−40 °C) hexane (3 x 0.2 mL). The red solid obtained was dried under vacuum to yield **1-Tm** in 42 % yield (32.1 mg). X-ray quality red-orange crystals suitable for diffraction of **1-Tm** were obtained by leaving the reaction mixture in hexane stand for 1 day at −40 °C.
Anal. Calcd for [{Fe(depe)$_2$}(μ-η^1:η^1-N$_2$)(Tm{N(SiMe$_3$)$_2$}$_3$)] $C_{38}H_{102}FeN_5P_4Si_6Tm$: C: 39.81%; H: 8.97%; N: 6.11%. Found: C: 39.83%; H: 9.00%; N: 5.51%.

^{1}H NMR (400 MHz, d$_8$-toluene, 298 K): δ = 73.82 (br), 1.85, 1.63, 1.18, 0.85 (Figure S35).
^{31}P{^{1}H} NMR (162 MHz, d$_8$-toluene, 298 K): δ = 84.2 (br) (Figure S36).

IR spectrum (KBr pill): ν(N≡N) = 1837 cm^{-1} (Figure S80).

Synthesis of [{Fe(depe)$_2$}(μ-η^1:η^1-N$_2$)(Yb{N(SiMe$_3$)$_2$}$_2$(OEt$_2$)$_{0.5}$(THF)$_{0.5}$)], 1*-Yb. A cold (−40 °C) orange-yellow solution of **A** (33.5 mg, 0.0674 mmol, 1 equiv) in toluene (1.0 mL) was added to an orange solution of [Sm^{II}{N(SiMe$_3$)$_2$}$_2$(thf)$_2$] (43.1 mg, 0.0676 mmol, 1 equiv) in toluene (2.5 mL) under Ar at 25 °C, resulting in a red-orange solution. Volatiles were removed under vacuum and the resultant red-orange solid was dissolved in Et$_2$O (0.4 mL). X-ray quality red-orange crystals of **1*-Yb** were obtained by leaving it stand for 12 h at −40 °C, in 73% yield (52.5 mg).
Anal. Calcd for [{Fe(depe)$_2$}(μ-η^1:η^1-N$_2$)(Yb{N(SiMe$_3$)$_2$}$_2$(OEt$_2$)$_{0.5}$(THF)$_{0.5}$)] $C_{36}H_{91}FeN_4P_4Si_4Yb$: C: 40.67%; H: 8.82%; N: 5.27%. Found: C: 40.54; H: 8.55%; N: 5.29%.

^{1}H NMR (400 MHz, d$_8$-toluene, 233 K): δ = 1.35 (br), 1.17 (br), 1.02 (br), 0.80 (br), 0.52 (s) (Figure S37).
^{31}P{^{1}H} NMR (162 MHz, d$_8$-toluene, 233 K): δ = 81.2 (s) (Figure S39).

IR spectrum (KBr pill): ν(N≡N) = 1874 cm^{-1} (Figure S78).

Synthesis of [{Fe(depe)$_2$}(μ-η^1:η^1-N$_2$)(Sm{N(SiMe$_3$)$_2$}$_2$)], 1*-Sm. An orange-yellow solution of **A** (90.1 mg, 0.182 mmol, 2 equiv) in hexane (3.0 mL) was added to a cold (−40 °C) dark violet solution of [Sm^{II}{N(SiMe$_3$)$_2$}$_2$]$_2$ (85.5 mg, 0.0907 mmol, 1 equiv) in hexane (3.0 mL) under Ar at −40 °C, resulting in a dark brown-yellow solution. X-ray quality dark brown-green crystals of **1*-Sm** were obtained by slow evaporation of a saturated hexane solution over the course of 12 hs at −40 °C, in 83.6% yield (146.8 mg).
Anal. Calcd for [{Fe(depe)$_2$}(μ-η^1:η^1-N$_2$)(Sm{N(SiMe$_3$)$_2$}$_2$)] $C_{32}H_{84}FeN_4P_4Si_4Sm$: C: 39.73%; H: 8.75%; N: 5.79%. Found: C: 39.54; H: 8.66%; N: 5.52%.

^{1}H NMR (400 MHz, C_6D_{12}, 298 K): δ = 2.57 (s), 1.07 (br), 0.61 (br), 0.16 (br) (Figure S42).
^{31}P{^{1}H} NMR (162 MHz, C_6D_{12}, 298 K): δ = 110.9 (br) (Figure S43)

Multinuclear NMR spectroscopy (Figures S44 and S45) shows the slow decomposition of **1*-Sm** in cyclohexane solution at 25 °C over the course of 24 h. The formation of **1-Sm** over the course of 1 week was confirmed by measurement of single crystals.

^{1}H NMR (400 MHz, d_8-toluene, 233 K): δ = 7.28 (br), 1.22 (s), 0.53 (s), −0.11 (s), −1.44 (s), −2.22 (br), −4.06 (br) (Figure S46).
^{31}P{^{1}H} NMR (162 MHz, d_8-toluene, 233 K): δ = 113.3 (s) (Figure 48)

Multinuclear NMR spectroscopy (Figures S50 and S51) shows the slow disappearance of **1*-Sm** in toluene solution at −40 °C over the course of several days, with concomitant formation of **3** (Scheme S1), probably due to disproportionation of "[Sm^{II}{N($SiMe_3$)$_2$}$_2$]" into Sm(III) and Sm(0).

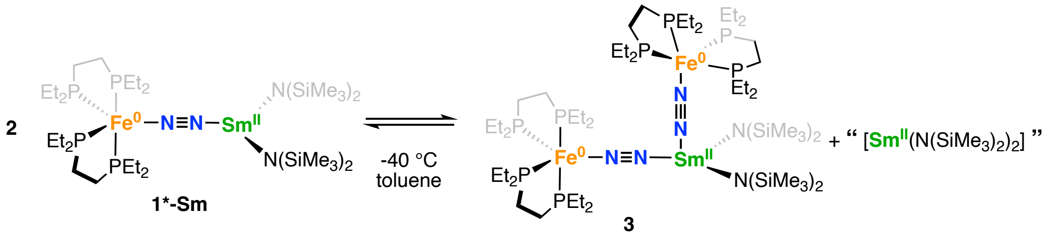

Scheme S1: Equilibrium observed upon dissolution of **1*-Sm** in toluene at −40 °C

IR spectrum (KBr pill): ν(N≡N) = 1888 cm^{-1} (Figure S81)

Reaction of A with [Sm^{II}{N($SiMe_3$)$_2$}$_2$]$_2$ at −40 °C in toluene. A cold (−40 °C) dark violet solution of [Sm^{II}{N($SiMe_3$)$_2$}$_2$]$_2$ (9.4 mg, 0.010 mmol, 1 equiv) in d_8-toluene (0.4 mL) was added to orange solid **A** (9.9 mg, 0.02 mmol, 2 equiv) under Ar at −40°C, resulting in a dark brown-green solution. The ^{31}P{^{1}H} NMR spectrum at −40 °C of the reaction mixture (Figure S53) shows a sharp resonance at δ = 125.0 ppm (s), that suggest the formation of a new species, different to **1-Sm** and **A** together with two broad resonances at δ = 112 ppm (s) and some **1-Sm**. The ^{1}H NMR spectrum also suggest the formation of a new species (Figure S52) with 3 broad resonances at δ = 6.37 ppm (br), 2.55 ppm (br) and 1.15 ppm (br), together with two sharp resonances at δ = 0.85 ppm (s) and −0.87 ppm. Isolation attempts resulted only in the crystallization of the Sm(III) species, **1-Sm** and [Sm^{III}{N($SiMe_3$)$_2$}$_3$], suggesting the ligand scrambling and oxidation upon decomposition of the species observed in the multinuclear NMR spectra.

Synthesis of [({Fe(depe)$_2$}(μ-η^1:η^1-N_2))$_2$(Sm{N($SiMe_3$)$_2$}$_2$)], 3. An orange-yellow solution of **A** (134.6 mg, 0.271 mmol, 4 equiv) in hexane (6.0 mL) was added to a cold (−40 °C) dark violet solution of [Sm^{II}{N($SiMe_3$)$_2$}$_2$]$_2$ (63.8 mg, 0.0677 mmol, 1 equiv) in hexane (3.9 mL) under Ar at −40 °C, resulting in a dark brown-yellow solution. Dark green solid precipitated out of the reaction mixture over the course of 2 h. The solid was recovered by filtration and washed with minimal cold (−40 °C) hexane, giving **3** in 70% yield (139 mg). X-ray quality dark green crystals of **3** were obtained obtained by slow evaporation of a saturated hexane solution over the course of 12 h at −40 °C
Anal. Calcd for [({Fe(depe)$_2$}(μ-η^1:η^1-N_2))$_2$(Sm{N($SiMe_3$)$_2$}$_2$)] $C_{55}H_{139}Fe_2N_6P_8Si_4Sm$: C: 43.53%; H: 9.13%; N: 5.21%. Found: C: 43.84; H: 9.30%; N: 5.58%.

^{1}H NMR (400 MHz, C_6D_{12}, 298 K): δ = 2.16 (br), 1.65 (br), 1.33(br), 1.19 (s) (Figure S54).
^{31}P{^{1}H} NMR (162 MHz, C_6D_{12}, 298 K): δ = 103.56 (br) (Figure S55)
^{1}H NMR (400 MHz, d_8-toluene, 233 K): δ = 6.34 (br), 2.58 (br), 1.35 (br), 1.15 (s) (Figure S58).
^{31}P{^{1}H} NMR (162 MHz, d_8-toluene, 233 K): δ = 125.25 (s) (Figure S60)

Multinuclear NMR spectroscopy shows the slow decomposition of **3** in cyclohexane solution at 25 °C over the course of 24 h (Figure S56 and S57). The formation of **1-Sm** over the course of 1 week was confirmed by XRD measurement of single crystals.

IR spectrum (KBr pill): ν(N≡N) = 1888 cm^{-1} and 1896 cm^{-1} (Figure S82)

NMR Spectra

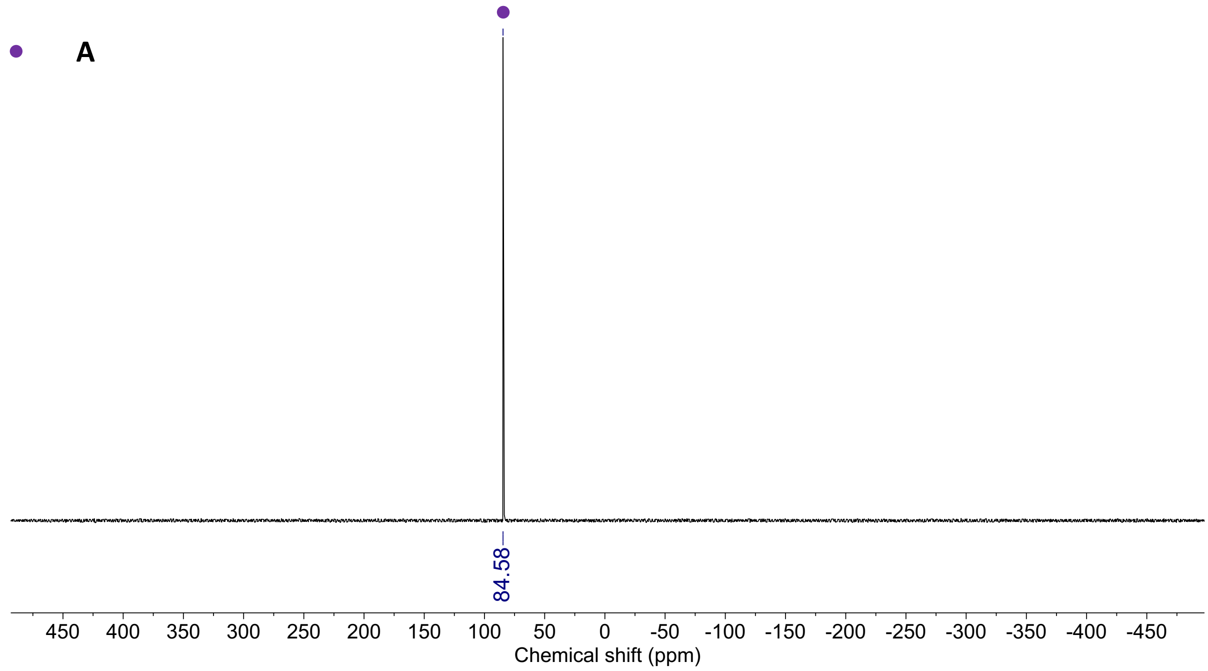

Figure S1: $^{31}P\{^1H\}$ NMR spectrum (162 MHz, d_8-toluene, 233 K) of the reaction mixture obtained after addition of **A** to $[U^{III}(C_5Me_4H)_3]$ in toluene at −40 °C under Ar

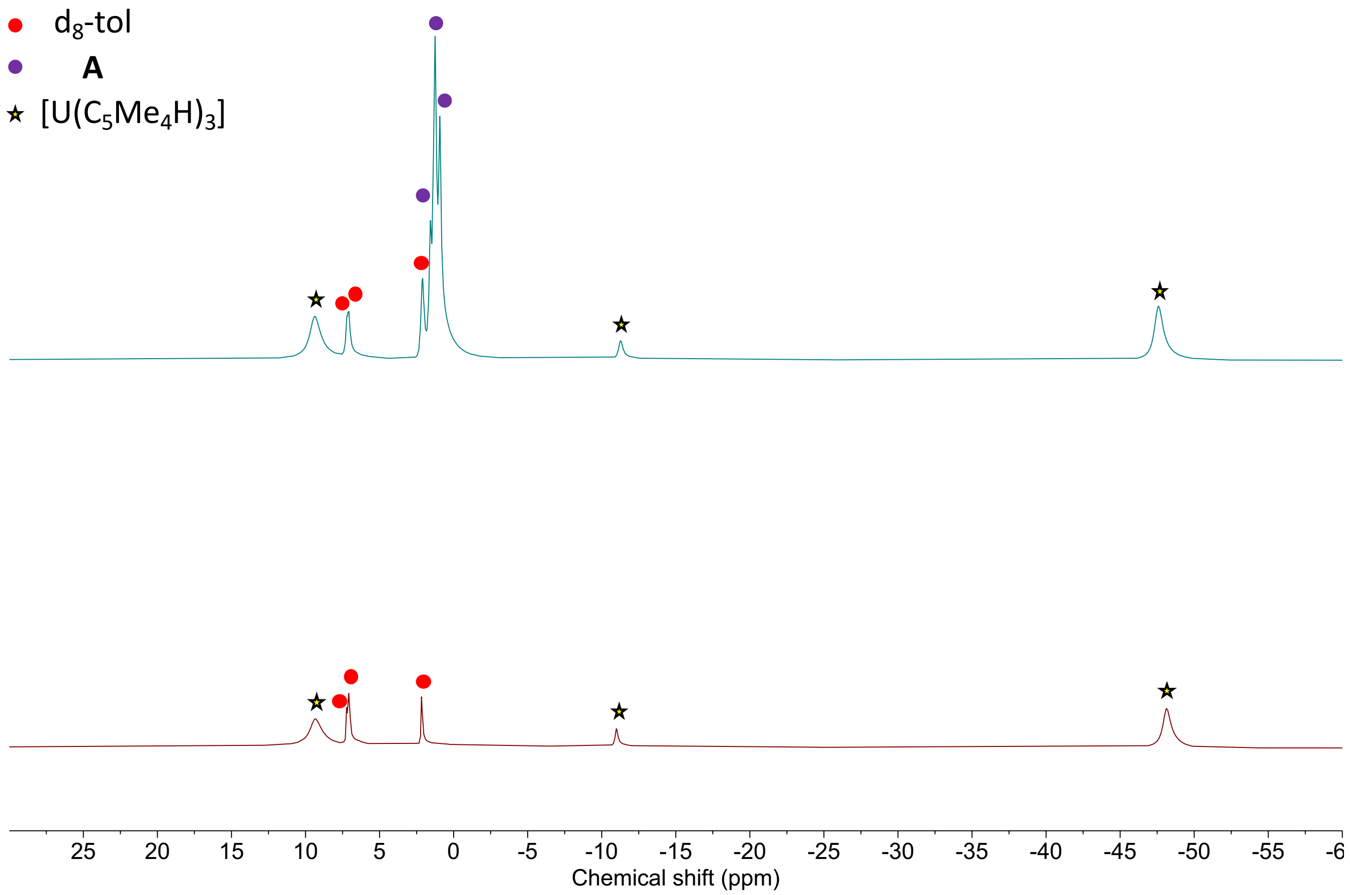

Figure S2: ^{1}H NMR spectra (400 MHz, d_8-toluene, 233 K) comparison of the reaction mixture obtained before (bottom) and after (top) addition of **A** to [$U^{III}(C_5Me_4H\}_3$] in toluene at −40 °C under Ar

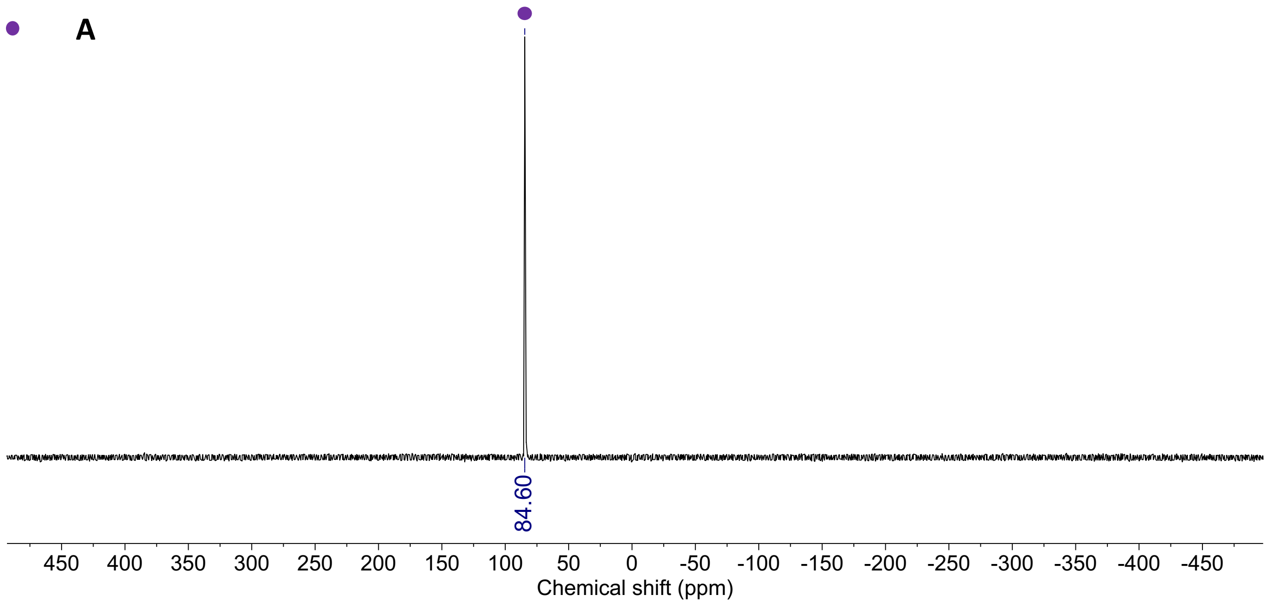

Figure S3: $^{31}P\{^{1}H\}$ NMR spectrum (162 MHz, d_8-toluene, 298 K) of the reaction mixture obtained after addition of **A** to [$U^{III}(C_5Me_4H\}_3$] in toluene at −40 °C under Ar

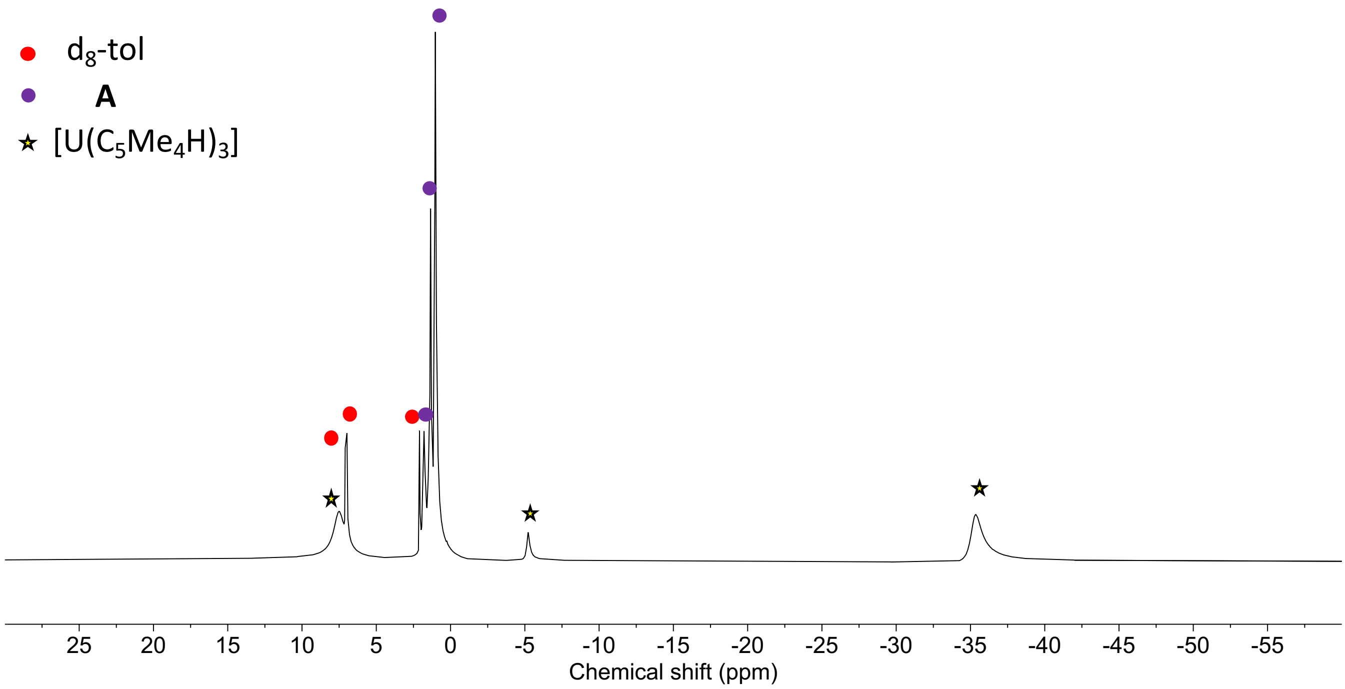

Figure S4: ^{1}H NMR spectrum (400 MHz, d_8-toluene, 298 K) of the reaction mixture obtained after addition of **A** to [$U^{III}(C_5Me_4H\}_3$] in toluene at −40 °C under Ar

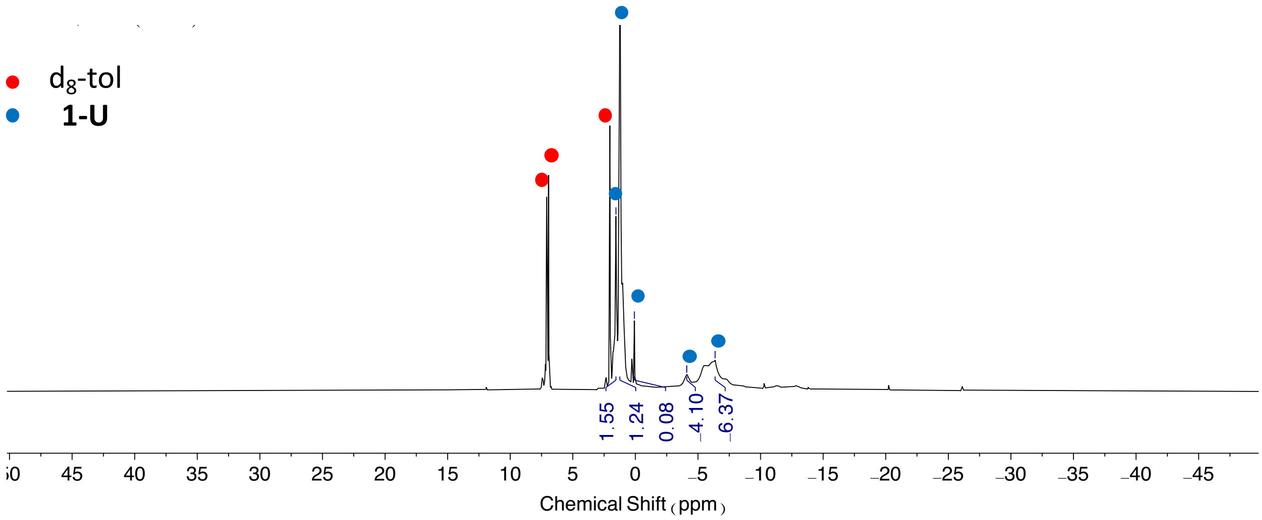

Figure S5: ^{1}H NMR spectrum (400 MHz, d_8-toluene, 233 K) of crystals of **1-U**.

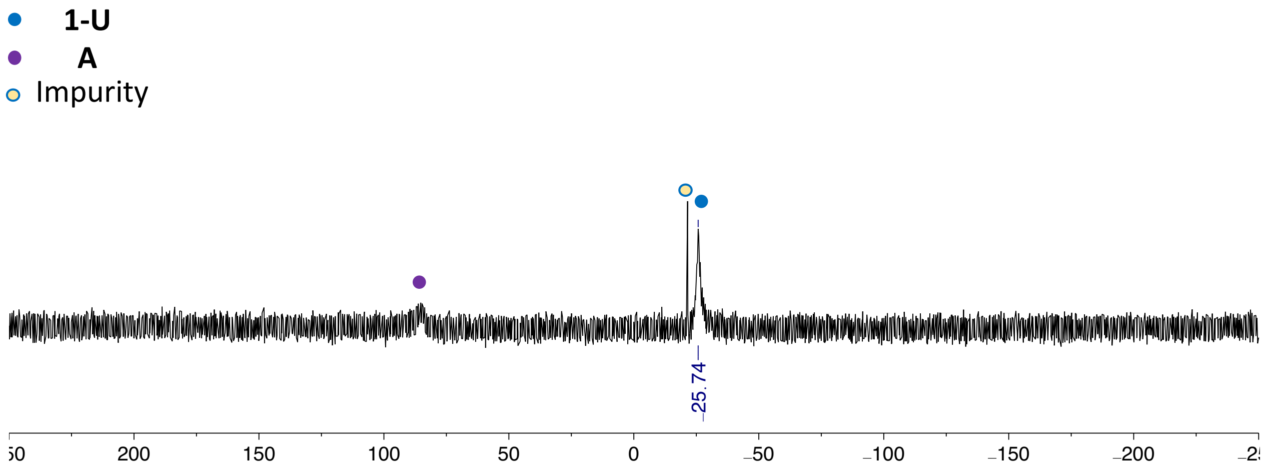

Figure S6: $^{31}P\{^1H\}$ NMR spectrum (162 MHz, d_8-toluene, 233 K) of crystals of **1-U**.

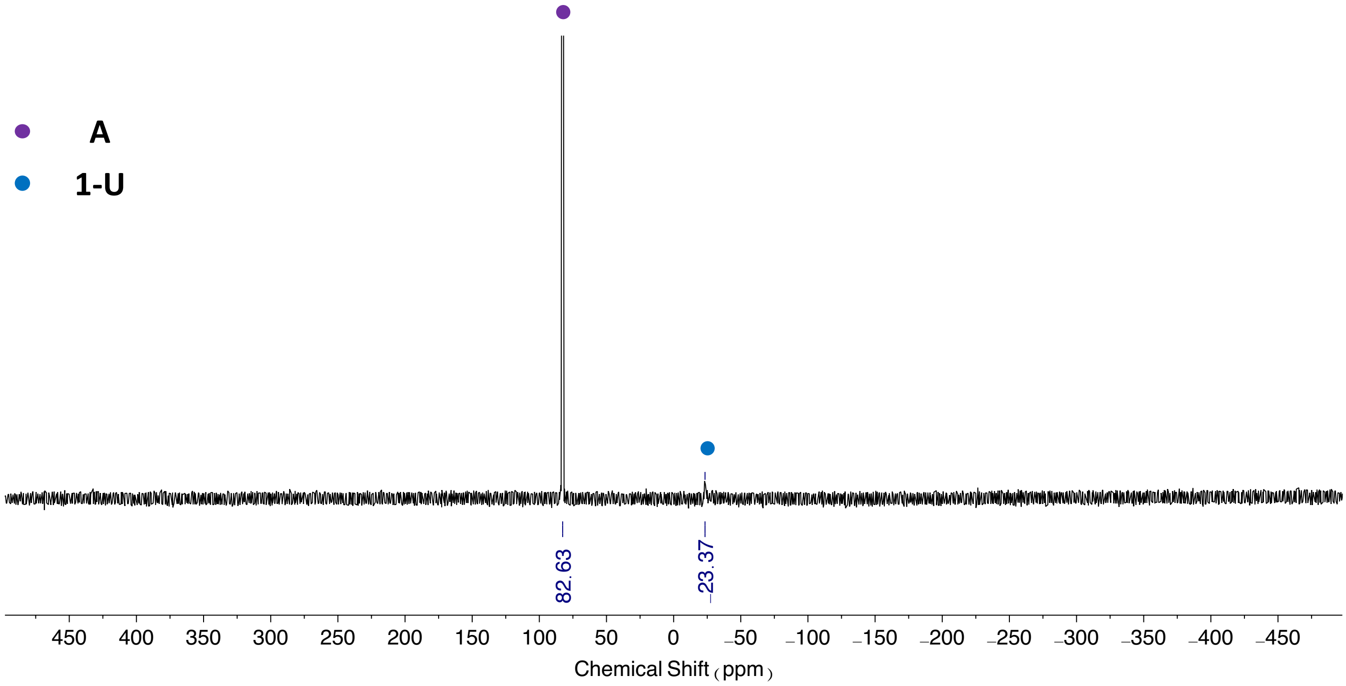

Figure S7: $^{31}P\{^1H\}$ NMR spectrum (162 MHz, d_8-THF, 233 K) of crystals of **1-U**.

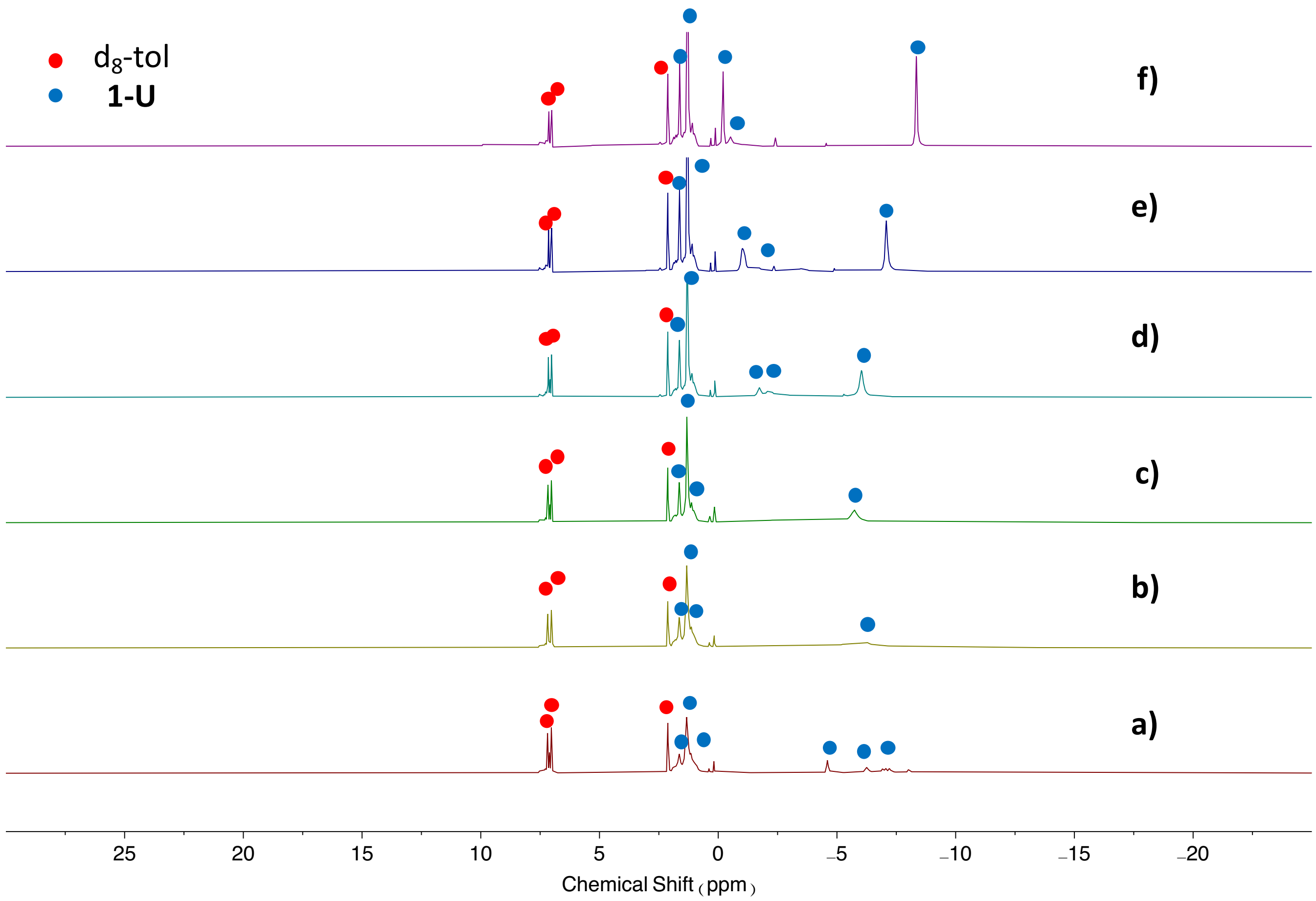

Figure S8: Variable temperature ^{1}H NMR spectra (400 MHz, d_8-toluene) of crystals of **1-U**. a) 218 K, b) 233 K, c) 248 K, d) 263 K, e) 278 K, and f) 293 K.

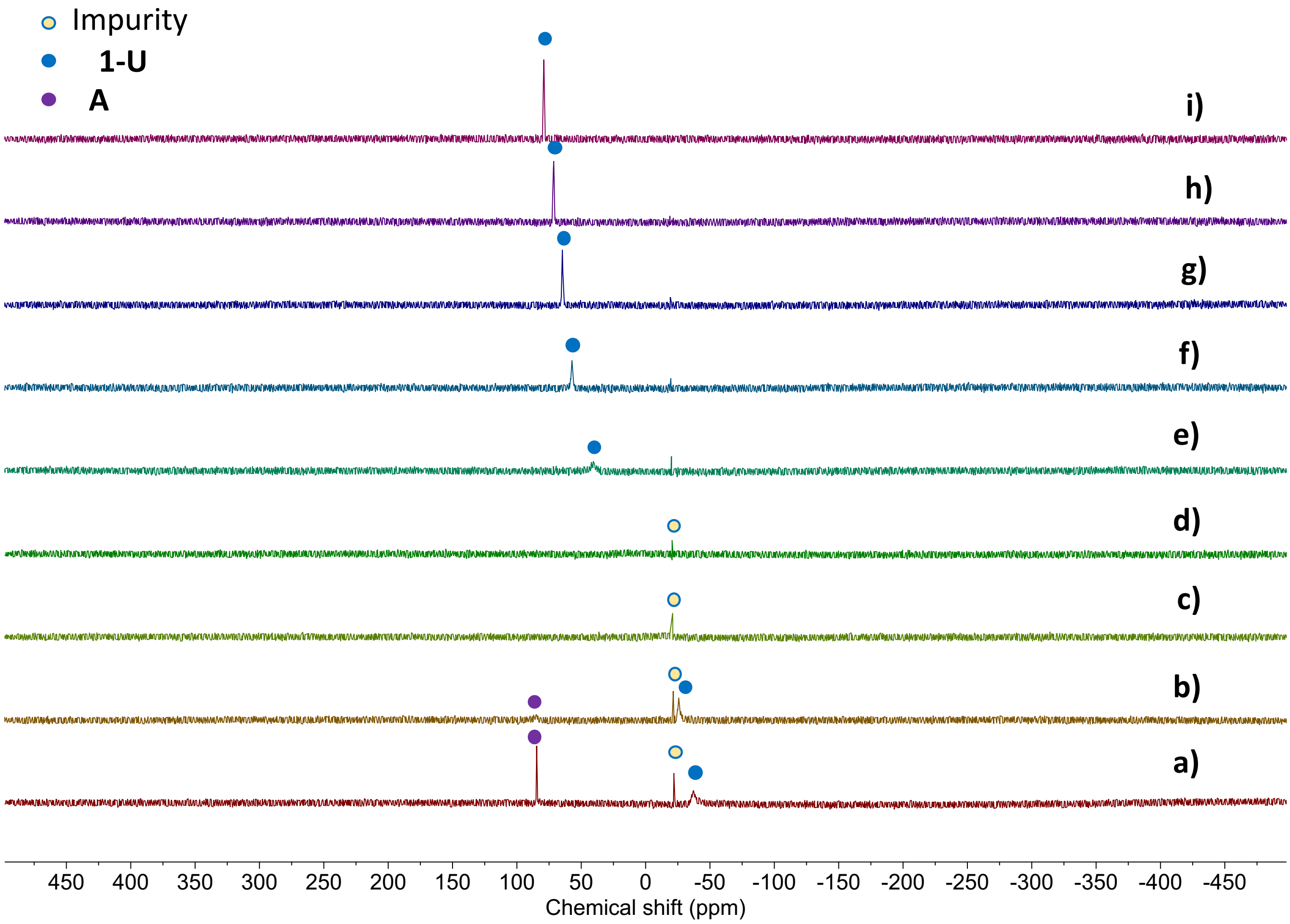

Figure S9: Variable temperature $^{31}P\{^{1}H\}$ NMR spectra (162 MHz, d_8-toluene) of crystals of **1-U**. a) 218 K, b) 233 K, c) 248 K, d) 263 K, e) 278 K, and f) 293 K, g) 308 K, h) 323 K, and i) 338 K.

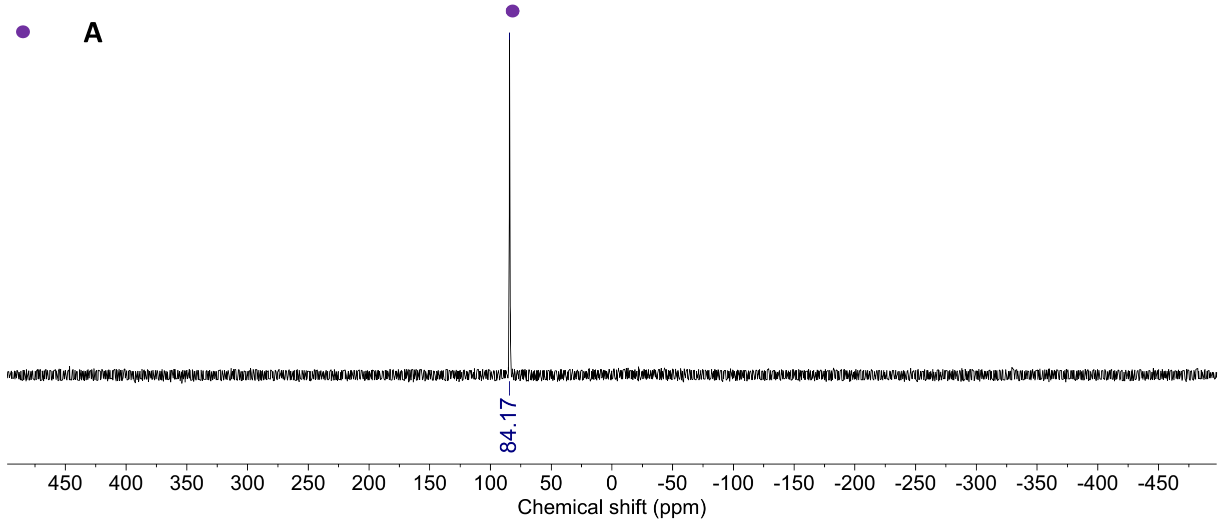

Figure S10: $^{31}P\{^{1}H\}$ NMR spectrum (162 MHz, d_8-toluene, 233 K) of the reaction mixture obtained after addition of **A** to $[U^{III}\{N(SiMe_2Ph)_2\}_3]$ in toluene at −40 °C under Ar

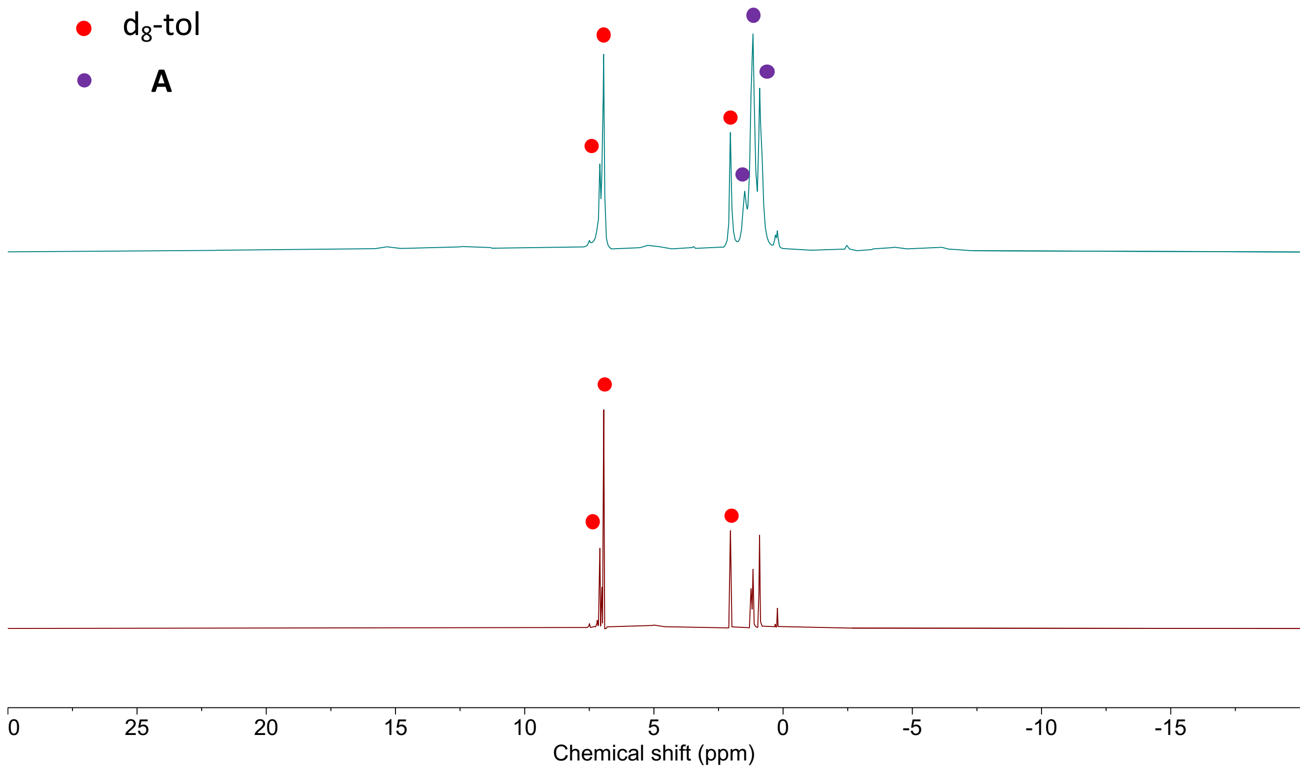

Figure S11: ^{1}H NMR spectra (400 MHz, d_8-toluene, 233 K) comparison of the reaction mixture obtained before (bottom) and after (top) addition of **A** to [$U^{III}\{N(SiMe_2Ph)_2\}_3$] in toluene at −40 °C under Ar

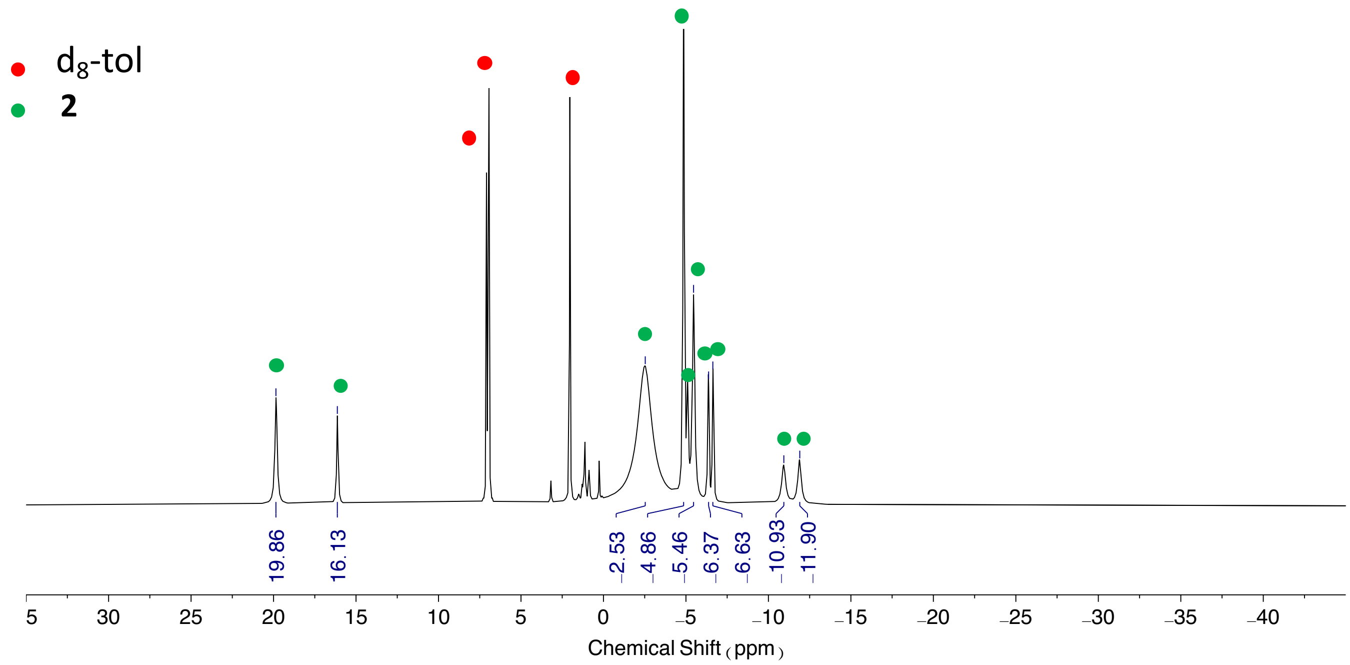

Figure S12: ^{1}H NMR spectrum (400 MHz, d_8-toluene, 233 K) of crystals of **2**.

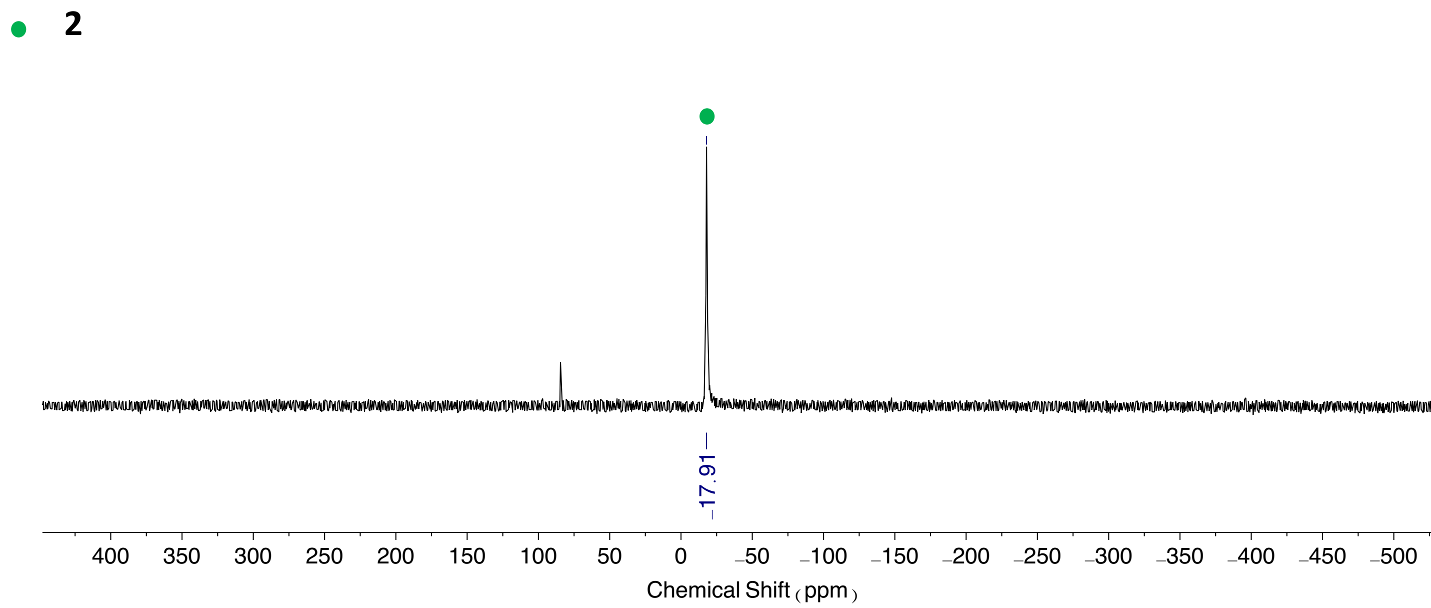

Figure S13: $^{31}P\{^{1}H\}$ NMR spectrum (162 MHz, d_8-toluene, 233 K) of crystals of **2**.

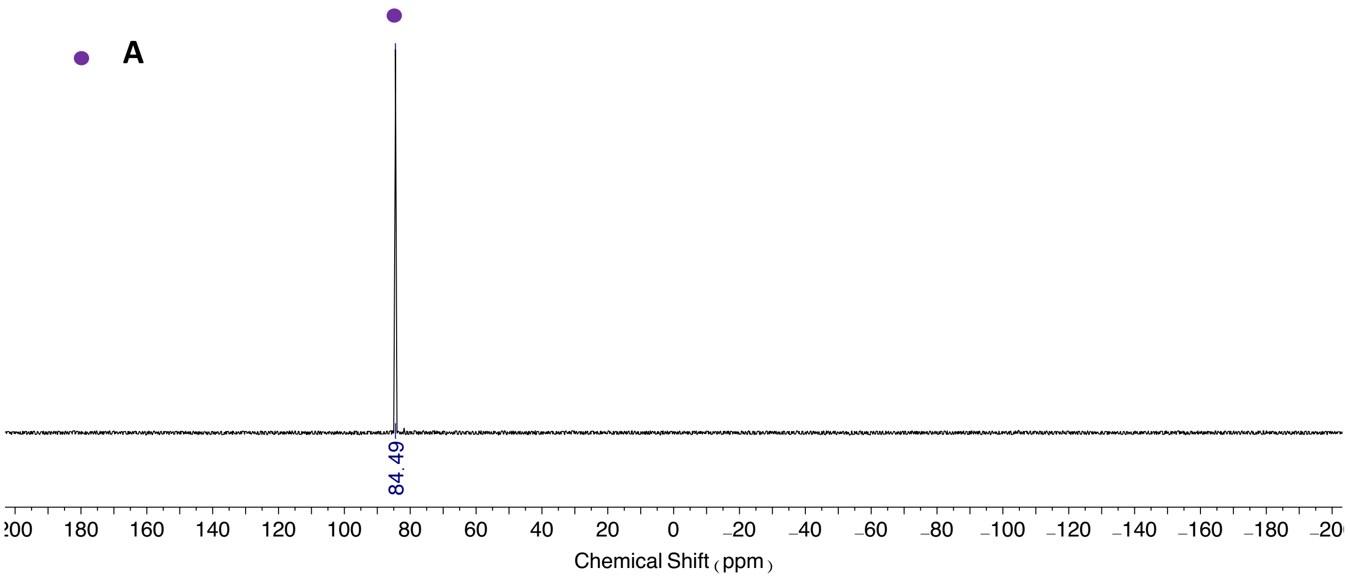

Figure S14: $^{31}P\{^{1}H\}$ NMR spectrum (162 MHz, d_8-THF, 233 K) of crystals of **2**.

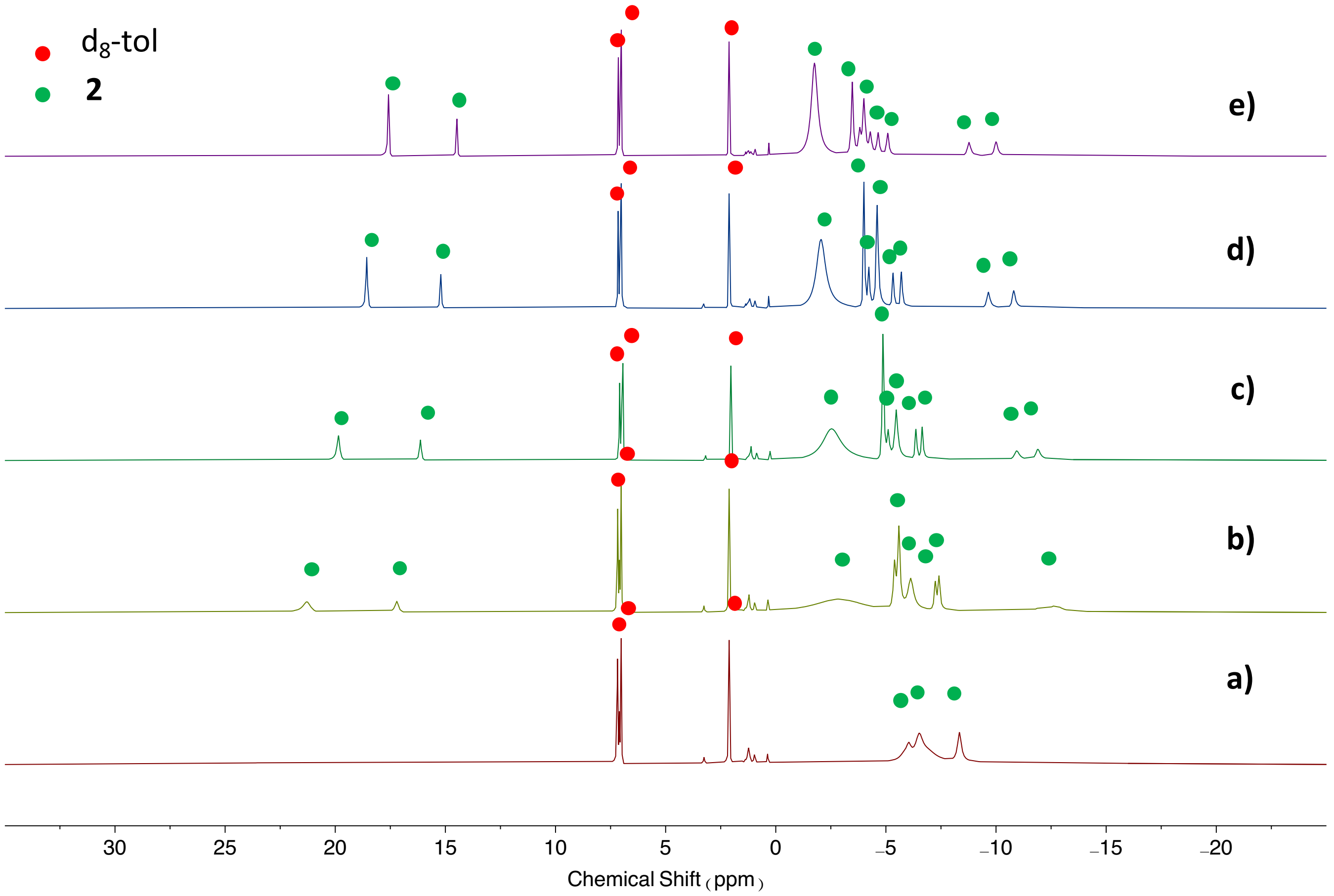

Figure S15: Variable temperature ^{1}H NMR spectra (162 MHz, d_8-toluene) of crystals of **2.** A) 203 K, b) 218 K, c) 233 K, d) 248 K, and e) 263 K.

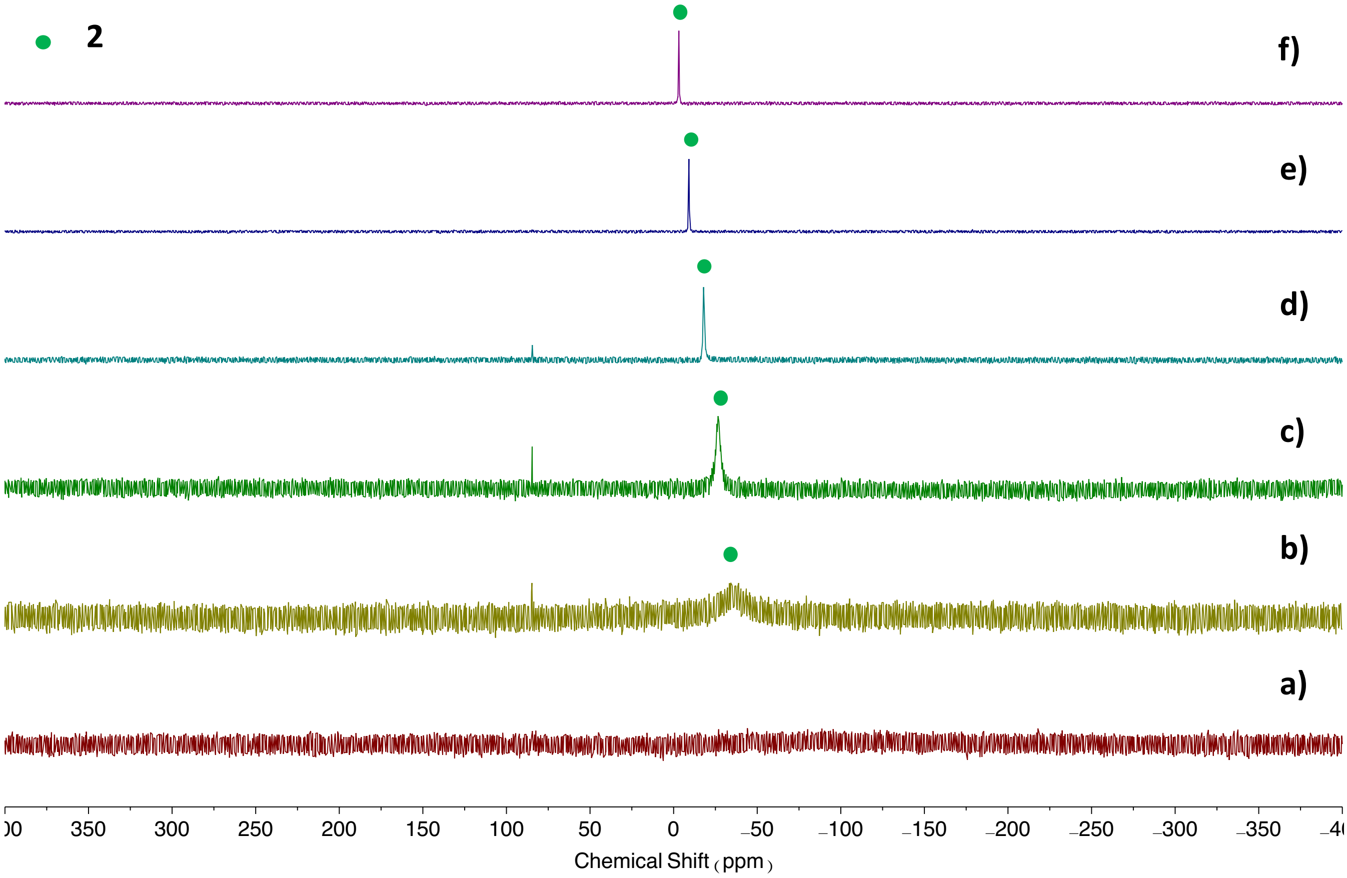

Figure S16: Variable temperature $^{31}P\{^1H\}$ NMR spectra (162 MHz, d_8-toluene) of crystals of **2.** A) 193 K, b) 203 K, c) 218 K, d) 233 K, e) 248 K, and f) 263 K.

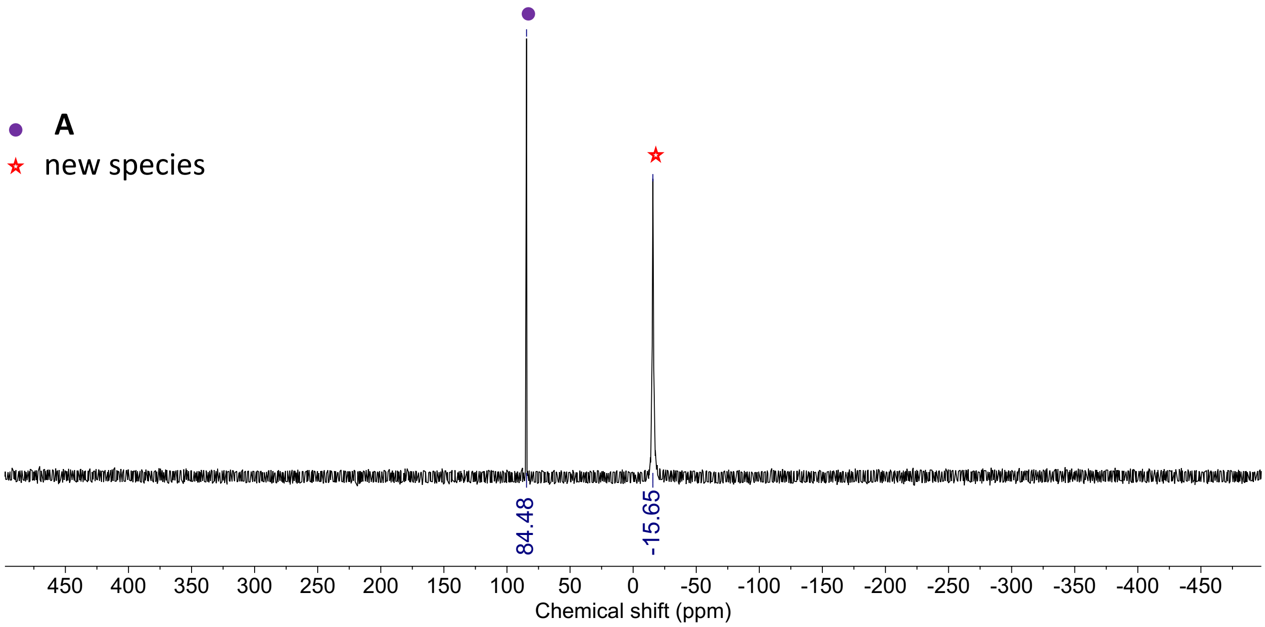

Figure S17: $^{31}P\{^1H\}$ NMR spectrum (162 MHz, d_8-toluene, 233 K) of the reaction mixture obtained after addition of **A** to [U(OTtbp)$_3$], OTtbp = O-2,4,6-$^tBu_3C_6H_2$in toluene at −40 °C under Ar

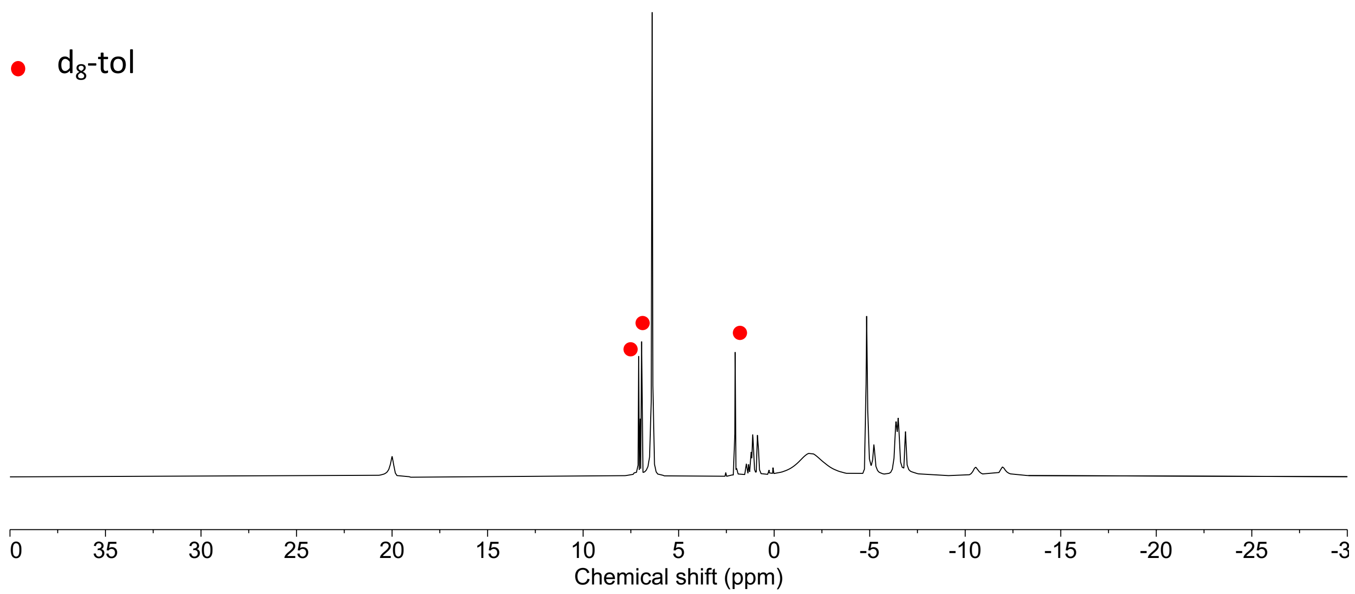

Figure S18: ^{1}H NMR spectrum (400 MHz, d_8-toluene, 233 K) of the reaction mixture obtained after addition of **A** to $[U(OTtbp)_3]$, OTtbp = O-2,4,6-$^{t}Bu_3C_6H_2$ in toluene at −40 °C under Ar

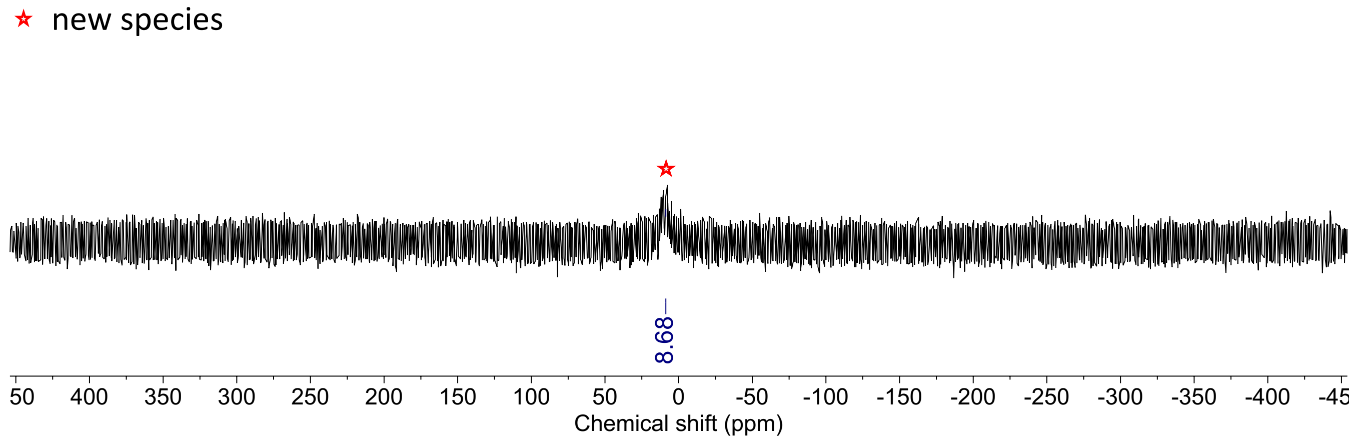

Figure S19: $^{31}P\{^{1}H\}$ NMR spectrum (162 MHz, d_8-toluene, 298 K) of the reaction mixture obtained after addition of **A** to $[U(OTtbp)_3]$, OTtbp = O-2,4,6-$^{t}Bu_3C_6H_2$ in toluene at −40 °C under Ar

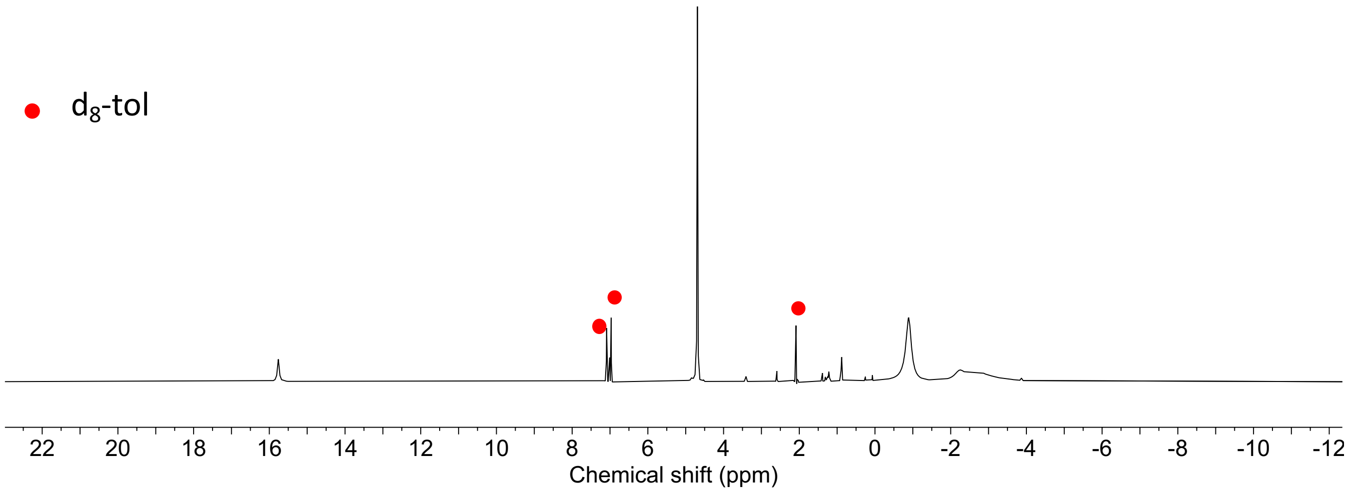

Figure S20: ^{1}H NMR spectrum (400 MHz, d_8-toluene, 298 K) of the reaction mixture obtained after addition of **A** to $[U(OTtbp)_3]$, OTtbp = O-2,4,6-$^{t}Bu_3C_6H_2$ in toluene at −40 °C under Ar

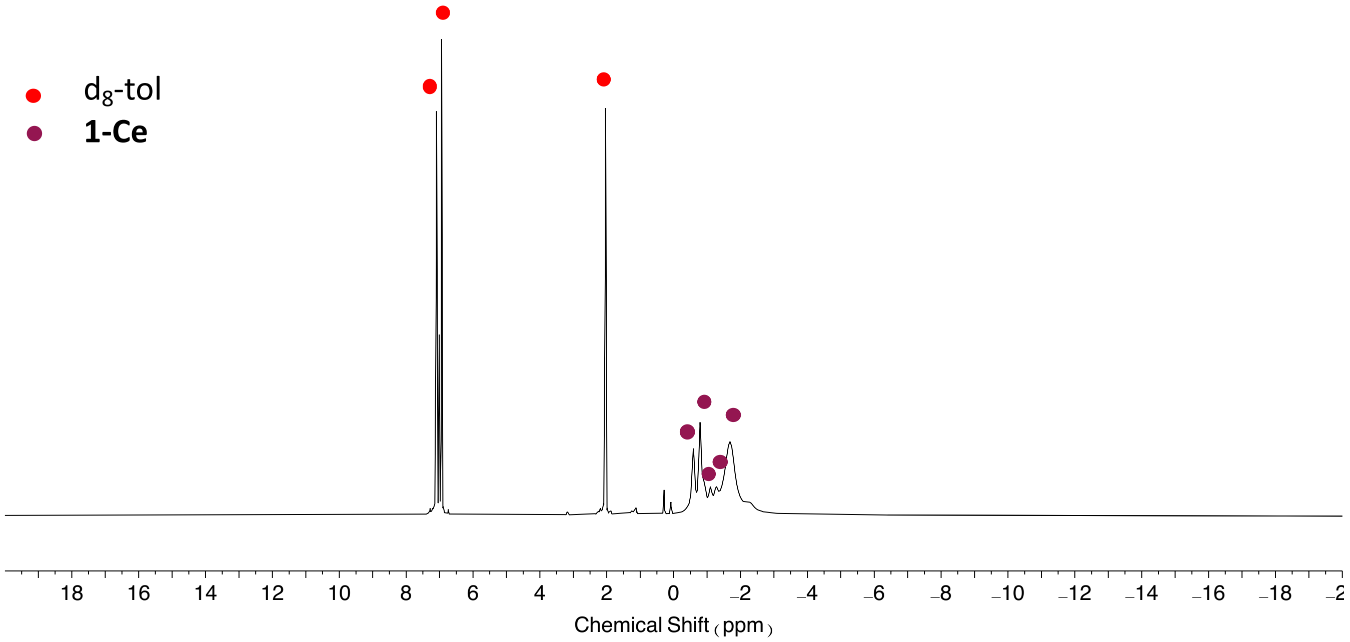

Figure S21: ^{1}H NMR spectrum (400 MHz, d_8-toluene, 233 K) of crystals of **1-Ce**.

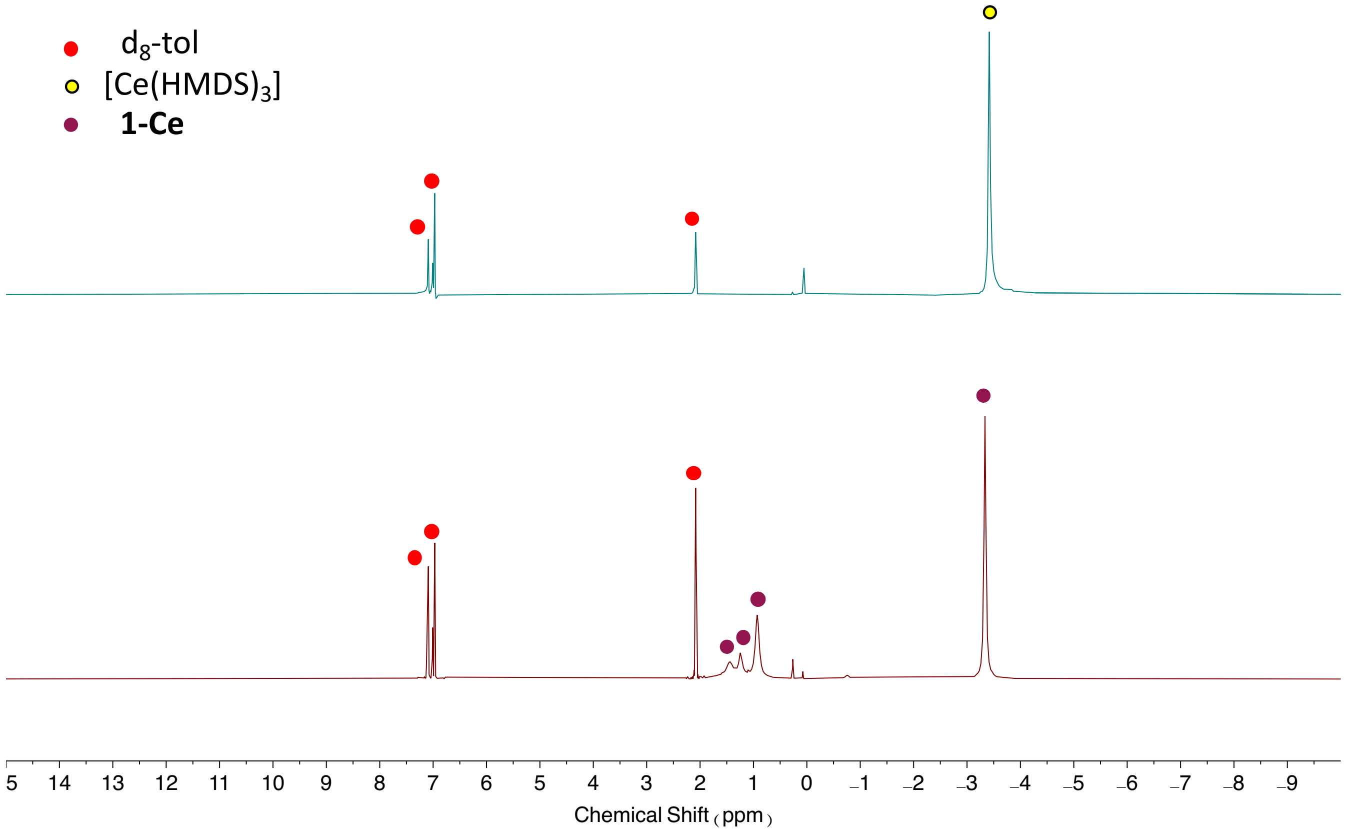

Figure S22: ^{1}H NMR spectra comparison (400 MHz, d_8-toluene, 298 K) of crystals of **1-Ce** (bottom) and [Ce{N(SiMe$_3$)$_2$}$_3$] (top).

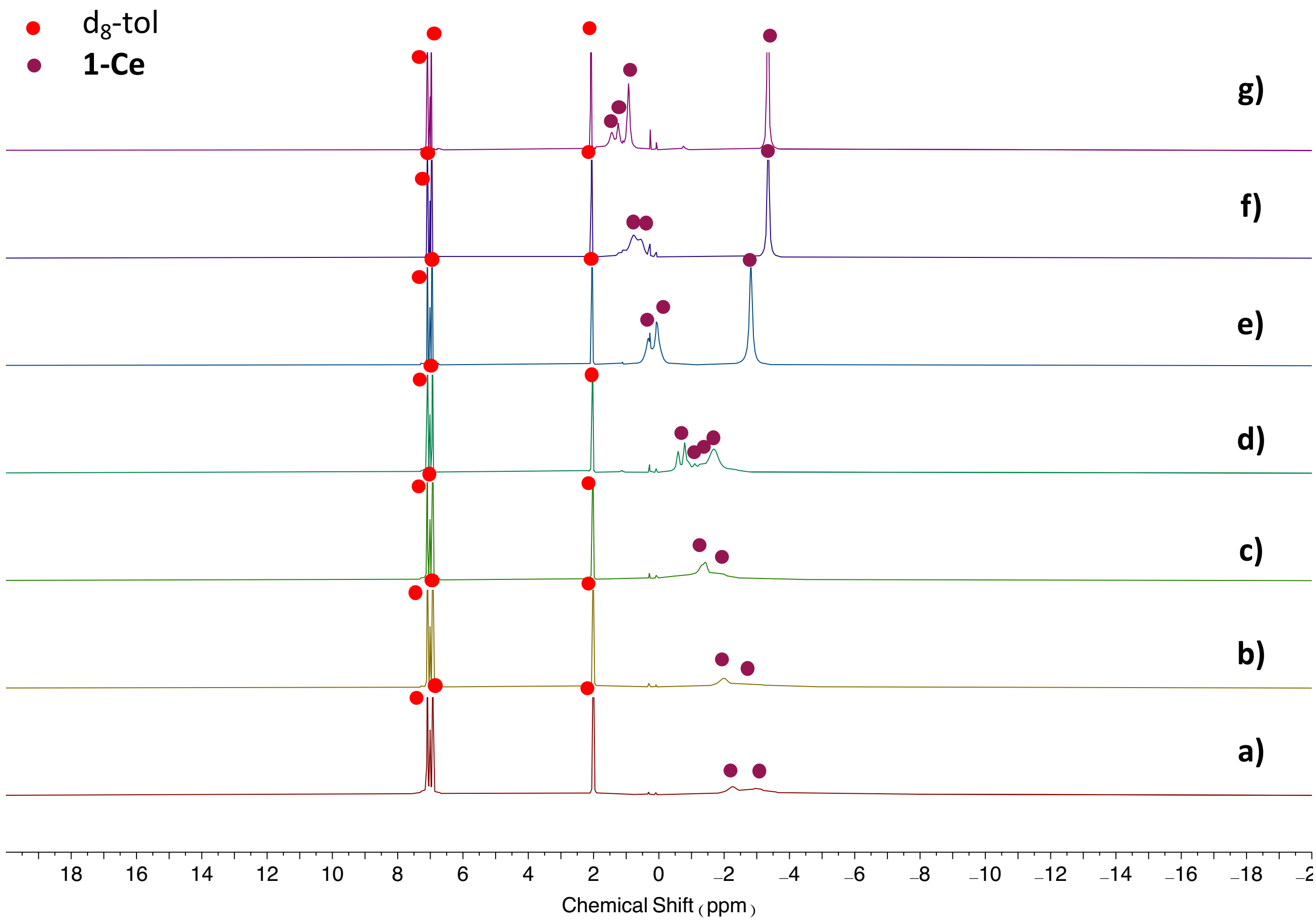

Figure S23: Variable temperature ^{1}H NMR spectra (400 MHz, d_8-toluene) of crystals of **1-Ce**. A) 193 K, b) 203 K, c) 218 K, d) 233 K, e) 248 K, f) 263 K, and g) 298 K.

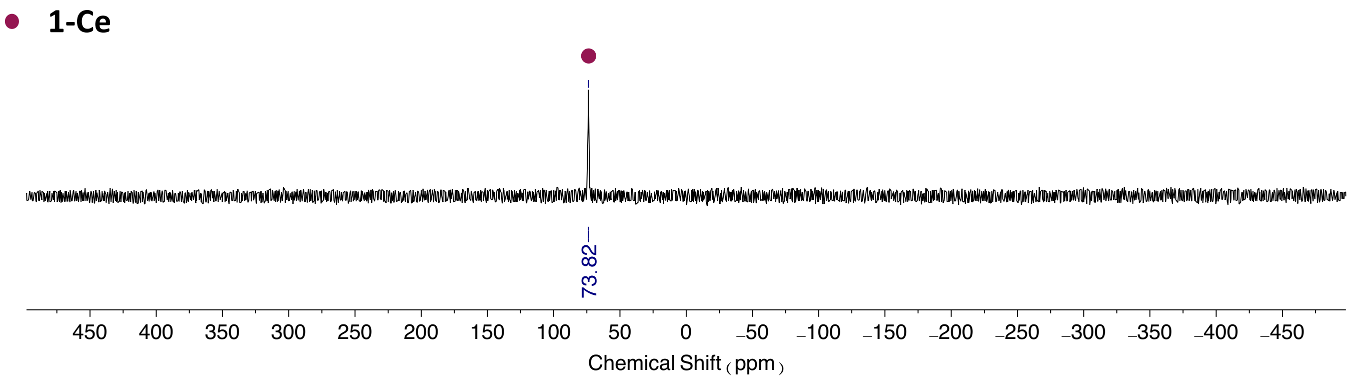

Figure S24: $^{31}P\{^{1}H\}$ NMR spectrum (162 MHz, d_8-toluene, 233 K) of crystals of **1-Ce**.

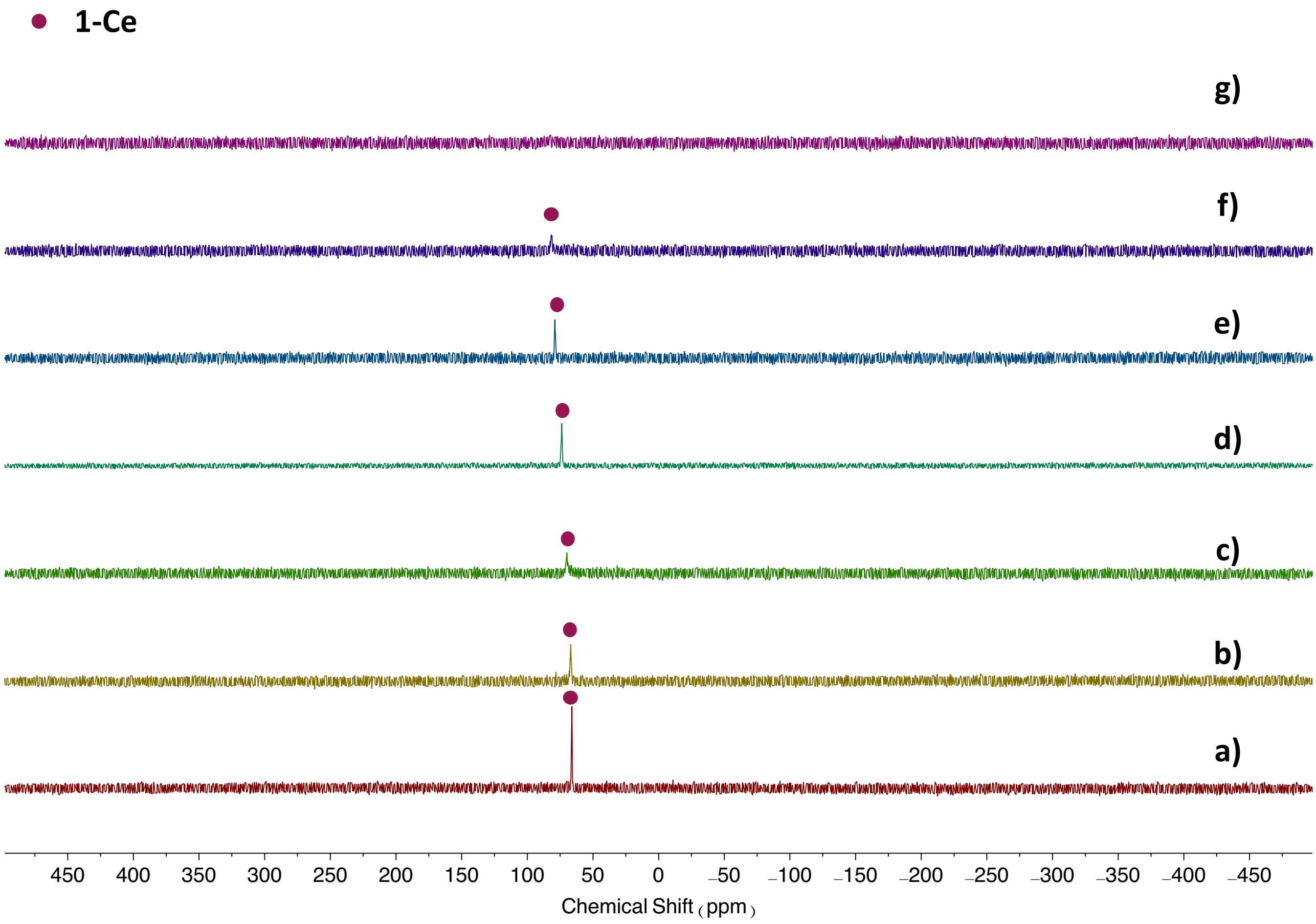

Figure S25: Variable temperature $^{31}P\{^{1}H\}$ NMR spectra (162 MHz, d_8-toluene) of crystals of **1-Ce.** A) 193 K, b) 203 K, c) 218 K, d) 233 K, e) 248 K, f) 263 K, and g) 298 K.

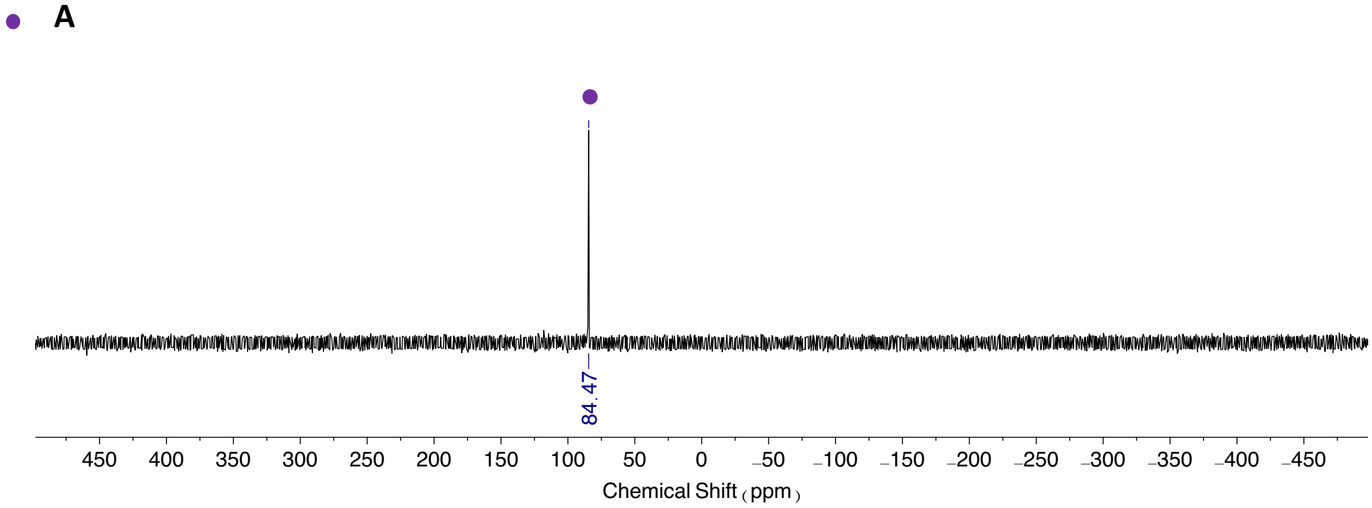

Figure S26: $^{31}P\{^{1}H\}$ NMR spectrum (162 MHz, d_8-THF, 233 K) of crystals of **1-Ce**.

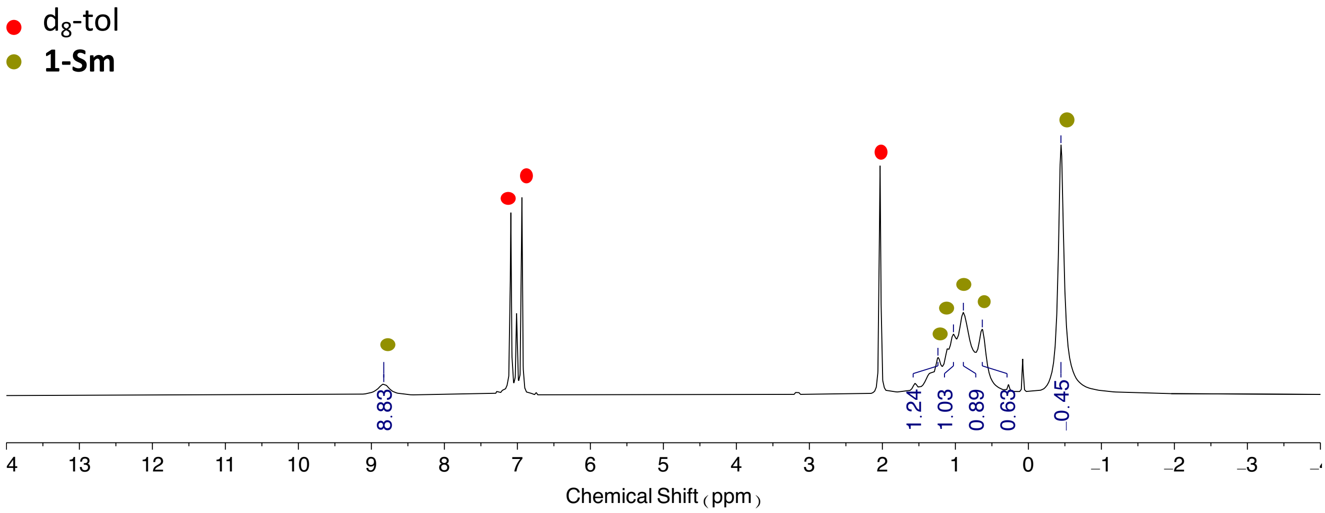

Figure S27: ^{1}H NMR spectrum (400 MHz, d_8-toluene, 233 K) of crystals of **1-Sm**.

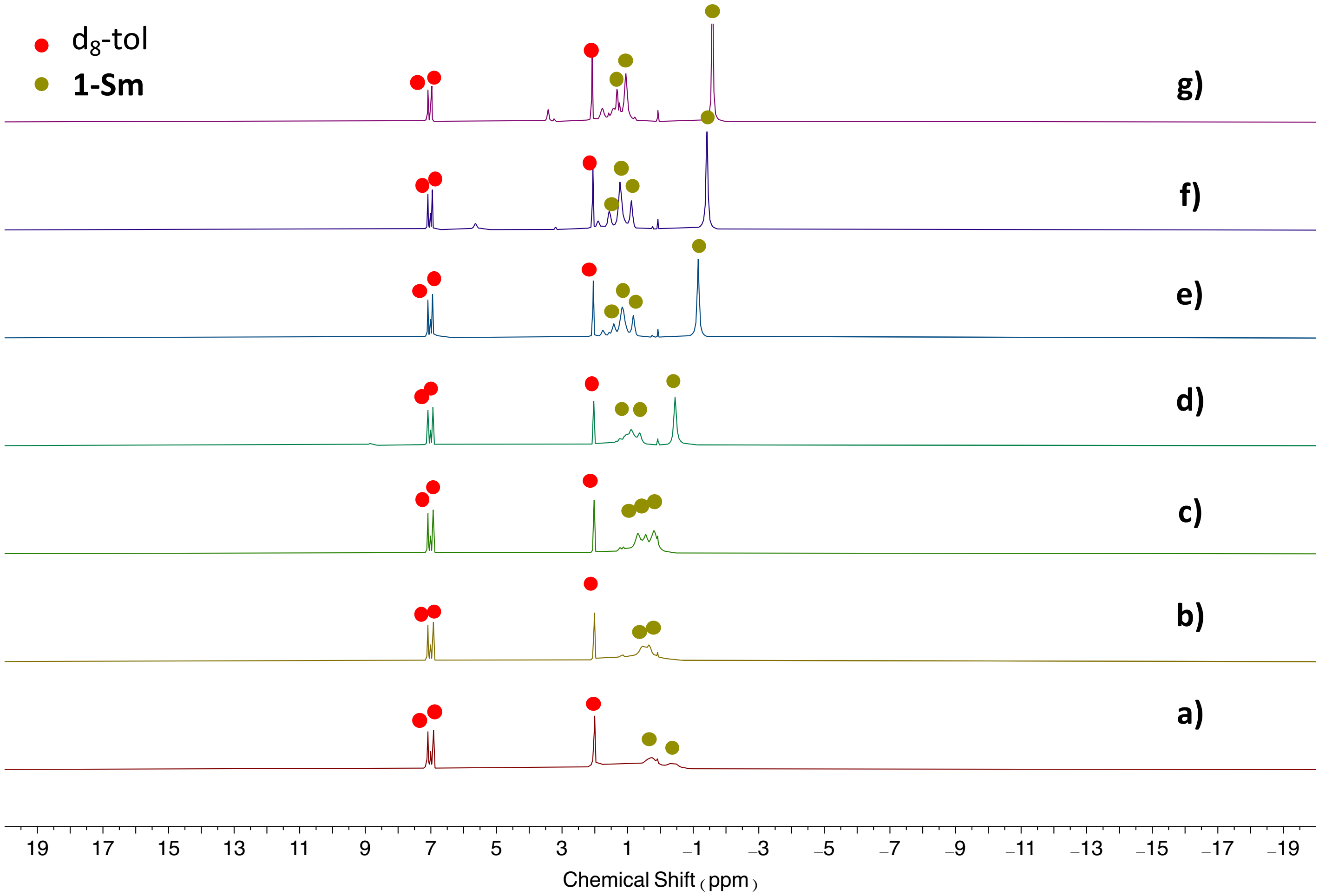

Figure S28: Variable temperature ^{1}H NMR spectra (400 MHz, d_8-toluene) of crystals of **1-Sm.** A) 193 K, b) 203 K, c) 218 K, d) 233 K, e) 248 K, f) 263 K, and g) 298 K.

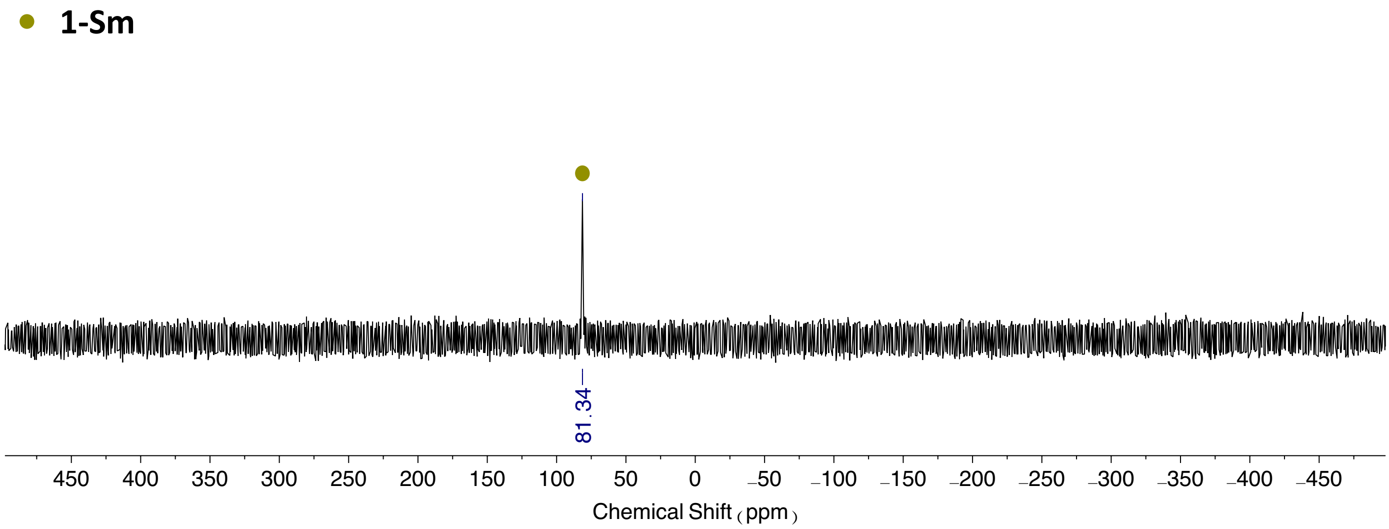

Figure S29: $^{31}P\{^{1}H\}$ NMR spectrum (162 MHz, d_8-toluene, 233 K) of crystals of **1-Sm**.

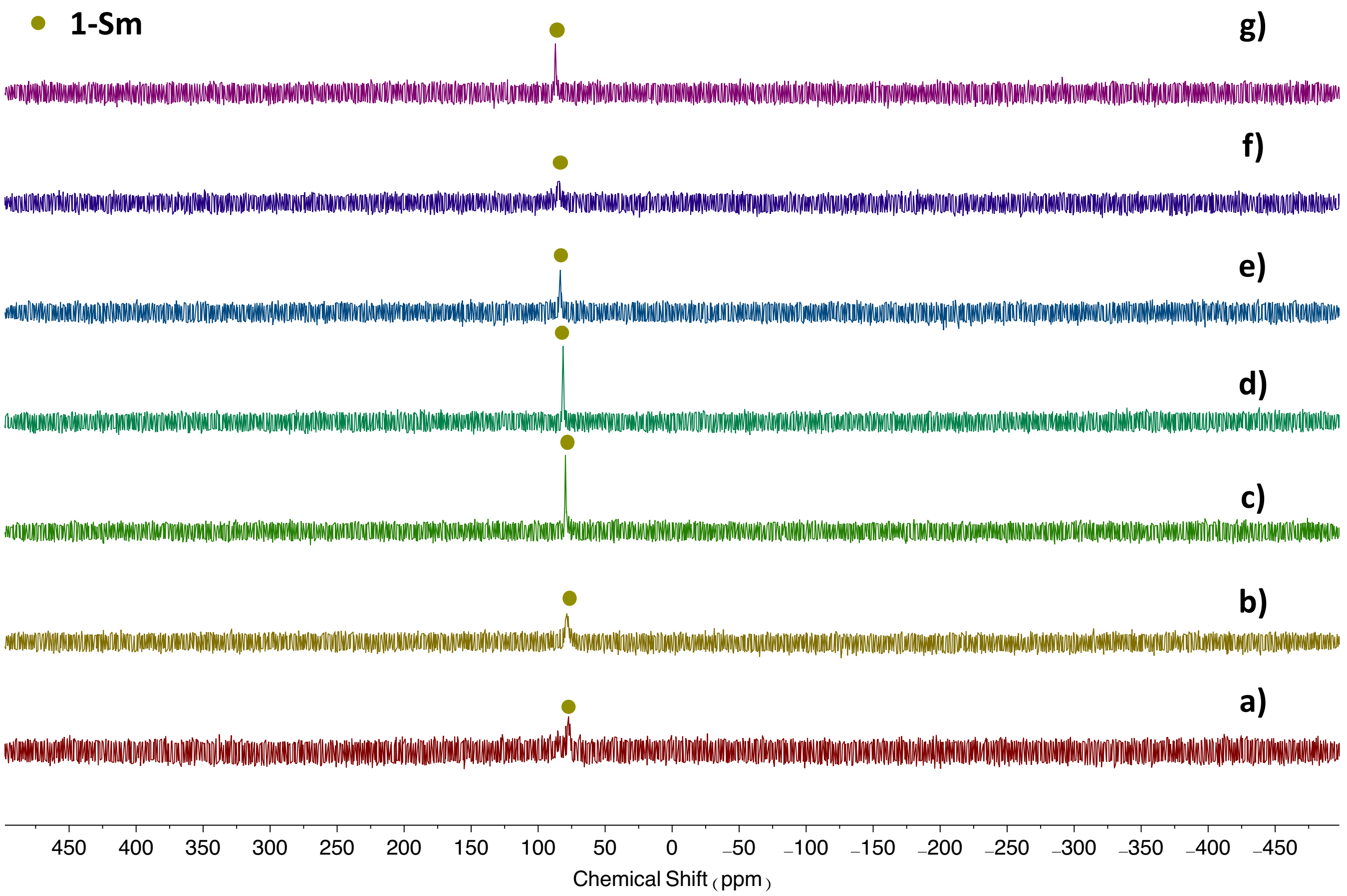

Figure S30: Variable temperature $^{31}P\{^{1}H\}$ NMR spectra (162 MHz, d_8-toluene) of crystals of **1-Sm** a) 193 K, b) 203 K, c) 218 K, d) 233 K, e) 248 K, f) 263 K, and g) 298 K.

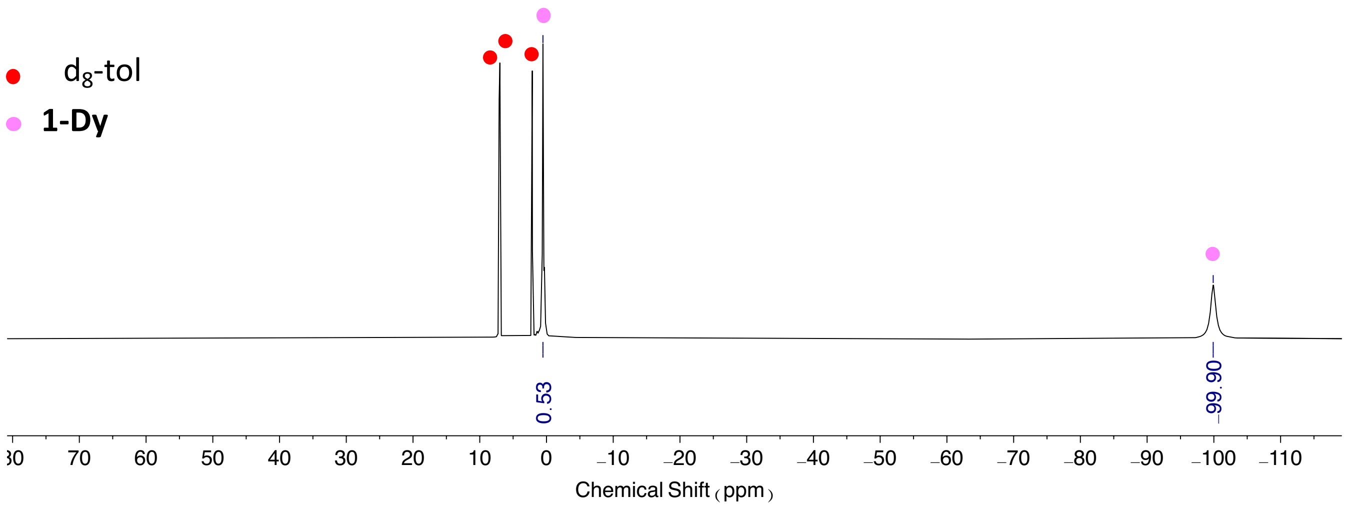

Figure S31: ^{1}H NMR spectrum (400 MHz, d$_8$-toluene, 298 K) of crystals of **1-Dy**.

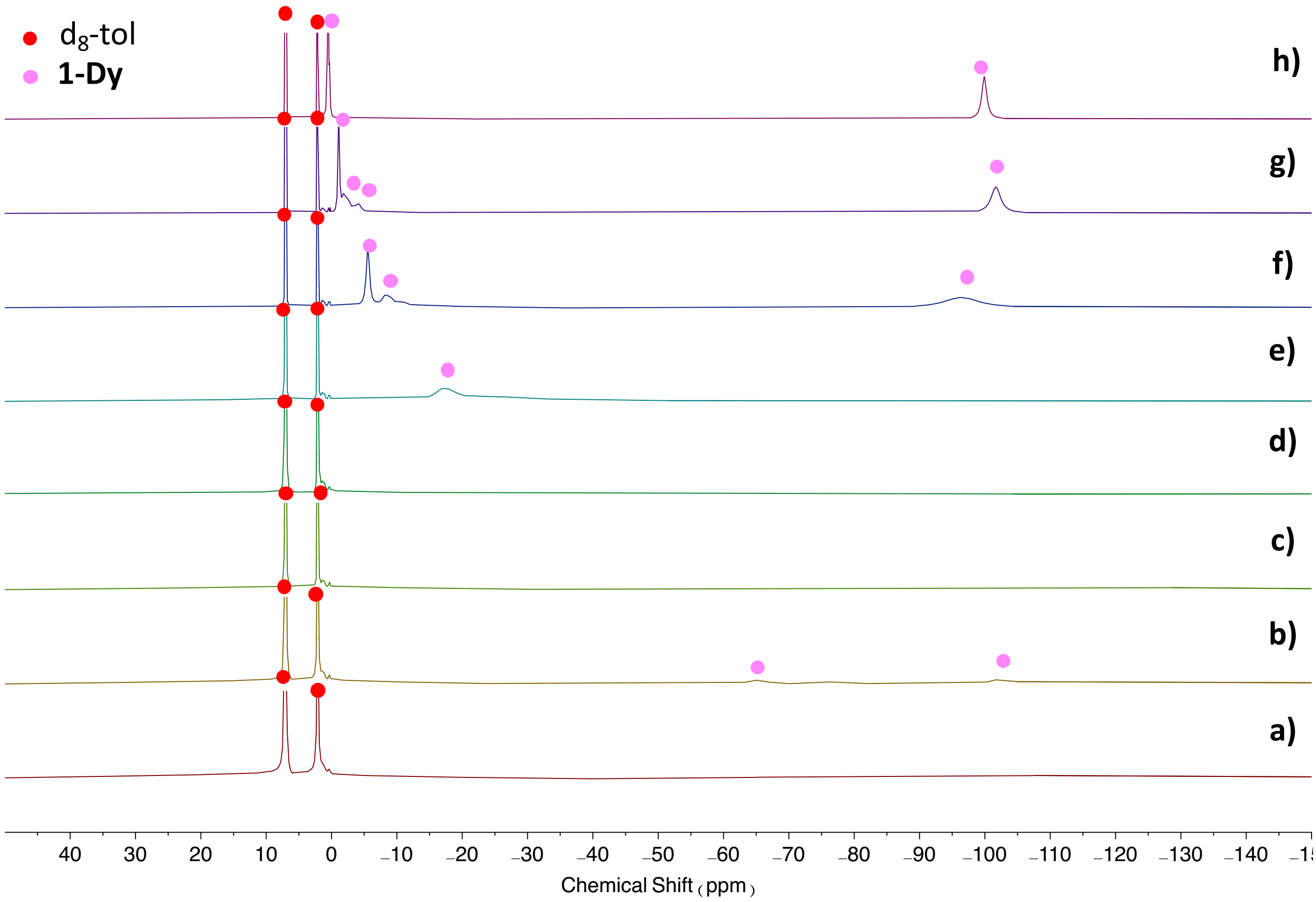

Figure S32: Variable temperature ^{1}H NMR spectra (400 MHz, d$_8$-toluene) of crystals of **1-Dy.** a) 193 K, b) 203 K, c) 218 K, d) 233 K, e) 248 K, f) 263 K, g) 278 K and h) 298 K.

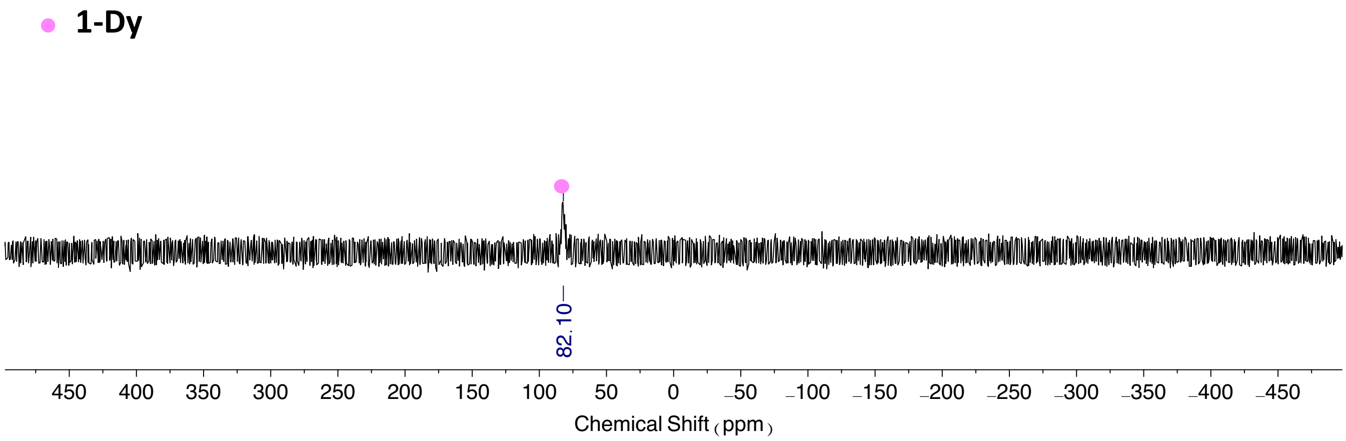

Figure S33: $^{31}P\{^{1}H\}$ NMR spectrum (162 MHz, d_8-toluene, 298 K) of crystals of **1-Dy**.

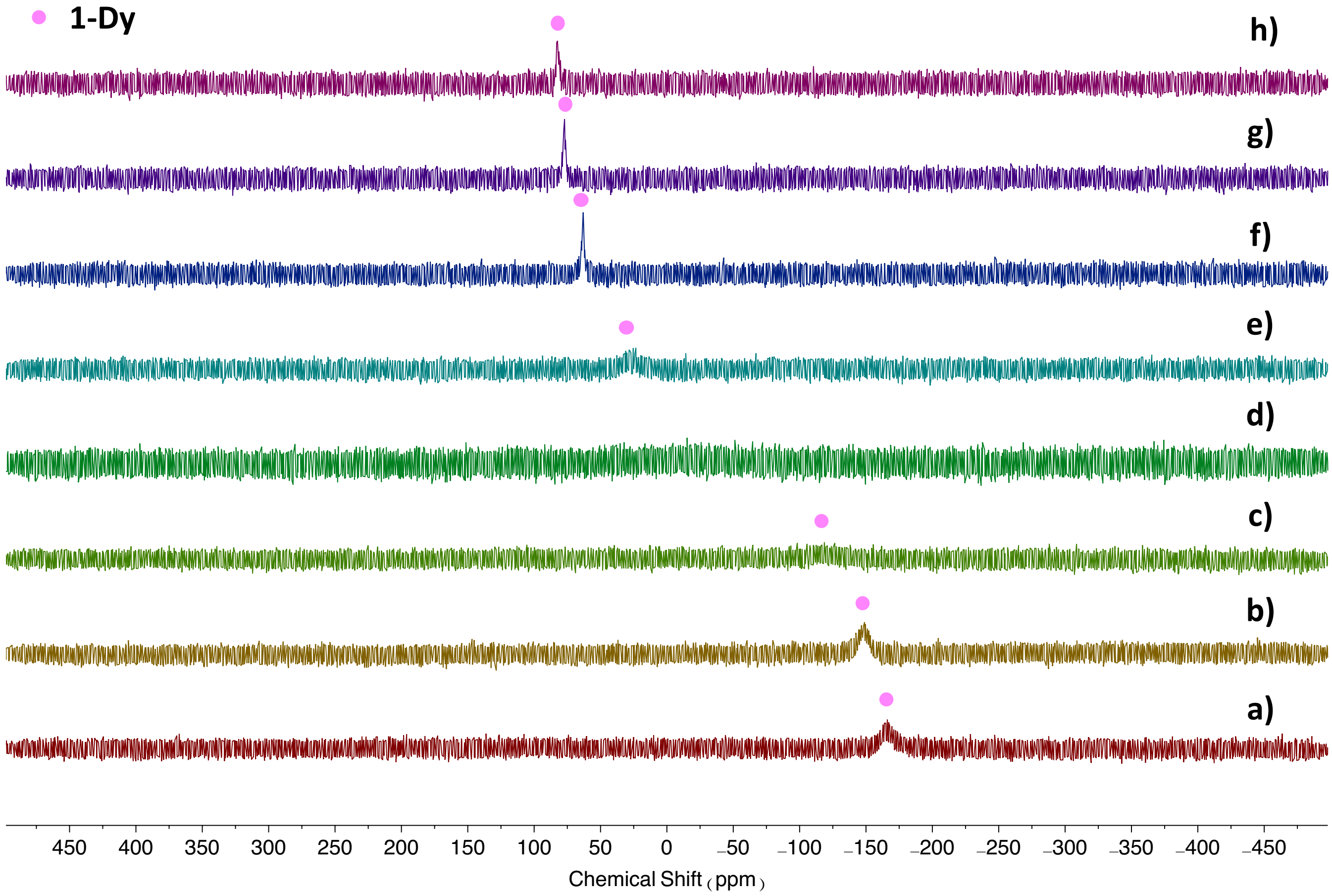

Figure S34: Variable temperature $^{31}P\{^{1}H\}$ NMR spectra (162 MHz, d_8-toluene) of crystals of **1-Dy** a) 193 K, b) 203 K, c) 218 K, d) 233 K, e) 248 K, f) 263 K, g) 278 K and h) 298 K.

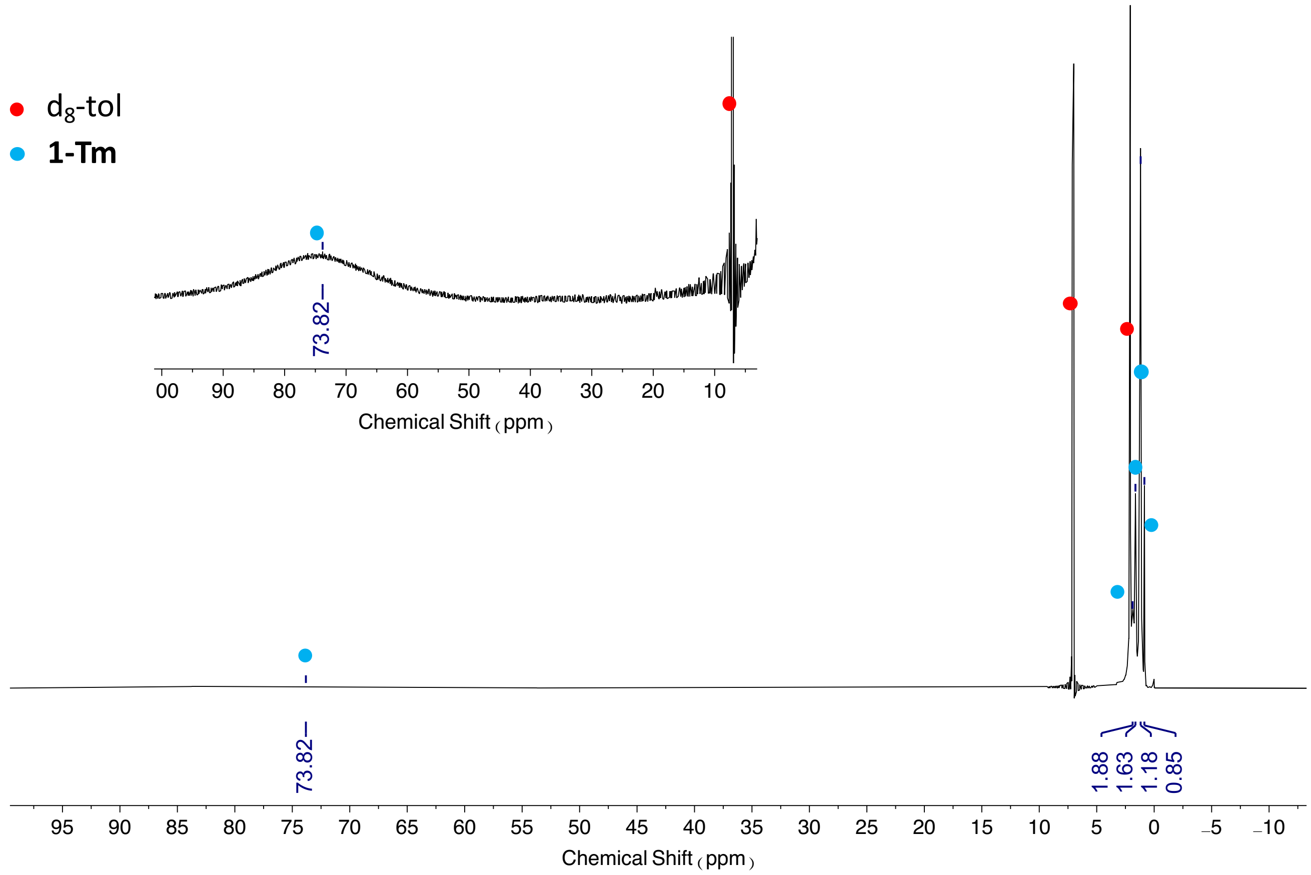

Figure S35: ^{1}H NMR spectrum (400 MHz, d_8-toluene, 298 K) of crystals of **1-Tm**.

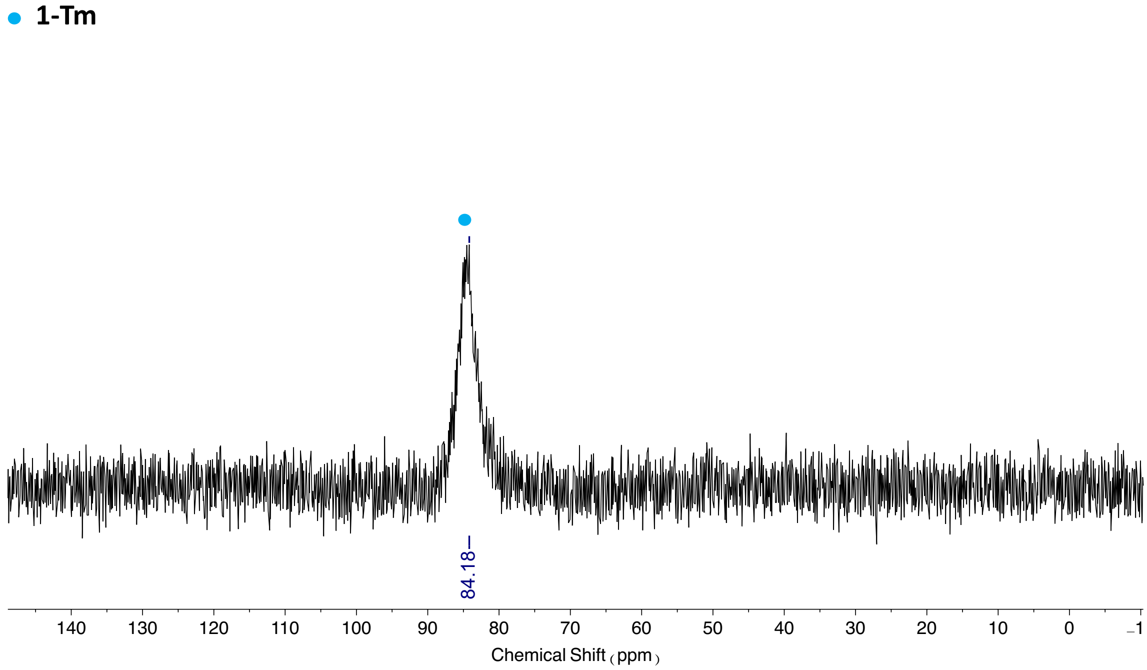

Figure S36: ^{31}P{^{1}H} NMR spectrum (162 MHz, d_8-toluene, 298 K) of crystals of **1-Tm**

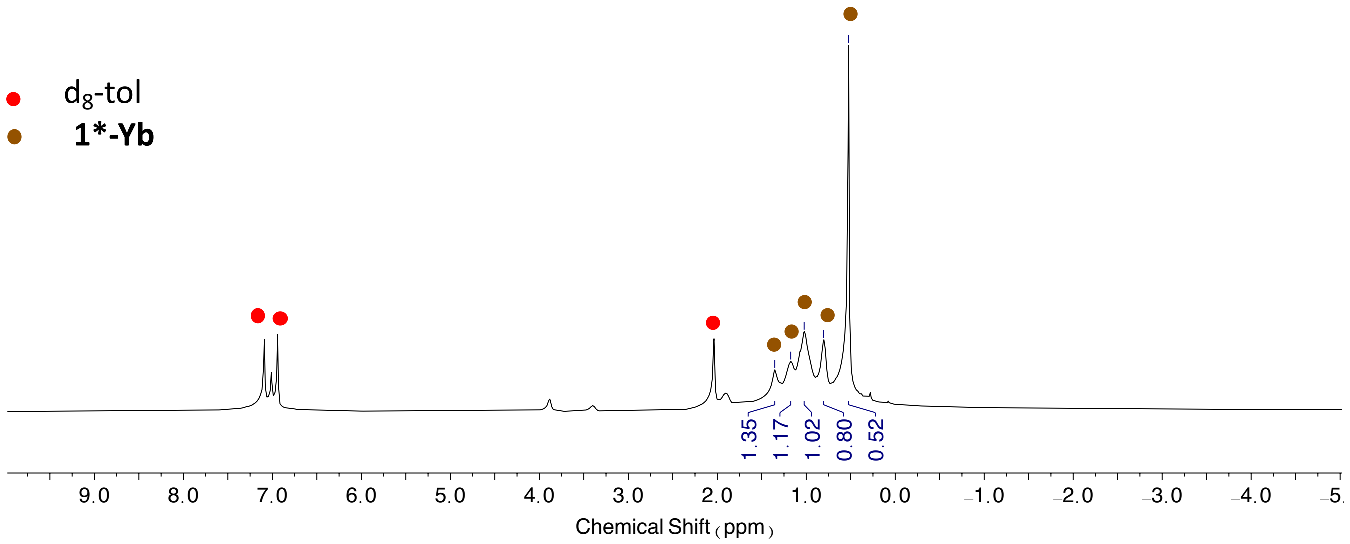

Figure S37: ^{1}H NMR spectrum (400 MHz, d_8-toluene, 233 K) of crystals of **1*-Yb**.

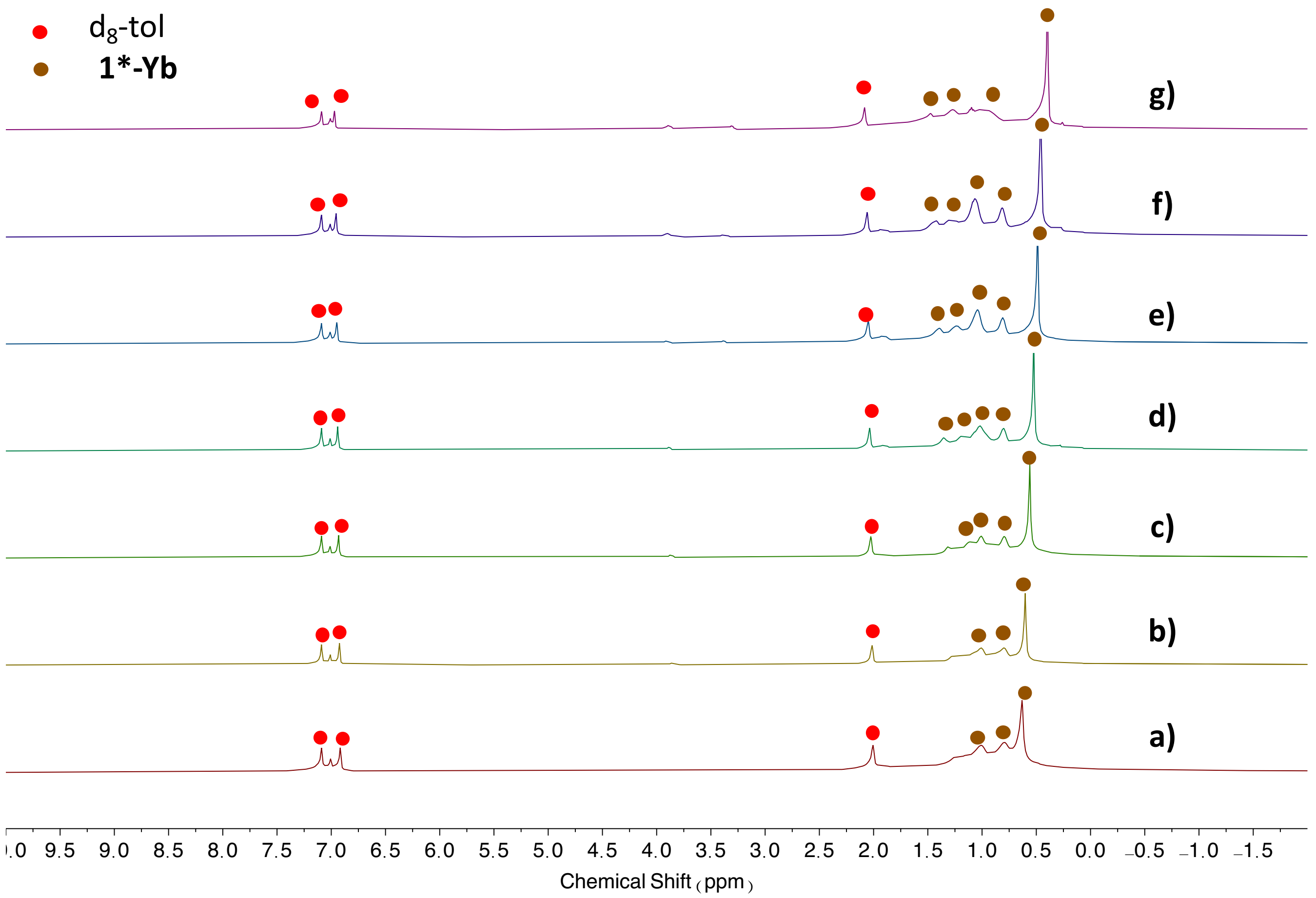

Figure S38: Variable temperature ^{1}H NMR spectra (400 MHz, d_8-toluene) of crystals of **1*-Yb.** a) 193 K, b) 203 K, c) 218 K, d) 233 K, e) 248 K, f) 263 K, and g) 298 K.

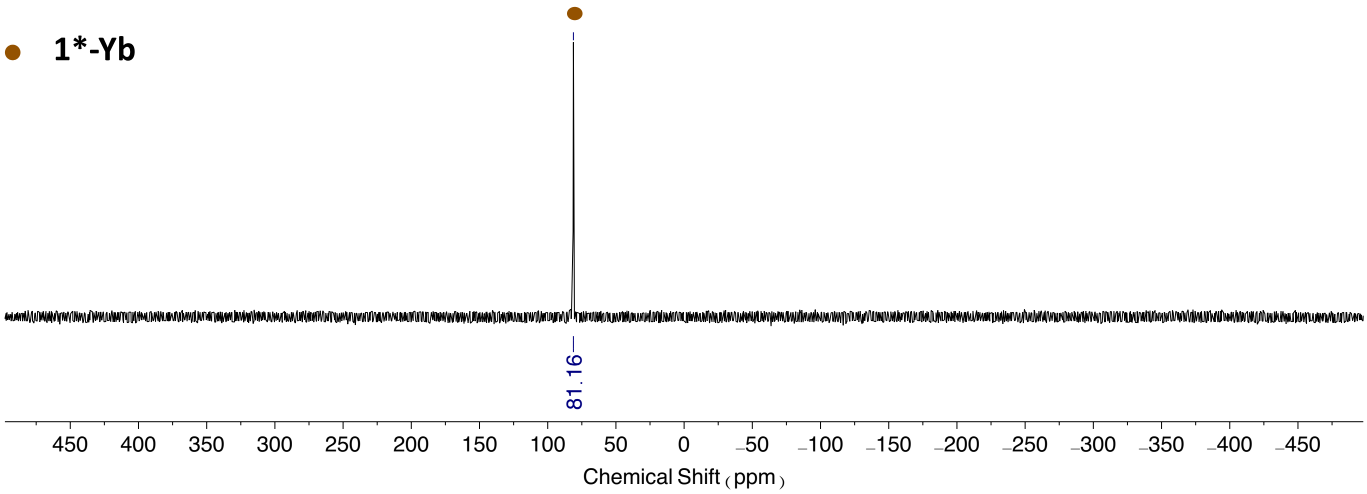

Figure S39: $^{31}P\{^{1}H\}$ NMR spectrum (162 MHz, d_8-toluene, 233 K) of crystals of **1*-Yb**.

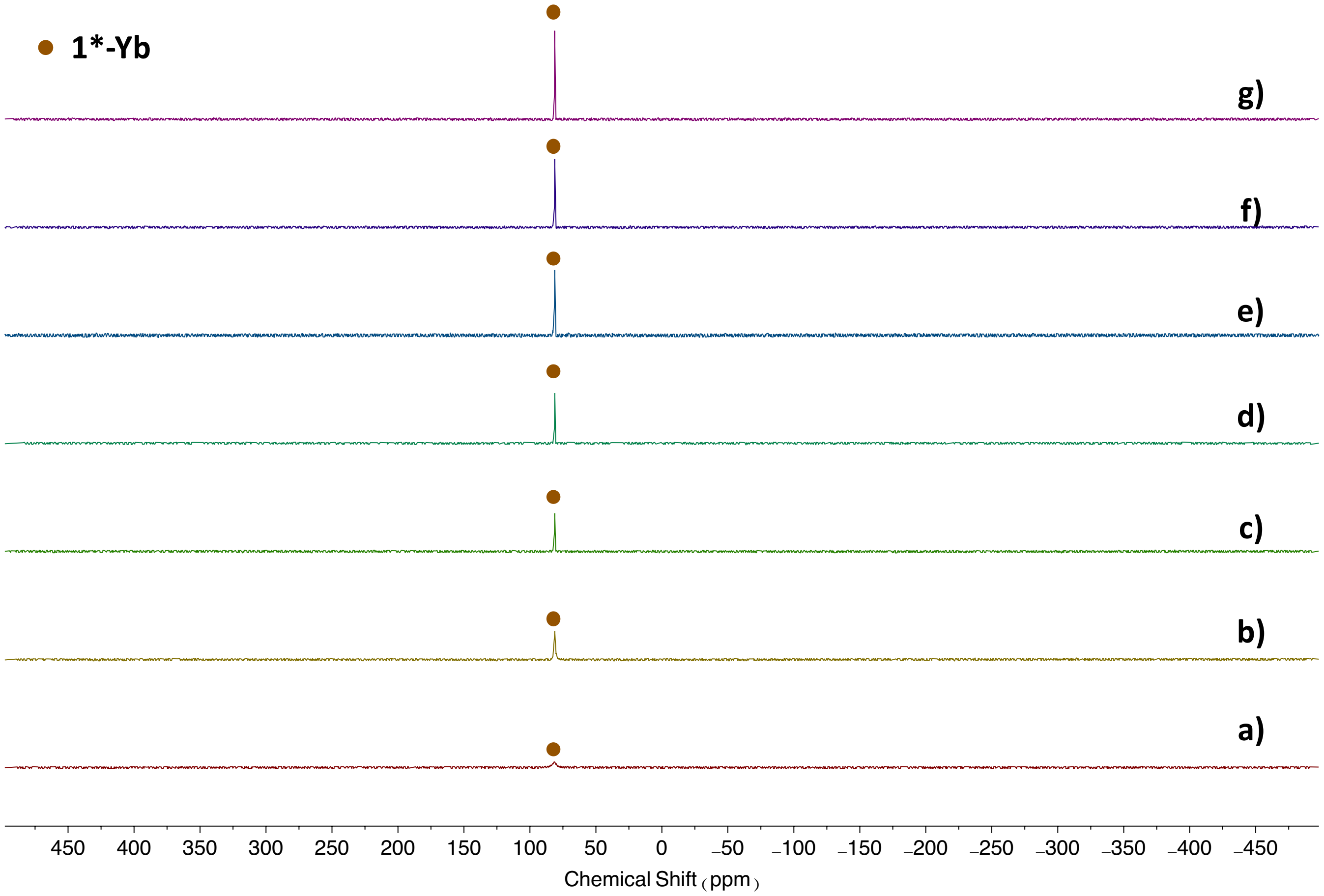

Figure S40: Variable temperature $^{31}P\{^{1}H\}$ NMR spectra (162 MHz, d_8-toluene) of crystals of **1*-Yb** a) 193 K, b) 203 K, c) 218 K, d) 233 K, e) 248 K, f) 263 K, and g) 298 K.

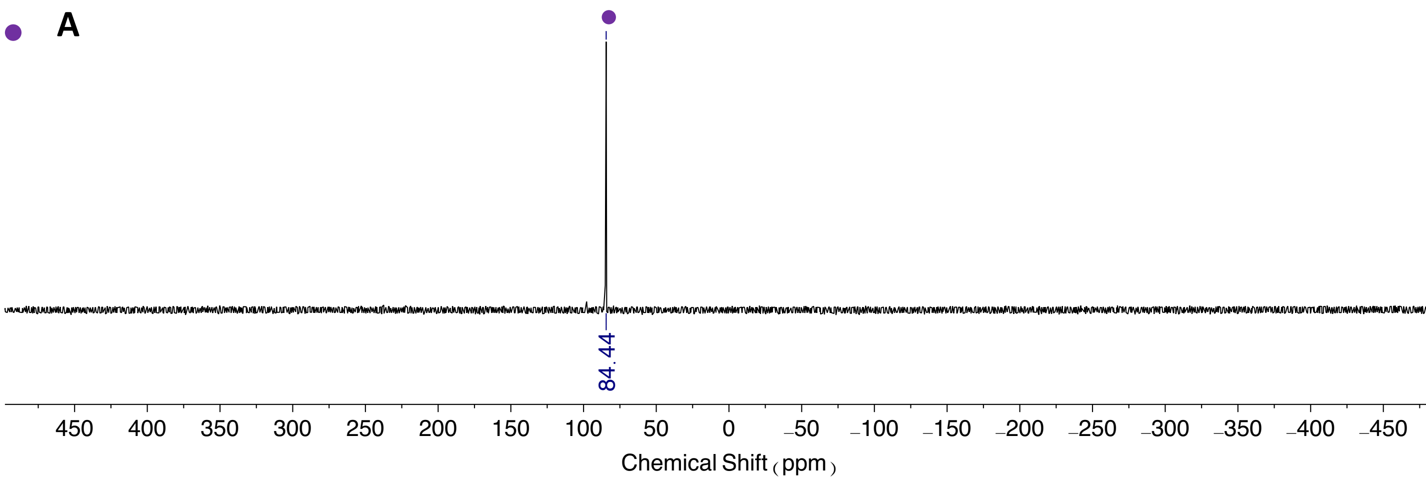

Figure S41: $^{31}P\{^{1}H\}$ NMR spectrum (162 MHz, d_8-THF, 233 K) of crystals of **1*-Yb**.

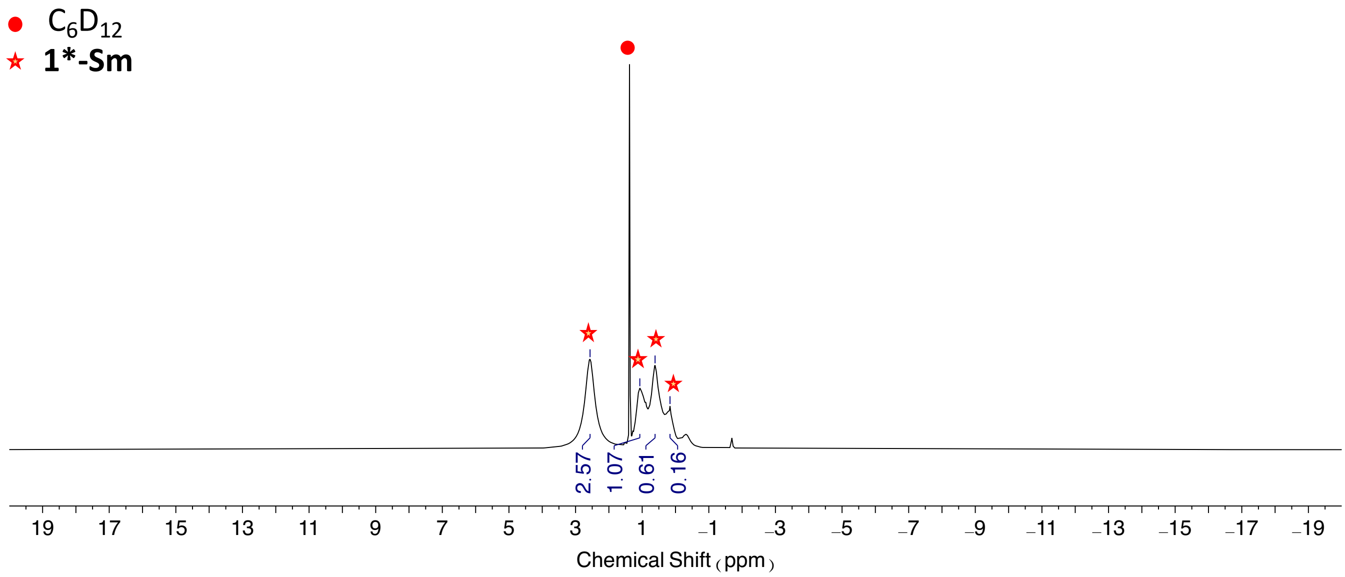

Figure S42: ^{1}H NMR spectrum (400 MHz, C_6D_{12}, 298 K) of crystals of **1*-Sm**.

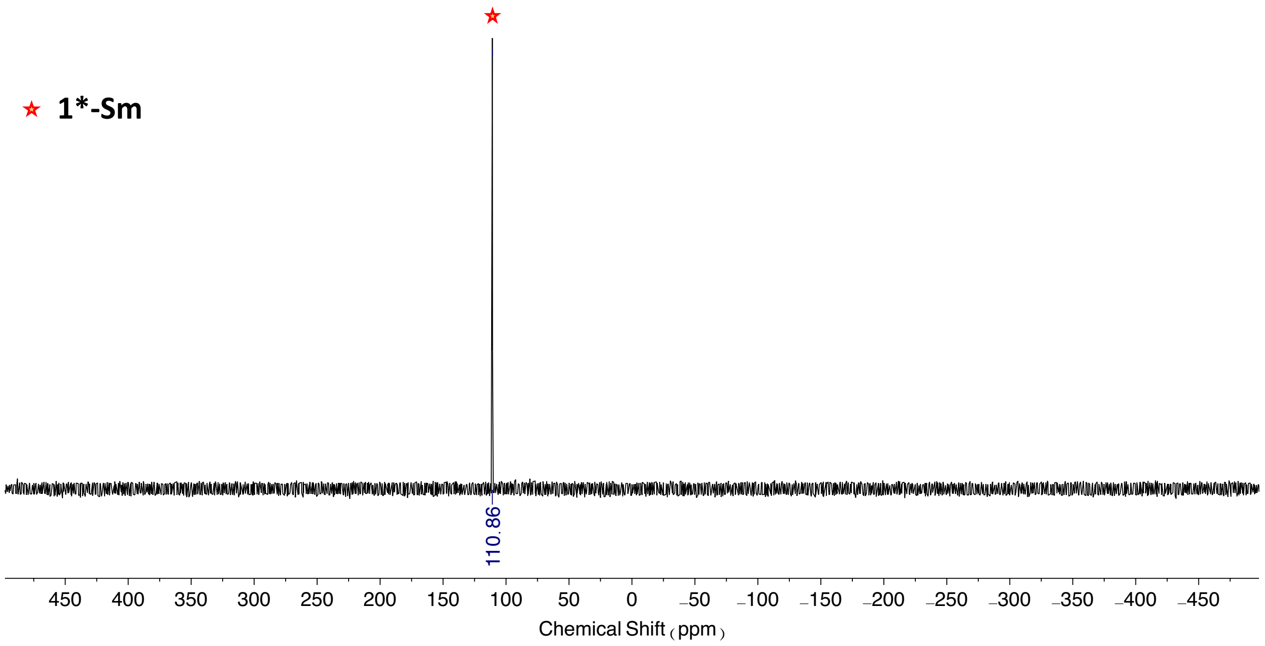

Figure S43: $^{31}P\{^1H\}$ NMR spectrum (162 MHz, C_6D_{12}, 298 K) of crystals of **1*-Sm**.

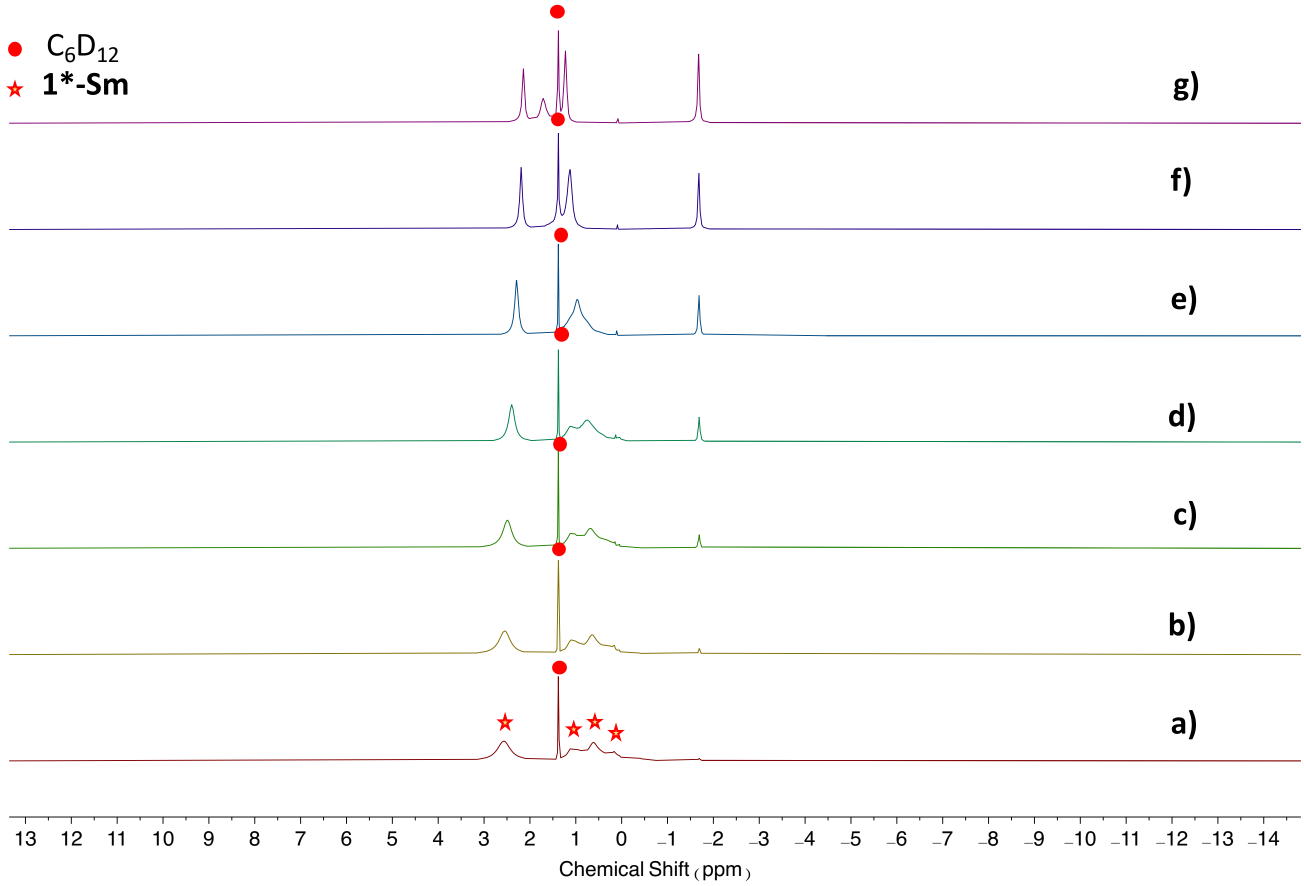

Figure S44: ^1H NMR spectrum (400 MHz, C_6D_{12}, 298 K) evolution of crystals of **1*-Sm** in cyclohexane solution a) immediately, b) after 30 min, c) after 1 h, d) after 3 h, e) after 6 h, f) after 12 h, and g) after 24 h at 25 °C.

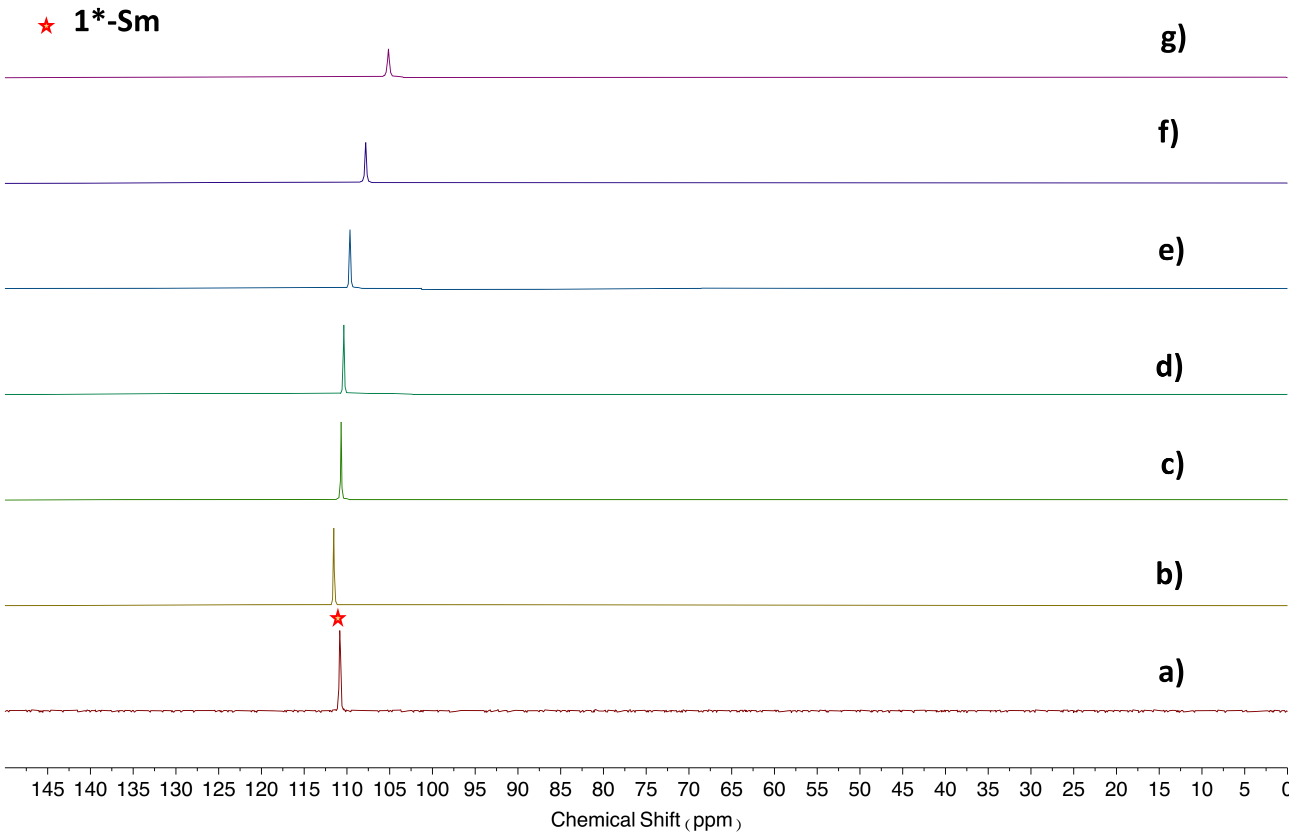

Figure S45: $^{31}P\{^1H\}$ NMR spectrum (162 MHz, C_6D_{12}, 298 K) evolution of crystals of **1*-Sm** in cyclohexane solution a) immediately, b) after 30 min, c) after 1 h, d) after 3 h, e) after 6 h, f) after 12 h, and g) after 24 h at 25 °C.

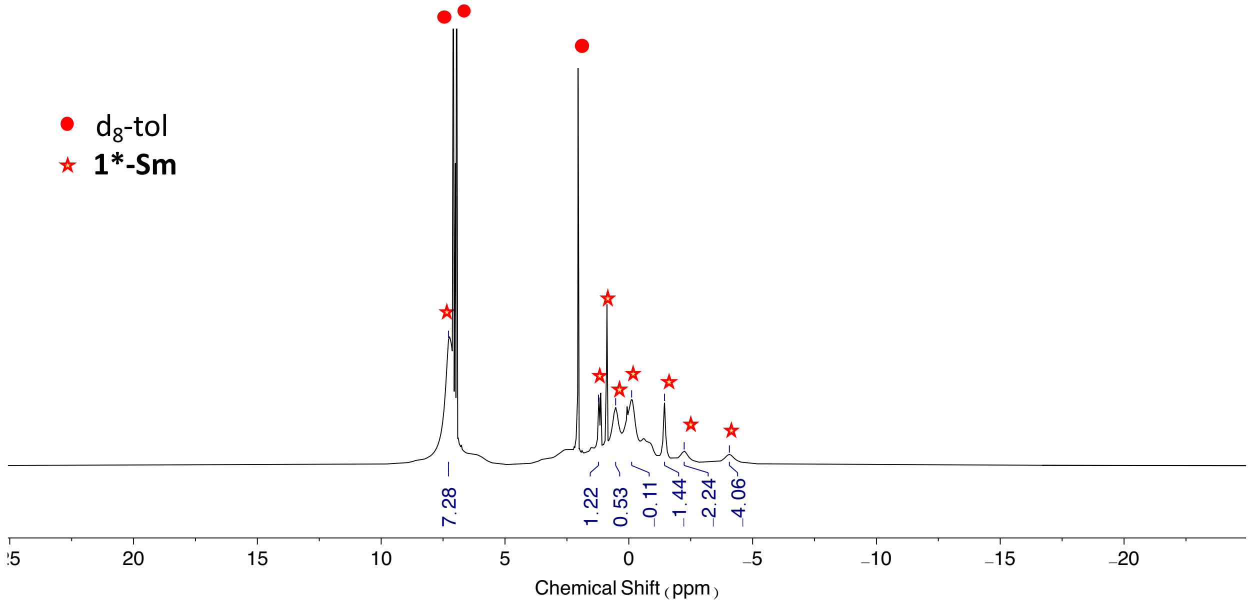

Figure S46: ^1H NMR spectrum (400 MHz, d_8-toluene, 233 K) of crystals of **1*-Sm** immediately after dissolution in toluene at −40 °C.

.

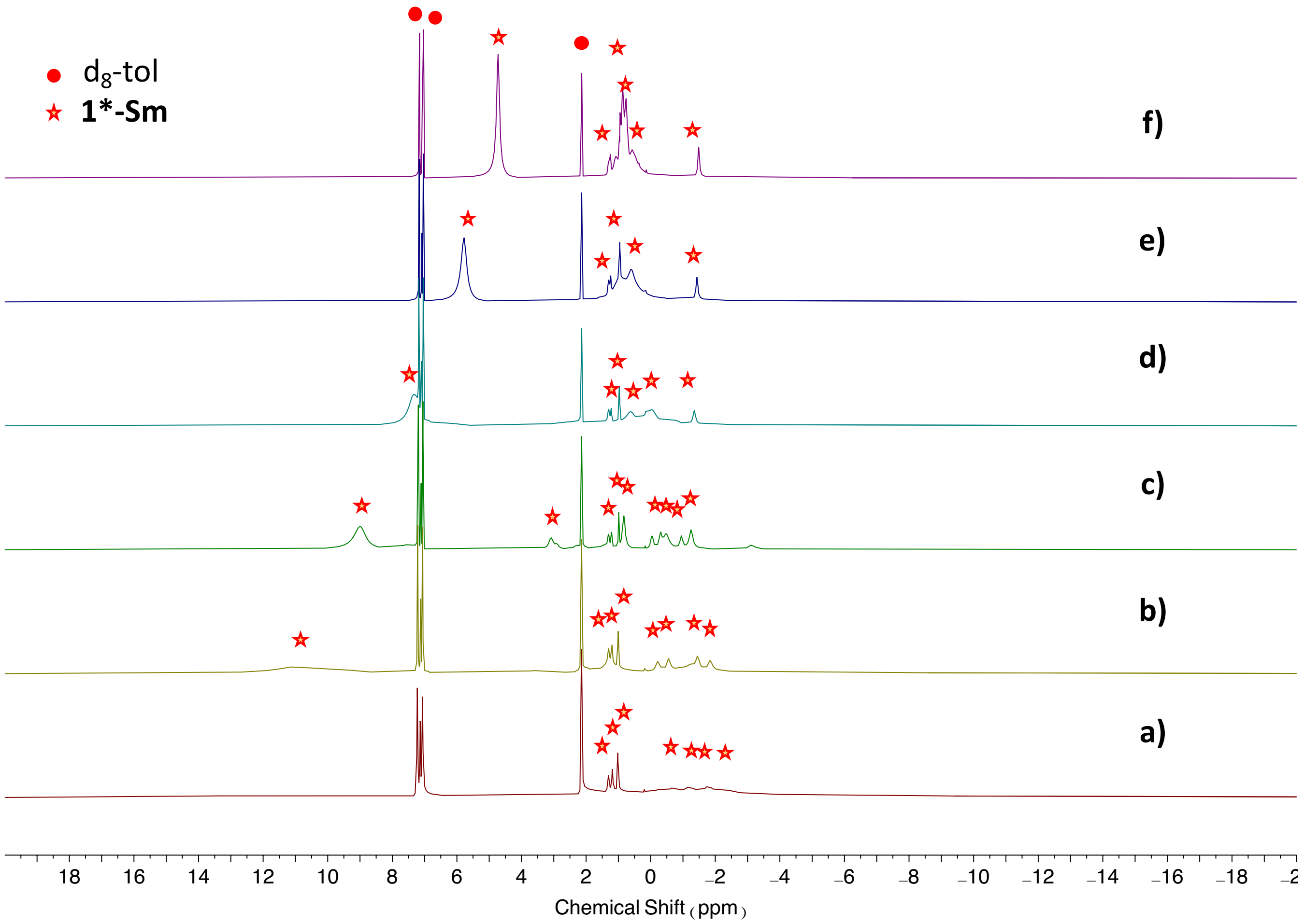

Figure S47: Variable temperature ^{1}H NMR spectra (400 MHz, d_8-toluene) of crystals of **1*-Sm.** a) 193 K, b) 203 K, c) 218 K, d) 233 K, e) 248 K, and f) 263 K immediately after dissolution in toluene at −40 °C.

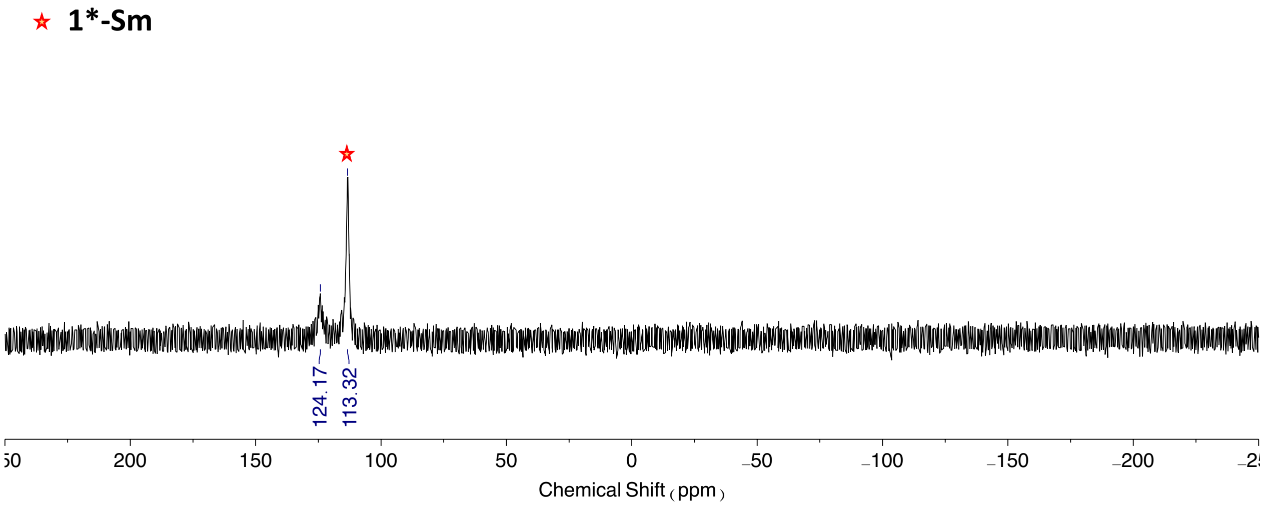

Figure S48: $^{31}P\{^{1}H\}$ NMR spectrum (162 MHz, d_8-toluene, 233 K) of crystals of **1*-Sm** immediately after dissolution in toluene at −40 °C.

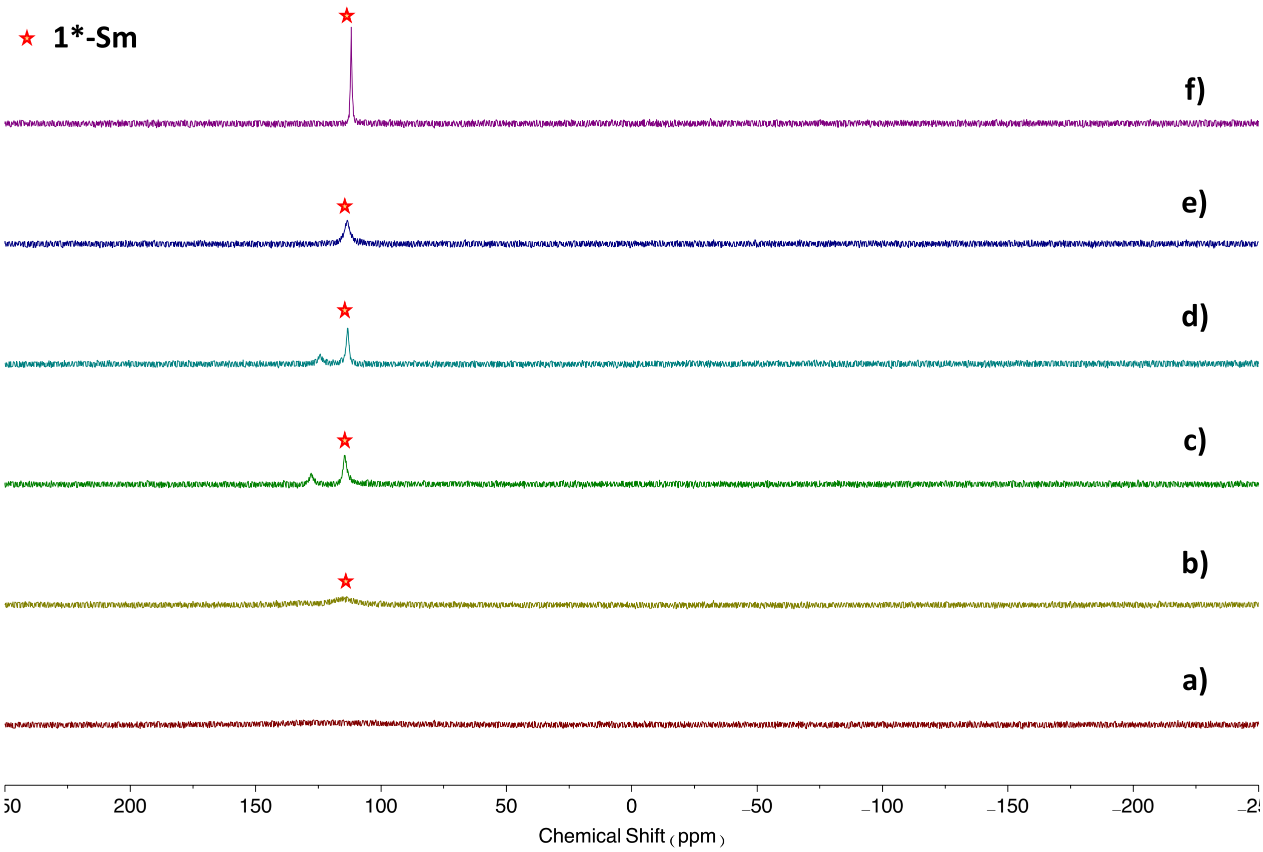

Figure S49: Variable temperature $^{31}P\{^{1}H\}$ NMR spectra (162 MHz, d_8-toluene) of crystals of **1*-Sm** a) 193 K, b) 203 K, c) 218 K, d) 233 K, e) 248 K, and f) 263 K immediately after dissolution in toluene at −40 °C.

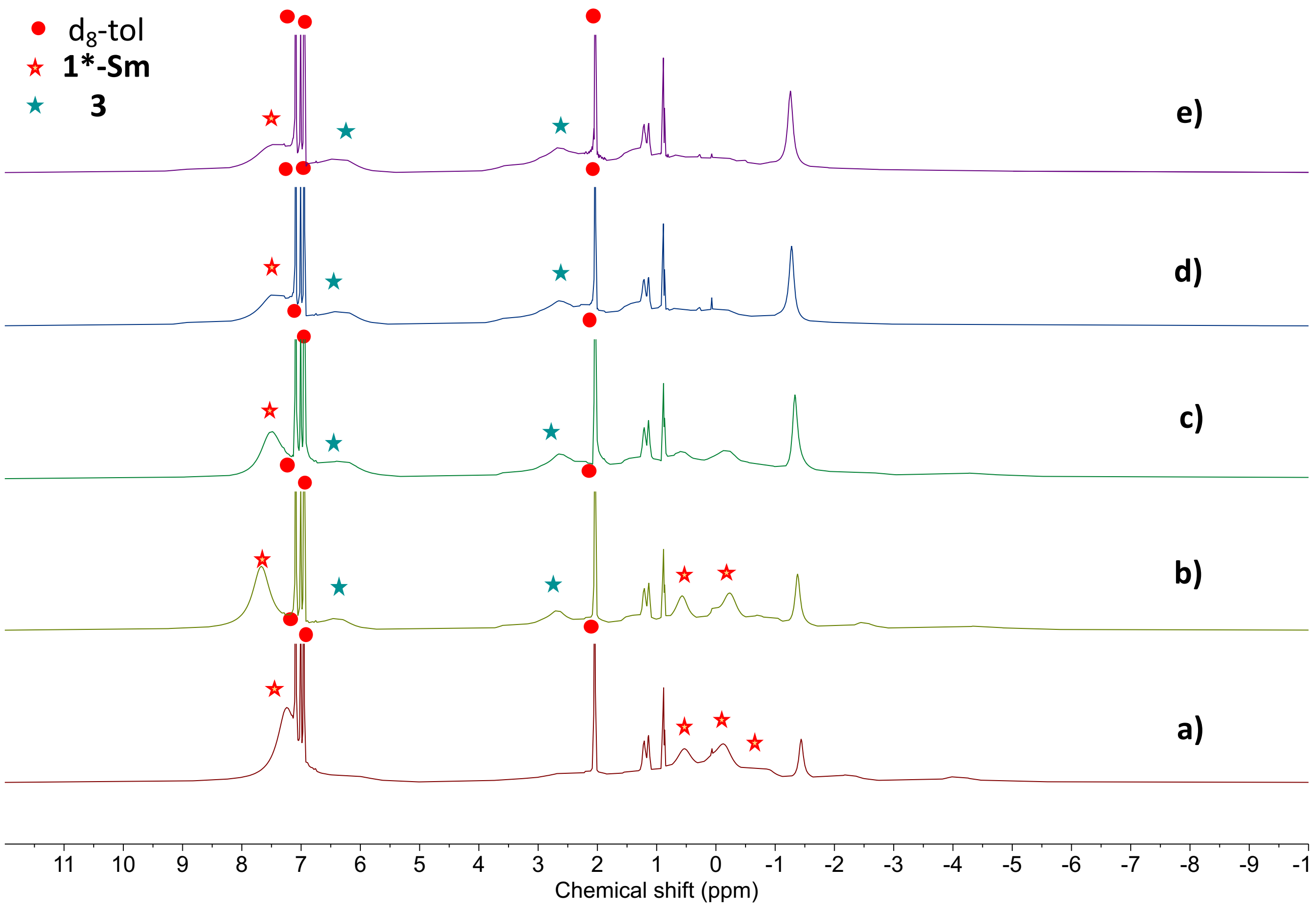

Figure S50: ^{1}H NMR spectrum (400 MHz, d_8-toluene, 233 K) evolution of crystals of **1*-Sm** in toluene solution a) immediately, b) after 24 h, c) after 5 days, d) after 14 days, and e) after 21 days at −40 °C.

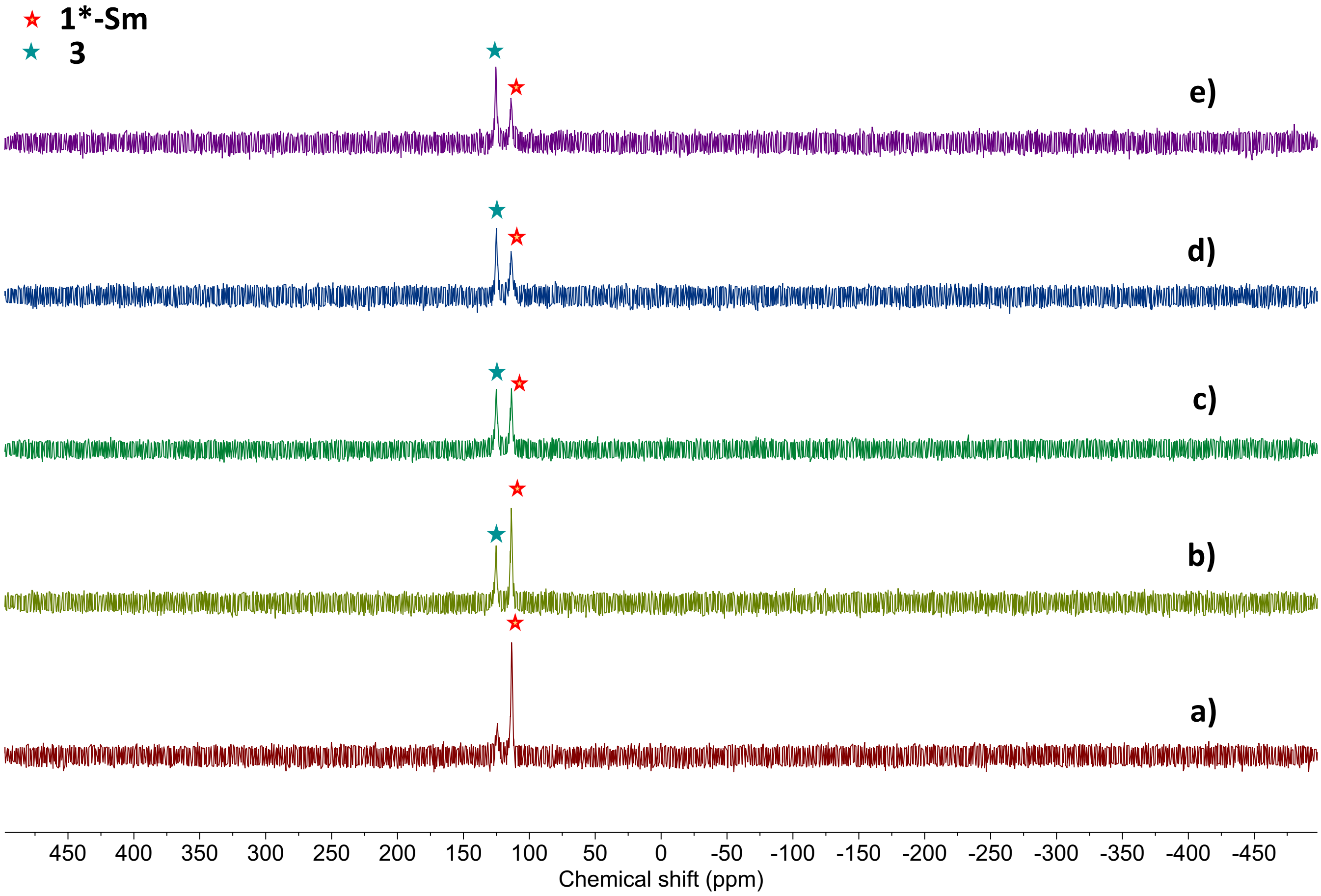

Figure S51: $^{31}P\{^{1}H\}$ NMR spectrum (162 MHz, d_8-toluene, 233 K) evolution of crystals of **1*-Sm** in toluene solution a) immediately, b) after 24 h, c) after 5 days, d) after 14 days, and e) after 21 days at −40 °C.

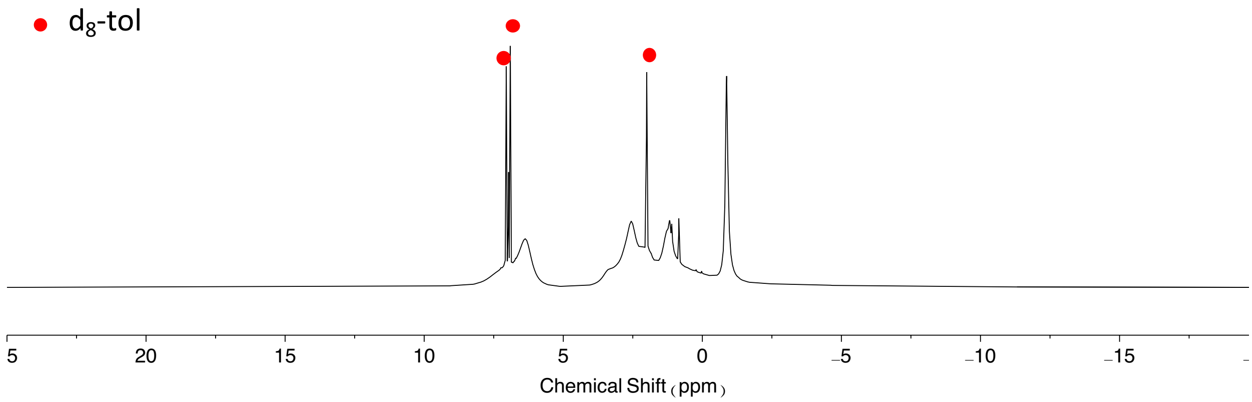

Figure S52: ^{1}H NMR spectrum (400 MHz, d_8-toluene, 233 K) of the reaction mixture obtained after addition of **A** to $[Sm^{II}\{N(SiMe_3)_2\}_2]_2$ in toluene at −40 °C under Ar.

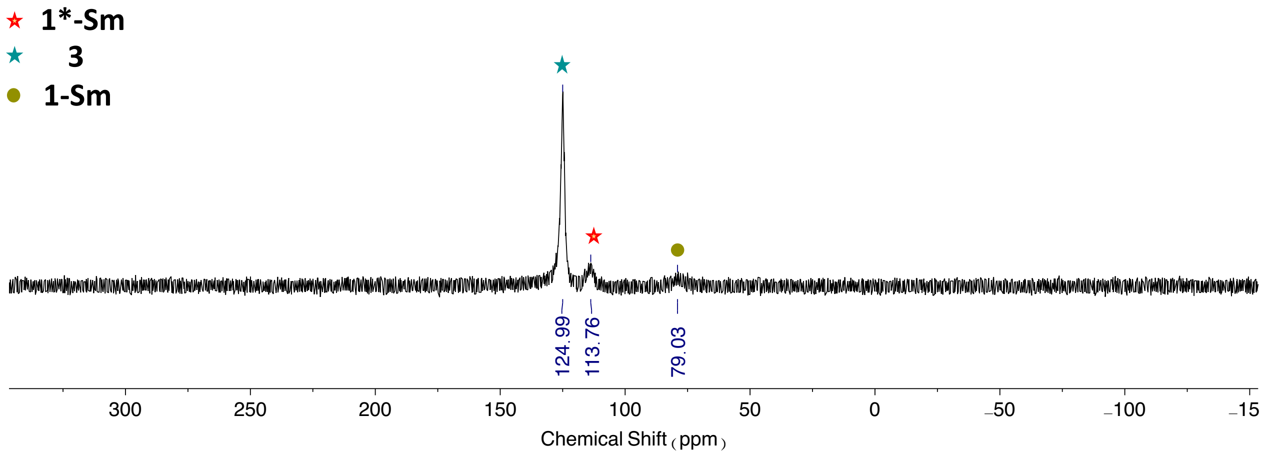

Figure S53: $^{31}P\{^{1}H\}$ NMR spectrum (162 MHz, d_8-toluene, 233 K) of the reaction mixture obtained after addition of **A** to $[Sm^{II}\{N(SiMe_3)_2\}_2]_2$ in toluene at −40 °C under Ar.

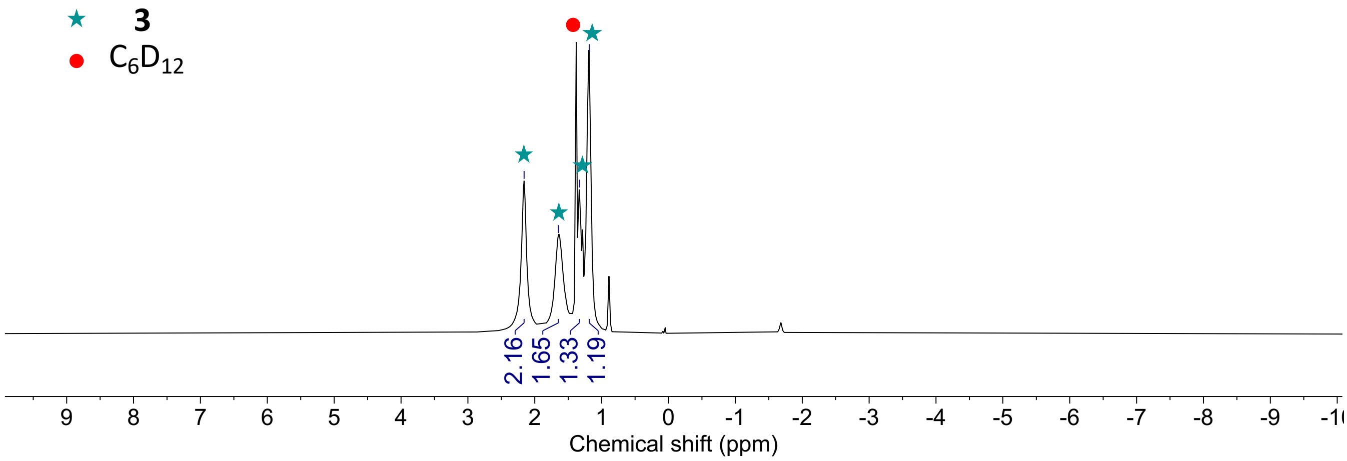

Figure S54: ^{1}H NMR spectrum (400 MHz, C_6D_{12}, 298 K) of crystals of **3**.

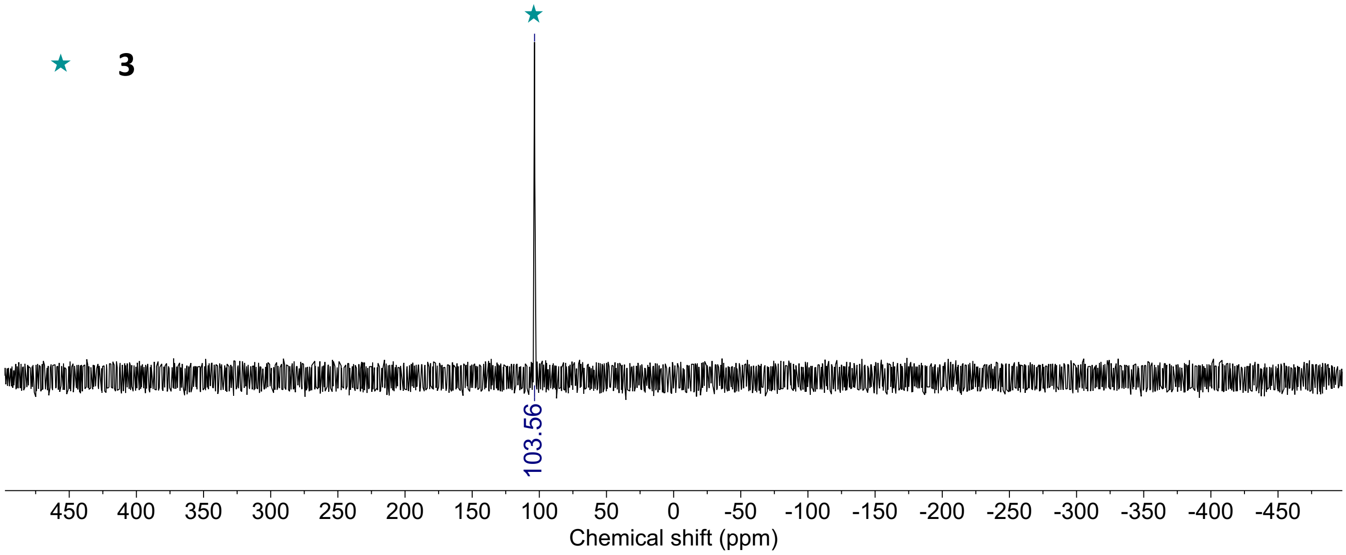

Figure S55: $^{31}P\{^{1}H\}$ NMR spectrum (162 MHz, C_6D_{12}, 298 K) of crystals of **3**.

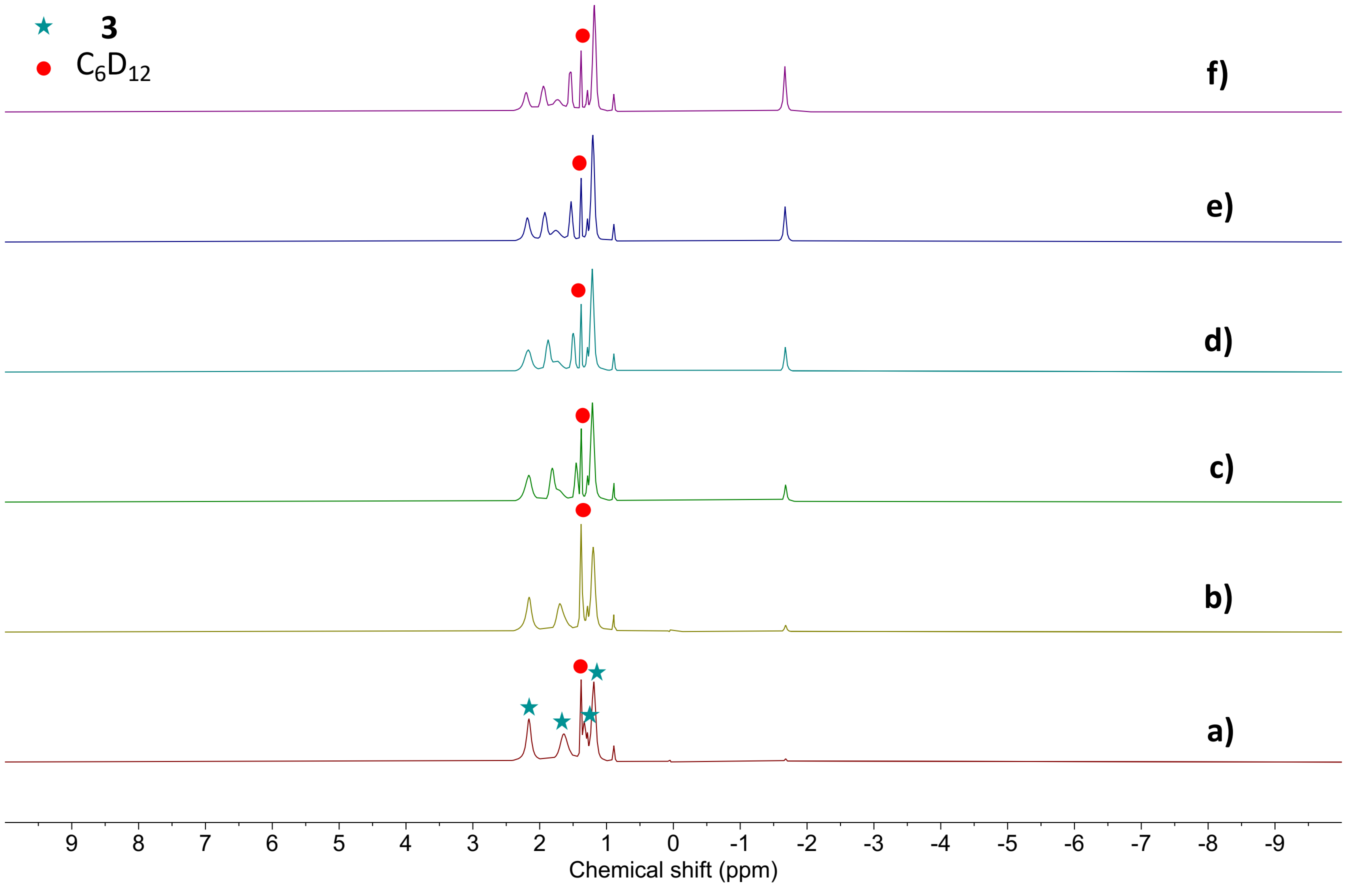

Figure S56: ^{1}H NMR spectrum (400 MHz, C_6D_{12}, 298 K) evolution of crystals of **3** in cyclohexane solution a) immediately, b) after 3 h, c) after 24 h, d) after 2 days, e) after 4 days, and f) after 7 days at 25 °C.

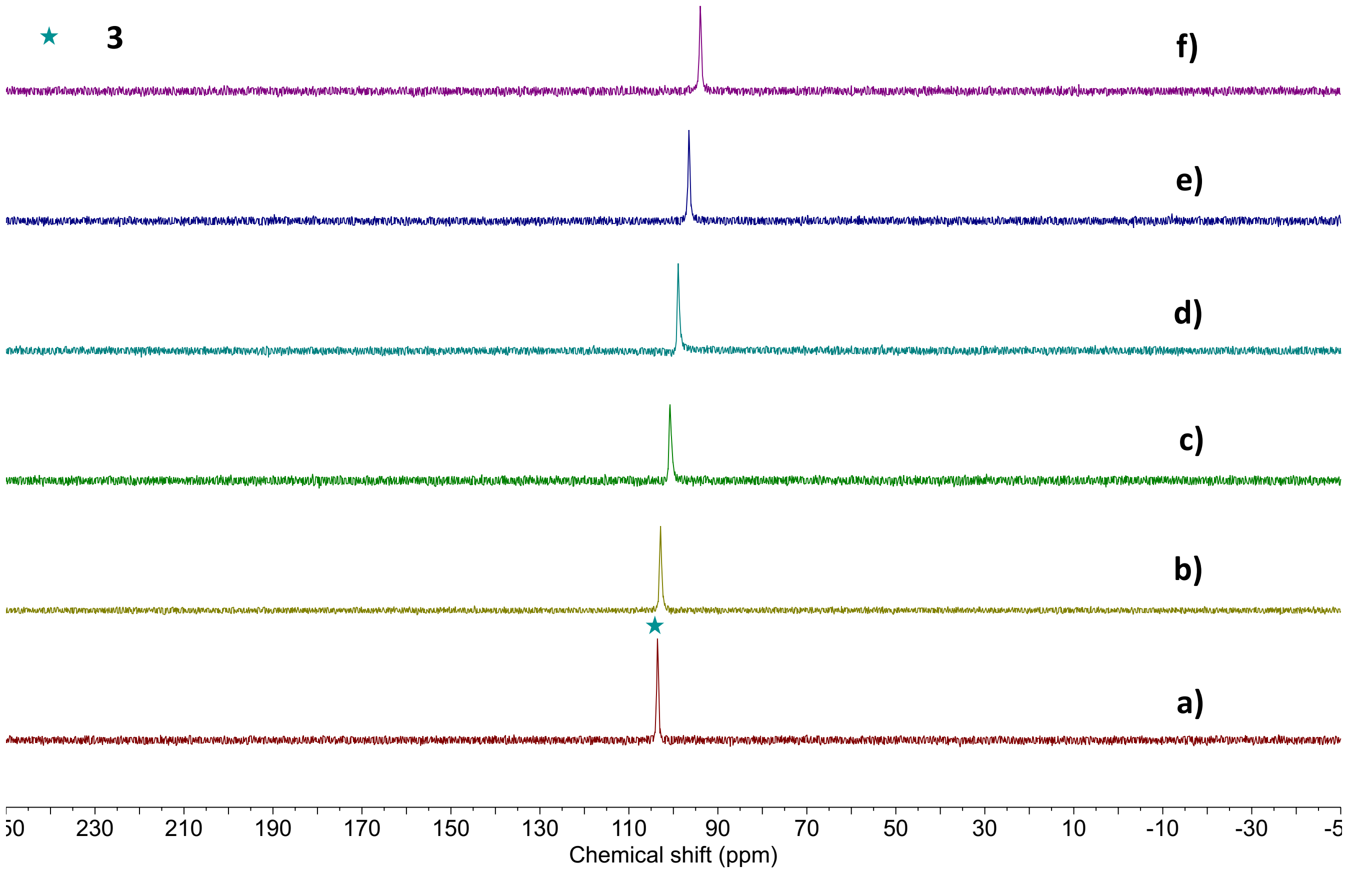

Figure S57: $^{31}P\{^1H\}$ NMR spectrum (162 MHz, C_6D_{12}, 298 K) evolution of crystals of **3** in cyclohexane solution a) immediately, b) after 3 h, c) after 24 h, d) after 2 days, e) after 4 days, and f) after 7 days at 25 °C.

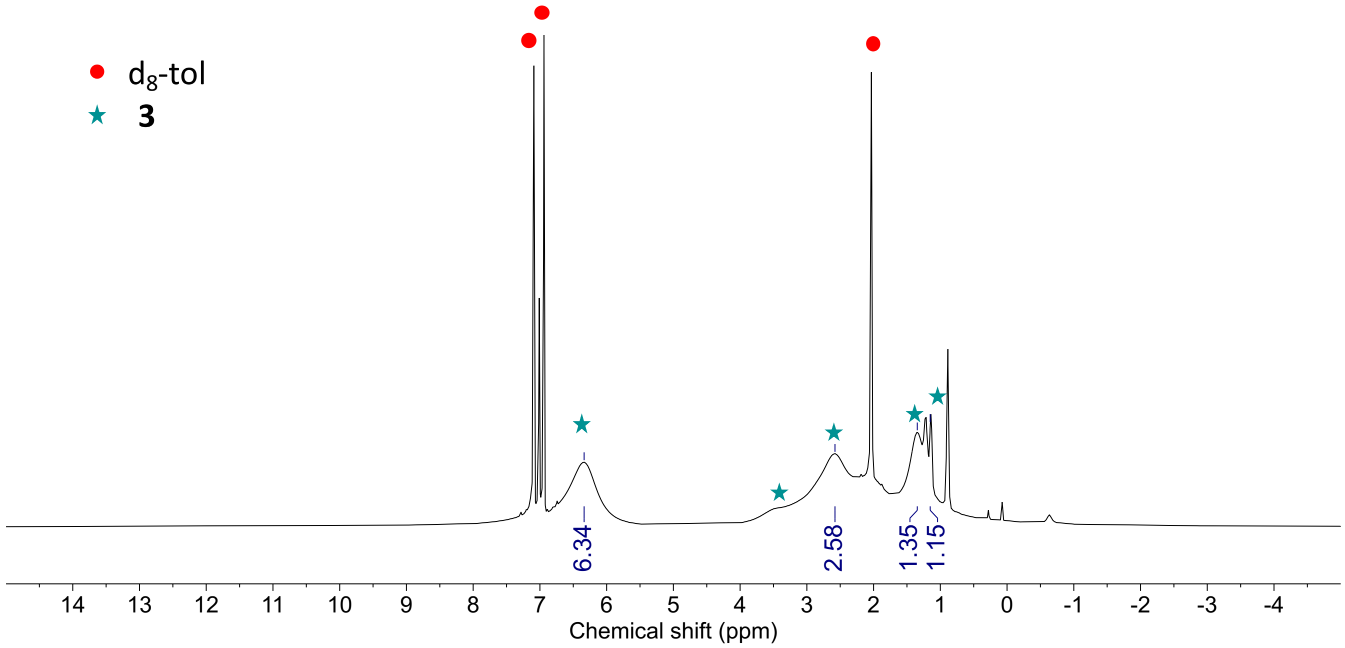

Figure S58: ^1H NMR spectrum (400 MHz, d_8-toluene, 233 K) of crystals of **3**.

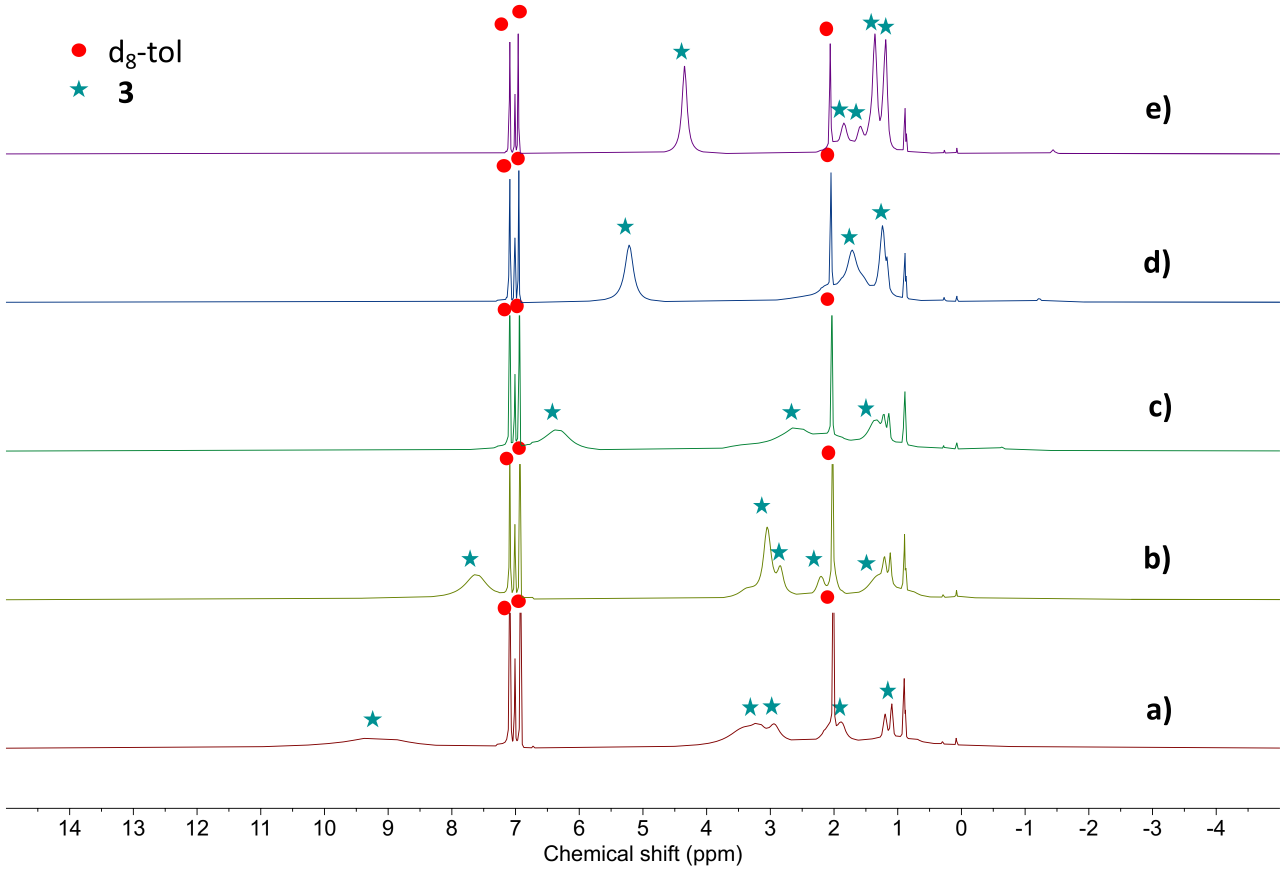

Figure S59: Variable temperature ^{1}H NMR spectra (400 MHz, d_8-toluene) of crystals of **3** a) 203 K, b) 218 K, c) 233 K, d) 248 K, and e) 263 K.

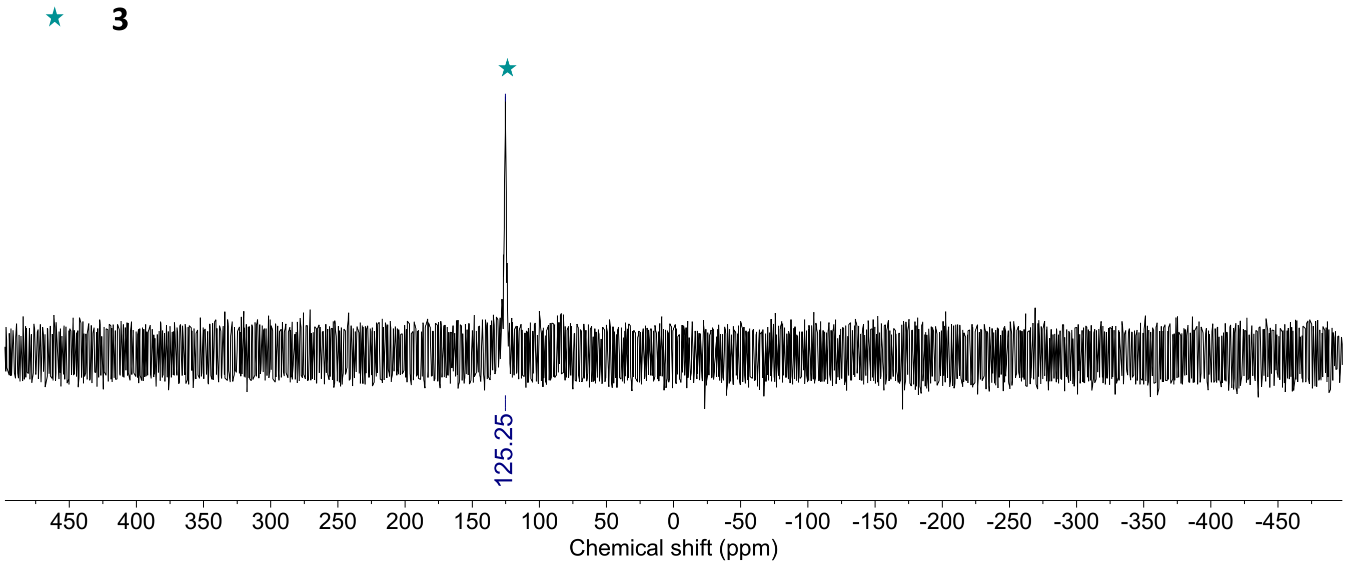

Figure S60: $^{31}P\{^{1}H\}$ NMR spectrum (162 MHz, d_8-toluene, 233 K) of crystals of **3**.

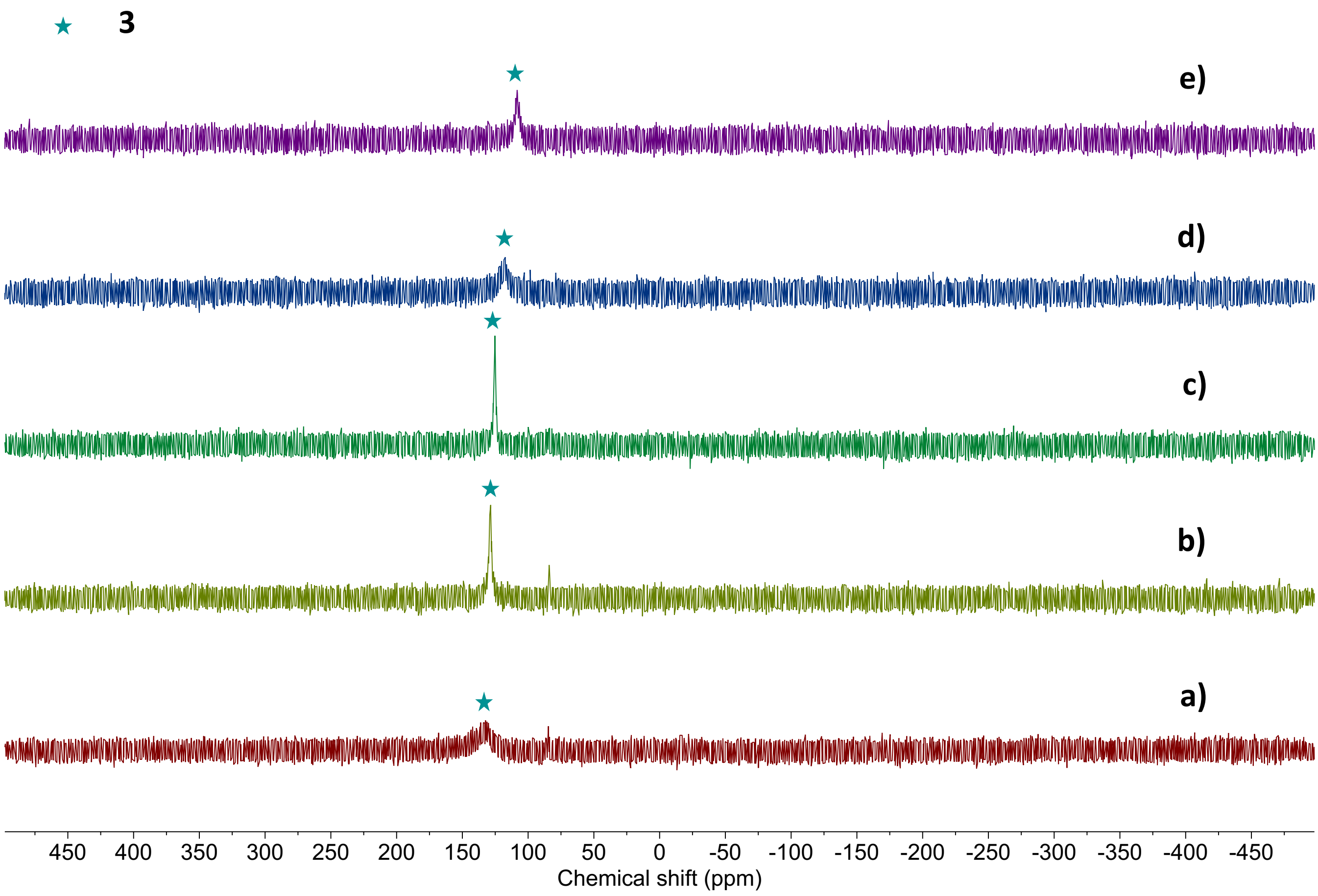

Figure S61: Variable temperature $^{31}P\{^{1}H\}$ NMR spectra (162 MHz, d_8-toluene) of crystals of **3** a) 203 K, b) 218 K, c) 233 K, d) 248 K, and e) 263 K.

X-ray crystallography

Suitable crystals were selected and mounted on various Rigaku diffractometers (XtaLAB Synergy R, DW system, HyPix-Arc 150 detector or SuperNova, Dual, Cu at home/near, AtlasS type detectors). The crystals were kept at a steady temperature during data collection. Data were measured using ω scans with Cu K_α radiation. The diffraction patterns were indexed and the total number of runs and images were based on the strategy calculation from the program CrysAlisPro 1.171.43.107a (Rigaku OD, 2024).[10] The unit cells were refined using CrysAlisPro 1.171.43.107a (Rigaku OD, 2024).[10] Data reduction, scaling and absorption corrections were performed using CrysAlisPro 1.171.43.107a (Rigaku OD, 2024).[10]

The structures were solved with the **ShelXT** (Sheldrick, 2015)[11] solution program using dual methods and by using **Olex2** 1.5 (Dolomanov et al., 2009)[12] as the graphical interface. The models were refined with **ShelXL** 2019/3 (Sheldrick, 2015)[13] using full matrix least squares minimisation on $\boldsymbol{F^2}$. All non-hydrogen atoms were refined anisotropically. Hydrogen atom positions were calculated geometrically and refined using the riding model.

Several issues were encountered during the stages of the refinement of the 9 crystal structures presented in the main text. In case of compound **1-U**, disorder affected partially 1 ligand. The used restraints (SADI, SIMU) were employed to get acceptable geometric and anisotropic parameters. The data were also treated for twinning (BASF parameter = 0.2833(9)). The crystal structure of compound **2** shows some disorder dealing with the ligand. Some restraints were used (SADI, SIMU, ISOR) to obtain reasonable distances and ellipsoids. The data collection of compound **1-Ce** was treated for twinning (BASF parameter = 0.319(1)). The crystal structure of **1-Sm** needed a wide range of restraints (RIGU, DFIX, SADI, ISOR, DELU, SIMU) to get acceptable behavior for 1 Ligand. In the case of compound **1*-Yb**, several restraints (SIMU, SADI, ISOR) were used to treat the disorder of the ligand and of the solvent.

Compound	**1-U**	**2**	**1-Ce**	**1-Tm**
Formula	$C_{38}H_{102}FeN_5P_4Si_6U$	$C_{62}H_{111}FeN_2O_3P_4U$	$C_{38}H_{102}CeFeN_5P_4Si_6$	$C_{38}H_{102}TmFeN_5P_4Si_6$
Crystal size [mm^3]	0.35×0.09×0.07	0.21×0.09×0.08	0.52×0.11×0.10	0.28×0.24×0.19
Crystal system	triclinic	orthorhombic	triclinic	monoclinic
Space group	P-1	$P2_12_12_1$	P-1	$P2_1/n$
V [Å^3]	5961.05(17)	6680.19(11)	5973.4(2)	5865.83(14)
a [Å]	11.55749(16)	16.81721(16)	11.56977(16)	11.60150(16)
b [Å]	19.2532(3)	19.7326(2)	19.2708(5)	18.3958(2)
c [Å]	27.3197(5)	20.13034(17)	27.3324(7)	27.5562(4)
α [°]	78.7323(15)	90	78.622(2)	90
β [°]	89.4820(13)	90	89.5036(15)	94.1162(12)
γ [°]	89.0470(12)	90	89.1024(16)	90
Z	4	4	4	4
Absorption coefficient [mm^{-1}]	11.923	9.726	1.243	7.159
F (000)	2508.0	2796.0	2372.0	2416.0
T [K]	140.00(10)	140.00(10)	140.00(10)	139.99(10)
Total no. reflexions	43530	41209	28872	62660
Unique reflexions [R(int)]	23035 [0.0377]	13920 [0.0331]	28872 [-]	12041
Final R indice [I>2σ(I)]	0.0306	0.0263	0.0645	0.0421
Largest diff. peak and hole [eA^{-3}]	1.116 and -1.177	0.981 and -0.674	2.604 and -1.990	1.581 and -1.135
GooF	1.008	1.091	1.040	1.046
CCDC number	2330344	2330342	2330341	2330340

Table S1. X-ray crystallographic data for **1-U, 2, 1-Ce,** and **1-Tm**.

Compound	**1-Sm**	**1*-Yb**$(thf)_{0.4}(Et_2O)_{0.6}$	**1-Dy**
Formula	$C_{38}H_{102}FeN_5P_4Si_6Sm$	$C_{36}H_{93.2}FeN_4OP_4Si_4Yb$	$C_{38}H_{102}DyFeN_5P_4Si_6$
Crystal size [mm^3]	0.31×0.16×0.13	0.52×0.29×0.23	0.26×0.13×0.12
Crystal system	monoclinic	monoclinic	monoclinic
Space group	$P2_1/n$	$P2_1/n$	$P2_1/n$
V [Å^3]	5930.7(4)	5514.23(12)	5915.5(5)
a [Å]	11.8173(5)	10.35562(15)	11.6319(5)
b [Å]	18.0593(7)	36.8715(3)	18.3899(9)
c [Å]	27.8343(12)	14.64313(18)	27.7329(15)
α [°]	90	90	90
β [°]	93.235(4)	99.5143(13)	94.323(4)
γ [°]	90	90	90
Z	4	4	4
Absorption coefficient [mm^{-1}]	11.715	7.304	11.048
F (000)	2388.0	2228.8	2404.0
T [K]	140.00(10)	200.07(16)	139.99(10)
Total no. reflexions	94924	65865	47358
Unique reflexions [R(int)]	11946 [0.0752]	11541 [0.0351]	11642 [0.1327]
Final R indice [I>2σ(I)]	0.0659	0.0314	0.0603
Largest diff. peak and hole [eA^{-3}]	2.028 and -1.098	0.956 and -0.592	1.734 and -0.725
GooF	1.081	1.066	0.867
CCDC number	2330343	2331570	2330345

Table S2. X-ray crystallographic data for **1-Sm**, **1-Dy** and **1*-Yb**.

Compound	**1*-Sm**	**3**
Formula	$C_{32}H_{84}FeN_4P_4Si_4Sm$	$C_{55}H_{139}Fe_2N_6P_8Si_4Sm$
Crystal size [mm^3]	0.31×0.22×0.18	0.21×0.07×0.06
Crystal system	orthorhombic	monoclinic
Space group	*Pbca*	$P2_1/c$
V [$Å^3$]	10243.3(4)	7905.9(5)
a [Å]	20.9865(4)	11.4532(5)
b [Å]	19.1066(5)	29.5233(9)
c [Å]	25.5457(5)	23.5755(8)
α [°]	90	90
β [°]	90	97.369(4)
γ [°]	90	90
Z	8	4
Absorption coefficient [mm^{-1}]	13.044	10.776
F (000)	4064.0	3204.0
T [K]	200.15	140.00(10)
Total no. reflexions	34072	37561
Unique reflexions [R(int)]	9892 [0.0445]	15331 [0.1009]
Final R indice [I>2σ(I)]	0.0378	0.0585
Largest diff. peak and hole [eA^{-3}]	0.960 and -0.336	0.992 and -0.594
GooF	1.020	0.976
CCDC number	2331569	2330339

Table S3. X-ray crystallographic data for **1*-Sm** and **3**.

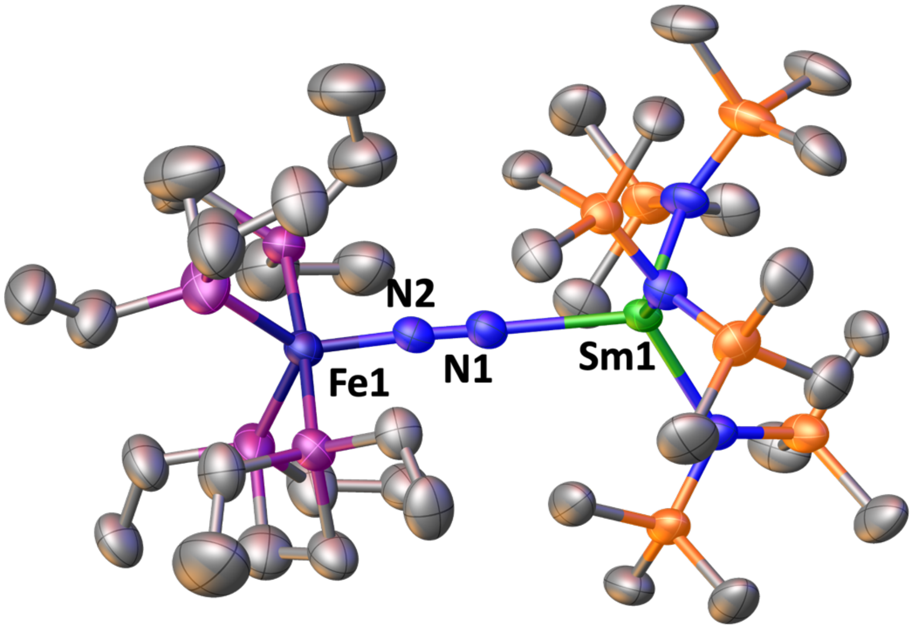

Figure S62: Solid-state molecular structure of **1-Sm** with 50% probability ellipsoids. Color code: samarium (light green), phosphorus (purple), iron (midnight blue), carbon (grey), silicon (orange). Hydrogen atoms were omitted for clarity.

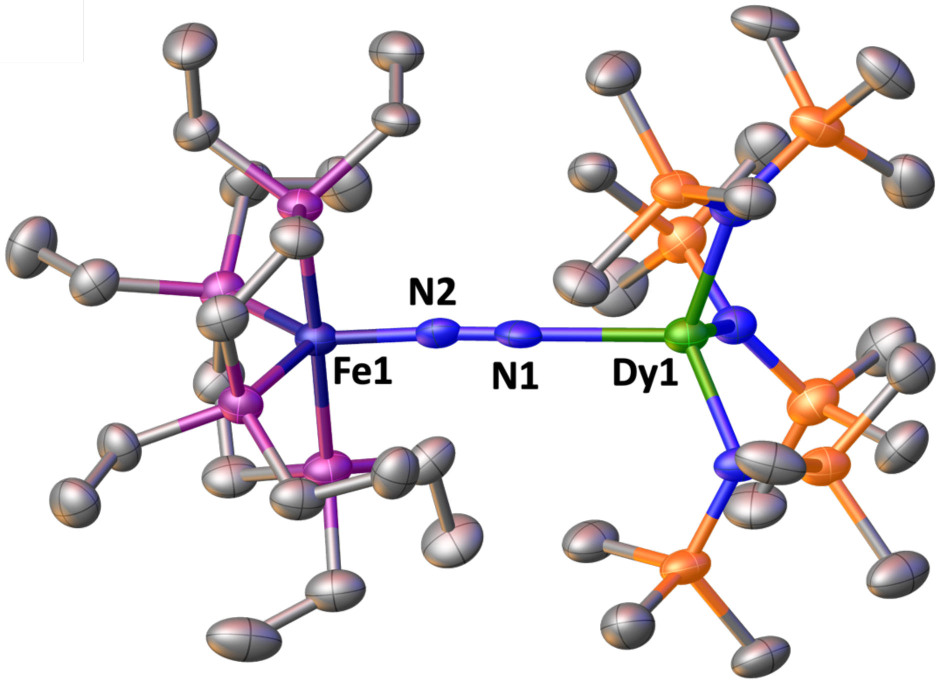

Figure S63: Solid-state molecular structure of **1-Dy** with 50% probability ellipsoids. Color code: dysprosium (light green), phosphorus (purple), iron (midnight blue), carbon (grey), silicon (orange). Hydrogen atoms were omitted for clarity.

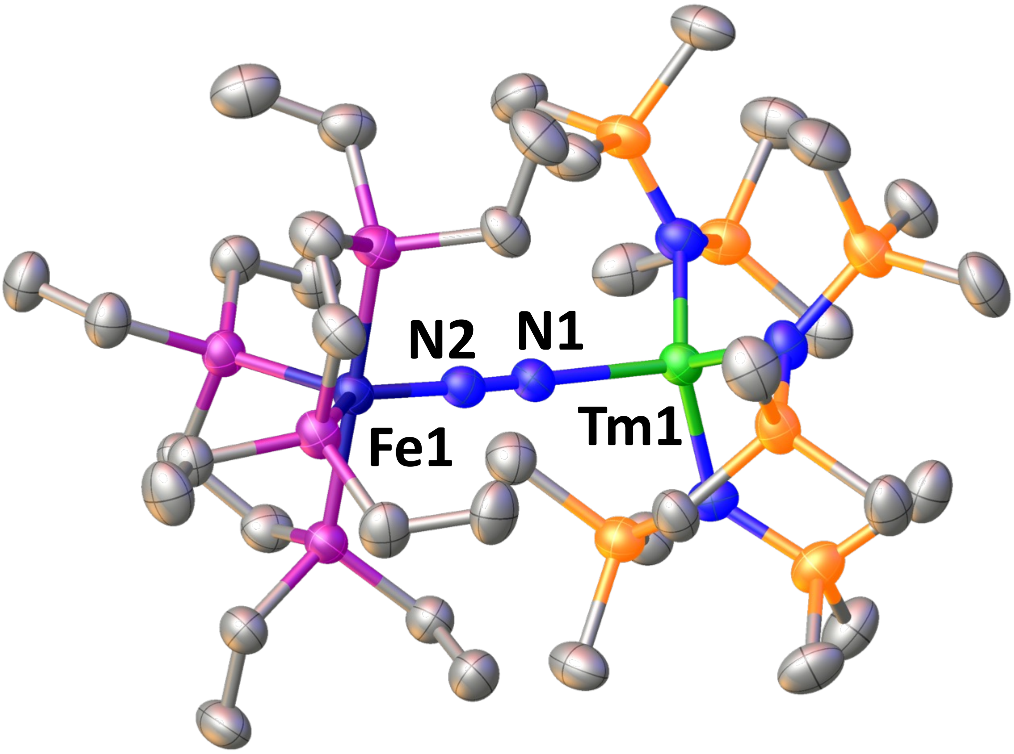

Figure S64: Solid-state molecular structure of **1-Tm** with 50% probability ellipsoids. Color code: thulium (light green), phosphorus (purple), iron (midnight blue), carbon (grey), silicon (orange). Hydrogen atoms were omitted for clarity.

EPR data

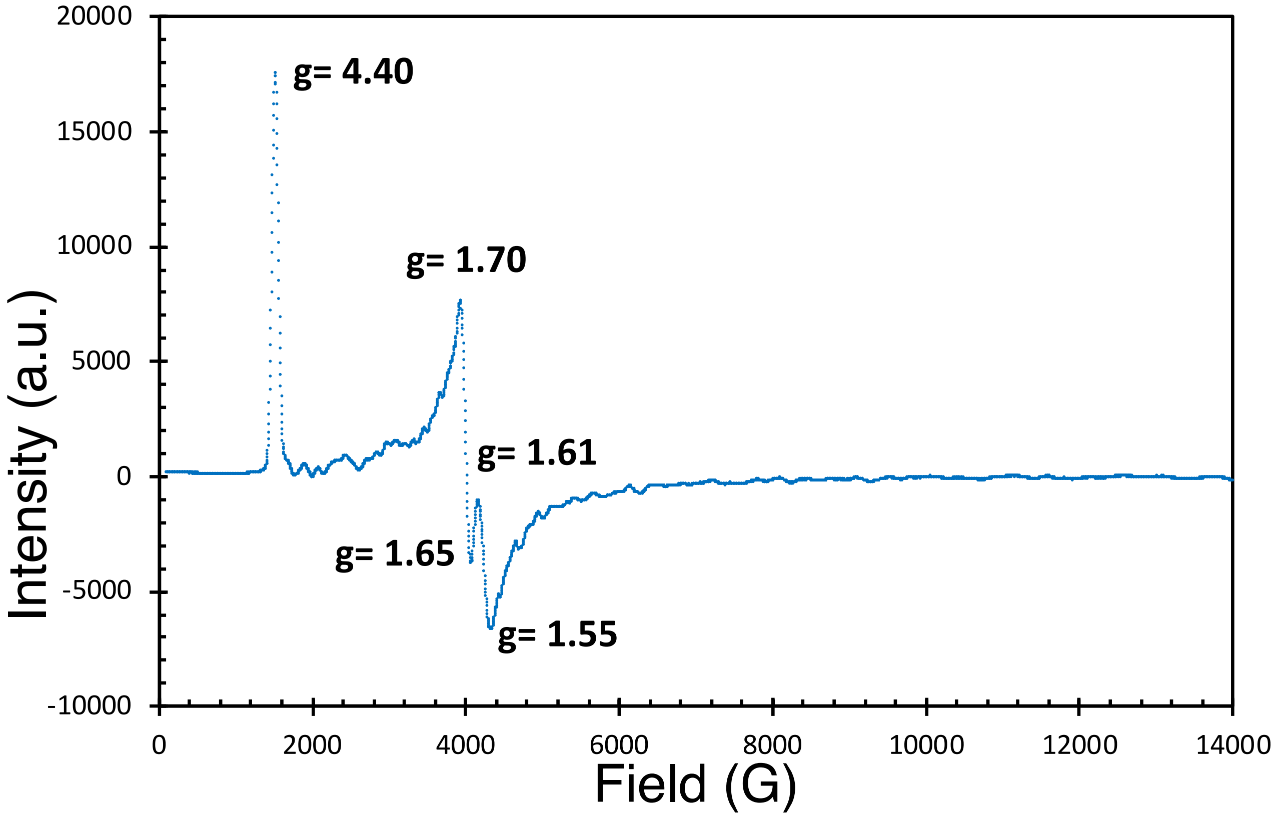

Figure S65: X-band EPR spectrum of pure powder of **1-U** at 6 K. (ν = 9.397400 GHz, P = 0.6332 mW, amplitude modulation 5 G, frequency modulation 100 kHz).

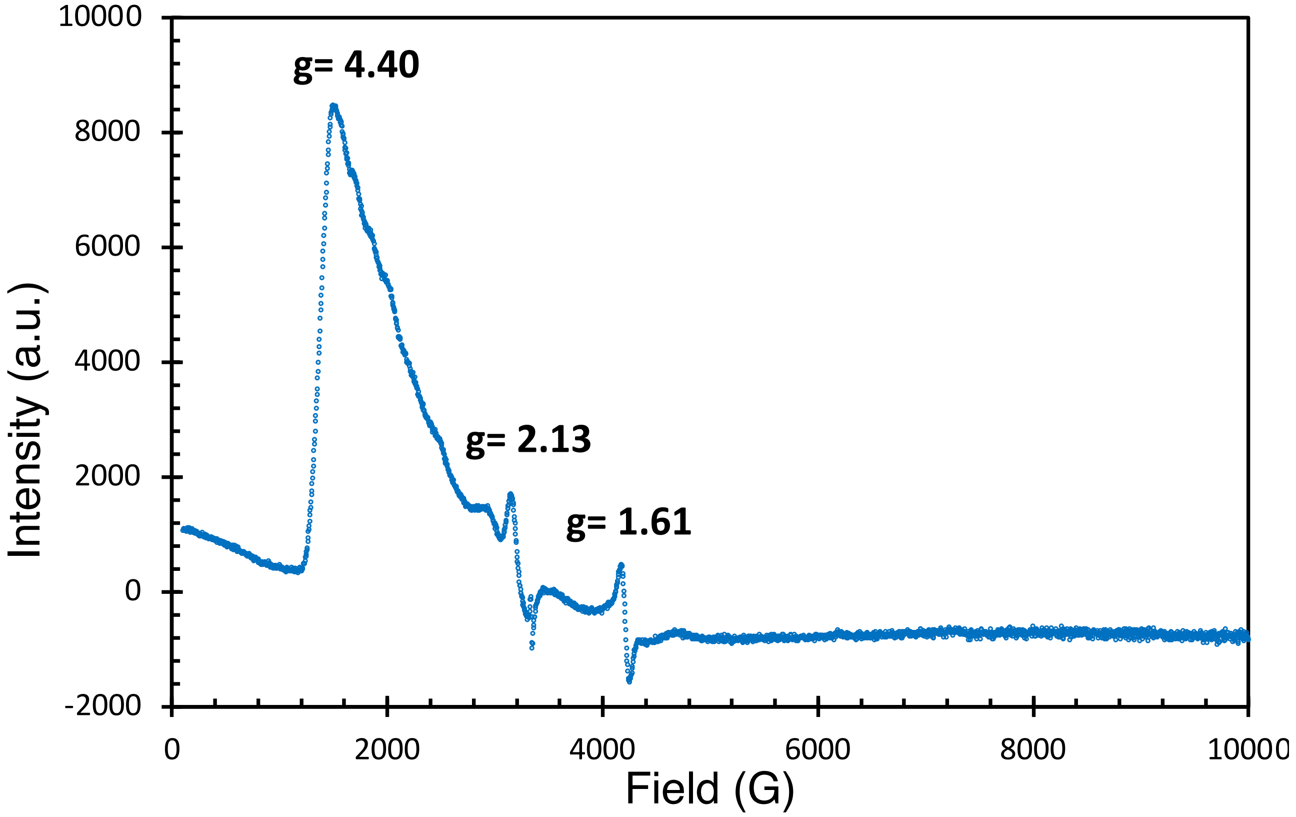

Figure S66: X-band EPR spectrum of a 20 mM frozen solution of **1-U** in a Hex:Tol (1:1) glass at 6 K. (ν = 9.397400 GHz, P = 0.6332 mW, amplitude modulation 5 G, frequency modulation 100 kHz).

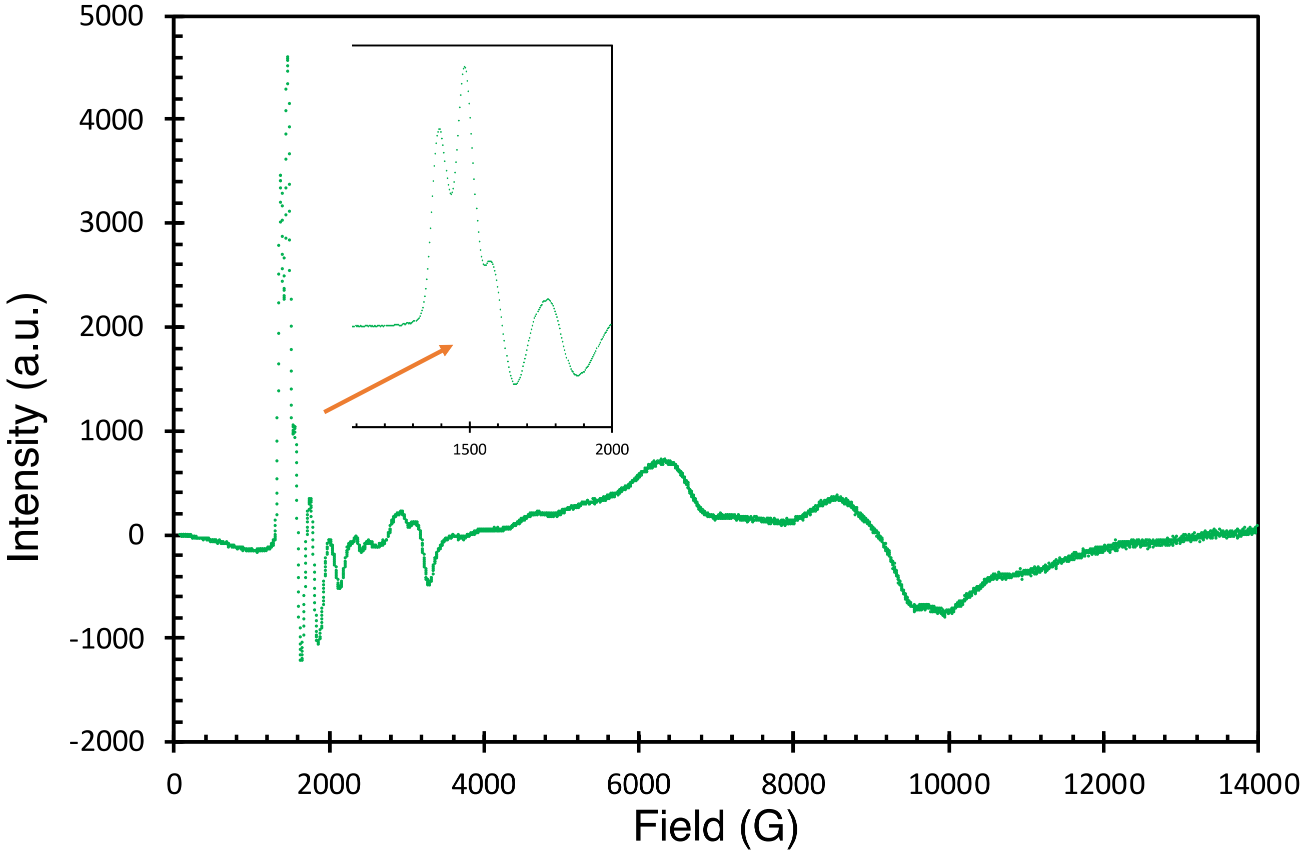

Figure S67: X-band EPR spectrum of pure powder of **2** at 6 K. (ν = 9.397400 GHz, P = 0.6332 mW, amplitude modulation 5 G, frequency modulation 100 kHz).

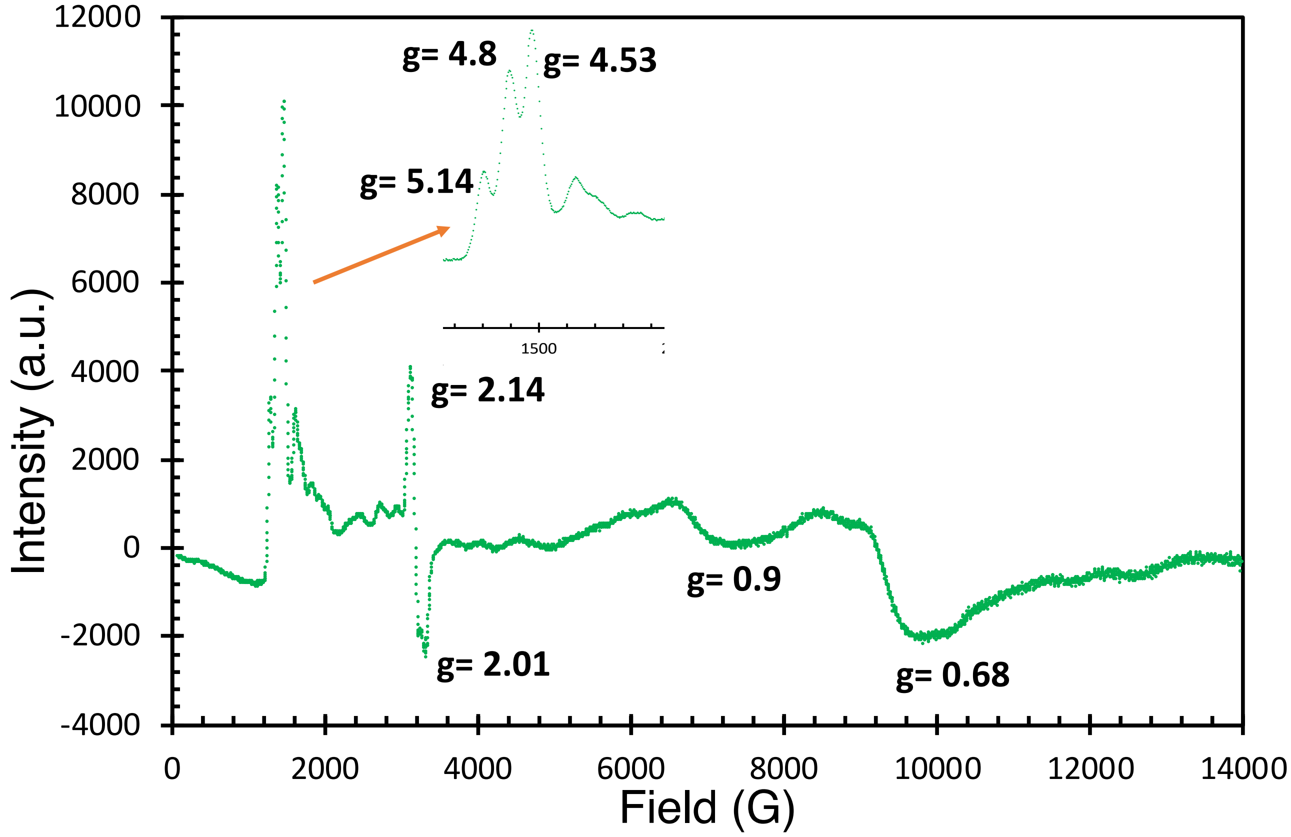

Figure S68: X-band EPR spectrum of a 20 mM frozen solution of **2** in a Hex:Tol (1:1) glass at 6 K. (v = 9.397400 GHz, P = 0.6332 mW, amplitude modulation 5 G, frequency modulation 100 kHz).

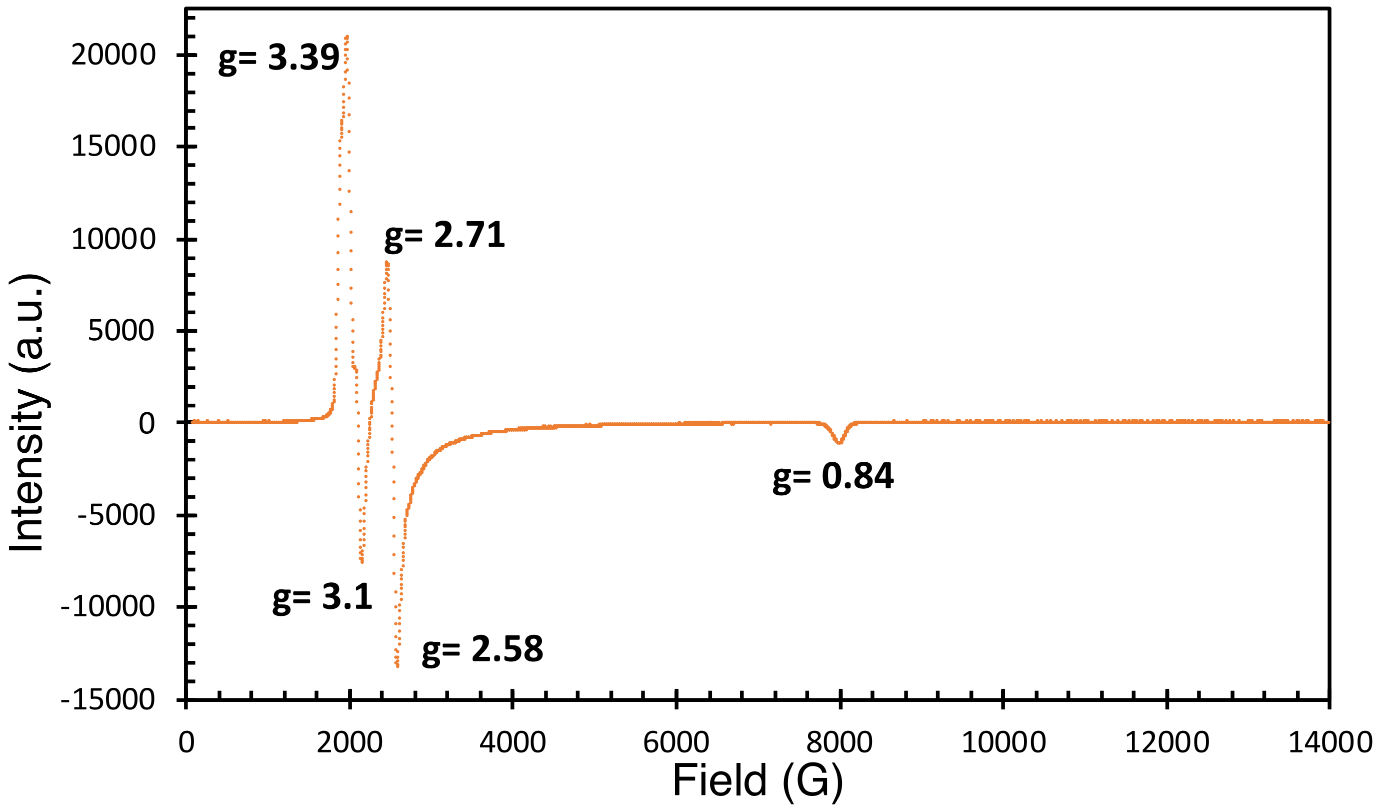

Figure S69: X-band EPR spectrum of pure powder of **[U(ODtbp)$_3$]** at 6 K. (ν = 9.397400 GHz, P = 0.006332 mW, amplitude modulation 5 G, frequency modulation 100 kHz).

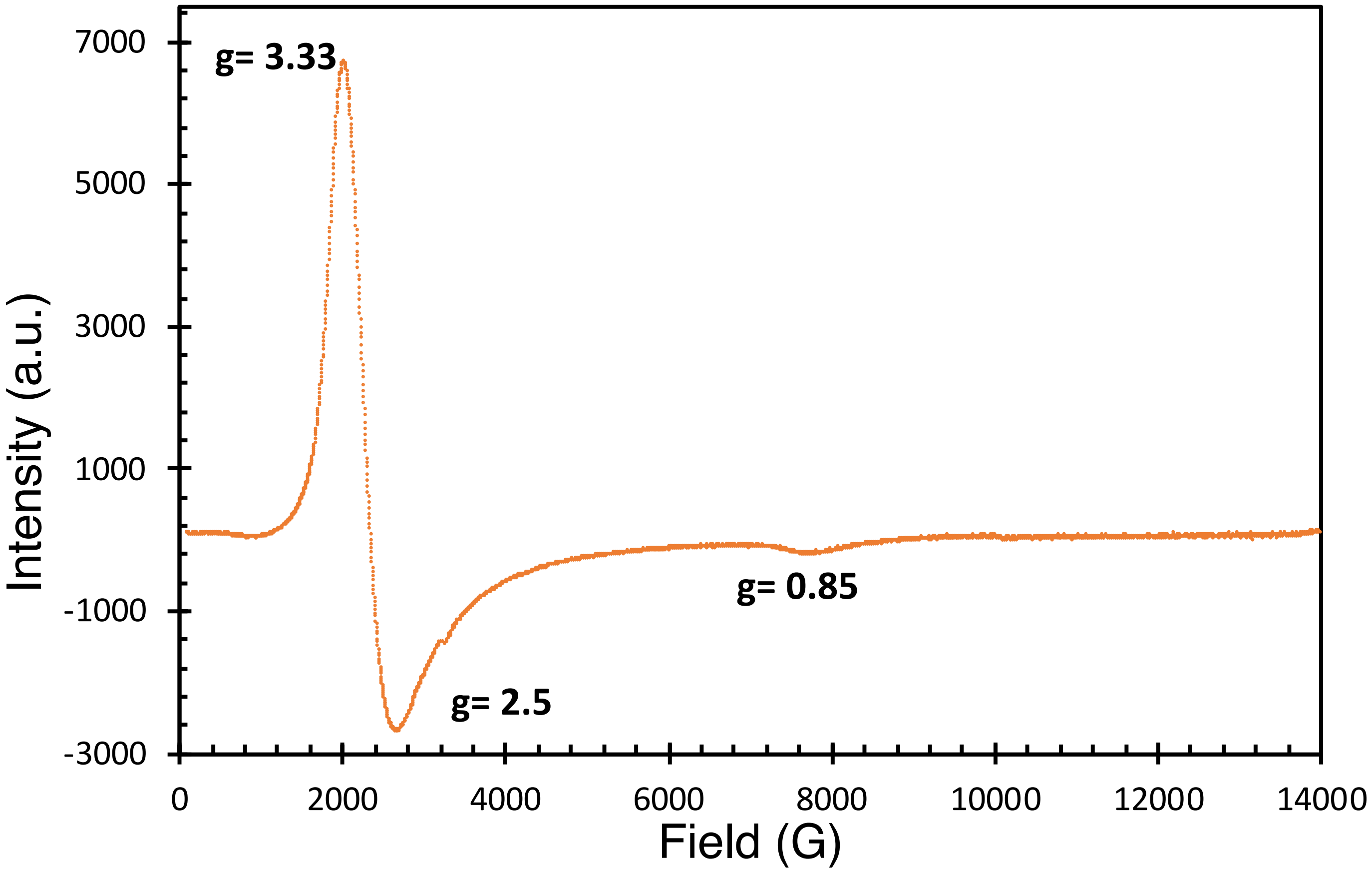

Figure S70: X-band EPR spectrum of a 20 mM frozen solution of **[U(ODtbp)$_3$]** in a Hex:Tol (1:1) glass at 6 K. (ν = 9.397400 GHz, P = 0.006332 mW, amplitude modulation 5 G, frequency modulation 100 kHz).

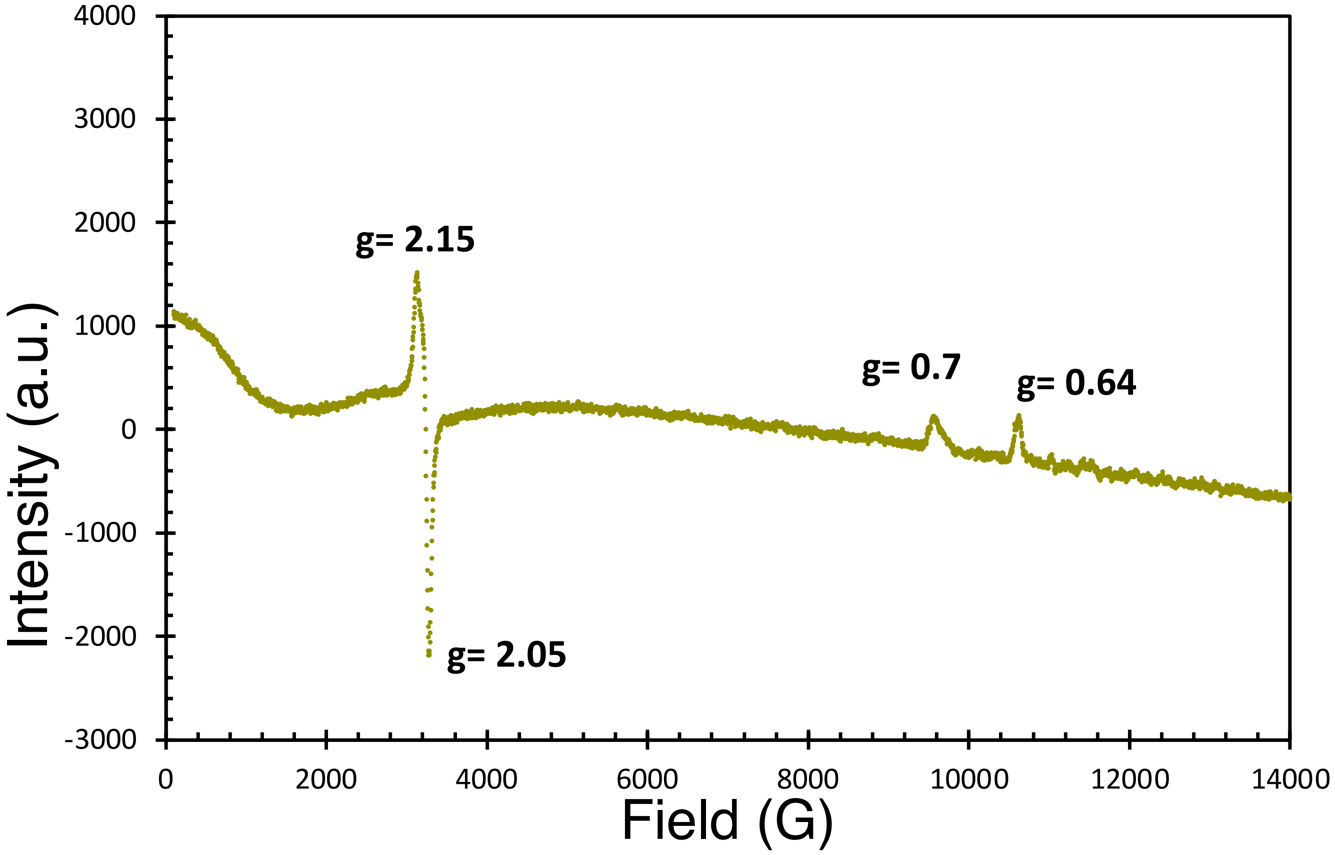

Figure S71: X-band EPR spectrum of pure powder of **1-Sm** in a Hex:Tol (1:1) glass at 6 K. (v = 9.4029 GHz, P = 0.000633307 mW, amplitude modulation 5 G, frequency modulation 100 kHz).

The X-band EPR spectrum of pure powder of **1*-Sm** does not show any signal as expected for the f^6 ion.

IR data

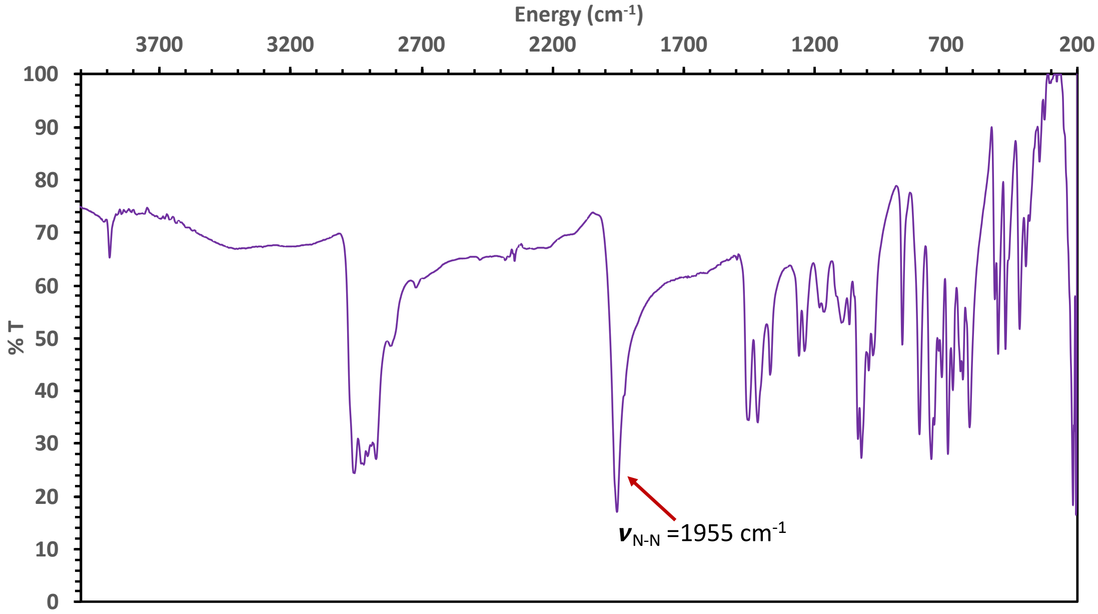

Figure S72: IR spectrum of isolated complex **A** in KBr pill measured at 25 °C.

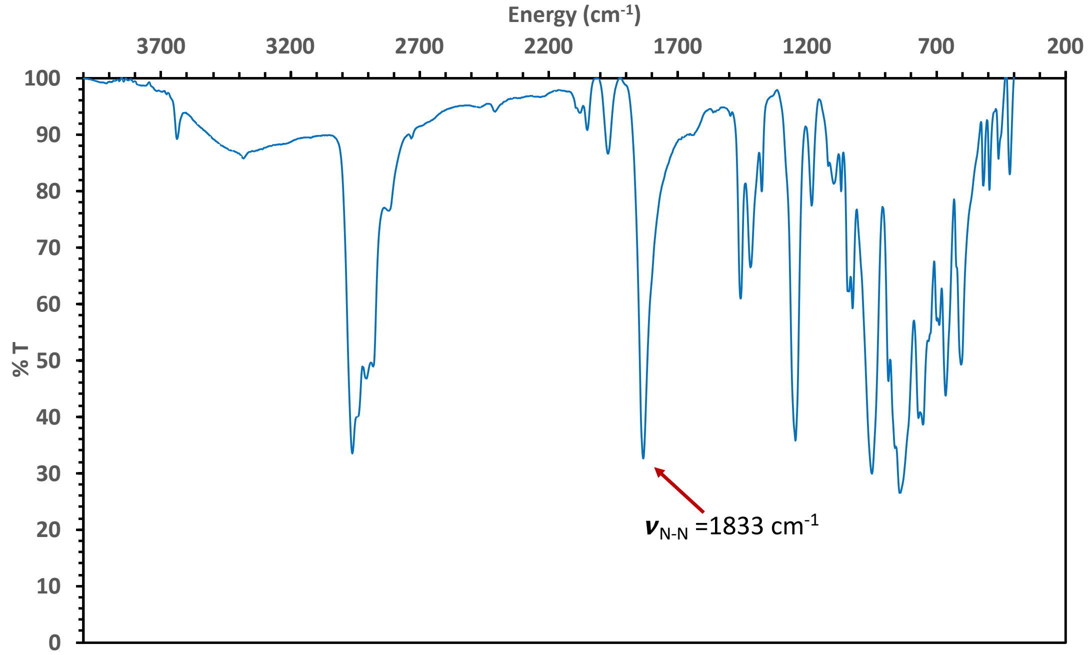

Figure S73: IR spectrum of isolated complex **1-U** in KBr pill measured at 25 °C.

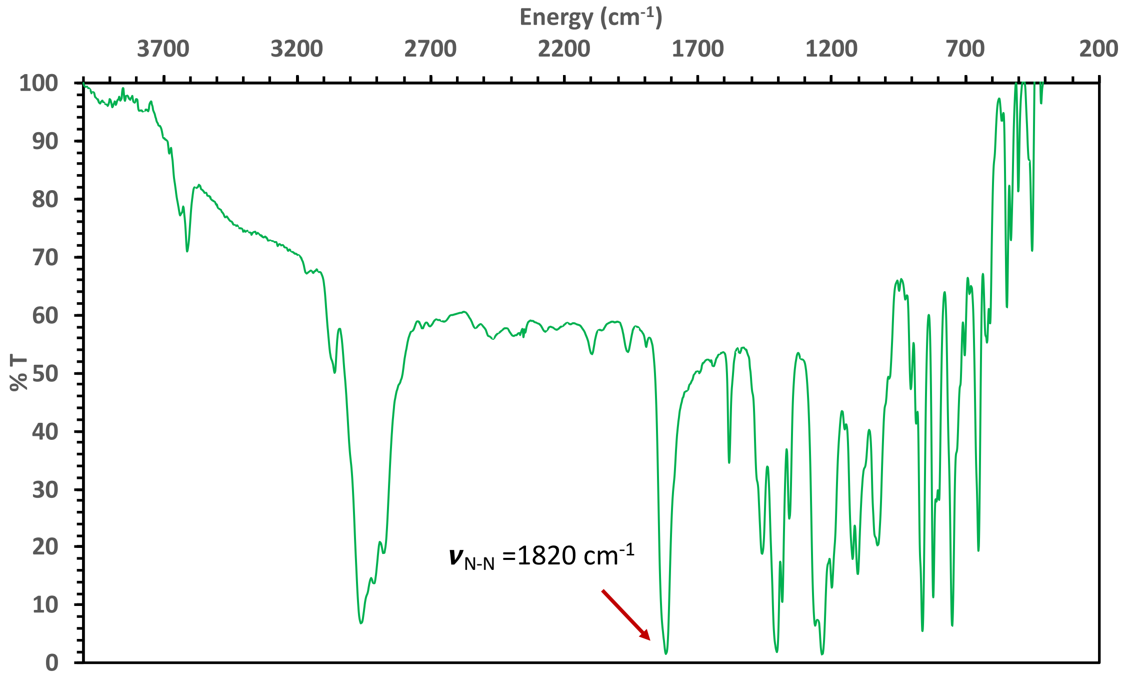

Figure S74: IR spectrum of isolated complex **2** in KBr pill measured at 25 °C.

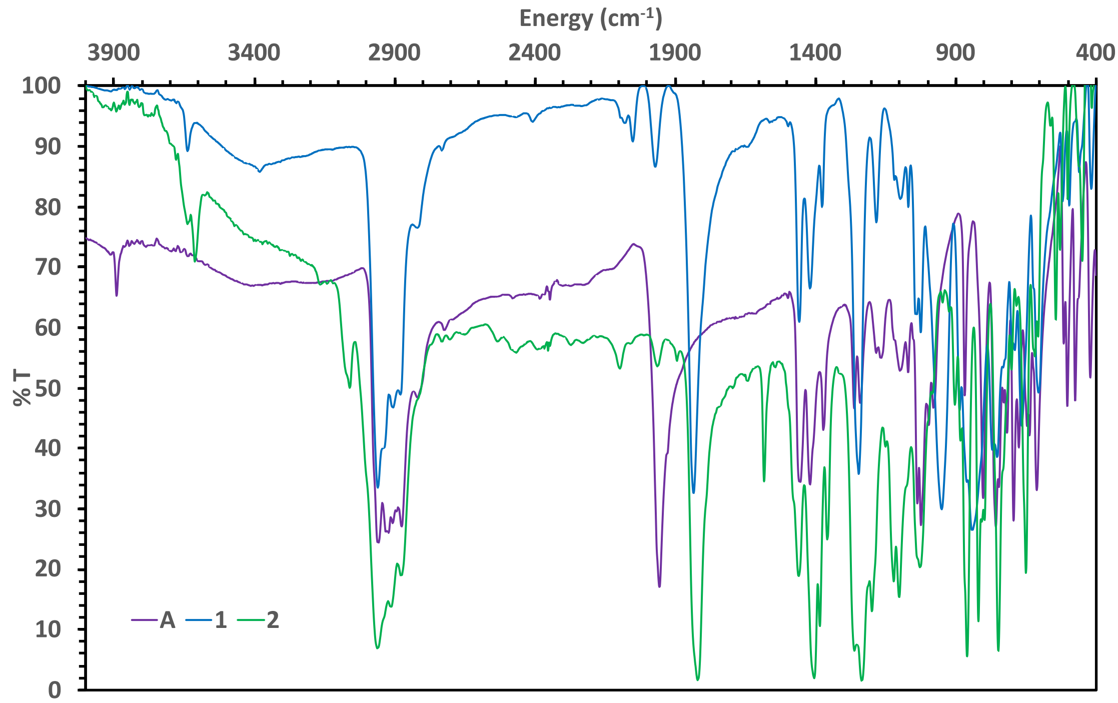

Figure S75: IR spectra comparison of isolated complexes **A** (purple), **1-U** (blue) and **2** (green) in KBr pill measured at 25 °C.

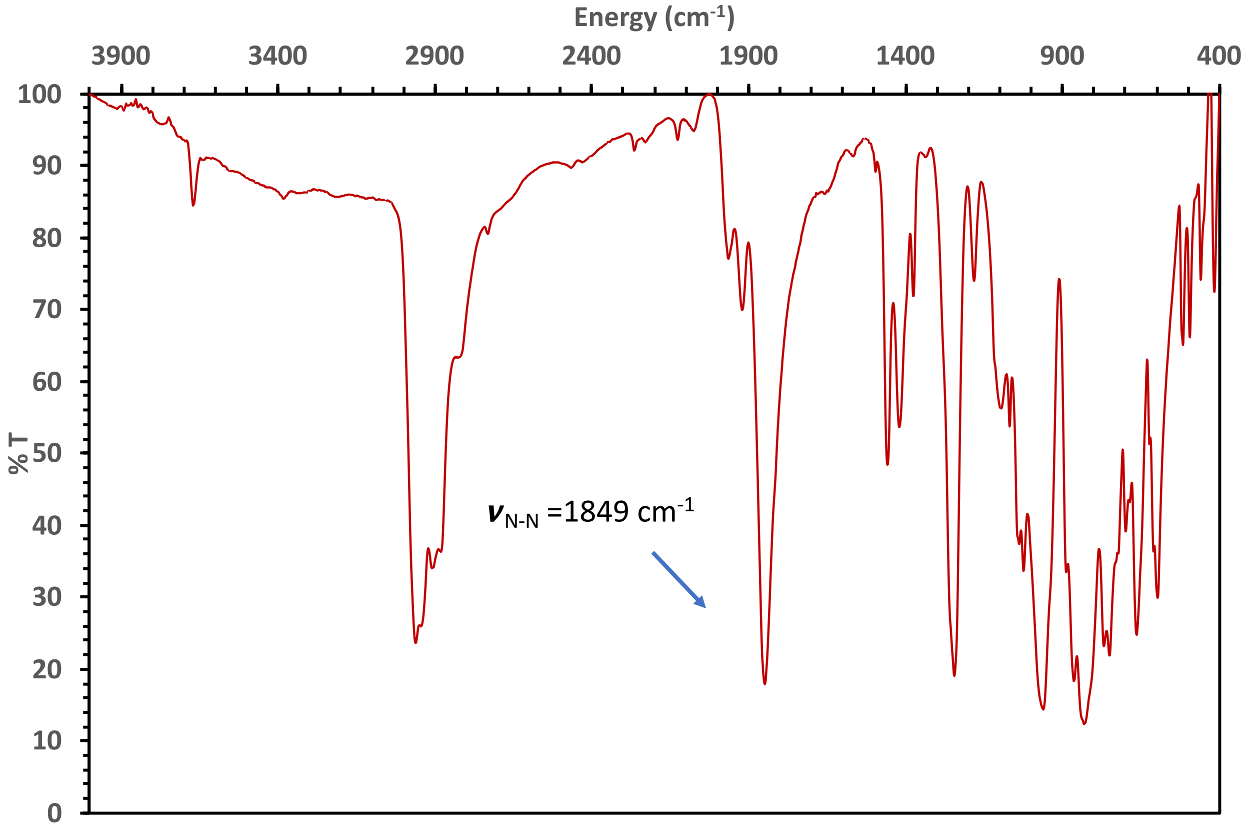

Figure S76: IR spectrum of isolated complex **1-Ce** in KBr pill measured at 25 °C.

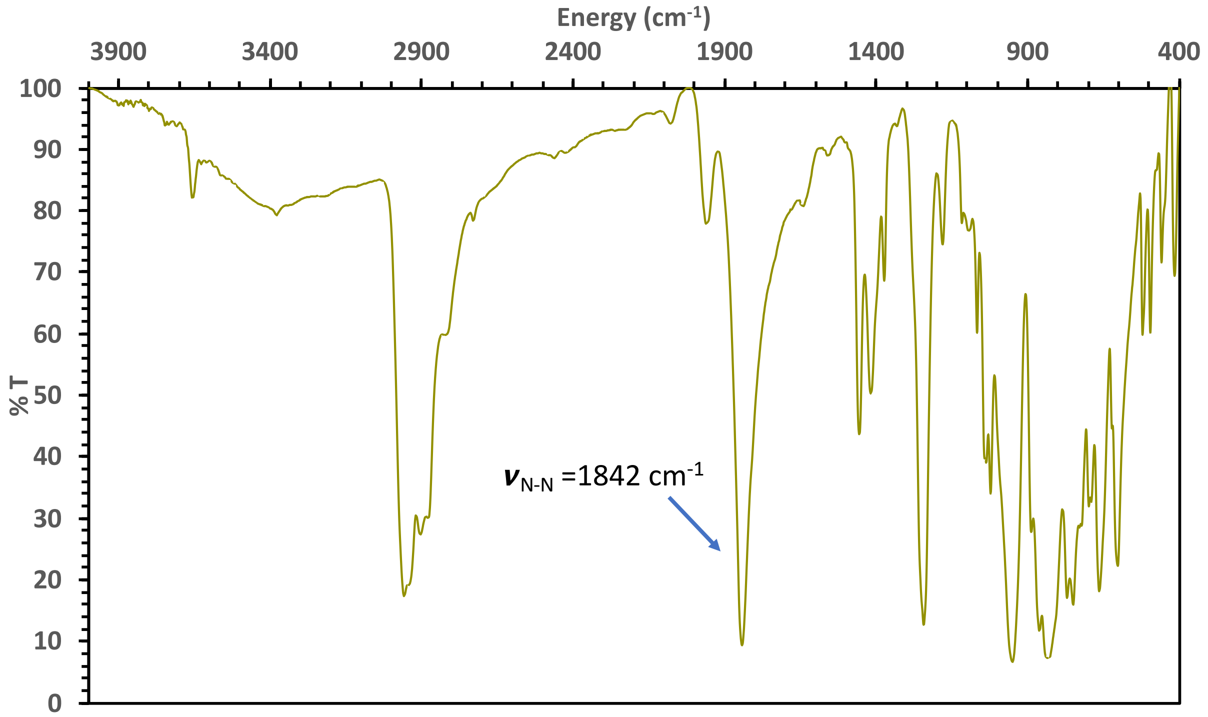

Figure S77: IR spectrum of isolated complex **1-Sm** in KBr pill measured at 25 °C.

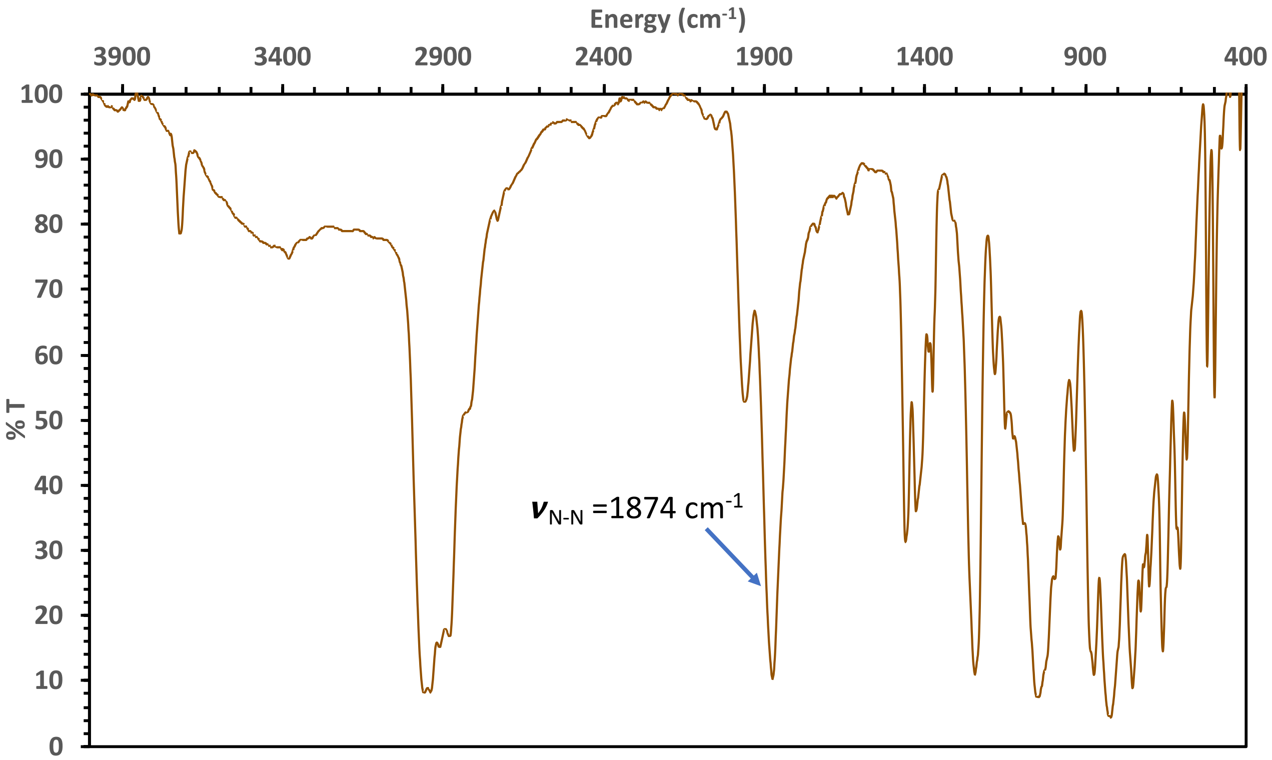

Figure S78: IR spectrum of isolated complex **1*-Yb** in KBr pill measured at 25 °C.

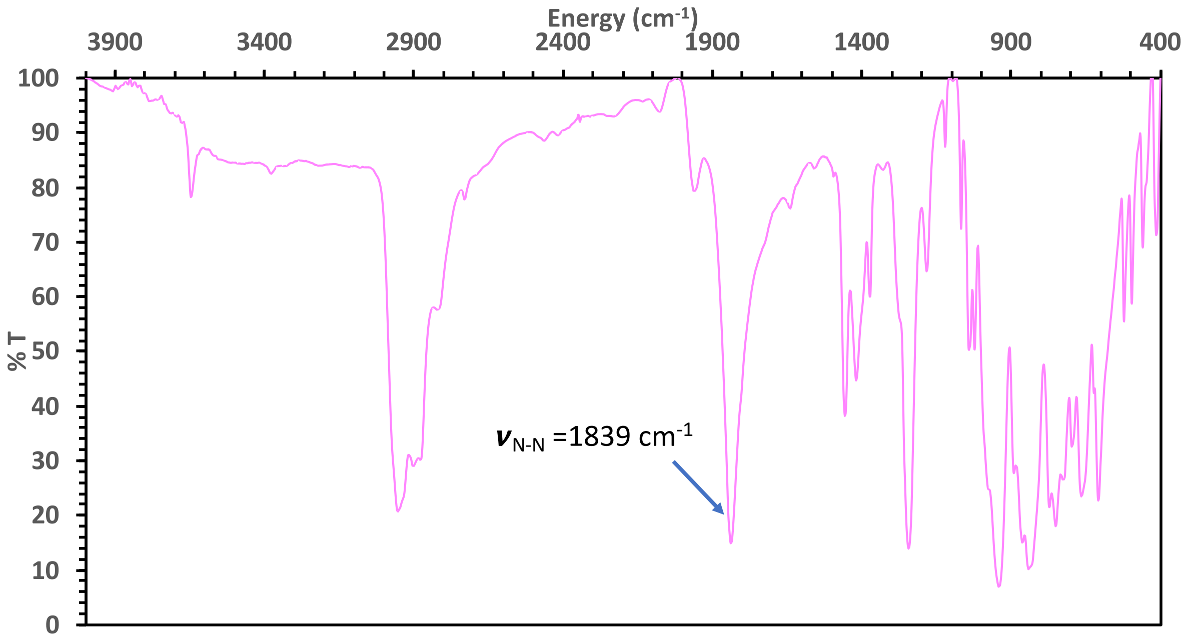

Figure S79: IR spectrum of isolated complex **1-Dy** in KBr pill measured at 25 °C.

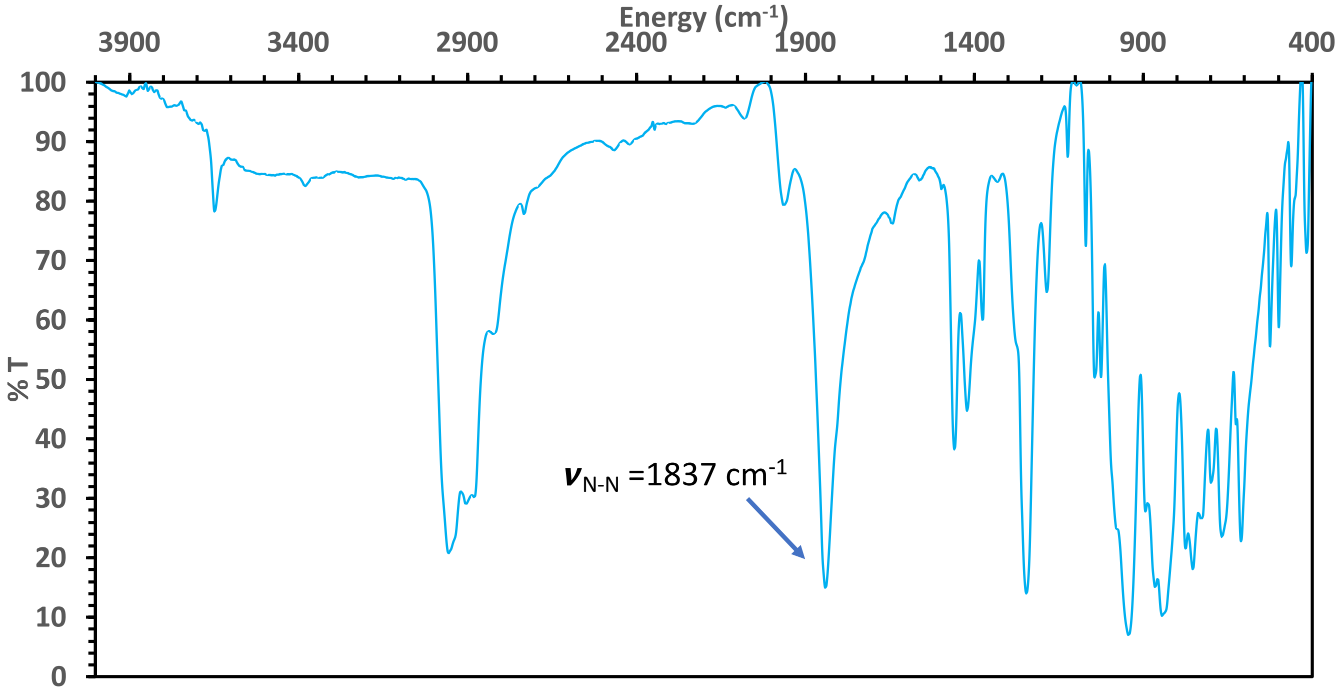

Figure S80: IR spectrum of isolated complex **1-Tm** in KBr pill measured at 25 °C.

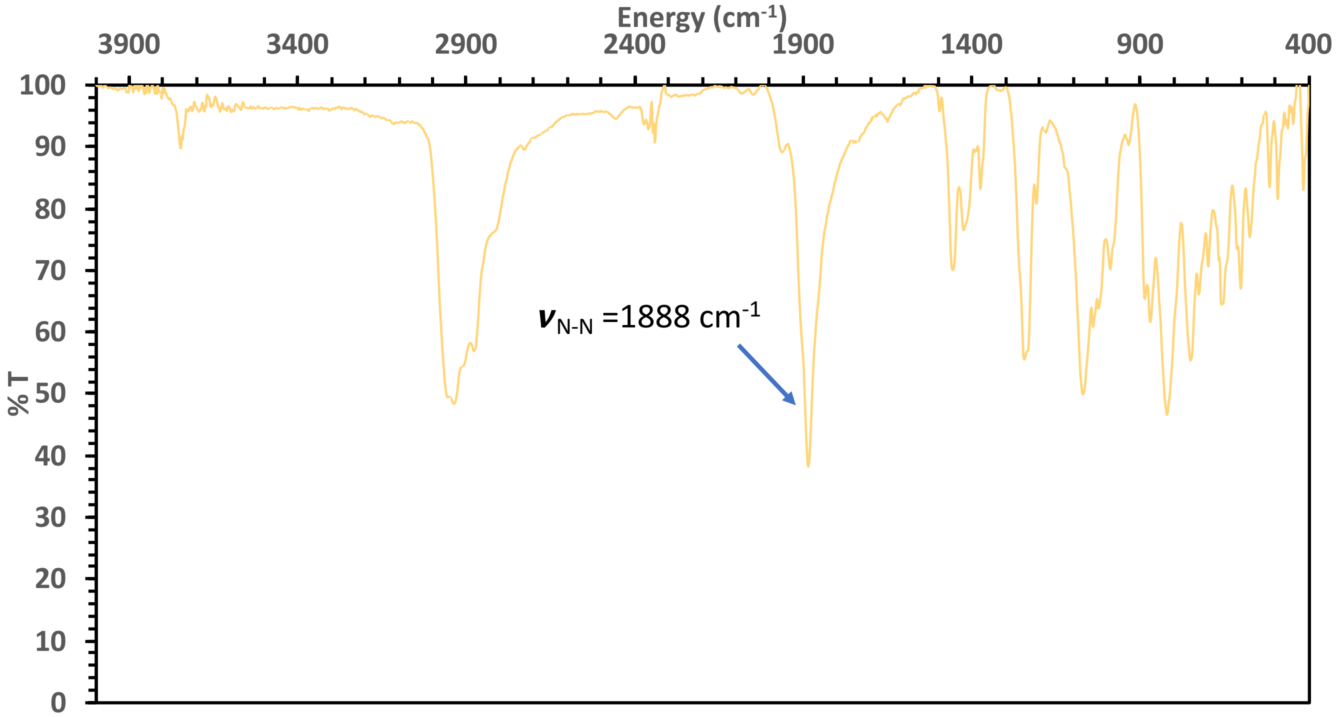

Figure S81: IR spectrum of isolated complex **1*-Sm** in KBr pill measured at 25 °C.

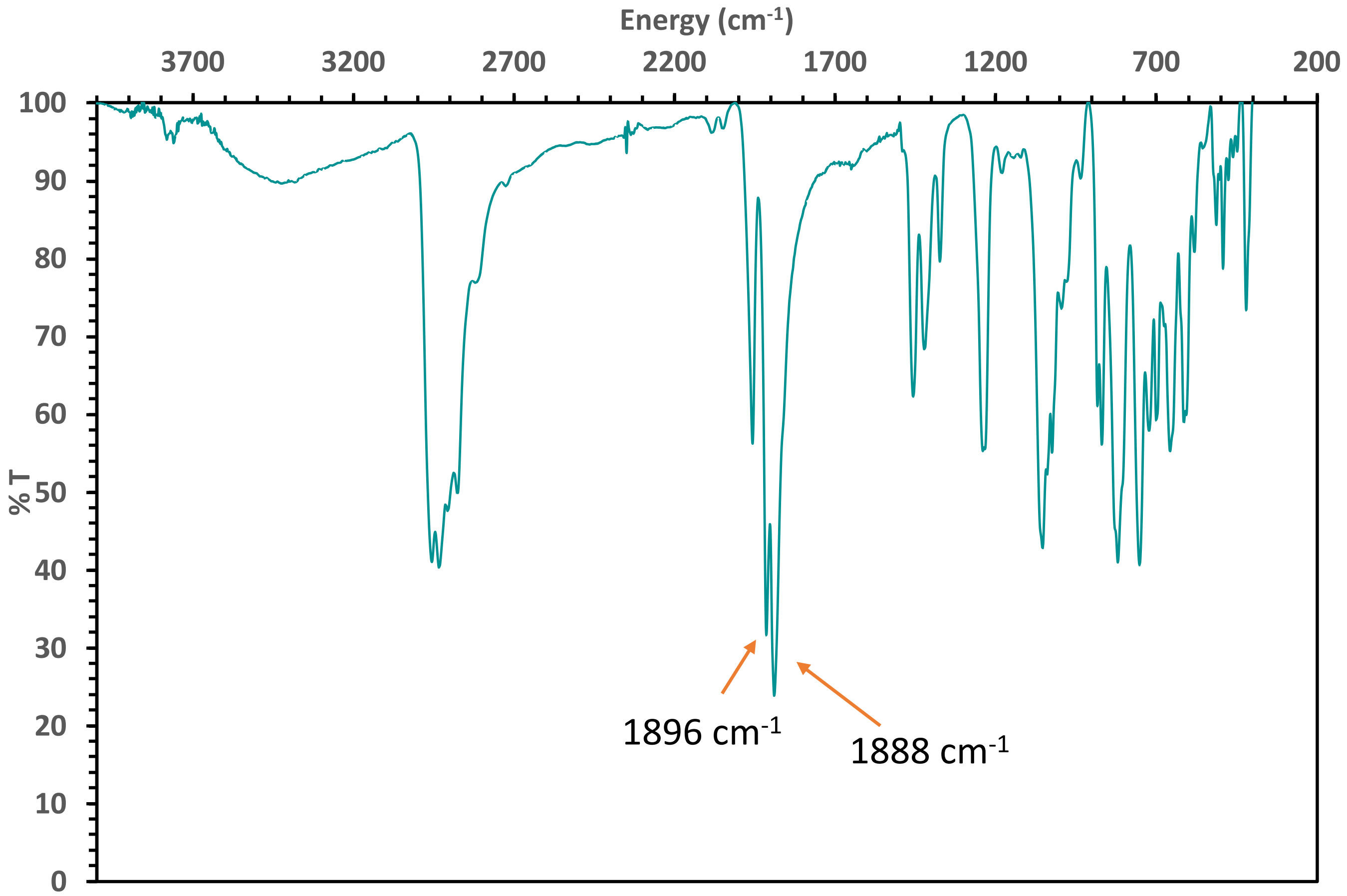

Figure S82: IR spectrum of isolated complex **3** in KBr pill measured at 25 °C.

Computational Details

The optimization of heterometallic complexes were carried out by employing DFT hybrid functional (B3PW91)[14] along with small core pseudopotential Stuggart basis set for uranium, cerium (small core or large core), samarium (small core or large core), dysprosium (small core or large core), ytterbium, iron, phosphorus and silicon atoms with additional polarization functions for phosphorus and silicon atoms.[15] Pople basis sets (6-31G**) were employed for the rest of the atoms.[16] Frequency calculations were performed to locate minima for the optimized structures. Dispersion corrections were included in our calculations by employing D3 version of Grimme's dispersion with Becke-Johnson damping.[17] To account for the solvation effects, SMD model using toluene solvent has been included in the calculations. [18] All the calculations were performed using Gaussian 09 suite of programs.[19]

Table ST1: DFT computed spin energetics for **1-U**.

Spin states	Δ*H* (kcal/mol)
S=3/2	0.0
S=1/2	15.3

Figure S83: DFT computed spin density plot for **1-U**, **(S=3/2)**.

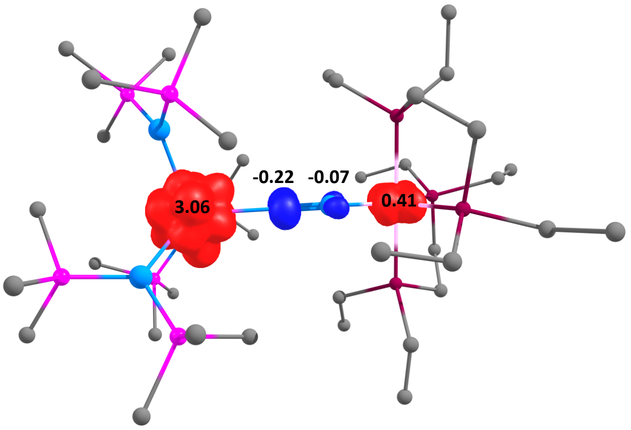

Table ST2: DFT computed spin energetics for **2**

Spin states	Δ*H* (kcal/mol)
S=3/2	0.0
S=1/2	15.6

Figure S84: DFT computed spin density plot for **2**, **(S=3/2)**.

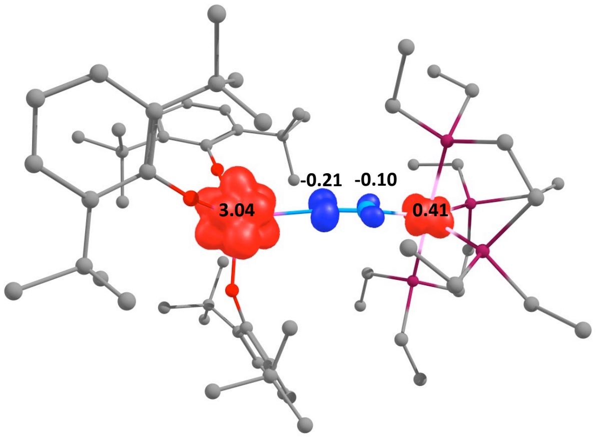

Table ST3. Comparison of selected structural parameters between DFT computed and x-ray structures.

		X-ray	DFT			
			Small core	Small core + solv+ disp	Large core	Large core + disp
**1-Ce** (S=1/2)	Fe-N	1.71	1.74	1.71	1.74	1.71
	N-N	1.11	1.16	1.16	1.16	1.16
	Ce-N	2.54	2.56	2.54	2.60	2.54
**1-Sm** (S=5/2)	Fe-N	1.76	1.78	1.73	1.74	1.71
	N-N	1.16	1.16	1.16	1.16	1.16
	Sm-N	2.48	2.50	2.44	2.53	2.48
**1*-Sm** (S=3)	Fe-N	1.75	1.74	1.72		
	N-N	1.13	1.15	1.15		
	Sm-N	2.57	2.61	2.58		
	Sm-N-N	174.0	176.8	144.0		
**1-Dy** (S=5/2)	Fe-N	1.75	1.75	1.71	1.73	1.71
	N-N	1.17	1.16	1.16	1.16	1.16
	Dy-N	2.40	2.42	2.38	2.43	2.40
**1*-Yb** (S=0)	Fe-N	1.76	1.74	1.72		
	N-N	1.15	1.15	1.16		
	Yb-N	2.44	2.51	2.39		
**3**	Fe-N	1.76, 1.76	1.75, 1.76	1.73, 1.73		
	N-N	1.14, 1.14	1.15, 1.15	1.15, 1.15		
	Sm-N	2.67, 2.57	2.73, 2.72	2.56, 2.55		
**1-U** (S=3/2)	Fe-N	1.73/1.74	1.74			
	N-N	1.16/1.14	1.16			
	U-N	2.48/2.52	2.53			
**2** (S=3/2)	Fe-N	1.75	1.75			
	N-N	1.16	1.17			
	U-N	2.47	2.52			
**A** (S=0)	Fe-N	1.75	1.77			
	N-N	1.14	1.14			
$N_2U(NSiMe_2)_3$ (S=3/2)	U-N		2.46			
	N-N		1.12			
$N_2U(OAr)_3$ (S=3/2)	U-N		2.50			
	N-N		1.12			

Table ST4. Computed dissociation energies (Δ*H*, kcal/mol) for X_n(Ln/U)-N_2-Fe(depe)$_2$ → X_n(Ln/U) + **A** reaction (X = HMDS or OAr; n = 2 or 3).

	**1-Ce**	**1-Sm**	**1-Dy**	**1*-Yb**	**1*-Sm**	**1-U**	**2**
Small core	-4.1	-4.9	-2.7	0.6	1.5	-0.9	-2.2
Small core (solv + disp)				11.5	8.0	32.1	
Large core	2.9	-2.0	-0.7				
Large core (disp)	34.0	30.8	30.7				

Table ST5. Computed dissociation energies (Δ*H*, kcal/mol) for the X_3U-N_2 → X_3U + N_2 reaction (X = HMDS or OAr).

	N_2U(HMDS)$_3$	N_2U(OAr)$_3$
Small core	-4.2	-0.5

Table ST6. Computed dissociation energies (Δ*H*, kcal/mol) for the **3** → **1*-Sm** + **A** reaction.

	BDE
Small core	1.1

Table ST7. Computed Wiberg bond index (small core, large core) for the FeN_2Ln/U core.

**1-Ce** (S=1/2)	Wiberg bond Index	Atom Label	Wiberg bond Index	Atom Label	Wiberg bond Index
Ce1	0.0000	Fe2	0.0000	N13	0.0000
N13	0.2212<br>0.1708	N14	0.7535<br>0.7612	N14	2.3305<br>2.2893
**1-Sm** (S=5/2)	Wiberg bond Index	Atom Label	Wiberg bond Index	Atom Label	Wiberg bond Index
Sm1	0.0000	Fe2	0.0000	N13	0.0000
N13	0.2378<br>0.1840	N14	0.7526<br>0.7636	N14	2.3234<br>2.2884
**1-Dy** (S=5/2)	Wiberg bond Index	Atom Label	Wiberg bond Index	Atom Label	Wiberg bond Index
Dy1	0.0000	Fe2	0.0000	N13	0.0000
N13	0.2721<br>0.1920	N14	0.7679<br>0.7763	N14	2.2878<br>2.2656
**1*-Yb** (S=0)	Wiberg bond Index	Atom Label	Wiberg bond Index	Atom Label	Wiberg bond Index
Yb1	0.0000	Fe2	0.0000	N12	0.0000
N12	0.2249	N13	0.7427	N13	2.3389
**1*-Sm** (S=3)	Wiberg bond Index	Atom Label	Wiberg bond Index	Atom Label	Wiberg bond Index
Sm1	0.0000	Fe2	0.0000	N11	0.0000
N11	0.1999	N12	0.7500	N12	2.3358
**3** (S=3)	Wiberg bond Index	Atom Label	Wiberg bond Index	Atom Label	Wiberg bond Index
Sm1	0.0000	Sm1	0.0000	Fe2	0.0000
N17	0.1702	N18	0.1725	N16	0.7119
Atom label	Wiberg bond Index	Atom Label	Wiberg bond Index	Atom Label	Wiberg bond Index
Fe3	0.0000	N16	0.0000	N18	0.0000
N19	0.7053	N17	2.3846	N19	2.4002
**2** (S=3/2)	Wiberg bond Index	Atom Label	Wiberg bond Index	Atom Label	Wiberg bond Index
U1	0.0000	Fe2	0.0000	N10	0.0000
N10	0.4358	N11	0.8003	N11	2.1768
**1-U** (S=3/2)	Wiberg bond Index	Atom Label	Wiberg bond Index	Atom Label	Wiberg bond Index
U1	0.0000	Fe2	0.0000	N13	0.0000
N13	0.5013	N14	0.7579	N14	2.1613

**A** (S=0)	Wiberg bond Index	Atom Label	Wiberg bond Index		
Fe1	0.0000	N26	0.0000		
N26	0.6103	N27	2.5212		
**$N_2U(HMDS)_3$** (S=3/2)	Wiberg bond Index	Atom Label	Wiberg bond Index		
U1	0.0000	N8	0.0000		
N8	0.5271	N9	2.7479		
**$N_2U(ArO)_3$** (S=3/2)	Wiberg bond Index	Atom Label	Wiberg bond Index		
U1	0.0000	N5	0.0000		
N5	0.4548	N6	2.8052		

Table ST8. Computed natural charges (small core, large core) for the FeN_2Ln/U core.

	**1-Ce**	**1-Sm**	**1-Dy**	**1*-Yb**	**1*-Sm**	**3**	**1-U**	**2**
Ln/U	1.91455<br>2.08633	1.94680<br>2.03213	1.90292<br>2.02034	1.42366	1.51747	1.47972	1.61568	1.62517
N	-0.31663<br>-0.38043	-0.32146<br>-0.37222	-0.33280<br>-0.39289	-0.31254	-0.33194	-0.28935, -0.28068	-0.34898	-0.37707
N	-0.03694<br>-0.02820	-0.04616<br>-0.02973	-0.05808<br>-0.03071	-0.03491	-0.03989	-0.04476, -0.03929	-0.05617	-0.02627
Fe	-0.51181<br>-0.48368	-0.48679<br>-0.49494	-0.47864<br>-0.48415	-0.53811	-0.54023	-0.55031, -0.56539	-0.57201	-0.57414
	**A**	$N_2U(HMDS)_3$	$N_2U(OAr)_3$					
Ln/U		1.62265	1.60634					
N	-0.16685	-0.11017	-0.09860					
N	-0.08336	0.06588	0.09204					
Fe	-0.60233							

Table ST9. NBO Analysis of Canonical Molecular Orbitals for metal-to-N_2 back bonding (small core).

**A**	MO 108 (occ): orbital energy = -0.13237 a.u.<br>0.730*[30]: LP (4)Fe 1(lp)<br>-0.353*[183]: BD*(2) N26- N27*<br>-0.267*[31]: LP (1) P 2(lp)<br>0.261*[33]: LP (1) P 4(lp)
**1-Ce** (BMO-HOMO)	MO 227 (occ): orbital energy = -0.15373 a.u.<br>-0.532*[226]: BD*(1)Fe 2- P 6*<br>-0.448*[280]: BD*(2) N13- N14*<br>0.432*[241]: BD*(1)Fe 2- P 4*<br>-0.341*[62]: LP (2)Fe 2(lp)
**1-Sm** (BMO-HOMO)	MO 227 (occ): orbital energy = -0.14724 a.u.<br>0.672*[225]: BD*(1)Fe 2- P 4*<br>-0.470*[281]: BD*(3) N13- N14*<br>-0.295*[226]: BD*(1)Fe 2- P 6*<br>-0.225*[69]: BD (1)Fe 2- P 4
**1-Dy** (BMO-HOMO)	MO 229 (occ): orbital energy = -0.14657 a.u.<br>0.687*[228]: BD*(1)Fe 2- N13*<br>-0.456*[242]: BD*(1)Fe 2- P 5*<br>0.225*[243]: BD*(1)Fe 2- N14*<br>0.225*[77]: BD (2)Fe 2- N14
**1*-Yb** (HOMO-7)	MO 213 (occ): orbital energy = -0.15207 a.u.<br>0.594*[227]: BD*(1)Fe 2- P 5*<br>-0.470*[261]: BD*(3) N12- N13*<br>-0.468*[220]: BD*(1)Fe 2- P 4*
**1*-Sm**	MO 192 (occ): orbital energy = -0.15330 a.u.

(BMO-HOMO)	0.539*[206]: BD*(1)Fe 2- P 5*<br>-0.538*[191]: BD*(1)Fe 2- P 4*<br>0.497*[238]: BD*(3) N11- N12*<br>-0.225*[96]: BD (3) N11- N12
**3**<br>(AMO-HOMO-6, HOMO-7)	MO 301 (occ): orbital energy = -0.14009 a.u.<br>0.505*[300]: BD*(1)Fe 3- P11*<br>-0.392*[299]: BD*(1)Fe 2- P 5*<br>0.331*[363]: BD*(2) N18- N19*<br>0.271*[361]: BD*(3) N16- N17*<br>0.252*[316]: LV (1)Fe 3(lv)<br>MO 300 (occ): orbital energy = -0.14245 a.u.<br>0.466*[299]: BD*(1)Fe 2- P 5*<br>0.398*[300]: BD*(1)Fe 3- P11*<br>-0.343*[361]: BD*(3) N16- N17*<br>0.273*[363]: BD*(2) N18- N19*<br>0.246*[315]: LV (1)Fe 2(lv)<br>-0.231*[87]: LP (1) P 6(lp)
**1-U**<br>(AMO-HOMO-3)	MO 227 (occ): orbital energy = -0.16841 a.u.<br>0.553*[229]: BD*(1)Fe 2- P 4*<br>-0.413*[242]: BD*(1)Fe 2- P 5*<br>-0.406*[281]: BD*(3) N13- N14*<br>-0.266*[65]: LP (2)Fe 2(lp)<br>-0.226*[62]: LP (2) U 1(lp)
**2**<br>(AMO-HOMO-4)	MO 293 (occ): orbital energy = -0.16013 a.u.<br>-0.622*[289]: BD*(1)Fe 2- P 4*<br>0.432*[332]: BD*(3) N10- N11*<br>0.261*[290]: BD*(1)Fe 2- P 5*<br>-0.231*[311]: BD*(1)Fe 2- N11*
$N_2U(HMDS)_3$	MO 129 (vir): orbital energy = -0.06472 a.u. (HOMO)<br>0.713*[165]: BD*(3) N 8- N 9*<br>0.338*[164]: BD*(2) N 8- N 9*<br>0.268*[136]: BD*(1) U 1- N11*<br>-0.234*[130]: LV (2) U 1(lv)<br>-0.229*[135]: BD*(2) U 1- N10*<br>MO 128 (occ): orbital energy = -0.15804 a.u. (HOMO-1)<br>-0.584*[128]: BD*(2) U 1- N12*<br>-0.404*[129]: LV (1) U 1(lv)<br>-0.330*[164]: BD*(2) N 8- N 9*<br>0.327*[133]: BD*(1) U 1- N 8*<br>-0.319*[37]: LP (1) U 1(lp)<br>-0.249*[38]: LP (2) U 1(lp)
$N_2U(OAr)_3$	MO 194 (occ): orbital energy = -0.16708 a.u. (HOMO)<br>0.592*[195]: LV (1) U 1(lv)<br>0.358*[201]: BD*(2) U 1- O 2*<br>0.329*[203]: BD*(2) U 1- O 3*<br>0.298*[61]: LP (1) U 1(lp)<br>0.246*[211]: BD*(3) N 5- N 6*<br>0.229*[205]: BD*(2) U 1- O 4*<br>MO 193 (occ): orbital energy = -0.17331 a.u. (HOMO-1)<br>-0.902*[62]: LP (2) U 1(lp)<br>0.258*[210]: BD*(2) N 5- N 6*

Table ST10. Computed MOs for [(depe)$_2$FeN$_2$Ln(HMDS)$_n$] (n = 2 or 3) complexes (small core).

	1-Ce (S=1/2)		1-Sm (S=5/2)		1*-Sm (S=3)		1-Dy (S=5/2)
				AMO-HOMO-11	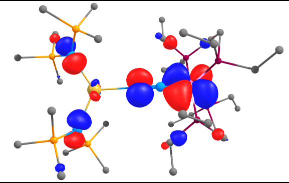	AMO-HOMO-108	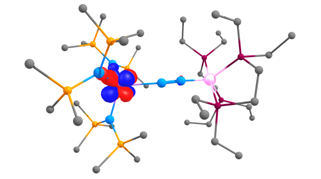
		AMO-HOMO-38	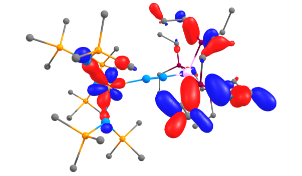	AMO-HOMO-10	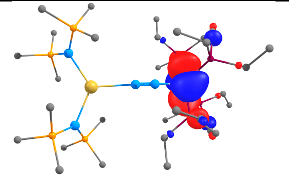	AMO-HOMO-107	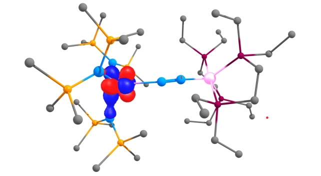
		AMO-HOMO-35	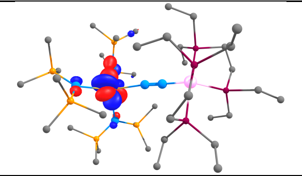	AMO-HOMO-7	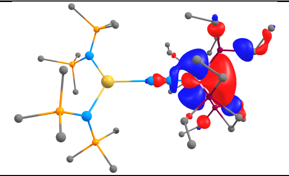	AMO-HOMO-105	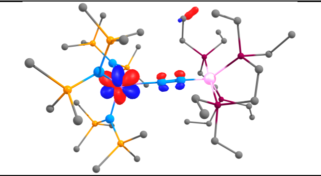
		AMO-HOMO-30	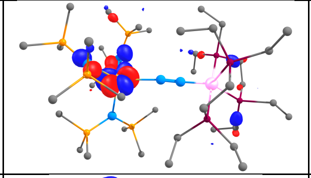	AMO-HOMO-6	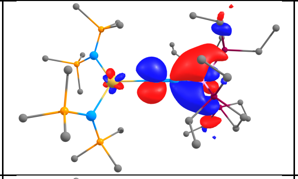	AMO-HOMO-104	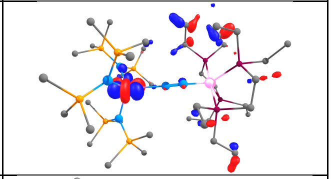
		AMO-HOMO-20	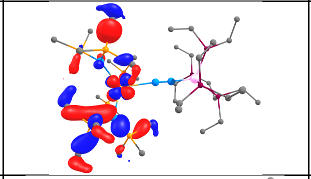	AMO-HOMO-5	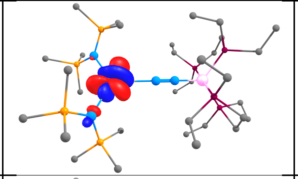	AMO-HOMO-101	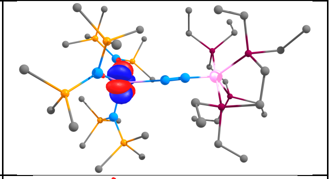
AMO-HOMO-2	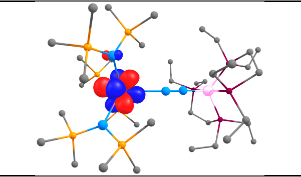	AMO-HOMO-19	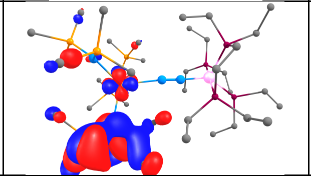	AMO-HOMO-4	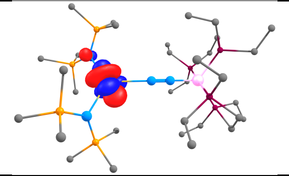	AMO-HOMO-93	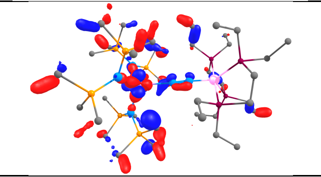

AMO-HOMO-1	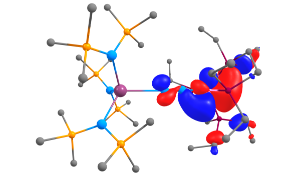	AMO-HOMO-6	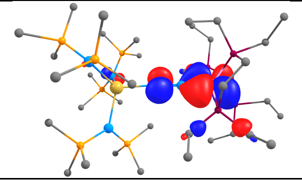	AMO-HOMO-3	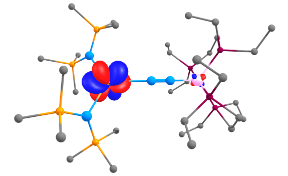	AMO-HOMO-68	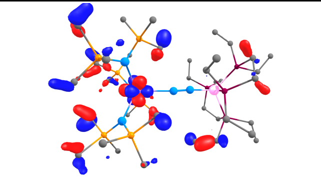
AMO-HOMO	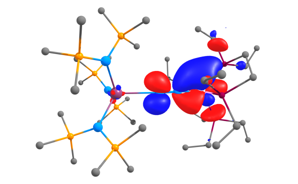	AMO-HOMO-5	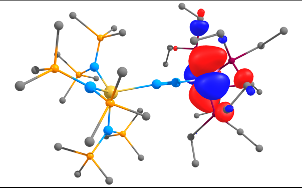	AMO-HOMO-2	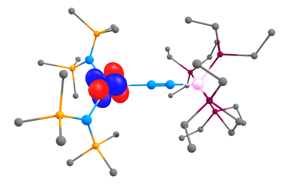	AMO-HOMO-6	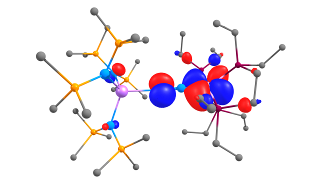
AMO-LUMO	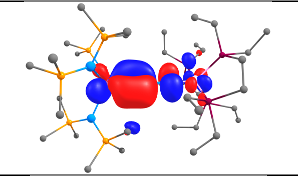	AMO-HOMO-1	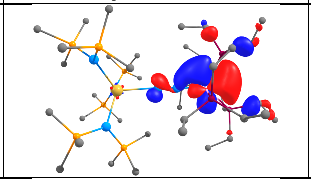	AMO-HOMO-1	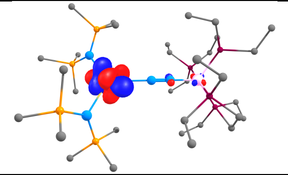	AMO-HOMO-3	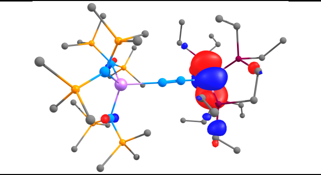
BMO-HOMO-1	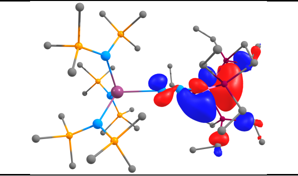	AMO-HOMO	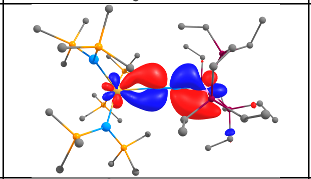	AMO-HOMO	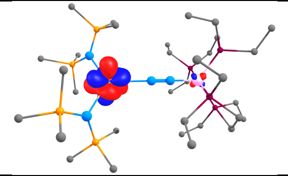	AMO-HOMO-1	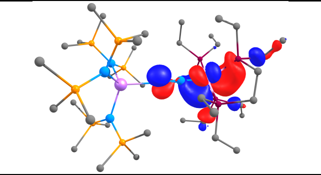
BMO-HOMO	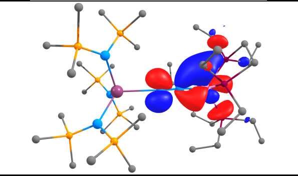	AMO-LUMO	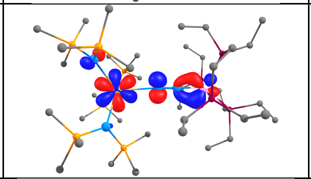	AMO-LUMO	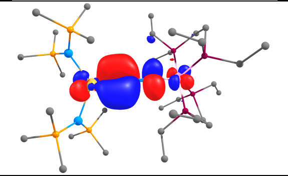	AMO-HOMO	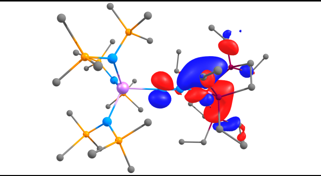
BMO-LUMO	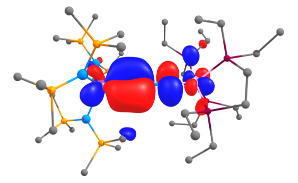	BMO-HOMO-5	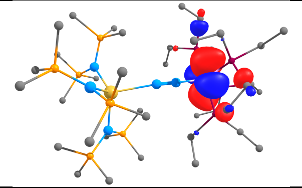	BMO-HOMO-5	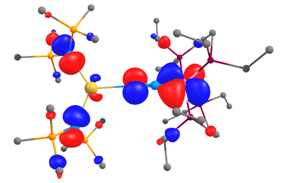	AMO-LUMO	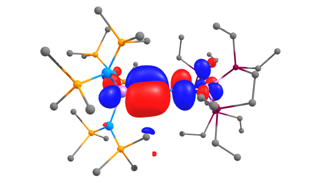

		BMO-HOMO-1	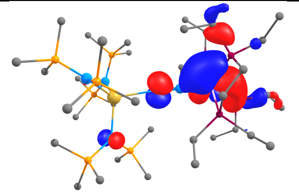	BMO-HOMO-4	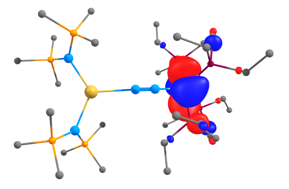	BMO-HOMO-20	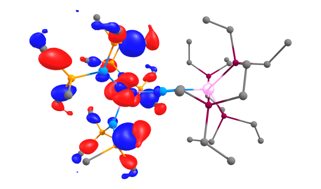
		BMO-HOMO	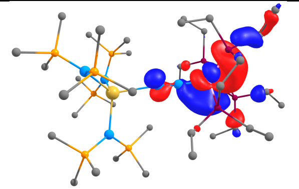	BMO-HOMO-1	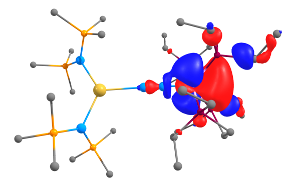	BMO-HOMO-10	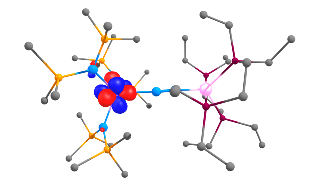
		BMO-LUMO	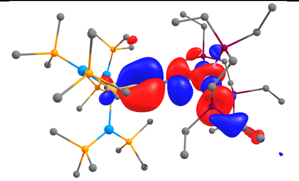	BMO-HOMO	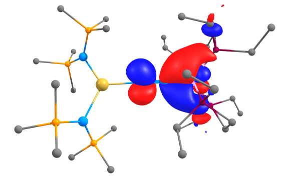	BMO-HOMO-4	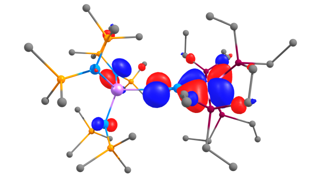
				BMO-LUMO	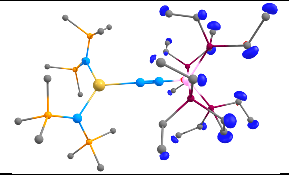	BMO-HOMO-2	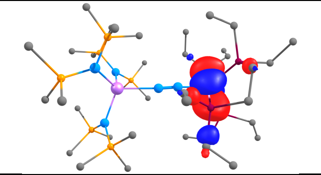
						BMO-HOMO-1	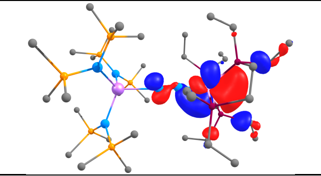
						BMO-HOMO	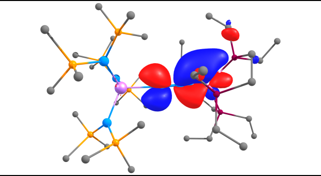

						BMO-LUMO	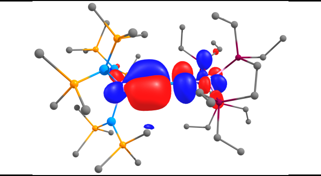

Table ST11. Computed MOs for [{(depe)$_2$FeN$_2$}$_n$Ln(X)$_m$] (n = 1 or 2, m = 2 or 3, X = HMDS or OAr) complexes (small core).

	**1*-Yb (S=0)**		**3 (S=3)**		**A**		**1-U (S=3/2)**		**2 (S=3/2)**
		AMO-HOMO-15	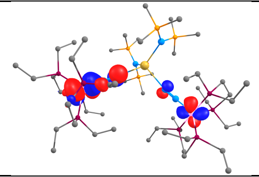						
		AMO-HOMO-14	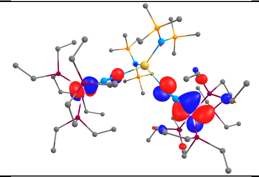						
		AMO-HOMO-13	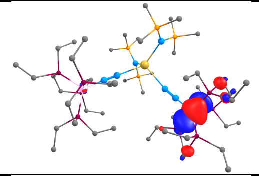						
HOMO-13	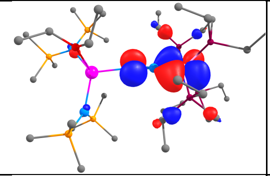	AMO-HOMO-12	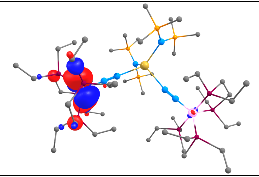						
HOMO-12	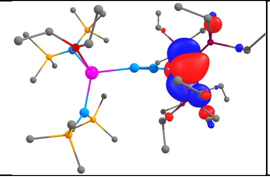	AMO-HOMO-9	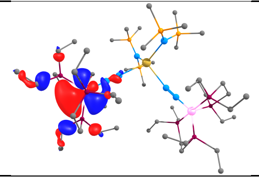						

HOMO-8	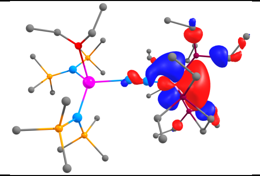	AMO-HOMO-8	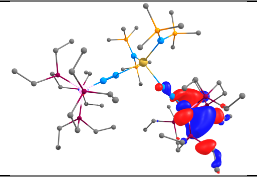						
HOMO-7	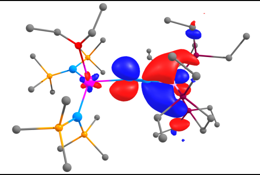	AMO-HOMO-7	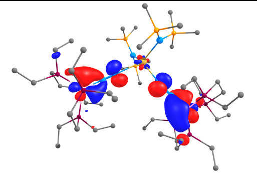						
HOMO-6	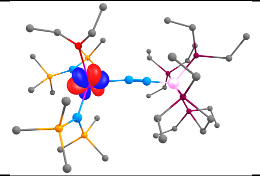	AMO-HOMO-6	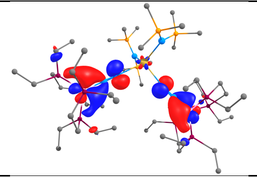			AMO-HOMO-9	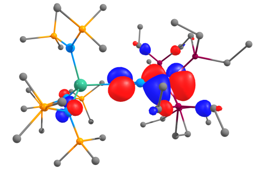	AMO-HOMO-11	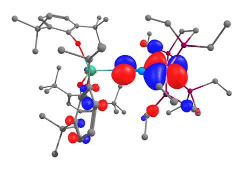
HOMO-5	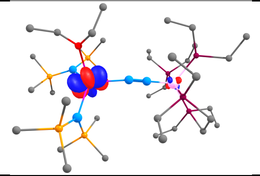	AMO-HOMO-5	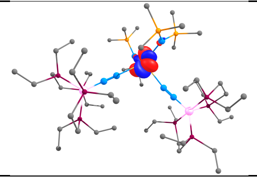			AMO-HOMO-7	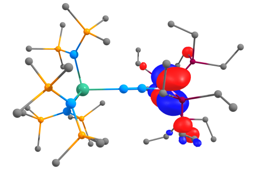	AMO-HOMO-8	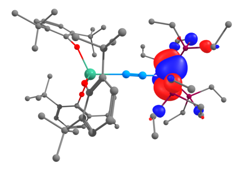
HOMO-4	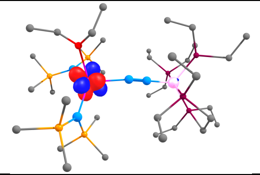	AMO-HOMO-4	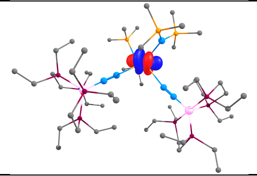			AMO-HOMO-4	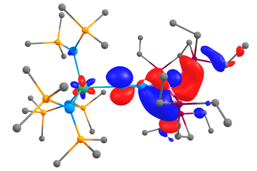	AMO-HOMO-4	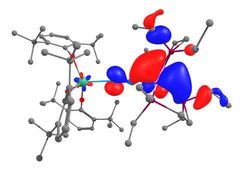
HOMO-3	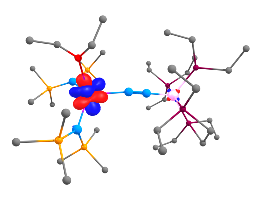	AMO-HOMO-3	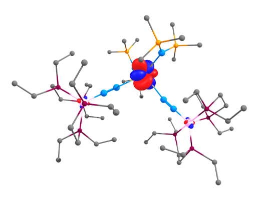	HOMO-3	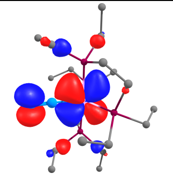 ε= -0.17848	AMO-HOMO-3	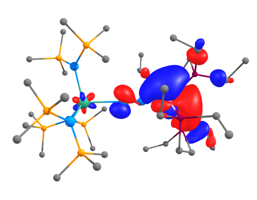	AMO-HOMO-3	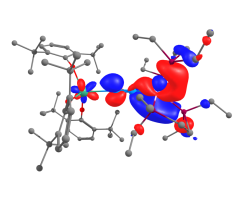
HOMO-2	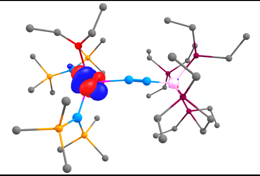	AMO-HOMO-2	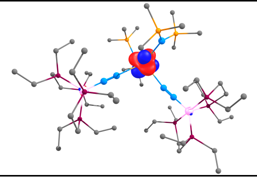	HOMO-2	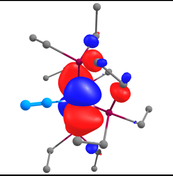	AMO HOMO-2	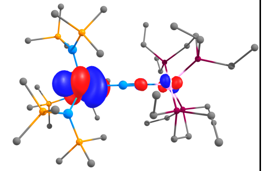	AMO-HOMO-2	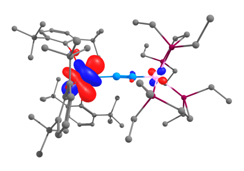

					ε= -0.17605				
HOMO-1	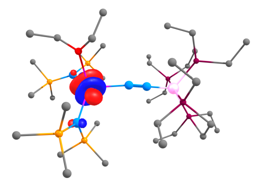	AMO-HOMO-1	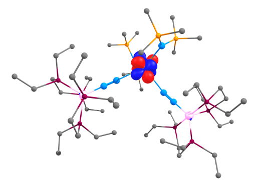	HOMO-1	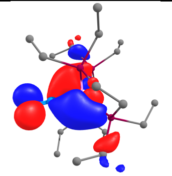 ε= -0.13234	AMO-HOMO-1	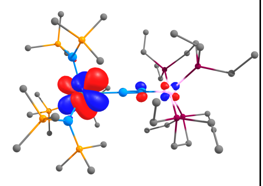	AMO-HOMO-1	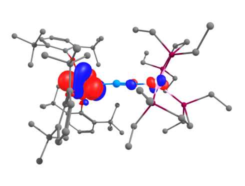
HOMO	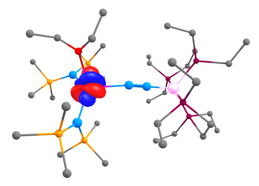	AMO-HOMO	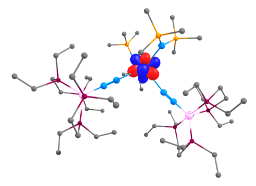	HOMO	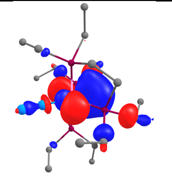 ε= -0.13003	AMO-HOMO	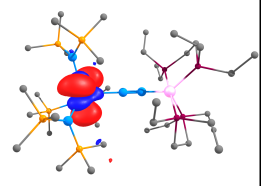	AMO-HOMO	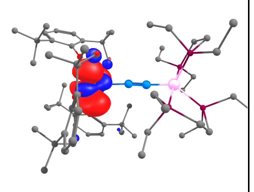
LUMO	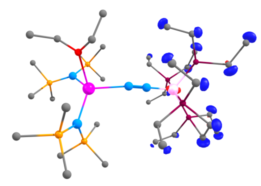	AMO-LUMO	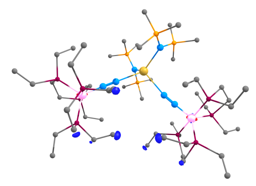	LUMO	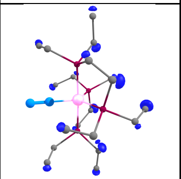 ε= 0.00062	AMO-LUMO	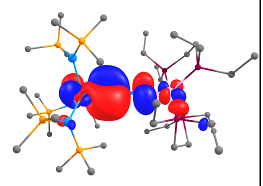	AMO-LUMO	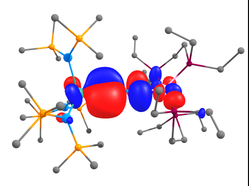
		BMO-HOMO-9	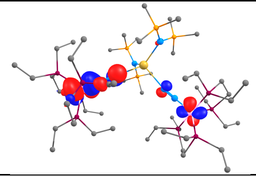			BMO-HOMO-3	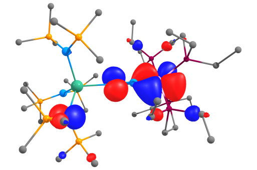	BMO-HOMO-6	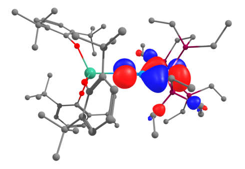
		BMO-HOMO-8	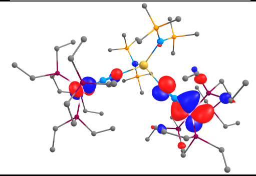			BMO-HOMO-2	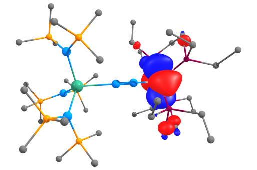	BMO-HOMO-5	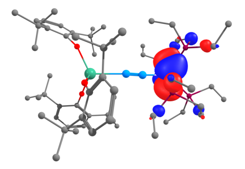
		BMO-HOMO-7	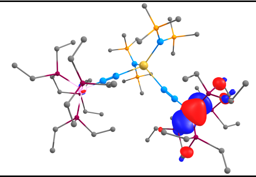			BMO-HOMO-1	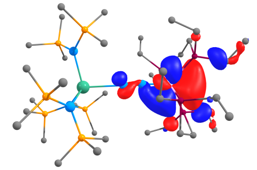	BMO-HOMO-1	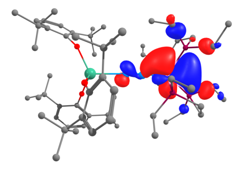

		BMO-HOMO-6	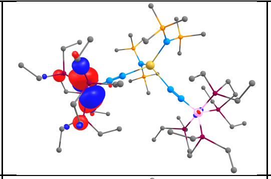			BMO-HOMO	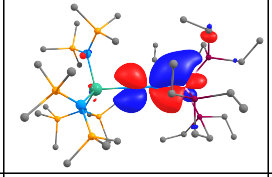	BMO-HOMO	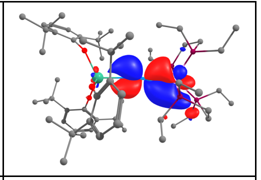
		BMO-HOMO-3	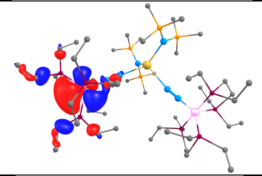			BMO-LUMO	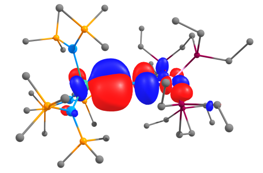	BMO-LUMO	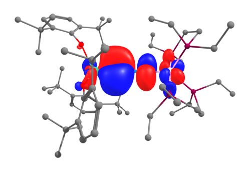
		BMO-HOMO-2	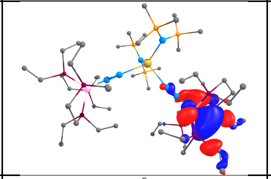						
		BMO-HOMO-1	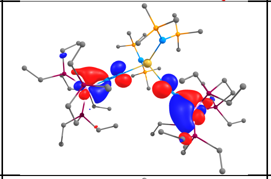						
		BMO-HOMO	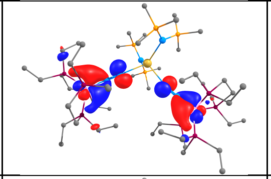						
		BMO-LUMO	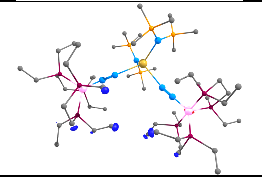						

Table ST12. Computed MOs for $N_2U(HMDS)_3$ and $N_2U(OAr)_3$ complexes.

	$N_2U(HMDS)_3$	$N_2U(OAr)_3$
AMO-HOMO-2	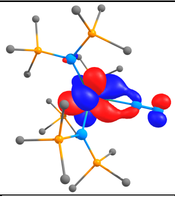	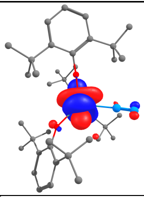
AMO-HOMO-1	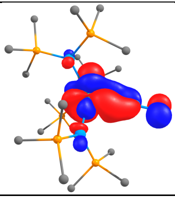	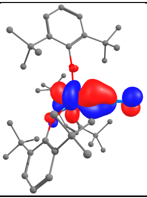
AMO-HOMO	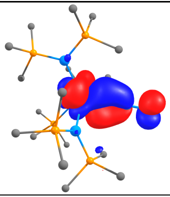	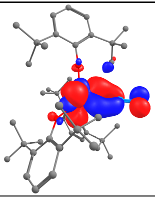
AMO-LUMO	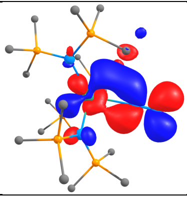	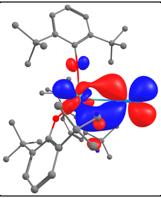

Table ST13. Computed MOs for **1-Ln** (large core).

	**1-Ce**	**1-Sm**	**1-Dy**
HOMO-6	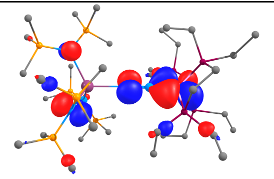 ε= -0.20914	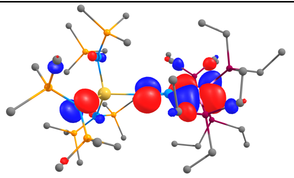 ε= -0.21048	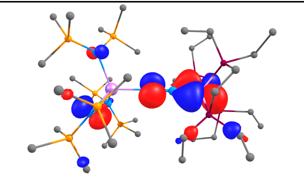 ε= -0.21036
HOMO-4/ HOMO-3	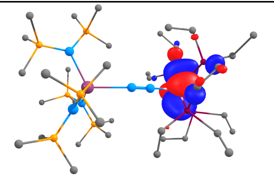 ε= -0.20195	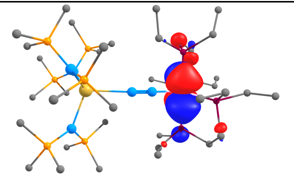 ε= -0.20086	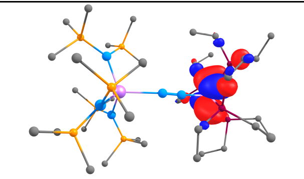 ε= -0.20111
HOMO-1	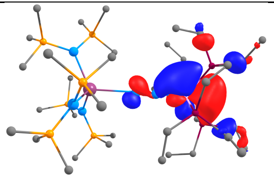 ε= -0.16446	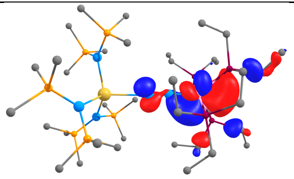 ε= -0.16847	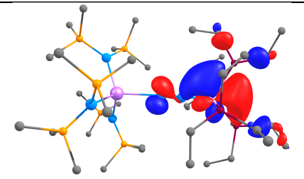 ε= -0.16829
HOMO	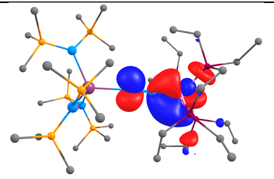 ε= -0.15736	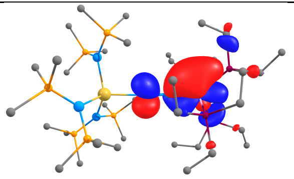 ε= -0.15473	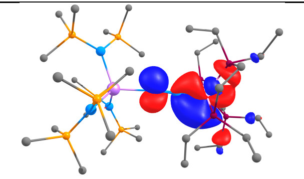 ε= -0.15469
LUMO	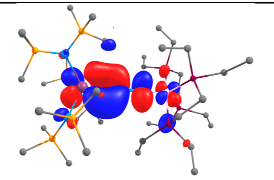 ε= -0.02216	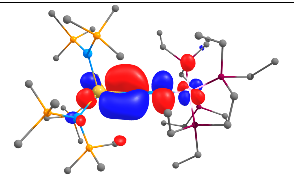 ε= -0.02164	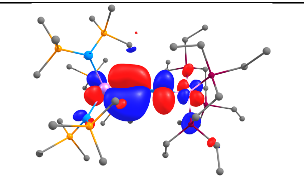 ε= -0.02112

Optimized Coordinates

1-Ce, S=1/2, small core

Ce	9.394056000	8.103014000	15.865607000
Fe	8.780971000	6.432011000	10.501874000
P	6.513527000	6.254165000	10.659750000
P	8.645061000	4.316967000	9.712759000
P	11.043002000	6.543254000	10.276478000
P	8.762371000	8.019451000	8.915549000
Si	6.987053000	10.580060000	15.524890000
Si	8.875611000	11.179317000	17.829207000
Si	7.319718000	6.447757000	18.464895000
Si	8.952377000	4.733311000	16.600076000
Si	12.824320000	7.606441000	17.218965000
Si	12.381977000	9.634579000	15.020455000
N	9.036877000	7.297299000	13.255079000
N	8.926079000	6.925102000	12.166181000
N	8.363445000	10.124135000	16.511431000
N	8.430901000	6.337152000	17.093602000
N	11.726815000	8.404786000	16.090418000
C	6.400951000	9.017688000	14.596684000
H	7.085353000	8.717853000	13.794988000
H	5.437449000	9.235011000	14.123977000
H	6.244607000	8.165059000	15.267860000
C	7.384641000	11.875580000	14.186507000
H	8.181490000	11.539113000	13.516368000
H	7.703526000	12.823174000	14.631456000

H	6.494984000	12.076768000	13.577563000
C	5.469083000	11.259298000	16.448004000
H	5.656627000	12.257849000	16.855072000
H	5.152773000	10.609352000	17.267924000
H	4.634261000	11.347220000	15.742254000
C	9.326174000	12.929905000	17.226280000
H	8.435357000	13.476708000	16.897837000
H	10.030167000	12.907441000	16.388809000
H	9.783094000	13.508493000	18.037443000
C	7.585420000	11.413318000	19.211512000
H	7.266220000	10.453444000	19.629056000
H	6.692252000	11.953042000	18.887362000
H	8.044856000	11.990067000	20.023585000
C	10.379575000	10.437533000	18.715444000
H	11.182175000	10.157951000	18.029328000
H	10.091529000	9.545158000	19.282168000
H	10.775002000	11.163373000	19.435207000
C	6.087270000	7.871668000	18.249505000
H	5.361072000	7.645267000	17.461157000
H	6.593454000	8.805117000	17.991722000
H	5.528744000	8.026930000	19.179889000
C	6.215157000	4.914107000	18.732369000
H	6.767588000	3.990122000	18.925239000
H	5.552499000	4.744091000	17.877841000
H	5.580296000	5.103176000	19.606476000
C	8.253054000	6.729706000	20.097115000
H	8.822150000	7.664642000	20.078009000
H	8.959864000	5.918313000	20.297551000
H	7.552122000	6.783514000	20.938223000
C	9.698137000	3.665542000	17.989437000
H	8.999851000	3.508240000	18.816739000
H	10.604755000	4.119105000	18.400272000
H	9.966951000	2.679152000	17.592873000
C	10.311683000	4.911642000	15.277038000
H	11.112535000	5.607346000	15.551114000
H	9.901248000	5.226212000	14.313105000
H	10.784371000	3.933795000	15.130578000
C	7.590582000	3.695176000	15.765573000
H	7.078525000	4.283644000	14.997321000
H	6.839287000	3.340741000	16.475743000
H	8.033613000	2.816760000	15.281434000
C	11.852696000	6.885765000	18.681057000
H	10.909671000	6.420403000	18.378923000
H	11.615202000	7.670021000	19.406406000
H	12.452522000	6.127566000	19.197474000
C	14.134230000	8.756854000	17.989798000
H	13.676411000	9.616158000	18.489108000
H	14.857993000	9.131203000	17.258703000
H	14.695586000	8.193900000	18.745219000
C	13.823538000	6.192518000	16.417717000
H	14.409272000	6.554588000	15.566849000
H	13.190302000	5.372843000	16.066256000
H	14.524813000	5.776533000	17.150954000
C	13.896370000	9.060680000	14.016849000
H	13.714282000	8.111811000	13.505505000
H	14.770736000	8.929363000	14.662906000
H	14.157527000	9.812436000	13.262562000
C	11.020446000	10.137187000	13.780373000
H	10.163494000	10.592793000	14.292864000
H	10.659212000	9.313647000	13.155089000
H	11.421008000	10.905970000	13.109715000
C	12.906195000	11.266472000	15.845699000
H	13.762044000	11.139455000	16.513566000
H	12.091105000	11.709017000	16.424625000
H	13.194291000	11.986748000	15.070260000
C	4.466615000	5.319298000	12.532531000
H	4.254443000	4.401436000	11.975243000
H	3.755008000	6.081662000	12.200150000
H	4.250466000	5.112257000	13.586202000
C	5.916269000	5.767960000	12.371355000

H	6.606897000	4.984241000	12.699456000
H	6.128052000	6.630607000	13.011324000
C	5.210986000	7.512739000	10.140125000
H	5.506336000	7.864521000	9.145449000
H	4.271349000	6.964119000	10.004448000
C	4.998813000	8.684301000	11.092847000
H	4.310255000	9.417232000	10.658128000
H	5.932787000	9.201218000	11.326713000
H	4.566169000	8.352063000	12.040286000
C	5.928929000	4.819877000	9.602490000
H	4.887807000	4.564590000	9.827966000
H	5.963342000	5.170316000	8.563823000
C	6.880226000	3.656781000	9.817740000
H	6.722805000	2.848071000	9.096097000
H	6.740988000	3.234241000	10.817655000
C	9.566750000	2.889820000	10.535317000
H	9.346833000	1.961821000	9.996985000
H	10.636213000	3.088754000	10.406405000
C	9.240757000	2.736984000	12.017783000
H	8.232144000	2.341741000	12.173153000
H	9.301822000	3.697023000	12.538831000
H	9.935449000	2.041548000	12.500839000
C	8.957914000	3.955798000	7.873645000
H	8.125681000	4.463271000	7.370478000
H	9.857043000	4.508159000	7.590341000
C	9.061061000	2.507173000	7.401728000
H	9.103631000	2.461867000	6.307354000
H	8.202192000	1.903885000	7.714567000
H	9.963600000	2.018545000	7.780666000
C	12.174433000	6.381396000	11.757790000
H	13.114955000	6.888074000	11.514715000
H	11.702297000	6.953146000	12.561942000
C	12.433467000	4.949613000	12.216068000
H	12.944993000	4.357583000	11.449814000
H	11.502344000	4.439753000	12.472639000
H	13.064894000	4.945589000	13.109908000
C	11.976883000	5.544103000	8.977383000
H	11.790848000	4.487427000	9.193651000
H	11.468878000	5.751850000	8.029753000
C	13.476638000	5.794538000	8.831458000
H	13.891632000	5.156391000	8.043228000
H	14.024776000	5.572067000	9.751133000
H	13.697190000	6.829850000	8.555587000
C	11.430452000	8.283802000	9.700490000
H	11.204173000	8.939235000	10.547548000
H	12.492538000	8.405023000	9.461508000
C	10.526207000	8.588680000	8.513542000
H	10.861251000	8.040058000	7.625184000
H	10.531512000	9.653123000	8.255995000
C	8.097285000	7.700722000	7.172738000
H	8.637042000	6.807129000	6.838712000
H	7.053186000	7.387993000	7.299205000
C	8.196688000	8.807011000	6.124051000
H	7.864085000	8.441876000	5.145539000
H	9.224478000	9.164601000	6.003706000
H	7.572101000	9.669122000	6.374816000
C	7.982030000	9.704170000	9.257281000
H	8.184815000	10.357456000	8.401312000
H	6.898579000	9.559041000	9.295672000
C	8.478890000	10.349098000	10.546026000
H	9.535845000	10.625510000	10.475750000
H	8.374163000	9.669882000	11.396981000
H	7.920833000	11.264809000	10.768177000

1-Sm, S=5/2, small core

Sm	2.427138000	4.780453000	10.750741000
Fe	2.437997000	4.142832000	16.246785000
P	0.178361000	3.765345000	16.139394000
P	1.860915000	6.014487000	17.407957000
P	4.704178000	4.421013000	16.392266000

P	2.795209000	2.402216000	17.679283000
Si	1.671185000	1.318039000	10.726905000
Si	-0.118117000	2.933046000	8.933267000
Si	-0.038164000	7.236890000	10.911101000
Si	2.007931000	7.873604000	8.800463000
Si	5.287721000	3.613538000	8.812509000
Si	5.676678000	5.911512000	10.746254000
N	2.517126000	4.536630000	13.339214000
N	2.493983000	4.336215000	14.478375000
N	1.232661000	2.883433000	10.065853000
N	1.423279000	6.785756000	10.061995000
N	4.623302000	4.721788000	10.012440000
C	2.456113000	0.089228000	9.501029000
H	1.762919000	-0.220240000	8.715084000
H	2.766078000	-0.811801000	10.044540000
H	3.343393000	0.508545000	9.018763000
C	0.207358000	0.388138000	11.525674000
H	-0.302381000	1.010826000	12.267608000
H	0.562545000	-0.519006000	12.029154000
H	-0.535870000	0.080791000	10.782971000
C	2.965347000	1.527109000	12.109453000
H	3.818464000	2.146687000	11.809501000
H	3.366269000	0.539079000	12.365291000
H	2.534467000	1.960801000	13.015509000
C	-1.800966000	3.095568000	9.817488000
H	-1.984601000	2.230695000	10.463841000
H	-2.616441000	3.142862000	9.086103000
H	-1.859217000	3.996082000	10.436198000
C	-0.291047000	1.400849000	7.809633000
H	0.608225000	1.241177000	7.206627000
H	-1.125984000	1.571912000	7.119298000
H	-0.504308000	0.477437000	8.356814000
C	0.065240000	4.388413000	7.730517000
H	0.243892000	5.334831000	8.249097000
H	-0.842275000	4.494534000	7.124860000
H	0.902707000	4.209563000	7.047638000
C	-1.651655000	7.207360000	9.901129000
H	-1.782600000	6.256768000	9.376445000
H	-1.691702000	8.007481000	9.157975000
H	-2.505858000	7.338440000	10.576632000
C	0.042793000	8.949122000	11.744024000
H	0.936206000	9.046161000	12.369055000
H	-0.837892000	9.111597000	12.376924000
H	0.065379000	9.754924000	11.003000000
C	-0.320390000	5.976891000	12.316404000
H	-0.366152000	4.944377000	11.945970000
H	-1.286835000	6.178812000	12.792338000
H	0.455779000	6.042081000	13.085061000
C	0.630710000	8.690977000	7.763548000
H	-0.035130000	7.948770000	7.312702000
H	1.098124000	9.256949000	6.948647000
H	0.020534000	9.394712000	8.339018000
C	3.079260000	6.932799000	7.549834000
H	3.848079000	6.331154000	8.042741000
H	3.574946000	7.636423000	6.871102000
H	2.458546000	6.265135000	6.943425000
C	3.024519000	9.328920000	9.495388000
H	2.417175000	9.947941000	10.163645000
H	3.373767000	9.968723000	8.676195000
H	3.903041000	8.997979000	10.055394000
C	3.939576000	3.081103000	7.588357000
H	3.032297000	2.744442000	8.098786000
H	4.299794000	2.261967000	6.955359000
H	3.672674000	3.917022000	6.933325000
C	6.680236000	4.351811000	7.738901000
H	6.355899000	5.257931000	7.218278000
H	6.960857000	3.613680000	6.977847000
H	7.583602000	4.589544000	8.309802000
C	6.045663000	2.056111000	9.607289000
H	6.860876000	2.325611000	10.287444000

H	6.460337000	1.395761000	8.836487000
H	5.310825000	1.480916000	10.177441000
C	6.379774000	7.253122000	9.592282000
H	5.605165000	7.719646000	8.978473000
H	7.143762000	6.853182000	8.920631000
H	6.849070000	8.039951000	10.195506000
C	7.201372000	5.155160000	11.608412000
H	6.927543000	4.347545000	12.293101000
H	7.746309000	5.918837000	12.176387000
H	7.897077000	4.740160000	10.871149000
C	4.677559000	6.858353000	12.072123000
H	3.765136000	7.310978000	11.663578000
H	5.288436000	7.681679000	12.459078000
H	4.395055000	6.232994000	12.924474000
C	-0.480641000	3.048229000	14.545624000
H	-0.033507000	3.651810000	13.752749000
H	-0.018876000	2.057555000	14.466343000
C	-1.991421000	2.952511000	14.351645000
H	-2.213996000	2.588151000	13.343486000
H	-2.481971000	3.924913000	14.457342000
H	-2.455799000	2.257200000	15.057136000
C	-0.746618000	2.732321000	17.430566000
H	-0.799556000	1.724017000	17.003735000
H	-0.100812000	2.660232000	18.308207000
C	-2.138721000	3.187890000	17.867751000
H	-2.564764000	2.461774000	18.569300000
H	-2.109328000	4.153011000	18.382402000
H	-2.833959000	3.279235000	17.030226000
C	-0.645778000	5.440065000	16.279147000
H	-0.433643000	5.955302000	15.337452000
H	-1.732570000	5.355137000	16.373141000
C	-0.025059000	6.176868000	17.461130000
H	-0.357983000	5.732212000	18.407164000
H	-0.317193000	7.232449000	17.478556000
C	2.272474000	6.264118000	19.235510000
H	1.842352000	5.388889000	19.733471000
H	3.360059000	6.152121000	19.322229000
C	1.811063000	7.545056000	19.928195000
H	0.733069000	7.702664000	19.820081000
H	2.318429000	8.431058000	19.536774000
H	2.025624000	7.498406000	21.001876000
C	2.313677000	7.717765000	16.741154000
H	1.807961000	8.466581000	17.360649000
H	3.388179000	7.847823000	16.903280000
C	1.966911000	7.929400000	15.271727000
H	2.414591000	7.162439000	14.634214000
H	2.324773000	8.905633000	14.927516000
H	0.885492000	7.908899000	15.105428000
C	5.741020000	3.924832000	14.910296000
H	5.376538000	4.530500000	14.075975000
H	6.772270000	4.241759000	15.107929000
C	5.696923000	2.447536000	14.535420000
H	6.210123000	2.283415000	13.582595000
H	6.198259000	1.824404000	15.282915000
H	4.668837000	2.095248000	14.418137000
C	5.588191000	6.023310000	16.849194000
H	5.034135000	6.458484000	17.688079000
H	6.571688000	5.733442000	17.240189000
C	5.757280000	7.038446000	15.722559000
H	6.191577000	7.968019000	16.106501000
H	6.426070000	6.664963000	14.942870000
H	4.807922000	7.287619000	15.242492000
C	5.410728000	3.336056000	17.755226000
H	6.482266000	3.178342000	17.584457000
H	5.310396000	3.905735000	18.687229000
C	4.633545000	2.033011000	17.835281000
H	4.837947000	1.487627000	18.763241000
H	4.894283000	1.373690000	17.003437000
C	2.161195000	0.659365000	17.338914000
H	2.649224000	-0.021150000	18.045486000

H	1.093472000	0.648395000	17.579033000
C	2.388288000	0.195581000	15.904599000
H	1.958870000	0.897429000	15.184721000
H	1.935090000	-0.787657000	15.738396000
H	3.453240000	0.106627000	15.669417000
C	2.385371000	2.582780000	19.524967000
H	3.097895000	3.337335000	19.879689000
H	1.400207000	3.055519000	19.592789000
C	2.436185000	1.344009000	20.416301000
H	1.659164000	0.620229000	20.153957000
H	2.282794000	1.620438000	21.465615000
H	3.401056000	0.829630000	20.357844000

1-Dy, S=5/2, small core

Dy	2.332950000	13.435597000	10.815644000
Fe	1.979425000	14.198870000	16.176621000
P	-0.281790000	14.390802000	16.010715000
P	1.758345000	16.050454000	17.480953000
P	2.026029000	12.304268000	17.392218000
P	4.244222000	14.122550000	16.356973000
Si	1.901957000	10.312089000	9.063237000
Si	-0.301442000	11.232969000	10.884390000
Si	0.085374000	15.269032000	8.704989000
Si	1.821357000	16.827028000	10.613737000
Si	5.495695000	12.171932000	11.141413000
Si	5.322219000	14.317955000	9.027477000
N	2.182393000	13.749878000	13.313547000
N	2.108034000	13.981871000	14.449054000
N	1.276755000	11.543555000	10.173454000
N	1.343829000	15.263582000	9.952124000
N	4.510904000	13.334115000	10.258447000
C	2.757379000	8.876480000	9.977784000
H	3.589503000	9.209595000	10.602919000
H	3.152577000	8.152560000	9.255229000
H	2.048166000	8.347446000	10.622326000
C	3.121281000	11.066483000	7.823975000
H	2.590940000	11.710535000	7.115138000
H	3.612738000	10.271633000	7.251155000
H	3.891169000	11.662716000	8.319399000
C	0.570461000	9.475961000	7.983692000
H	-0.131324000	8.861247000	8.555901000
H	1.078572000	8.812308000	7.273480000
H	-0.003805000	10.204069000	7.403012000
C	-0.408584000	9.571958000	11.810573000
H	-0.371036000	8.720583000	11.123370000
H	-1.353835000	9.505517000	12.362465000
H	0.412896000	9.462655000	12.525070000
C	-0.664064000	12.585663000	12.174834000
H	-0.026208000	12.485019000	13.056946000
H	-1.707230000	12.498705000	12.498805000
H	-0.541590000	13.597093000	11.767973000
C	-1.785414000	11.270252000	9.692250000
H	-1.791214000	12.169393000	9.070553000
H	-2.712912000	11.262308000	10.277816000
H	-1.808196000	10.403557000	9.027558000
C	-1.669099000	15.248398000	9.448540000
H	-1.858665000	14.358236000	10.054158000
H	-2.417467000	15.269850000	8.647474000
H	-1.835561000	16.125847000	10.081815000
C	0.112033000	16.788001000	7.553304000
H	-0.113791000	17.728711000	8.064566000
H	-0.656740000	16.642181000	6.784751000
H	1.073457000	16.896137000	7.042462000
C	0.270724000	13.782739000	7.543577000
H	1.166020000	13.895851000	6.923583000
H	-0.594730000	13.720701000	6.873720000
H	0.351175000	12.841514000	8.093049000
C	0.354515000	17.811211000	11.329763000
H	-0.349453000	18.123919000	10.551941000
H	0.714402000	18.717244000	11.831529000

H	-0.197336000	17.213345000	12.061905000
C	3.042511000	16.586078000	12.054845000
H	2.546564000	16.213330000	12.953853000
H	3.486744000	17.560544000	12.289416000
H	3.871891000	15.909756000	11.817013000
C	2.710821000	18.002012000	9.407396000
H	3.563871000	17.520820000	8.921485000
H	3.090848000	18.862844000	9.971108000
H	2.051544000	18.385164000	8.625359000
C	4.440377000	11.388076000	12.524122000
H	4.149004000	12.109304000	13.292091000
H	5.029864000	10.601249000	13.009397000
H	3.527610000	10.912561000	12.146036000
C	7.019632000	12.956583000	11.976174000
H	7.761668000	13.266136000	11.232891000
H	7.505345000	12.230710000	12.639312000
H	6.755461000	13.837001000	12.568256000
C	6.177910000	10.707472000	10.134435000
H	6.580688000	9.957009000	10.825606000
H	6.989188000	11.009424000	9.467467000
H	5.408305000	10.223400000	9.528706000
C	6.127522000	15.879734000	9.762368000
H	5.397149000	16.549696000	10.223621000
H	6.643291000	16.441591000	8.974648000
H	6.869823000	15.617335000	10.523168000
C	6.729385000	13.418715000	8.107725000
H	7.580296000	13.170728000	8.749552000
H	7.099946000	14.084844000	7.319166000
H	6.381781000	12.499241000	7.627300000
C	4.110091000	14.843896000	7.668236000
H	3.828948000	13.981873000	7.054951000
H	4.583600000	15.582275000	7.010862000
H	3.197883000	15.283039000	8.079940000
C	-2.114578000	12.450267000	17.165492000
H	-2.756587000	11.584486000	16.969722000
H	-2.728201000	13.203408000	17.668430000
H	-1.342918000	12.128291000	17.871165000
C	-1.527043000	12.980234000	15.859647000
H	-2.336291000	13.328062000	15.209956000
H	-1.014871000	12.183921000	15.312347000
C	-2.184952000	16.048863000	14.526327000
H	-2.361737000	16.569996000	13.579482000
H	-2.332427000	16.778670000	15.328966000
H	-2.966496000	15.288080000	14.622859000
C	-0.780124000	15.453228000	14.542596000
H	-0.022105000	16.239840000	14.482166000
H	-0.619273000	14.817545000	13.667115000
C	-0.942554000	15.391168000	17.447632000
H	-1.995682000	15.656322000	17.304567000
H	-0.875766000	14.760186000	18.340234000
C	-0.044954000	16.612150000	17.578799000
H	-0.225674000	17.162495000	18.508136000
H	-0.224138000	17.305218000	16.750962000
C	2.107422000	15.947399000	19.345342000
H	1.324553000	15.274727000	19.715596000
H	3.048320000	15.404625000	19.462906000
C	2.138520000	17.231709000	20.171118000
H	1.235162000	17.836630000	20.038606000
H	2.997243000	17.859787000	19.916472000
H	2.213936000	16.997726000	21.239257000
C	2.582738000	17.692632000	17.049930000
H	2.307123000	18.437052000	17.804393000
H	3.663166000	17.532631000	17.138093000
C	2.245163000	18.200001000	15.652350000
H	2.404517000	17.423293000	14.899253000
H	2.868491000	19.061562000	15.390011000
H	1.202390000	18.524710000	15.577960000
C	1.727737000	10.390339000	15.294288000
H	1.573812000	11.221148000	14.600957000
H	1.194673000	9.516508000	14.905223000

H	2.795138000	10.148432000	15.282357000
C	1.245246000	10.734307000	16.698825000
H	1.460215000	9.909741000	17.387525000
H	0.160887000	10.879436000	16.705651000
C	1.552488000	10.874720000	19.933224000
H	0.870180000	10.124780000	19.523043000
H	1.294675000	11.000812000	20.991044000
H	2.565080000	10.460075000	19.893880000
C	1.452544000	12.207590000	19.193761000
H	2.050721000	12.970873000	19.704398000
H	0.420967000	12.578464000	19.210778000
C	3.813795000	11.698173000	17.594523000
H	4.158120000	12.014800000	18.586259000
H	3.846412000	10.603548000	17.577930000
C	4.688640000	12.307576000	16.506274000
H	4.469998000	11.861049000	15.531578000
H	5.757228000	12.166814000	16.701878000
C	5.329533000	14.673559000	14.937554000
H	6.293990000	14.162171000	15.029057000
H	4.849891000	14.292308000	14.031848000
C	5.521748000	16.183363000	14.828473000
H	6.131212000	16.426340000	13.952243000
H	6.028663000	16.600069000	15.705204000
H	4.565206000	16.697616000	14.711921000
C	5.170792000	14.824960000	17.841203000
H	4.940355000	15.894300000	17.884392000
H	4.697567000	14.377522000	18.721682000
C	6.682002000	14.609308000	17.896679000
H	7.196519000	15.067058000	17.047324000
H	6.946574000	13.547970000	17.912362000
H	7.094913000	15.057404000	18.807383000

1*-Yb, S=0, small core

Yb	6.495408000	14.210359000	3.094728000
Fe	5.675943000	13.497247000	8.375320000
P	4.900248000	11.429759000	7.891647000
P	7.140137000	12.390913000	9.672801000
P	3.851284000	14.110846000	9.518451000
P	6.454811000	15.537092000	8.971489000
Si	7.925381000	16.342816000	0.569732000
Si	6.990275000	17.629793000	3.171981000
Si	9.047809000	12.118193000	2.945834000
Si	6.643273000	10.858732000	1.558841000
O	4.134282000	14.487875000	2.353025000
N	6.018775000	14.045448000	5.551078000
N	5.895138000	13.838059000	6.679581000
N	7.135556000	16.265970000	2.115775000
N	7.389517000	12.145261000	2.447354000
C	7.700771000	14.682539000	-0.334482000
H	7.949877000	13.817741000	0.289581000
H	8.332764000	14.642025000	-1.229240000
H	6.663365000	14.561337000	-0.669985000
C	9.798880000	16.691942000	0.675874000
H	9.995546000	17.660989000	1.148638000
H	10.256717000	16.710865000	-0.320197000
H	10.313541000	15.926189000	1.266082000
C	7.214435000	17.642057000	-0.640199000
H	7.635477000	17.483348000	-1.640463000
H	7.444889000	18.668954000	-0.343450000
H	6.124909000	17.556624000	-0.724315000
C	5.325044000	17.538564000	4.113402000
H	4.488876000	17.633244000	3.411907000
H	5.242298000	18.355096000	4.840489000
H	5.195125000	16.598391000	4.663252000
C	7.007627000	19.362914000	2.375356000
H	7.959019000	19.575712000	1.876785000
H	6.865604000	20.125044000	3.151090000
H	6.207634000	19.482157000	1.638085000
C	8.367229000	17.672353000	4.497555000
H	8.419445000	16.727413000	5.050599000

H	8.214354000	18.484686000	5.218571000
H	9.345223000	17.821521000	4.026919000
C	10.323488000	12.003612000	1.534953000
H	10.217573000	11.060196000	0.987552000
H	11.347888000	12.046286000	1.923488000
H	10.199885000	12.819219000	0.815675000
C	9.509961000	10.740144000	4.183103000
H	8.794820000	10.707793000	5.011162000
H	10.507820000	10.916481000	4.602148000
H	9.517530000	9.753886000	3.709703000
C	9.393416000	13.759900000	3.876711000
H	9.224948000	14.658198000	3.266991000
H	10.442969000	13.795317000	4.189373000
H	8.796160000	13.841110000	4.796112000
C	4.758074000	10.861108000	1.866607000
H	4.527394000	10.727725000	2.929924000
H	4.268404000	10.050781000	1.314481000
H	4.308219000	11.802942000	1.535828000
C	7.241507000	9.101233000	2.011713000
H	8.286217000	8.942954000	1.722445000
H	6.639705000	8.350960000	1.485107000
H	7.161618000	8.904476000	3.085624000
C	6.862284000	10.981428000	-0.331664000
H	6.462802000	11.920705000	-0.727031000
H	6.351596000	10.154440000	-0.839632000
H	7.921613000	10.939678000	-0.606704000
C	5.363047000	10.255881000	9.278744000
H	4.690026000	10.480040000	10.113545000
H	5.191308000	9.213080000	8.990360000
C	6.810337000	10.523213000	9.661525000
H	7.485613000	10.082989000	8.920523000
H	7.067871000	10.083341000	10.630515000
C	3.098003000	10.952539000	7.645698000
H	3.032515000	9.858844000	7.678709000
H	2.552308000	11.323355000	8.520009000
C	2.478306000	11.486675000	6.357678000
H	2.946874000	11.042877000	5.473934000
H	2.588798000	12.572349000	6.282438000
H	1.408948000	11.253043000	6.311538000
C	5.700579000	10.626940000	6.396387000
H	5.452524000	11.261182000	5.539351000
H	6.777004000	10.761104000	6.542106000
C	5.369790000	9.166600000	6.102838000
H	5.905334000	8.833030000	5.208443000
H	4.302768000	9.010770000	5.915525000
H	5.663651000	8.502527000	6.922056000
C	7.204191000	12.704089000	11.543549000
H	7.251442000	13.791136000	11.670353000
H	6.211745000	12.403866000	11.899905000
C	8.295521000	12.036878000	12.378154000
H	9.289463000	12.421440000	12.132368000
H	8.318506000	10.950708000	12.240632000
H	8.130587000	12.222342000	13.445811000
C	8.990235000	12.361350000	9.292332000
H	9.389331000	13.342163000	9.570804000
H	9.467877000	11.626365000	9.950017000
C	9.307250000	12.059246000	7.833253000
H	8.751920000	12.719146000	7.160461000
H	9.050099000	11.029300000	7.565982000
H	10.376255000	12.182000000	7.629806000
C	4.069124000	15.809366000	10.336491000
H	3.608415000	16.548152000	9.672265000
H	3.524978000	15.849495000	11.285816000
C	5.549041000	16.111489000	10.511258000
H	5.967376000	15.541390000	11.347679000
H	5.740002000	17.171635000	10.710755000
C	3.234058000	13.100809000	11.001787000
H	3.205577000	12.057081000	10.669782000
H	4.054974000	13.160950000	11.726004000
C	1.909276000	13.472605000	11.664458000

H	1.057202000	13.294383000	11.002124000
H	1.877742000	14.524450000	11.967588000
H	1.748843000	12.872854000	12.567792000
C	2.204821000	14.425630000	8.643501000
H	1.789373000	13.446512000	8.382593000
H	1.510311000	14.885582000	9.355574000
C	2.340226000	15.288838000	7.394823000
H	3.105483000	14.890920000	6.721339000
H	2.626470000	16.316776000	7.641882000
H	1.390560000	15.344320000	6.851521000
C	8.220002000	15.961795000	9.457096000
H	8.206002000	16.949494000	9.932859000
H	8.509200000	15.248306000	10.236252000
C	9.216436000	15.937068000	8.301287000
H	9.005954000	16.721236000	7.568381000
H	9.195827000	14.981144000	7.770943000
H	10.237407000	16.096473000	8.664746000
C	6.078165000	16.899285000	7.739059000
H	6.643735000	16.645367000	6.837553000
H	5.026717000	16.763513000	7.467766000
C	6.352012000	18.341925000	8.153020000
H	6.097223000	19.023044000	7.334098000
H	7.405788000	18.510859000	8.394432000
H	5.757930000	18.644761000	9.021403000
C	2.976426000	13.897484000	2.955121000
H	3.370208000	13.301774000	3.783129000
H	2.506888000	13.200574000	2.249686000
C	1.966616000	14.908139000	3.473210000
H	1.449624000	15.436630000	2.667315000
H	2.450363000	15.645619000	4.119409000
H	1.204326000	14.383571000	4.058049000
C	3.894230000	15.325207000	1.204841000
H	3.180987000	16.111818000	1.477614000
H	4.859402000	15.809603000	1.029826000
C	3.419411000	14.556088000	-0.014652000
H	3.346282000	15.242313000	-0.864586000
H	2.431741000	14.105979000	0.127685000
H	4.125275000	13.764063000	-0.280209000

1*-Sm, S=3, small core

Sm	7.949596000	12.054914000	15.210201000
Fe	8.164363000	9.689855000	20.163447000
P	9.767309000	8.292222000	19.398656000
P	9.672879000	10.199978000	21.737737000
P	6.779658000	8.054559000	20.813391000
P	6.566437000	11.062297000	20.979915000
Si	4.573655000	11.873641000	14.460902000
Si	5.898771000	14.522967000	13.591043000
Si	10.112874000	11.191665000	12.608073000
Si	11.267257000	13.064179000	14.829769000
N	8.015371000	10.991725000	17.588741000
N	8.075623000	10.471704000	18.616460000
N	5.911386000	12.953939000	14.322489000
N	10.051331000	12.115798000	14.062183000
C	5.251646000	10.172988000	15.041904000
H	5.933223000	9.727045000	14.305928000
H	4.426150000	9.463770000	15.170685000
H	5.762780000	10.224520000	16.013582000
C	3.283514000	12.387201000	15.769131000
H	2.503721000	11.625274000	15.886058000
H	2.795426000	13.328097000	15.495212000
H	3.763134000	12.536331000	16.742894000
C	3.621361000	11.540063000	12.845630000
H	3.119762000	12.446203000	12.488585000
H	2.852732000	10.771646000	12.989481000
H	4.296647000	11.200816000	12.053296000
C	4.468683000	15.648137000	14.161706000
H	3.497900000	15.253972000	13.841104000
H	4.570203000	16.655336000	13.740810000
H	4.447466000	15.738673000	15.253001000

C	5.848606000	14.486017000	11.687633000
H	6.686186000	13.905143000	11.286721000
H	5.908243000	15.496866000	11.267451000
H	4.923816000	14.026242000	11.323788000
C	7.520664000	15.408551000	14.077114000
H	7.575704000	15.579511000	15.159842000
H	7.592731000	16.389874000	13.594782000
H	8.405757000	14.838327000	13.765638000
C	8.458736000	10.225939000	12.498380000
H	8.344318000	9.511357000	13.325959000
H	8.412344000	9.636100000	11.576179000
H	7.584788000	10.892240000	12.479817000
C	10.248412000	12.222285000	11.014384000
H	9.440208000	12.958685000	10.955538000
H	10.195398000	11.588300000	10.121643000
H	11.196381000	12.769711000	10.979265000
C	11.500350000	9.885294000	12.547339000
H	12.487893000	10.357983000	12.510399000
H	11.405177000	9.249555000	11.659262000
H	11.475161000	9.238985000	13.431195000
C	11.968585000	14.488252000	13.779316000
H	12.512380000	14.103368000	12.909743000
H	12.664232000	15.106110000	14.359185000
H	11.167573000	15.136022000	13.408686000
C	12.758656000	12.066369000	15.476202000
H	12.426578000	11.275596000	16.157433000
H	13.464279000	12.707306000	16.017747000
H	13.303087000	11.589336000	14.654597000
C	10.463071000	13.866689000	16.377931000
H	9.662606000	14.569220000	16.111440000
H	11.204584000	14.444887000	16.940588000
H	10.063850000	13.117076000	17.075807000
C	8.857617000	6.588787000	17.276834000
H	9.516515000	7.044928000	16.531785000
H	7.916534000	7.145405000	17.268571000
H	8.646110000	5.567637000	16.941876000
C	9.497628000	6.583592000	18.662797000
H	10.467729000	6.073575000	18.636341000
H	8.876019000	6.030435000	19.375183000
C	10.913695000	9.059280000	18.124194000
H	10.283141000	9.291502000	17.259719000
H	11.197813000	10.030734000	18.540880000
C	12.146342000	8.268166000	17.695507000
H	12.717976000	8.834046000	16.952373000
H	11.885556000	7.308369000	17.239088000
H	12.819743000	8.065356000	18.534455000
C	10.933555000	7.868441000	20.805108000
H	10.401563000	7.150393000	21.438806000
H	11.837860000	7.372167000	20.436130000
C	11.242244000	9.145276000	21.572209000
H	11.976150000	9.746495000	21.025365000
H	11.669759000	8.938919000	22.558855000
C	10.479342000	11.908287000	21.806653000
H	9.731771000	12.596898000	22.214294000
H	11.305566000	11.875921000	22.525895000
C	10.968769000	12.407358000	20.451885000
H	10.177928000	12.347567000	19.698260000
H	11.816113000	11.818682000	20.084348000
H	11.306978000	13.447228000	20.516003000
C	9.286431000	9.952664000	23.578505000
H	8.307933000	10.416174000	23.747754000
H	9.126056000	8.872456000	23.677323000
C	10.283575000	10.442112000	24.626658000
H	11.288134000	10.037384000	24.464384000
H	9.970402000	10.132090000	25.630266000
H	10.364635000	11.532841000	24.635192000
C	7.336963000	6.739033000	22.061385000
H	8.300526000	6.364894000	21.697349000
H	7.562102000	7.308677000	22.970736000
C	6.410689000	5.567484000	22.380640000

H	6.273928000	4.908396000	21.518543000
H	5.418274000	5.899151000	22.703680000
H	6.825868000	4.958556000	23.191970000
C	5.906833000	6.947963000	19.553892000
H	6.654209000	6.240250000	19.179993000
H	5.144933000	6.357643000	20.075494000
C	5.286847000	7.718126000	18.393966000
H	4.909204000	7.034609000	17.625847000
H	6.015929000	8.389270000	17.930627000
H	4.439654000	8.330794000	18.720507000
C	5.250368000	8.744543000	21.701459000
H	4.445744000	8.794994000	20.960352000
H	4.919924000	8.055251000	22.485319000
C	5.546988000	10.132207000	22.249625000
H	6.163319000	10.071811000	23.153404000
H	4.636853000	10.683298000	22.510797000
C	6.849977000	12.678390000	21.897058000
H	5.906641000	12.960776000	22.379025000
H	7.557342000	12.459291000	22.704253000
C	7.365864000	13.816643000	21.020239000
H	6.620835000	14.123228000	20.279821000
H	8.270082000	13.529431000	20.476727000
H	7.603843000	14.697718000	21.625834000
C	5.281474000	11.587040000	19.713872000
H	5.827377000	12.175481000	18.968993000
H	4.990591000	10.667252000	19.196795000
C	4.052414000	12.345825000	20.206667000
H	3.395498000	12.587650000	19.364348000
H	4.314720000	13.290306000	20.693289000
H	3.462145000	11.758588000	20.917367000

3, S=3, small core

Sm	5.255327000	17.944420000	19.118625000
Fe	6.873958000	12.583694000	18.859109000
Fe	4.450456000	19.492533000	13.771504000
P	8.846330000	12.974016000	17.810396000
P	6.542386000	11.122076000	17.195977000
P	7.796636000	11.704481000	20.699824000
P	4.897610000	12.165155000	19.898430000
P	6.368949000	20.689903000	13.851153000
P	3.535090000	21.442391000	13.164296000
P	2.512771000	18.346909000	13.568537000
P	5.110242000	18.248944000	12.042137000
Si	8.503353000	19.427555000	19.627976000
Si	6.084570000	21.215253000	20.249235000
Si	4.021535000	17.324018000	22.399882000
Si	1.824196000	17.564853000	20.283040000
N	6.432972000	14.276600000	18.786435000
N	6.124415000	15.384499000	18.762210000
N	4.781992000	18.621969000	16.522446000
N	4.647307000	18.972790000	15.436653000
N	6.807567000	19.743326000	19.689232000
N	3.481809000	17.516381000	20.764559000
C	10.786846000	14.541536000	19.263901000
H	10.799651000	15.356405000	18.535184000
H	11.752161000	14.550325000	19.782000000
H	10.012415000	14.778285000	19.997637000
C	10.546183000	13.193750000	18.591122000
H	10.663080000	12.372586000	19.306947000
H	11.291275000	13.023799000	17.804603000
C	9.972691000	14.603044000	15.655291000
H	9.831957000	15.504412000	15.048524000
H	10.074222000	13.760595000	14.963384000
H	10.925956000	14.716500000	16.180919000
C	8.800460000	14.423135000	16.615598000
H	7.858587000	14.313062000	16.067964000
H	8.672830000	15.313783000	17.239255000
C	9.239818000	11.528189000	16.679925000
H	10.064790000	11.767651000	15.999680000
H	9.577813000	10.713397000	17.331462000

C	7.965022000	11.146213000	15.947199000
H	8.050250000	10.180489000	15.437420000
H	7.728931000	11.895952000	15.185329000
C	6.500485000	9.248343000	17.517264000
H	7.531177000	9.020310000	17.815953000
H	5.890591000	9.094083000	18.410676000
C	6.049217000	8.306803000	16.402925000
H	6.603642000	8.466999000	15.471854000
H	4.984967000	8.424112000	16.177351000
H	6.205215000	7.261409000	16.694027000
C	5.084314000	11.321670000	16.008850000
H	5.126942000	10.543142000	15.239271000
H	4.175953000	11.143812000	16.594936000
C	5.024374000	12.705324000	15.368892000
H	4.068687000	12.858467000	14.855412000
H	5.815015000	12.840564000	14.623820000
H	5.142236000	13.491503000	16.121099000
C	9.328595000	9.500963000	21.942884000
H	9.793500000	8.525016000	21.760394000
H	8.533635000	9.348011000	22.680046000
H	10.089071000	10.138853000	22.402347000
C	8.791758000	10.094212000	20.641564000
H	8.119548000	9.384435000	20.145867000
H	9.613396000	10.269836000	19.936133000
C	8.923223000	12.728835000	21.820238000
H	9.136800000	12.144429000	22.722186000
H	9.873992000	12.855568000	21.293393000
C	8.337183000	14.085855000	22.191742000
H	8.022983000	14.637580000	21.300830000
H	9.067440000	14.696851000	22.733340000
H	7.460472000	13.983207000	22.838820000
C	6.465683000	11.227219000	21.961431000
H	6.224534000	10.169214000	21.801664000
H	6.854545000	11.319917000	22.981168000
C	5.234420000	12.095384000	21.741543000
H	5.419929000	13.127601000	22.054513000
H	4.363015000	11.734145000	22.298649000
C	3.958958000	10.540165000	19.676654000
H	3.819082000	10.389116000	18.601963000
H	4.658130000	9.761870000	20.002348000
C	2.625173000	10.381296000	20.403850000
H	2.231192000	9.369138000	20.257078000
H	1.870081000	11.080450000	20.033988000
H	2.719481000	10.536620000	21.482867000
C	3.505330000	13.409521000	19.821130000
H	2.783973000	13.152507000	20.604424000
H	3.926213000	14.383588000	20.089511000
C	2.825468000	13.487370000	18.457904000
H	3.531084000	13.806535000	17.686711000
H	2.003600000	14.209505000	18.481491000
H	2.405161000	12.523412000	18.149846000
C	8.783168000	17.860607000	18.565794000
H	8.335743000	16.969514000	19.022852000
H	9.855575000	17.663534000	18.455312000
H	8.362702000	17.978277000	17.559712000
C	9.323322000	19.069856000	21.313315000
H	9.258817000	19.939960000	21.975395000
H	10.384889000	18.822948000	21.190089000
H	8.836876000	18.232567000	21.823928000
C	9.581912000	20.783674000	18.823966000
H	10.609380000	20.424114000	18.690084000
H	9.628808000	21.683397000	19.446507000
H	9.195844000	21.078255000	17.843164000
C	6.401800000	21.598784000	22.090036000
H	6.124402000	20.753890000	22.728033000
H	5.827641000	22.473664000	22.417398000
H	7.461474000	21.813862000	22.268212000
C	6.591265000	22.795252000	19.302802000
H	7.660349000	23.011135000	19.392679000
H	6.042324000	23.661250000	19.691998000

H	6.352520000	22.699457000	18.238844000
C	4.198174000	21.082305000	20.001275000
H	3.947120000	20.991800000	18.935833000
H	3.694073000	21.981800000	20.372916000
H	3.769307000	20.224689000	20.532994000
C	5.927430000	17.249153000	22.379580000
H	6.284046000	16.393738000	21.791328000
H	6.325873000	17.130145000	23.393551000
H	6.368531000	18.163059000	21.963234000
C	3.445623000	15.720076000	23.266450000
H	2.368791000	15.727252000	23.463026000
H	3.956894000	15.604591000	24.229950000
H	3.664138000	14.835545000	22.659763000
C	3.524963000	18.736383000	23.581056000
H	3.819113000	19.712420000	23.182843000
H	3.999658000	18.613938000	24.561943000
H	2.441022000	18.756227000	23.739579000
C	0.864092000	19.137424000	20.778516000
H	0.800428000	19.237713000	21.867151000
H	-0.160209000	19.106084000	20.387361000
H	1.346999000	20.040456000	20.392245000
C	1.757576000	17.500864000	18.372565000
H	2.227408000	18.380616000	17.915700000
H	0.717254000	17.474399000	18.028651000
H	2.257091000	16.604483000	17.986505000
C	0.740460000	16.112825000	20.891991000
H	1.215243000	15.144216000	20.707794000
H	-0.231558000	16.118755000	20.383988000
H	0.547248000	16.185952000	21.967498000
C	8.713455000	19.100238000	14.317063000
H	9.666163000	18.726633000	13.925460000
H	8.061446000	18.239948000	14.492653000
H	8.918779000	19.559109000	15.288478000
C	8.081637000	20.095947000	13.348450000
H	7.981946000	19.657420000	12.349796000
H	8.720982000	20.979273000	13.236078000
C	6.739238000	21.499409000	15.503487000
H	5.792995000	21.950238000	15.819268000
H	6.917657000	20.687026000	16.216109000
C	7.870599000	22.523190000	15.547192000
H	7.679181000	23.378001000	14.890433000
H	8.833482000	22.090751000	15.257764000
H	7.985347000	22.915407000	16.562211000
C	6.196806000	22.174405000	12.716560000
H	7.024017000	22.879384000	12.852819000
H	6.255580000	21.792134000	11.691846000
C	4.839721000	22.806357000	12.980565000
H	4.550357000	23.510431000	12.193481000
H	4.858188000	23.366345000	13.921548000
C	2.751019000	22.428146000	15.733433000
H	3.633590000	23.063856000	15.861430000
H	3.006573000	21.435418000	16.116047000
H	1.959938000	22.849146000	16.363447000
C	2.310865000	22.360303000	14.275170000
H	2.157887000	23.367550000	13.870903000
H	1.351870000	21.838008000	14.194415000
C	2.653592000	21.616822000	11.491034000
H	3.445262000	21.431093000	10.755014000
H	1.968529000	20.765569000	11.412074000
C	1.912464000	22.911958000	11.165703000
H	1.551270000	22.900888000	10.130686000
H	2.552038000	23.794390000	11.273308000
H	1.041416000	23.055986000	11.811559000
C	0.771080000	19.031520000	13.767615000
H	0.718835000	19.930947000	13.144712000
H	0.076551000	18.308243000	13.323663000
C	0.367593000	19.346241000	15.204636000
H	-0.615020000	19.829737000	15.236257000
H	1.084636000	20.013127000	15.690746000
H	0.306885000	18.440260000	15.813241000

C	2.417133000	16.792105000	14.617081000
H	3.380101000	16.290618000	14.476239000
H	2.418166000	17.140359000	15.654593000
C	1.259292000	15.827751000	14.376896000
H	1.267661000	15.413618000	13.363351000
H	0.285786000	16.302544000	14.533358000
H	1.319145000	14.982822000	15.071822000
C	2.377727000	17.673784000	11.821288000
H	1.569351000	16.938523000	11.740129000
H	2.111205000	18.524318000	11.184092000
C	3.729359000	17.103100000	11.424377000
H	3.811963000	16.948558000	10.343452000
H	3.883393000	16.128771000	11.900506000
C	5.638255000	19.043647000	10.399531000
H	4.736295000	19.561591000	10.051966000
H	6.353383000	19.832193000	10.658812000
C	6.213350000	18.166268000	9.289496000
H	6.368404000	18.751602000	8.375614000
H	5.548067000	17.335927000	9.029858000
H	7.180920000	17.738519000	9.568021000
C	6.483752000	16.960537000	12.222729000
H	6.503882000	16.337506000	11.321119000
H	7.433065000	17.505351000	12.253380000
C	6.334989000	16.096664000	13.469324000
H	5.451587000	15.451753000	13.411514000
H	6.229913000	16.711763000	14.367987000
H	7.203488000	15.440696000	13.596144000

1-U, S=3/2, small core

U	8.468519000	15.065209000	2.410495000
Fe	8.994482000	13.697899000	-2.830795000
P	11.251060000	13.920303000	-2.656165000
P	9.517329000	11.715948000	-3.801507000
P	8.684965000	15.342043000	-4.356002000
P	6.739719000	13.412462000	-2.999705000
Si	9.369490000	11.748978000	3.076280000
Si	10.994610000	13.702343000	4.710935000
Si	10.334485000	18.045767000	1.938802000
Si	8.792817000	18.025642000	4.529374000
Si	5.221975000	16.173216000	1.850554000
Si	5.270439000	14.083677000	4.020219000
N	8.704553000	14.405403000	-0.025305000
N	8.815597000	14.091957000	-1.140903000
N	9.722815000	13.419568000	3.511365000
N	9.251940000	17.194533000	3.035929000
N	6.161723000	15.045964000	2.829783000
C	8.865524000	10.637660000	4.538568000
H	7.923608000	10.962921000	4.988559000
H	9.623093000	10.621000000	5.327880000
H	8.732086000	9.607021000	4.188434000
C	7.927621000	11.706863000	1.835229000
H	8.246832000	12.044860000	0.845377000
H	7.066522000	12.311806000	2.137850000
H	7.575077000	10.673040000	1.739142000
C	10.794461000	10.852215000	2.181568000
H	11.592401000	10.542626000	2.860650000
H	11.234975000	11.492186000	1.410790000
H	10.406906000	9.951186000	1.691194000
C	12.064089000	15.192844000	4.229962000
H	11.451534000	16.072350000	4.016766000
H	12.658088000	14.960078000	3.338894000
H	12.760083000	15.443198000	5.039020000
C	12.252150000	12.283139000	4.928117000
H	12.836107000	12.114356000	4.018283000
H	11.807343000	11.331269000	5.231686000
H	12.953719000	12.581099000	5.716873000
C	10.257223000	14.004971000	6.436408000
H	9.667088000	13.142813000	6.763900000
H	9.600140000	14.879489000	6.452424000
H	11.050658000	14.171101000	7.174298000

C	11.793007000	18.949424000	2.762905000
H	12.377136000	18.287019000	3.407923000
H	11.463876000	19.805128000	3.360527000
H	12.459685000	19.334468000	1.981890000
C	11.127229000	16.790134000	0.743389000
H	10.392084000	16.251434000	0.135215000
H	11.732573000	16.059725000	1.290418000
H	11.790181000	17.326160000	0.053792000
C	9.471602000	19.353476000	0.856416000
H	9.008485000	20.135835000	1.464517000
H	8.691119000	18.915281000	0.227717000
H	10.203212000	19.834127000	0.195594000
C	10.204027000	18.202351000	5.796275000
H	11.024665000	18.831521000	5.443069000
H	10.621249000	17.228731000	6.070035000
H	9.804383000	18.659528000	6.709693000
C	7.445647000	17.053839000	5.441034000
H	7.806074000	16.068744000	5.754759000
H	6.550549000	16.904827000	4.834252000
H	7.165646000	17.598124000	6.350557000
C	8.123711000	19.784284000	4.228686000
H	7.314650000	19.793540000	3.491698000
H	8.906213000	20.465077000	3.877039000
H	7.730231000	20.196679000	5.165080000
C	6.288016000	16.859412000	0.427901000
H	6.725236000	16.082459000	-0.206731000
H	7.096706000	17.493096000	0.807646000
H	5.655469000	17.493687000	-0.205543000
C	3.688260000	15.383258000	1.038930000
H	2.935232000	15.094335000	1.778803000
H	3.949575000	14.491380000	0.461836000
H	3.218537000	16.103118000	0.358027000
C	4.602586000	17.723387000	2.763012000
H	5.433093000	18.282709000	3.203482000
H	3.892392000	17.491159000	3.560304000
H	4.097575000	18.385800000	2.049473000
C	4.417587000	12.556010000	3.259754000
H	5.132054000	11.820190000	2.880279000
H	3.751487000	12.834661000	2.437805000
H	3.810617000	12.060065000	4.026790000
C	3.875893000	15.032339000	4.910360000
H	3.058216000	15.327306000	4.245447000
H	4.250911000	15.929975000	5.411431000
H	3.449672000	14.377883000	5.680412000
C	6.415598000	13.470448000	5.405623000
H	6.524315000	14.234775000	6.180841000
H	7.417130000	13.212573000	5.049148000
H	5.984178000	12.580131000	5.877909000
C	13.280908000	12.694082000	-0.903321000
H	13.521105000	12.463530000	0.140334000
H	14.095105000	13.306278000	-1.298192000
H	13.286309000	11.744812000	-1.448194000
C	11.922591000	13.389332000	-0.977893000
H	11.916249000	14.294552000	-0.361402000
H	11.146277000	12.741577000	-0.561653000
C	12.134457000	15.543888000	-2.987106000
H	11.914340000	15.776192000	-4.035740000
H	11.608323000	16.290807000	-2.383850000
C	13.637745000	15.631024000	-2.735073000
H	13.876389000	15.508853000	-1.675347000
H	14.014553000	16.613983000	-3.039653000
H	14.198393000	14.879209000	-3.299165000
C	12.098228000	12.761603000	-3.857860000
H	11.986503000	13.210929000	-4.851770000
H	13.169709000	12.674798000	-3.650867000
C	11.383745000	11.420821000	-3.782016000
H	11.622612000	10.918485000	-2.839385000
H	11.678970000	10.747002000	-4.593470000
C	9.290911000	9.909793000	-1.595944000
H	9.003034000	10.788667000	-1.012175000

H	10.365809000	9.760258000	-1.454664000
H	8.786655000	9.036060000	-1.169494000
C	8.929706000	10.079752000	-3.067888000
H	7.841381000	10.053295000	-3.191763000
H	9.338830000	9.254609000	-3.660705000
C	9.207523000	11.422704000	-5.651594000
H	8.199805000	11.787429000	-5.866138000
H	9.891549000	12.126679000	-6.140676000
C	9.389979000	10.019063000	-6.224671000
H	8.627800000	9.326323000	-5.856136000
H	10.369375000	9.593215000	-5.982116000
H	9.309633000	10.037678000	-7.317628000
C	9.325601000	15.220180000	-6.131998000
H	10.412406000	15.089713000	-6.053158000
H	8.935300000	14.263345000	-6.496306000
C	8.995796000	16.339111000	-7.118291000
H	9.472604000	17.284022000	-6.842978000
H	7.917860000	16.517915000	-7.187445000
H	9.346736000	16.081686000	-8.124252000
C	9.159974000	17.128951000	-3.973747000
H	10.249681000	17.198513000	-4.034487000
H	8.757596000	17.761713000	-4.772614000
C	8.668589000	17.612629000	-2.615388000
H	8.996635000	16.947739000	-1.812074000
H	7.575786000	17.657653000	-2.574773000
H	9.041761000	18.619129000	-2.399620000
C	6.831582000	15.593744000	-4.674387000
H	6.624105000	16.649019000	-4.880954000
H	6.568845000	15.030608000	-5.577948000
C	6.038933000	15.082477000	-3.478381000
H	4.964056000	15.026666000	-3.681791000
H	6.178083000	15.730301000	-2.607149000
C	5.960296000	12.322788000	-4.326236000
H	6.404965000	12.649835000	-5.271997000
H	6.327747000	11.305796000	-4.155612000
C	4.436987000	12.321905000	-4.439086000
H	3.953675000	11.987459000	-3.516925000
H	4.116732000	11.644367000	-5.238526000
H	4.041725000	13.312906000	-4.680468000
C	5.708929000	12.981358000	-1.498626000
H	6.116319000	13.572350000	-0.672992000
H	4.689101000	13.339995000	-1.677831000
C	5.705156000	11.500179000	-1.131891000
H	5.133548000	11.338385000	-0.212888000
H	6.717805000	11.131480000	-0.954175000
H	5.251630000	10.882871000	-1.914388000

2, S=3/2, small core

U	9.455708000	10.388735000	9.435439000
Fe	14.624482000	8.810847000	9.705612000
P	14.337954000	6.801801000	8.674270000
P	15.882688000	9.343305000	7.930955000
P	16.073258000	7.990356000	11.260093000
P	14.974737000	10.771965000	10.794547000
O	8.304329000	9.235858000	10.895579000
O	8.458828000	10.174638000	7.476488000
O	9.861602000	12.497440000	9.990810000
N	11.800531000	9.474603000	9.520546000
N	12.915459000	9.157552000	9.640937000
C	12.625891000	6.455728000	8.000463000
H	11.955100000	6.427642000	8.865046000
H	12.349108000	7.354071000	7.440696000
C	12.461133000	5.206474000	7.138204000
H	13.107479000	5.224636000	6.254935000
H	11.429618000	5.125357000	6.779680000
H	12.681723000	4.287801000	7.689724000
C	14.729361000	5.125842000	9.449683000
H	14.829719000	4.399946000	8.634814000
H	15.717433000	5.208130000	9.913603000
C	13.696110000	4.639690000	10.463004000

H	12.746257000	4.390532000	9.982305000
H	13.484331000	5.393988000	11.224610000
H	14.048756000	3.736900000	10.973919000
C	15.376631000	6.713303000	7.086192000
H	15.787587000	5.705689000	6.963637000
H	14.678714000	6.873454000	6.259043000
C	16.468250000	7.776198000	7.061784000
H	16.788275000	7.992477000	6.036677000
H	17.351322000	7.441505000	7.617362000
C	17.517130000	10.289638000	8.044379000
H	17.271376000	11.268942000	8.470890000
H	18.099508000	9.762907000	8.808687000
C	18.357252000	10.467676000	6.780429000
H	17.838845000	11.058618000	6.020292000
H	18.629495000	9.508099000	6.330567000
H	19.290786000	10.992201000	7.013969000
C	15.088254000	10.305074000	6.512687000
H	14.985145000	11.335876000	6.871861000
H	15.788218000	10.331374000	5.669854000
C	15.622066000	6.751581000	12.612812000
H	15.548191000	5.766380000	12.142538000
H	16.458695000	6.702806000	13.318770000
C	14.327059000	7.089683000	13.339586000
H	13.506955000	7.243597000	12.631583000
H	14.419488000	8.005101000	13.933505000
H	14.043391000	6.287408000	14.029228000
C	17.709350000	7.233844000	10.665859000
H	17.452917000	6.530261000	9.866423000
H	18.228019000	8.065109000	10.174340000
C	18.632898000	6.561267000	11.679198000
H	19.577852000	6.273405000	11.204512000
H	18.189434000	5.652591000	12.096122000
H	18.882256000	7.221955000	12.516149000
C	16.733010000	9.370148000	12.375104000
H	16.115911000	9.369754000	13.279473000
H	17.758353000	9.147158000	12.688835000
C	16.628989000	10.712701000	11.669597000
H	17.400263000	10.811139000	10.899248000
H	16.745325000	11.552784000	12.361291000
C	14.977324000	12.438044000	9.919954000
H	15.144108000	12.231111000	8.859193000
H	13.952065000	12.815652000	10.000951000
C	15.964134000	13.498036000	10.408237000
H	15.837223000	14.420421000	9.831141000
H	17.004802000	13.179988000	10.288419000
H	15.810889000	13.750824000	11.460195000
C	13.766069000	11.047744000	12.198327000
H	12.781950000	11.081506000	11.719471000
H	13.780616000	10.116083000	12.773334000
C	13.955921000	12.259893000	13.105056000
H	13.811368000	13.199225000	12.565064000
H	14.944057000	12.276854000	13.577716000
H	13.212934000	12.242549000	13.909461000
C	7.587116000	8.573112000	11.832462000
C	8.254890000	7.730800000	12.773685000
C	7.490958000	7.093934000	13.756849000
H	7.980183000	6.459498000	14.485951000
C	6.115404000	7.243253000	13.835032000
H	5.548635000	6.738823000	14.612799000
C	5.472750000	8.035785000	12.896770000
H	4.395487000	8.132096000	12.957365000
C	6.165342000	8.706265000	11.883955000
C	9.773306000	7.481172000	12.738076000
C	10.545122000	8.791358000	12.969832000
H	10.300719000	9.547720000	12.223780000
H	11.624834000	8.612882000	12.929425000
H	10.301934000	9.206954000	13.953446000
C	10.166356000	6.835580000	11.396704000
H	9.700253000	5.848777000	11.300024000
H	11.253930000	6.711267000	11.337264000

H	9.845419000	7.439883000	10.548649000
C	10.235873000	6.508085000	13.837848000
H	10.042991000	6.896371000	14.843569000
H	11.316843000	6.357937000	13.747136000
H	9.759810000	5.525909000	13.748898000
C	5.366003000	9.542467000	10.868940000
C	5.576131000	8.988519000	9.449382000
H	5.186574000	7.967063000	9.381434000
H	6.629297000	8.958504000	9.172866000
H	5.049908000	9.599044000	8.707894000
C	3.849262000	9.503355000	11.130745000
H	3.344366000	10.104883000	10.367203000
H	3.582490000	9.922001000	12.107297000
H	3.444451000	8.487943000	11.065949000
C	5.777194000	11.020068000	10.963172000
H	6.840627000	11.166436000	10.766447000
H	5.580528000	11.406860000	11.968553000
H	5.209109000	11.626691000	10.250116000
C	7.481278000	9.828318000	6.606746000
C	7.381340000	8.482580000	6.140550000
C	6.233574000	8.108636000	5.434077000
H	6.117119000	7.081262000	5.109746000
C	5.231220000	9.015149000	5.125150000
H	4.338072000	8.693378000	4.596972000
C	5.419818000	10.352365000	5.443080000
H	4.670688000	11.067102000	5.123879000
C	6.544038000	10.801889000	6.143254000
C	8.512369000	7.454205000	6.326305000
C	9.833674000	8.057141000	5.809776000
H	9.736678000	8.326277000	4.752473000
H	10.645301000	7.326097000	5.894840000
H	10.111899000	8.958071000	6.358993000
C	8.636735000	7.009959000	7.791879000
H	8.814421000	7.850966000	8.466198000
H	9.460075000	6.296987000	7.916011000
H	7.715348000	6.526021000	8.130691000
C	8.272167000	6.174008000	5.504066000
H	7.396062000	5.614444000	5.847655000
H	9.138690000	5.511754000	5.610937000
H	8.148976000	6.389000000	4.437589000
C	6.781054000	12.317922000	6.277883000
C	6.777829000	12.795372000	7.738190000
H	5.836047000	12.532768000	8.228499000
H	6.889153000	13.884978000	7.784539000
H	7.595885000	12.362136000	8.315403000
C	8.121051000	12.650603000	5.594468000
H	8.951818000	12.127996000	6.072199000
H	8.315996000	13.727354000	5.636872000
H	8.090817000	12.352531000	4.540791000
C	5.700418000	13.143487000	5.555360000
H	5.638085000	12.900407000	4.489681000
H	5.953211000	14.206157000	5.636628000
H	4.707927000	13.010174000	5.999595000
C	10.547293000	13.544143000	10.488651000
C	11.545144000	14.193045000	9.693631000
C	12.384216000	15.123315000	10.319539000
H	13.169792000	15.603367000	9.747980000
C	12.233284000	15.477301000	11.653204000
H	12.907265000	16.194033000	12.114442000
C	11.158272000	14.960362000	12.362661000
H	10.995977000	15.307465000	13.376264000
C	10.277911000	14.025699000	11.808566000
C	9.012327000	13.631881000	12.595319000
C	9.023623000	12.152147000	13.007762000
H	9.018965000	11.480190000	12.149768000
H	8.139077000	11.913565000	13.609192000
H	9.910334000	11.926102000	13.609452000
C	7.776252000	13.954365000	11.732762000
H	7.727274000	15.029585000	11.528604000
H	6.858173000	13.667700000	12.257539000

H	7.804660000	13.430388000	10.776712000
C	8.861294000	14.442416000	13.895824000
H	9.659247000	14.230002000	14.616045000
H	7.914042000	14.170167000	14.373380000
H	8.839445000	15.521527000	13.710905000
C	11.615078000	14.050228000	8.158155000
C	10.332323000	14.684802000	7.587359000
H	9.444474000	14.172342000	7.963201000
H	10.326802000	14.626300000	6.493005000
H	10.267287000	15.739557000	7.874305000
C	12.804685000	14.820194000	7.553689000
H	12.755299000	15.893394000	7.760983000
H	12.787362000	14.702332000	6.465084000
H	13.769616000	14.443674000	7.911658000
C	11.737382000	12.599217000	7.656524000
H	12.485472000	12.029925000	8.215185000
H	12.026507000	12.590583000	6.599381000
H	10.781916000	12.073186000	7.716779000
C	13.728749000	9.783040000	6.061390000
H	13.283017000	10.457074000	5.322262000
H	13.036333000	9.703535000	6.903376000
H	13.806701000	8.798891000	5.589378000

N2U(HMDS)3, S=3/2, small core

U	8.504228000	15.162031000	2.540278000
Si	9.239992000	11.791347000	3.021704000
Si	11.021034000	13.678549000	4.632706000
Si	10.160208000	18.156974000	2.044464000
Si	8.794799000	18.057009000	4.750469000
Si	5.402730000	15.995582000	1.676101000
Si	5.278643000	14.171214000	4.115093000
N	9.152737000	14.221818000	0.363237000
N	9.479139000	13.762640000	-0.606699000
N	9.658881000	13.451385000	3.506373000
N	9.196126000	17.251434000	3.219472000
N	6.247257000	15.021576000	2.886629000
C	8.820374000	10.677606000	4.503085000
H	7.934790000	11.036813000	5.035363000
H	9.640118000	10.610693000	5.224309000
H	8.609294000	9.661397000	4.149931000
C	7.711385000	11.783566000	1.875862000
H	8.002651000	11.810667000	0.822258000
H	6.986150000	12.584792000	2.053765000
H	7.178164000	10.838612000	2.034614000
C	10.600403000	10.936809000	2.005013000
H	11.451466000	10.616595000	2.609770000
H	10.974549000	11.593349000	1.212719000
H	10.179125000	10.045529000	1.524921000
C	12.042829000	15.187556000	4.111417000
H	11.427570000	16.086954000	4.022180000
H	12.531147000	15.002186000	3.148063000
H	12.829216000	15.384897000	4.849037000
C	12.274402000	12.249848000	4.712987000
H	12.790568000	12.109411000	3.758976000
H	11.843759000	11.291866000	5.016576000
H	13.031238000	12.518917000	5.459575000
C	10.371223000	13.935966000	6.397048000
H	9.807003000	13.061002000	6.735551000
H	9.707203000	14.803215000	6.463992000
H	11.198940000	14.094803000	7.097492000
C	11.704778000	18.989916000	2.767140000
H	12.347226000	18.275387000	3.289565000
H	11.449170000	19.793247000	3.464951000
H	12.288000000	19.438140000	1.954216000
C	10.779394000	16.953470000	0.697041000
H	10.004754000	16.636407000	-0.009003000
H	11.288008000	16.066120000	1.091492000
H	11.522699000	17.495323000	0.100156000
C	9.180638000	19.498229000	1.120575000
H	8.918451000	20.334439000	1.774146000

H	8.250956000	19.106516000	0.696347000
H	9.783034000	19.896987000	0.295868000
C	10.259295000	18.150696000	5.958781000
H	11.079581000	18.769106000	5.584504000
H	10.662865000	17.158618000	6.182059000
H	9.917789000	18.589551000	6.903850000
C	7.439490000	17.077308000	5.634439000
H	7.786114000	16.081150000	5.926847000
H	6.549280000	16.959999000	5.011343000
H	7.150659000	17.598214000	6.554297000
C	8.156172000	19.833880000	4.524442000
H	7.313762000	19.884096000	3.828016000
H	8.935562000	20.512924000	4.164273000
H	7.813835000	20.217430000	5.492656000
C	6.596689000	16.339634000	0.215011000
H	6.838664000	15.440871000	-0.360583000
H	7.523925000	16.871385000	0.469742000
H	6.066886000	17.016778000	-0.465560000
C	3.910486000	15.132091000	0.882552000
H	3.096980000	14.970310000	1.596179000
H	4.186786000	14.162934000	0.455947000
H	3.516149000	15.757026000	0.072866000
C	4.839279000	17.693675000	2.305138000
H	5.688687000	18.276350000	2.674368000
H	4.112848000	17.605098000	3.117753000
H	4.367535000	18.261952000	1.495145000
C	4.418279000	12.613600000	3.443703000
H	5.129498000	11.829204000	3.172862000
H	3.798088000	12.823132000	2.568331000
H	3.763600000	12.210091000	4.225457000
C	3.891010000	15.254842000	4.838227000
H	3.125299000	15.507933000	4.097713000
H	4.274025000	16.186939000	5.264189000
H	3.393336000	14.702306000	5.643940000
C	6.372594000	13.620402000	5.566719000
H	6.438381000	14.399217000	6.330970000
H	7.389386000	13.361691000	5.254756000
H	5.934597000	12.732737000	6.037060000

N2U(OAr)3, S=3/2, small core

U	9.197775000	10.442773000	9.196825000
O	8.327090000	9.267835000	10.769730000
O	8.154436000	10.265439000	7.307424000
O	9.566440000	12.439580000	9.941690000
N	10.969386000	8.777169000	8.618373000
N	11.795106000	8.073262000	8.354135000
C	7.867327000	8.788776000	11.954995000
C	8.796623000	8.327031000	12.932108000
C	8.296296000	7.943604000	14.180132000
H	8.979791000	7.606271000	14.949929000
C	6.940026000	7.973599000	14.468286000
H	6.580834000	7.677163000	15.449710000
C	6.044720000	8.356398000	13.480552000
H	4.986233000	8.338681000	13.710132000
C	6.466287000	8.758407000	12.208890000
C	10.298310000	8.149094000	12.634617000
C	10.988278000	9.480371000	12.281638000
H	10.492579000	10.014027000	11.465659000
H	12.033451000	9.309236000	11.999507000
H	10.976099000	10.164553000	13.135527000
C	10.448855000	7.112842000	11.502692000
H	10.077621000	6.139291000	11.838775000
H	11.502070000	6.992454000	11.222063000
H	9.877975000	7.391646000	10.615923000
C	11.077120000	7.593607000	13.841168000
H	11.039778000	8.266038000	14.704614000
H	12.129873000	7.475048000	13.562576000
H	10.709344000	6.610467000	14.150680000
C	5.413267000	9.127191000	11.145689000
C	5.593251000	8.254124000	9.889944000

H	5.454786000	7.196377000	10.138636000
H	6.584139000	8.366649000	9.452415000
H	4.851893000	8.523527000	9.129351000
C	3.974937000	8.885959000	11.639002000
H	3.277293000	9.132967000	10.831718000
H	3.714819000	9.514467000	12.497259000
H	3.805234000	7.839087000	11.911156000
C	5.502701000	10.619962000	10.789168000
H	6.499404000	10.903846000	10.444189000
H	5.278078000	11.241795000	11.661257000
H	4.787816000	10.869112000	9.997775000
C	7.256424000	10.036361000	6.311350000
C	7.195586000	8.752538000	5.692257000
C	6.148531000	8.507839000	4.797616000
H	6.057422000	7.531396000	4.337420000
C	5.217200000	9.480687000	4.467833000
H	4.404860000	9.255149000	3.782791000
C	5.367092000	10.758969000	4.983507000
H	4.671522000	11.527019000	4.667941000
C	6.388282000	11.082612000	5.882589000
C	8.266154000	7.664364000	5.904946000
C	9.647046000	8.242325000	5.533272000
H	9.669567000	8.500245000	4.469266000
H	10.436681000	7.504235000	5.716772000
H	9.877642000	9.147950000	6.096482000
C	8.245172000	7.115999000	7.341791000
H	8.409160000	7.891836000	8.092027000
H	9.021479000	6.353133000	7.473588000
H	7.277403000	6.654683000	7.561242000
C	8.054016000	6.446493000	4.986210000
H	7.121562000	5.915472000	5.204705000
H	8.874558000	5.737691000	5.141926000
H	8.055467000	6.724063000	3.927266000
C	6.571399000	12.551278000	6.310234000
C	6.300686000	12.742255000	7.809498000
H	5.270966000	12.466152000	8.056325000
H	6.454079000	13.787146000	8.100268000
H	6.958767000	12.128906000	8.427662000
C	7.995463000	13.010070000	5.944177000
H	8.755833000	12.385482000	6.415482000
H	8.155782000	14.048085000	6.257050000
H	8.144437000	12.956890000	4.860469000
C	5.606099000	13.500449000	5.576562000
H	5.724099000	13.447828000	4.489469000
H	5.820252000	14.530154000	5.882300000
H	4.557591000	13.300490000	5.821526000
C	10.323593000	13.357582000	10.598796000
C	11.601929000	13.707666000	10.077631000
C	12.411933000	14.564724000	10.829038000
H	13.399384000	14.825582000	10.468203000
C	11.985170000	15.102695000	12.033392000
H	12.635797000	15.760343000	12.602725000
C	10.706495000	14.815041000	12.487409000
H	10.373736000	15.273973000	13.410451000
C	9.841599000	13.959380000	11.797415000
C	8.422563000	13.723461000	12.348016000
C	8.269689000	12.270929000	12.829365000
H	8.432200000	11.548738000	12.029589000
H	7.264883000	12.100631000	13.231107000
H	8.987966000	12.053355000	13.626592000
C	7.375989000	14.064373000	11.270608000
H	7.440490000	15.124027000	10.999373000
H	6.365088000	13.875314000	11.649692000
H	7.520837000	13.477034000	10.364902000
C	8.109404000	14.622382000	13.557982000
H	8.748694000	14.403054000	14.419735000
H	7.074861000	14.444934000	13.870211000
H	8.203622000	15.686603000	13.317213000
C	12.072459000	13.264918000	8.680263000
C	11.226690000	14.011470000	7.629910000

H	10.162140000	13.819147000	7.779369000
H	11.496624000	13.699201000	6.613543000
H	11.390369000	15.090635000	7.708544000
C	13.549616000	13.616339000	8.422910000
H	13.726736000	14.694843000	8.456183000
H	13.830660000	13.276704000	7.420440000
H	14.219827000	13.133267000	9.142288000
C	11.941126000	11.737398000	8.459421000
H	11.977127000	11.185296000	9.406729000
H	12.755074000	11.347721000	7.839181000
H	11.027466000	11.507387000	7.885848000

A, S=0, small core

Fe	11.747085000	0.497067000	13.999779000
P	10.555561000	-0.631941000	15.512589000
P	12.218418000	-1.532908000	13.130311000
P	10.868599000	1.342669000	12.139357000
P	11.392605000	2.551816000	14.878953000
C	10.570605000	-2.489787000	15.148117000
C	11.853617000	-2.853666000	14.418580000
C	10.294302000	3.128463000	12.395917000
C	11.133762000	3.784183000	13.479684000
C	8.698221000	-0.407570000	15.848035000
C	7.962728000	-1.458049000	16.679206000
C	11.161588000	-0.620957000	17.309572000
C	12.583838000	-1.142143000	17.490747000
C	13.998291000	-1.985305000	12.736233000
C	14.586736000	-1.275533000	11.521503000
C	11.312018000	-2.209994000	11.619992000
C	11.651754000	-3.617734000	11.136361000
C	11.993733000	1.573980000	10.627575000
C	13.212629000	2.456525000	10.874589000
C	9.365750000	0.742098000	11.140712000
C	8.174162000	0.311267000	11.983288000
C	12.791639000	3.417400000	15.784944000
C	13.125641000	2.830223000	17.152153000
C	9.922491000	2.949580000	15.995399000
C	9.771419000	4.375949000	16.518925000
N	13.478418000	0.726179000	14.284176000
N	14.592770000	0.870907000	14.464783000
H	9.700277000	-2.691789000	14.511667000
H	10.441584000	-3.069059000	16.069241000
H	12.708807000	-2.837183000	15.102333000
H	11.812427000	-3.852906000	13.971159000
H	9.242309000	3.081313000	12.701367000
H	10.333709000	3.678432000	11.447886000
H	12.136652000	4.018157000	13.107558000
H	10.694423000	4.718928000	13.845026000
H	8.243807000	-0.321856000	14.855982000
H	8.590377000	0.576979000	16.313534000
H	6.904558000	-1.190753000	16.785623000
H	7.999368000	-2.448532000	16.215564000
H	8.371875000	-1.551171000	17.689995000
H	10.466778000	-1.186931000	17.940950000
H	11.107644000	0.425614000	17.631197000
H	12.945647000	-0.953608000	18.507999000
H	12.638067000	-2.223657000	17.326530000
H	13.273569000	-0.661594000	16.789972000
H	14.056679000	-3.073973000	12.621431000
H	14.569413000	-1.724702000	13.633040000
H	15.634649000	-1.560668000	11.377146000
H	14.050680000	-1.526878000	10.599597000
H	14.553896000	-0.190379000	11.647640000
H	11.489589000	-1.488193000	10.815907000
H	10.246879000	-2.131827000	11.864996000
H	11.034966000	-3.883324000	10.270034000
H	12.697859000	-3.703376000	10.827533000
H	11.469405000	-4.375802000	11.904221000
H	11.395042000	1.965447000	9.794520000
H	12.318931000	0.566967000	10.341436000

H	13.907700000	2.415501000	10.028174000
H	12.928278000	3.506031000	11.005312000
H	13.752783000	2.141457000	11.772905000
H	9.705173000	-0.089944000	10.514781000
H	9.085178000	1.553191000	10.456354000
H	7.334152000	-0.004375000	11.353592000
H	8.443751000	-0.523712000	12.636958000
H	7.815843000	1.122402000	12.626956000
H	12.540801000	4.481518000	15.864483000
H	13.660037000	3.339150000	15.123212000
H	13.971012000	3.359835000	17.605050000
H	12.282848000	2.905335000	17.848229000
H	13.402093000	1.776229000	17.068169000
H	9.981247000	2.250853000	16.836509000
H	9.038680000	2.655470000	15.418126000
H	8.857052000	4.468227000	17.116377000
H	10.608280000	4.667530000	17.160377000
H	9.701810000	5.110376000	15.710602000

1-Ce, S=1/2, small core+solv+disp

Ce	2.202497000	0.026262000	-0.100887000
Fe	-3.178073000	-0.137835000	0.111659000
P	-3.315865000	-2.349934000	0.268257000
P	-4.529431000	-0.391162000	-1.592146000
P	-3.117501000	2.075890000	-0.050263000
P	-4.172421000	0.218218000	2.019699000
Si	2.522004000	-1.672736000	2.946433000
Si	5.020386000	-0.251552000	2.078474000
Si	3.829862000	-2.726800000	-1.824907000
Si	1.881130000	-1.085564000	-3.383365000
Si	3.365400000	3.166604000	-1.450613000
Si	1.944938000	3.243522000	1.194805000
N	-0.325575000	-0.211945000	-0.174752000
N	-1.477116000	-0.193737000	-0.053351000
N	3.364865000	-0.678126000	1.816330000
N	2.682544000	-1.431260000	-1.891971000
N	2.495424000	2.373771000	-0.188510000
C	0.996576000	-2.399638000	2.097130000
H	0.260127000	-1.643085000	1.804892000
H	0.498895000	-3.081078000	2.793293000
H	1.262215000	-2.980141000	1.206645000
C	1.903800000	-0.754294000	4.481724000
H	1.215831000	0.059782000	4.235178000
H	2.735415000	-0.320572000	5.047292000
H	1.375211000	-1.445038000	5.150919000
C	3.519186000	-3.137721000	3.608904000
H	4.372212000	-2.809948000	4.213133000
H	3.899291000	-3.775117000	2.804749000
H	2.884717000	-3.757404000	4.255150000
C	5.333544000	0.435353000	3.813784000
H	5.239305000	-0.351665000	4.571191000
H	4.630292000	1.231896000	4.076656000
H	6.348164000	0.844188000	3.895515000
C	6.240165000	-1.680699000	1.849331000
H	6.200301000	-2.083908000	0.832521000
H	6.062363000	-2.509189000	2.541206000
H	7.263194000	-1.321042000	2.019711000
C	5.537158000	1.041964000	0.806042000
H	4.866184000	1.903214000	0.800180000
H	5.551796000	0.621303000	-0.206196000
H	6.554226000	1.396312000	1.011643000
C	3.828376000	-3.547944000	-0.128605000
H	2.912747000	-4.132897000	0.017367000
H	3.889535000	-2.805086000	0.670140000
H	4.677668000	-4.233599000	-0.023462000
C	3.501066000	-4.143968000	-3.039580000
H	3.499287000	-3.840820000	-4.090898000
H	2.542388000	-4.629978000	-2.826766000
H	4.283742000	-4.904262000	-2.921352000
C	5.570260000	-2.075162000	-2.174973000

H	5.846173000	-1.282934000	-1.469987000
H	5.626658000	-1.645199000	-3.181575000
H	6.331111000	-2.861887000	-2.102937000
C	3.043598000	-0.928191000	-4.868308000
H	3.575343000	-1.858109000	-5.092895000
H	3.794658000	-0.150941000	-4.691694000
H	2.480932000	-0.647053000	-5.767517000
C	1.001189000	0.581979000	-3.252826000
H	1.709329000	1.407164000	-3.147270000
H	0.294617000	0.623612000	-2.417223000
H	0.427348000	0.769027000	-4.167160000
C	0.546816000	-2.356437000	-3.807412000
H	-0.121113000	-2.488061000	-2.949533000
H	0.969201000	-3.335712000	-4.052701000
H	-0.056389000	-2.030184000	-4.662937000
C	4.307416000	1.878260000	-2.480418000
H	3.914225000	0.859026000	-2.386606000
H	5.361132000	1.844978000	-2.184023000
H	4.275799000	2.133000000	-3.546115000
C	4.656010000	4.412018000	-0.844797000
H	5.388408000	3.948155000	-0.176246000
H	4.201600000	5.257218000	-0.316105000
H	5.205058000	4.822696000	-1.701838000
C	2.247009000	4.149838000	-2.621064000
H	1.720457000	4.946273000	-2.084346000
H	1.493549000	3.517777000	-3.100547000
H	2.837326000	4.622428000	-3.416321000
C	1.068530000	4.867959000	0.776527000
H	0.337909000	4.744877000	-0.026933000
H	1.788100000	5.630086000	0.456100000
H	0.544174000	5.265366000	1.654660000
C	0.756707000	2.138436000	2.172286000
H	1.286843000	1.244997000	2.524734000
H	-0.119710000	1.811512000	1.606131000
H	0.407273000	2.662342000	3.069251000
C	3.313305000	3.685192000	2.421241000
H	4.064552000	4.350980000	1.985060000
H	3.829688000	2.784996000	2.767574000
H	2.896209000	4.187926000	3.303044000
C	-1.995600000	-4.681930000	-0.822924000
H	-2.826718000	-4.937427000	-1.487778000
H	-2.128933000	-5.242302000	0.107761000
H	-1.077344000	-5.046458000	-1.295920000
C	-1.901496000	-3.181772000	-0.582967000
H	-1.781059000	-2.642085000	-1.527773000
H	-1.010218000	-2.935744000	0.003664000
C	-3.523175000	-3.378069000	1.797680000
H	-4.324801000	-2.917219000	2.384749000
H	-3.901555000	-4.357805000	1.482288000
C	-2.261180000	-3.548069000	2.630129000
H	-2.481238000	-4.054808000	3.575939000
H	-1.795785000	-2.588860000	2.861764000
H	-1.517685000	-4.147979000	2.098988000
C	-4.794857000	-2.950449000	-0.668970000
H	-4.773737000	-4.036771000	-0.805973000
H	-5.669146000	-2.713598000	-0.049916000
C	-4.839660000	-2.188908000	-1.979975000
H	-5.787617000	-2.321432000	-2.511838000
H	-4.041835000	-2.539722000	-2.641451000
C	-3.962783000	0.197685000	-3.260569000
H	-4.706926000	-0.055872000	-4.023722000
H	-3.899698000	1.289842000	-3.224067000
C	-2.602013000	-0.390821000	-3.621769000
H	-2.676342000	-1.453097000	-3.871922000
H	-1.900416000	-0.299491000	-2.787650000
H	-2.172513000	0.116203000	-4.491465000
C	-6.304685000	0.178109000	-1.500339000
H	-6.772368000	-0.539165000	-0.813767000
H	-6.320211000	1.136730000	-0.978868000
C	-7.097753000	0.270461000	-2.798438000

H	-8.150859000	0.493874000	-2.592993000
H	-7.069239000	-0.664697000	-3.367698000
H	-6.715992000	1.064658000	-3.447234000
C	-1.644081000	2.908029000	-0.779583000
H	-1.629474000	3.940212000	-0.415412000
H	-0.754623000	2.418075000	-0.375044000
C	-1.604968000	2.868190000	-2.300654000
H	-2.457243000	3.389300000	-2.748295000
H	-1.609585000	1.840776000	-2.665391000
H	-0.693341000	3.346340000	-2.668660000
C	-4.510633000	3.040343000	-0.813519000
H	-4.660676000	2.665101000	-1.829958000
H	-5.406038000	2.754535000	-0.252097000
C	-4.374734000	4.559178000	-0.829236000
H	-5.269789000	5.013357000	-1.268495000
H	-3.516713000	4.890740000	-1.420163000
H	-4.263355000	4.971449000	0.177829000
C	-3.157559000	2.755933000	1.670025000
H	-2.190557000	2.517603000	2.121851000
H	-3.268813000	3.845073000	1.681423000
C	-4.289752000	2.044806000	2.399970000
H	-5.260340000	2.405127000	2.038329000
H	-4.258145000	2.224130000	3.479429000
C	-5.925441000	-0.332815000	2.295057000
H	-6.497227000	0.160291000	1.500207000
H	-5.952696000	-1.403326000	2.057610000
C	-6.558566000	-0.070305000	3.656912000
H	-7.615473000	-0.360204000	3.656629000
H	-6.510748000	0.989548000	3.927757000
H	-6.065217000	-0.639256000	4.450345000
C	-3.355935000	-0.368870000	3.585832000
H	-3.886531000	0.058829000	4.443889000
H	-3.488282000	-1.451999000	3.643794000
C	-1.875951000	-0.010929000	3.641372000
H	-1.730344000	1.065583000	3.769641000
H	-1.369246000	-0.298202000	2.715842000
H	-1.377965000	-0.510744000	4.478315000

1-Sm, S=5/2, small core+solv+disp

Sm	2.166689000	0.016221000	-0.028398000
Fe	-3.137832000	-0.221755000	-0.050152000
P	-3.076365000	1.894663000	0.656133000
P	-4.022788000	-0.682772000	1.900783000
P	-3.209587000	-2.297122000	-0.843331000
P	-4.736961000	0.188209000	-1.521075000
Si	1.641164000	2.430894000	-2.496845000
Si	3.548214000	3.277135000	-0.350673000
Si	2.251583000	0.819474000	3.290067000
Si	4.467523000	-1.054023000	2.530972000
Si	3.973747000	-1.504116000	-2.673853000
Si	2.458475000	-3.278550000	-0.747311000
N	-0.257245000	-0.298953000	-0.036096000
N	-1.413517000	-0.250273000	-0.096751000
N	2.466245000	2.102277000	-1.015748000
N	3.075989000	-0.117220000	2.102072000
N	2.954441000	-1.720163000	-1.292578000
C	2.735593000	2.463858000	-4.041895000
H	3.481075000	3.263458000	-4.010996000
H	2.112012000	2.629392000	-4.930029000
H	3.268634000	1.520126000	-4.186600000
C	0.720309000	4.086819000	-2.491843000
H	0.114671000	4.210473000	-1.589934000
H	0.052479000	4.162826000	-3.359338000
H	1.414170000	4.933511000	-2.540749000
C	0.361094000	1.079490000	-2.823097000
H	0.779100000	0.070951000	-2.722427000
H	-0.012798000	1.165601000	-3.850165000
H	-0.493879000	1.143987000	-2.146788000
C	2.665538000	4.523952000	0.768494000
H	1.949823000	5.116751000	0.187305000

H	3.379835000	5.223546000	1.220343000
H	2.116442000	4.042657000	1.583277000
C	4.467267000	4.311705000	-1.644607000
H	5.065639000	3.676457000	-2.306620000
H	5.156889000	4.998407000	-1.137248000
H	3.802973000	4.919762000	-2.267497000
C	4.910210000	2.428247000	0.642506000
H	4.512081000	1.703598000	1.358699000
H	5.508017000	3.162677000	1.195540000
H	5.587428000	1.896803000	-0.035718000
C	3.217352000	2.282572000	4.001863000
H	3.640156000	2.911098000	3.212286000
H	4.039730000	1.967114000	4.649982000
H	2.548197000	2.911470000	4.602973000
C	1.645947000	-0.210580000	4.755555000
H	1.112471000	-1.102546000	4.411808000
H	0.968958000	0.363760000	5.400236000
H	2.483196000	-0.545094000	5.378826000
C	0.730302000	1.591617000	2.469522000
H	0.996603000	2.164345000	1.570650000
H	0.254023000	2.299401000	3.156472000
H	-0.012650000	0.840547000	2.187929000
C	5.571453000	-0.220088000	3.824398000
H	5.883512000	0.778936000	3.501402000
H	6.479462000	-0.818737000	3.971312000
H	5.084264000	-0.125597000	4.801025000
C	5.567773000	-1.315765000	1.021171000
H	4.994714000	-1.662523000	0.156684000
H	6.353928000	-2.051516000	1.228868000
H	6.060279000	-0.375769000	0.749298000
C	4.016805000	-2.740140000	3.264548000
H	3.431286000	-2.618717000	4.183076000
H	4.920547000	-3.306557000	3.522104000
H	3.427117000	-3.354462000	2.579040000
C	4.984976000	0.077391000	-2.469983000
H	4.365682000	0.928204000	-2.168582000
H	5.493961000	0.346595000	-3.403120000
H	5.755747000	-0.071074000	-1.705657000
C	5.232507000	-2.895348000	-2.918258000
H	5.861103000	-3.026563000	-2.031032000
H	5.893585000	-2.650594000	-3.759313000
H	4.760125000	-3.857248000	-3.145427000
C	2.974075000	-1.415873000	-4.277743000
H	2.442014000	-2.360079000	-4.444347000
H	3.620871000	-1.243376000	-5.146934000
H	2.225939000	-0.618045000	-4.256791000
C	3.850247000	-4.409252000	-0.142774000
H	4.517115000	-3.907685000	0.563313000
H	4.462413000	-4.778890000	-0.970855000
H	3.425884000	-5.284365000	0.365648000
C	1.546855000	-4.264168000	-2.081859000
H	0.774716000	-3.663972000	-2.571072000
H	1.071170000	-5.162159000	-1.668150000
H	2.244578000	-4.596712000	-2.859517000
C	1.282862000	-3.063979000	0.726737000
H	1.698703000	-2.427359000	1.518039000
H	1.090345000	-4.041279000	1.183502000
H	0.317994000	-2.641742000	0.438058000
C	-1.589048000	2.870381000	0.178527000
H	-0.733392000	2.203660000	0.294353000
H	-1.687415000	3.040884000	-0.898832000
C	-1.337469000	4.183431000	0.908737000
H	-0.365421000	4.593625000	0.619055000
H	-1.317344000	4.048436000	1.993843000
H	-2.095100000	4.935834000	0.671987000
C	-4.449832000	3.108670000	0.304946000
H	-4.135084000	3.657272000	-0.589882000
H	-5.333118000	2.536200000	0.017057000
C	-4.836211000	4.088820000	1.408830000
H	-5.605919000	4.776373000	1.040936000

H	-5.251552000	3.573690000	2.280042000
H	-3.992571000	4.692593000	1.750103000
C	-3.050111000	1.833239000	2.503835000
H	-2.064612000	1.452483000	2.783777000
H	-3.165912000	2.819673000	2.960820000
C	-4.147041000	0.866218000	2.934971000
H	-5.134983000	1.305350000	2.747493000
H	-4.088132000	0.636942000	4.004154000
C	-5.734469000	-1.382212000	2.095801000
H	-6.398626000	-0.628239000	1.661929000
H	-5.797282000	-2.251074000	1.429782000
C	-6.204601000	-1.749294000	3.499483000
H	-6.112354000	-0.905715000	4.191426000
H	-5.636869000	-2.586191000	3.915542000
H	-7.259418000	-2.045942000	3.485699000
C	-3.070396000	-1.779779000	3.060448000
H	-3.540828000	-1.730897000	4.048900000
H	-3.193356000	-2.807716000	2.708465000
C	-1.591351000	-1.430690000	3.166270000
H	-1.115699000	-1.378664000	2.184283000
H	-1.060142000	-2.180972000	3.760955000
H	-1.442213000	-0.465005000	3.657398000
C	-1.775836000	-2.777381000	-1.905993000
H	-0.893700000	-2.718466000	-1.263300000
H	-1.888874000	-3.829284000	-2.194706000
C	-1.575277000	-1.889943000	-3.126374000
H	-0.601778000	-2.080581000	-3.588583000
H	-2.340733000	-2.073005000	-3.886756000
H	-1.610041000	-0.835004000	-2.845927000
C	-3.373153000	-3.838063000	0.177642000
H	-4.152841000	-3.653779000	0.924009000
H	-3.757171000	-4.624529000	-0.484563000
C	-2.080907000	-4.302772000	0.839118000
H	-2.276572000	-5.121534000	1.539682000
H	-1.365947000	-4.668570000	0.097948000
H	-1.590506000	-3.499400000	1.392335000
C	-4.671870000	-2.524627000	-1.960760000
H	-4.511141000	-3.374962000	-2.633471000
H	-5.521329000	-2.776882000	-1.313559000
C	-4.940489000	-1.228492000	-2.705934000
H	-5.935221000	-1.215303000	-3.164819000
H	-4.208754000	-1.083473000	-3.505014000
C	-4.637034000	1.588941000	-2.739712000
H	-5.371211000	1.406052000	-3.532750000
H	-4.945303000	2.508438000	-2.235341000
C	-3.241507000	1.748333000	-3.324554000
H	-2.500601000	1.878356000	-2.532967000
H	-3.190342000	2.613697000	-3.994110000
H	-2.941069000	0.866463000	-3.898025000
C	-6.498120000	0.325576000	-0.918087000
H	-6.750147000	-0.692477000	-0.597873000
H	-6.488436000	0.925957000	-0.002630000
C	-7.549338000	0.854673000	-1.886310000
H	-7.375482000	1.905005000	-2.137903000
H	-8.549858000	0.784351000	-1.444860000
H	-7.567404000	0.287756000	-2.822893000

1-Dy, S=5/2, small core+solv+disp

Dy	-2.164320000	0.007886000	-0.000649000
Fe	3.075920000	-0.018687000	-0.073475000
P	3.045913000	-2.150740000	0.524680000
P	4.592642000	0.151973000	1.517608000
P	3.987706000	-0.305723000	-2.030862000
P	3.091813000	2.138829000	-0.574308000
Si	-4.327482000	-0.959136000	-2.620363000
Si	-2.295530000	-2.931974000	-1.668005000
Si	-3.667017000	-1.957243000	2.495794000
Si	-1.703933000	0.171267000	3.325935000
Si	-2.246779000	2.855824000	-1.781661000
Si	-3.950389000	2.961170000	0.691345000

N	0.211567000	-0.005969000	-0.155899000
N	1.368381000	0.008340000	-0.064777000
N	-3.031011000	-1.373022000	-1.543753000
N	-2.578179000	-0.669942000	2.091194000
N	-2.861671000	2.102018000	-0.351696000
C	-3.690544000	-0.313009000	-4.280410000
H	-3.003773000	0.529673000	-4.166218000
H	-4.517674000	0.015695000	-4.921743000
H	-3.152694000	-1.103516000	-4.815998000
C	-5.454889000	0.326967000	-1.829448000
H	-6.008882000	-0.124905000	-0.999280000
H	-6.188928000	0.705620000	-2.550645000
H	-4.885837000	1.173547000	-1.438435000
C	-5.459499000	-2.417180000	-3.041599000
H	-4.951825000	-3.209262000	-3.602015000
H	-6.289209000	-2.059860000	-3.664822000
H	-5.893427000	-2.861988000	-2.139726000
C	-1.667134000	-3.305870000	-3.411962000
H	-2.489022000	-3.488178000	-4.113115000
H	-1.027008000	-4.197091000	-3.419069000
H	-1.081237000	-2.465112000	-3.797153000
C	-0.788319000	-2.980632000	-0.528277000
H	0.042615000	-2.407672000	-0.944902000
H	-0.453725000	-4.014285000	-0.389696000
H	-1.002574000	-2.583367000	0.471161000
C	-3.383627000	-4.389669000	-1.150025000
H	-3.825257000	-4.240254000	-0.160799000
H	-2.778390000	-5.304246000	-1.107972000
H	-4.201094000	-4.570900000	-1.853335000
C	-2.786040000	-3.628698000	2.594014000
H	-2.318825000	-3.913946000	1.647716000
H	-3.485229000	-4.427060000	2.872015000
H	-1.997493000	-3.600106000	3.354860000
C	-4.527187000	-1.735435000	4.166822000
H	-3.833363000	-1.759355000	5.013890000
H	-5.246451000	-2.551404000	4.312552000
H	-5.084785000	-0.793983000	4.209731000
C	-5.057388000	-2.058140000	1.224678000
H	-5.728637000	-1.199599000	1.337538000
H	-5.655643000	-2.966279000	1.365896000
H	-4.673457000	-2.056207000	0.200644000
C	-0.644669000	-0.982143000	4.385650000
H	-1.258689000	-1.646949000	5.003573000
H	0.004108000	-0.410896000	5.061224000
H	-0.006964000	-1.607062000	3.754066000
C	-0.521721000	1.415868000	2.535375000
H	0.359406000	0.930776000	2.112142000
H	-0.182325000	2.113159000	3.309361000
H	-0.983470000	2.020861000	1.745994000
C	-2.774589000	1.170512000	4.524996000
H	-3.482425000	1.819450000	4.001576000
H	-2.132448000	1.811655000	5.142289000
H	-3.348358000	0.533880000	5.204086000
C	-0.950196000	1.719696000	-2.570165000
H	-0.023514000	1.694475000	-1.993290000
H	-0.709640000	2.089967000	-3.573891000
H	-1.291336000	0.684060000	-2.690352000
C	-1.404027000	4.511357000	-1.419797000
H	-2.143007000	5.288921000	-1.195425000
H	-0.819500000	4.856717000	-2.281825000
H	-0.731118000	4.438249000	-0.560971000
C	-3.529054000	3.214073000	-3.125721000
H	-3.020952000	3.463043000	-4.065966000
H	-4.165216000	4.064668000	-2.864359000
H	-4.182437000	2.359985000	-3.319517000
C	-3.045046000	4.055046000	1.941337000
H	-2.418036000	3.479234000	2.626996000
H	-3.761985000	4.623178000	2.547137000
H	-2.399232000	4.778021000	1.430187000
C	-5.128732000	4.114838000	-0.238412000

H	-4.614261000	4.947943000	-0.729024000
H	-5.843743000	4.548545000	0.472206000
H	-5.705174000	3.575943000	-0.997681000
C	-5.059417000	1.756823000	1.627104000
H	-5.791221000	1.310449000	0.945596000
H	-5.616655000	2.276121000	2.416081000
H	-4.486488000	0.947888000	2.088652000
C	3.982774000	-4.222076000	-1.209586000
H	3.705670000	-4.977556000	-1.952474000
H	4.591231000	-4.717823000	-0.447843000
H	4.617753000	-3.487387000	-1.711752000
C	2.732866000	-3.592151000	-0.603447000
H	2.180356000	-4.351575000	-0.041973000
H	2.057349000	-3.240474000	-1.386953000
C	1.809136000	-3.733110000	2.617181000
H	1.024706000	-3.738656000	3.381243000
H	2.760922000	-3.915193000	3.126721000
H	1.616267000	-4.582996000	1.955407000
C	1.804135000	-2.406208000	1.871959000
H	1.963766000	-1.571281000	2.560430000
H	0.828185000	-2.221510000	1.416753000
C	4.630045000	-2.593520000	1.365689000
H	4.593919000	-3.584273000	1.830442000
H	5.411116000	-2.612223000	0.598010000
C	4.895938000	-1.483329000	2.370736000
H	5.910913000	-1.527796000	2.778845000
H	4.204738000	-1.566933000	3.214460000
C	6.359323000	0.547389000	1.060642000
H	6.705749000	-0.361249000	0.553412000
H	6.337363000	1.323803000	0.293744000
C	7.324833000	0.936007000	2.173913000
H	7.337953000	0.201410000	2.986163000
H	7.069717000	1.907043000	2.608868000
H	8.347569000	1.009943000	1.787076000
C	4.294150000	1.240858000	2.994405000
H	5.146496000	1.181762000	3.679714000
H	4.246026000	2.272476000	2.629905000
C	3.000837000	0.884977000	3.718718000
H	2.178744000	0.762672000	3.008889000
H	2.721315000	1.664913000	4.434493000
H	3.095767000	-0.050327000	4.279059000
C	1.615511000	-1.029165000	-3.367871000
H	1.128624000	-1.064478000	-2.390958000
H	1.101950000	-1.728494000	-4.035095000
H	1.460765000	-0.022840000	-3.768486000
C	3.096904000	-1.371105000	-3.266687000
H	3.581637000	-1.260453000	-4.243389000
H	3.225642000	-2.414761000	-2.970297000
C	6.257421000	-1.133601000	-3.651099000
H	5.755085000	-1.989512000	-4.111111000
H	7.331170000	-1.352791000	-3.640701000
H	6.106973000	-0.268284000	-4.305099000
C	5.747741000	-0.869088000	-2.238921000
H	6.343796000	-0.085509000	-1.757607000
H	5.870540000	-1.758375000	-1.610774000
C	4.028596000	1.305510000	-2.987386000
H	5.026741000	1.743362000	-2.867123000
H	3.880796000	1.127448000	-4.057427000
C	2.970711000	2.241963000	-2.417426000
H	1.966432000	1.898227000	-2.680528000
H	3.075299000	3.267937000	-2.785327000
C	1.698849000	3.202442000	-0.006740000
H	1.615221000	4.061911000	-0.680478000
H	0.788525000	2.609919000	-0.130959000
C	1.827772000	3.651071000	1.443188000
H	0.934410000	4.199800000	1.756459000
H	2.690190000	4.308180000	1.593879000
H	1.934995000	2.791365000	2.106301000
C	4.568926000	3.218699000	-0.267297000
H	4.794618000	3.179159000	0.802651000

H	5.405585000	2.723599000	-0.770766000
C	4.469608000	4.666605000	-0.735500000
H	3.653779000	5.204136000	-0.244548000
H	4.310228000	4.735571000	-1.815450000
H	5.398218000	5.202025000	-0.509243000

1*-Yb, S=0, small core+solv+disp

Yb	2.256320000	-0.026296000	0.266640000
Fe	-2.946272000	-0.054738000	-0.041188000
P	-2.820595000	1.959862000	0.833196000
P	-4.003001000	0.952724000	-1.666499000
P	-4.312776000	-0.829934000	1.459274000
P	-3.132136000	-2.047282000	-0.960076000
Si	5.135248000	-1.565474000	-0.963167000
Si	2.667414000	-3.303257000	-0.505210000
Si	2.529228000	2.158323000	-2.169136000
Si	3.186206000	3.433781000	0.523471000
O	2.535164000	-0.500403000	2.604778000
N	-0.114946000	-0.332841000	0.345678000
N	-1.250358000	-0.236838000	0.153623000
N	3.564331000	-1.861506000	-0.369343000
N	2.694444000	2.144937000	-0.472679000
C	5.704733000	0.132625000	-0.347954000
H	4.957782000	0.914859000	-0.527081000
H	6.634670000	0.450988000	-0.834453000
H	5.903520000	0.100564000	0.730711000
C	5.254438000	-1.568677000	-2.856538000
H	4.985289000	-2.551547000	-3.262712000
H	6.270715000	-1.338744000	-3.201001000
H	4.578462000	-0.833719000	-3.306916000
C	6.457174000	-2.785873000	-0.355660000
H	7.461549000	-2.422867000	-0.609301000
H	6.345908000	-3.780398000	-0.799797000
H	6.415587000	-2.904716000	0.733928000
C	1.527639000	-3.489659000	1.003638000
H	2.122532000	-3.582023000	1.919447000
H	0.897560000	-4.383975000	0.924990000
H	0.860455000	-2.628968000	1.127533000
C	3.676170000	-4.904939000	-0.597250000
H	4.293079000	-4.945643000	-1.502109000
H	3.011990000	-5.778511000	-0.615885000
H	4.342787000	-5.012892000	0.265534000
C	1.548891000	-3.294108000	-2.037448000
H	0.895753000	-2.414673000	-2.035073000
H	0.918160000	-4.189330000	-2.102322000
H	2.149120000	-3.239357000	-2.953461000
C	4.152507000	2.413007000	-3.111686000
H	4.575916000	3.402370000	-2.899304000
H	4.009158000	2.343981000	-4.197519000
H	4.902732000	1.668553000	-2.825389000
C	1.320003000	3.458604000	-2.833272000
H	0.401712000	3.471240000	-2.237611000
H	1.046460000	3.256898000	-3.876049000
H	1.751964000	4.464310000	-2.795272000
C	1.842281000	0.465469000	-2.705498000
H	2.510177000	-0.369785000	-2.455048000
H	1.699989000	0.428787000	-3.791567000
H	0.861114000	0.265423000	-2.254618000
C	2.377945000	3.234164000	2.230119000
H	1.285317000	3.214974000	2.145366000
H	2.646023000	4.045360000	2.917792000
H	2.695047000	2.294758000	2.697956000
C	2.747222000	5.164200000	-0.118884000
H	3.333716000	5.411175000	-1.011933000
H	2.969184000	5.929192000	0.635885000
H	1.690256000	5.256522000	-0.386915000
C	5.056594000	3.498356000	0.833861000
H	5.421992000	2.600689000	1.344151000
H	5.330576000	4.364874000	1.449413000
H	5.606089000	3.574087000	-0.111758000

C	-4.196307000	3.014383000	0.191060000
H	-5.110089000	2.680994000	0.693935000
H	-4.051351000	4.071688000	0.437082000
C	-4.289233000	2.770902000	-1.307415000
H	-3.505325000	3.333128000	-1.825254000
H	-5.246859000	3.104058000	-1.719760000
C	-2.880967000	2.314404000	2.646269000
H	-2.991512000	3.394734000	2.795648000
H	-3.793883000	1.851166000	3.034874000
C	-1.650403000	1.789386000	3.377253000
H	-0.754815000	2.353525000	3.100007000
H	-1.469226000	0.739525000	3.133216000
H	-1.766731000	1.870261000	4.463154000
C	-1.302702000	2.882364000	0.318830000
H	-0.442975000	2.289542000	0.647132000
H	-1.279556000	2.817287000	-0.772732000
C	-1.170862000	4.326145000	0.781560000
H	-0.239041000	4.758355000	0.406411000
H	-1.149454000	4.410197000	1.872475000
H	-1.992594000	4.950444000	0.415777000
C	-5.726076000	0.412803000	-2.131528000
H	-5.701713000	-0.678547000	-2.215332000
H	-6.331406000	0.629217000	-1.243097000
C	-6.366109000	1.014241000	-3.377574000
H	-5.850970000	0.696672000	-4.288820000
H	-6.359169000	2.109188000	-3.357923000
H	-7.411651000	0.697455000	-3.465854000
C	-3.214048000	1.111555000	-3.345453000
H	-3.264104000	0.132450000	-3.829210000
H	-3.805985000	1.798897000	-3.960660000
C	-1.768668000	1.580929000	-3.254084000
H	-1.231178000	1.029303000	-2.476827000
H	-1.708036000	2.643598000	-3.001564000
H	-1.242092000	1.444762000	-4.203737000
C	-4.898015000	-2.566601000	1.059685000
H	-4.276781000	-3.258226000	1.638507000
H	-5.932055000	-2.718055000	1.385661000
C	-4.721648000	-2.834533000	-0.426878000
H	-5.517941000	-2.349920000	-1.001648000
H	-4.749308000	-3.902946000	-0.666827000
C	-5.926379000	0.031430000	1.819110000
H	-5.685618000	1.092515000	1.945909000
H	-6.494499000	-0.035399000	0.883585000
C	-6.768907000	-0.453969000	2.993316000
H	-6.272599000	-0.269259000	3.950540000
H	-6.981956000	-1.526323000	2.932180000
H	-7.732532000	0.067557000	3.019335000
C	-3.694018000	-1.131691000	3.190122000
H	-3.599700000	-0.159958000	3.684463000
H	-4.446306000	-1.699161000	3.750544000
C	-2.356890000	-1.859532000	3.211218000
H	-1.624038000	-1.357959000	2.572937000
H	-2.453147000	-2.887757000	2.847222000
H	-1.958201000	-1.915310000	4.228957000
C	-3.155529000	-2.380536000	-2.778385000
H	-3.472141000	-3.417445000	-2.942559000
H	-3.939485000	-1.749265000	-3.208952000
C	-1.809320000	-2.115252000	-3.443571000
H	-1.074187000	-2.876323000	-3.170467000
H	-1.398409000	-1.146918000	-3.144813000
H	-1.899138000	-2.121351000	-4.534930000
C	-1.838779000	-3.232712000	-0.379405000
H	-0.882134000	-2.807526000	-0.694568000
H	-1.838166000	-3.161299000	0.712073000
C	-1.952953000	-4.681796000	-0.830532000
H	-1.130153000	-5.274163000	-0.416394000
H	-1.905675000	-4.778218000	-1.919341000
H	-2.887178000	-5.144156000	-0.495248000
C	1.628237000	-0.147302000	3.652479000
H	0.816280000	0.380348000	3.146502000

H	2.106992000	0.563797000	4.336851000
C	1.087900000	-1.350119000	4.398014000
H	1.857987000	-1.854776000	4.988399000
H	0.653961000	-2.071032000	3.700742000
H	0.307739000	-1.021312000	5.091723000
C	3.797922000	-1.060531000	3.000337000
H	3.628700000	-1.870812000	3.717862000
H	4.185425000	-1.508793000	2.079944000
C	4.744141000	-0.016264000	3.554818000
H	5.718444000	-0.474829000	3.753204000
H	4.384541000	0.416947000	4.493364000
H	4.892089000	0.794825000	2.835721000

1*-Sm, S=3, small core+solv+disp

Sm	2.299938000	-0.129757000	-0.663811000
Fe	-2.832413000	-0.072779000	-0.001135000
P	-3.762480000	-1.077538000	-1.721789000
P	-4.198729000	-1.298151000	1.176957000
P	-3.622156000	1.888643000	-0.492655000
Si	5.184057000	-1.931008000	-1.040098000
P	-1.992521000	0.922391000	1.779245000
Si	3.027641000	-3.345925000	0.630386000
Si	2.909122000	3.273660000	-0.837403000
Si	4.326082000	1.916093000	1.486035000
N	-1.262505000	-0.481912000	-0.568519000
N	3.653271000	-2.079126000	-0.316216000
N	3.220212000	1.951250000	0.185189000
N	-0.200909000	-0.689955000	-0.974286000
C	-5.457599000	2.145752000	-0.686453000
H	-5.815322000	1.335910000	-1.331739000
H	-5.872803000	1.938907000	0.307406000
C	-3.534926000	-2.654045000	2.268433000
H	-3.121051000	-2.189420000	3.167630000
H	-4.363533000	-3.292502000	2.596223000
C	-5.342283000	-2.322650000	0.102808000
H	-4.896393000	-3.318530000	0.007295000
H	-6.318743000	-2.453127000	0.580312000
C	-2.974629000	2.787893000	-1.988586000
H	-3.417659000	2.328627000	-2.876932000
H	-3.320039000	3.828122000	-1.965813000
C	-4.067941000	-0.299807000	-3.371462000
H	-4.738207000	-0.947389000	-3.949070000
H	-4.617182000	0.630213000	-3.191230000
C	5.146545000	-0.347583000	-2.100725000
H	4.465683000	-0.460225000	-2.957337000
H	6.134248000	-0.126109000	-2.521270000
H	4.853099000	0.546175000	-1.531164000
C	-5.453147000	-1.671656000	-1.266872000
H	-6.096501000	-0.786177000	-1.223922000
H	-5.870091000	-2.345571000	-2.022828000
C	-3.202903000	3.172770000	0.805159000
H	-2.301982000	3.694443000	0.464774000
H	-3.994841000	3.924155000	0.887037000
C	5.633290000	-3.346265000	-2.217174000
H	5.756025000	-4.288793000	-1.670173000
H	6.573297000	-3.152097000	-2.749097000
H	4.848826000	-3.501813000	-2.967167000
C	-2.880554000	-2.632220000	-2.202700000
H	-1.871916000	-2.324418000	-2.498547000
H	-2.745207000	-3.190013000	-1.271278000
C	-5.433328000	-0.478268000	2.308698000
H	-4.891283000	0.296043000	2.861693000
H	-6.107760000	0.060442000	1.631495000
C	-2.463420000	-3.479539000	1.568025000
H	-1.713754000	-2.829844000	1.105449000
H	-2.891466000	-4.101336000	0.774385000
H	-1.959340000	-4.151859000	2.270081000
C	2.437545000	-4.826419000	-0.394129000
H	1.676232000	-4.512862000	-1.119110000
H	1.999389000	-5.614919000	0.230644000

H	3.263947000	-5.270428000	-0.961344000
C	-1.875666000	0.219890000	3.488842000
H	-1.679867000	1.044713000	4.184288000
H	-2.866077000	-0.168682000	3.746000000
C	-2.780215000	-0.032381000	-4.144277000
H	-2.317859000	-0.966100000	-4.478296000
H	-2.045754000	0.492831000	-3.528315000
H	-2.973053000	0.576420000	-5.033739000
C	2.150655000	2.630780000	-2.465730000
H	2.866085000	2.013416000	-3.025972000
H	1.888747000	3.470363000	-3.120396000
H	1.224194000	2.056225000	-2.333697000
C	6.615027000	-1.781886000	0.189527000
H	6.392948000	-1.056242000	0.978045000
H	7.547470000	-1.473337000	-0.299256000
H	6.803564000	-2.745098000	0.677646000
C	-1.454961000	2.731307000	-2.071342000
H	-1.093908000	3.135101000	-3.022705000
H	-1.099495000	1.701986000	-1.970823000
H	-0.984527000	3.317316000	-1.275778000
C	-3.518535000	-3.506670000	-3.274198000
H	-2.897628000	-4.388854000	-3.465190000
H	-3.632334000	-2.977416000	-4.225363000
H	-4.507990000	-3.865831000	-2.973411000
C	-0.254023000	1.492374000	1.512303000
H	0.332380000	0.568149000	1.441574000
H	-0.231104000	1.937893000	0.512414000
C	1.671691000	4.526886000	-0.134866000
H	0.721226000	4.048389000	0.123992000
H	1.457313000	5.331398000	-0.849456000
H	2.060184000	4.989425000	0.779728000
C	-0.812027000	-0.861833000	3.629385000
H	0.193503000	-0.434371000	3.588403000
H	-0.882758000	-1.604997000	2.831942000
H	-0.903916000	-1.384344000	4.587238000
C	-5.955335000	3.488334000	-1.209534000
H	-5.653884000	3.653933000	-2.248021000
H	-5.575456000	4.327080000	-0.616879000
H	-7.049797000	3.535675000	-1.175218000
C	1.504744000	-2.695350000	1.558935000
H	1.781030000	-1.884376000	2.243350000
H	1.039679000	-3.481175000	2.165324000
H	0.732895000	-2.327872000	0.870563000
C	4.227272000	-4.004805000	1.938568000
H	5.090641000	-4.498885000	1.477752000
H	3.744811000	-4.736997000	2.598263000
H	4.608331000	-3.189533000	2.564099000
C	-2.930245000	2.479861000	2.128979000
H	-3.868396000	2.169223000	2.602593000
H	-2.400275000	3.121295000	2.841047000
C	-6.225430000	-1.352927000	3.273571000
H	-6.748129000	-2.165363000	2.757894000
H	-6.983308000	-0.761241000	3.799623000
H	-5.579083000	-1.804415000	4.031975000
C	6.141271000	1.923102000	0.939024000
H	6.341529000	1.169342000	0.171653000
H	6.817658000	1.727890000	1.780703000
H	6.415207000	2.897552000	0.518390000
C	4.440046000	4.262099000	-1.352527000
H	4.909884000	4.749346000	-0.490348000
H	4.188656000	5.048198000	-2.075636000
H	5.191835000	3.611376000	-1.813330000
C	0.368033000	2.427068000	2.535639000
H	1.392944000	2.652565000	2.231237000
H	0.398974000	1.982242000	3.535032000
H	-0.173972000	3.374957000	2.606034000
C	4.181358000	3.373756000	2.693959000
H	4.359793000	4.328518000	2.184637000
H	4.928376000	3.289634000	3.493707000
H	3.196872000	3.433141000	3.170295000

C	4.025153000	0.328423000	2.475797000
H	3.035902000	0.346531000	2.951224000
H	4.764233000	0.196787000	3.275064000
H	4.080968000	-0.559590000	1.832608000

3, S=3, small core+solv+disp

Sm	-0.101018000	2.191529000	0.008920000
Fe	4.071971000	-1.239262000	-0.224972000
Fe	-3.964758000	-1.529678000	0.149066000
P	3.847471000	-1.644948000	-2.370675000
P	4.204792000	-3.411610000	-0.038492000
P	6.091700000	-0.469354000	-0.359841000
P	4.177010000	-0.805731000	1.939593000
P	-4.666613000	-0.527919000	-1.682020000
P	-5.892154000	-0.936965000	0.959903000
P	-3.334470000	-2.559690000	1.984952000
P	-3.952058000	-3.503587000	-0.740432000
Si	-0.676131000	3.088674000	-3.315919000
Si	-2.066786000	4.872698000	-1.239873000
Si	2.193256000	4.428004000	1.478946000
Si	-0.061592000	3.459538000	3.295312000
N	2.720179000	-0.169875000	-0.327766000
N	1.852531000	0.582771000	-0.408094000
N	-1.638511000	0.161714000	0.201009000
N	-2.566871000	-0.516639000	0.187424000
N	-1.205059000	3.495407000	-1.753657000
N	0.833069000	3.474771000	1.852504000
C	3.910243000	0.825521000	-3.686533000
H	2.876838000	0.785046000	-4.039873000
H	4.455376000	1.515406000	-4.339068000
H	3.893831000	1.253318000	-2.680388000
C	4.549974000	-0.559591000	-3.688529000
H	5.626768000	-0.491318000	-3.500341000
H	4.433168000	-1.044825000	-4.664965000
C	1.767480000	-2.131909000	-4.326864000
H	0.684318000	-2.169575000	-4.486847000
H	2.176430000	-3.105741000	-4.616599000
H	2.168473000	-1.383673000	-5.017612000
C	2.073875000	-1.787274000	-2.876480000
H	1.641678000	-2.524716000	-2.192497000
H	1.602523000	-0.839817000	-2.602521000
C	4.538610000	-3.306987000	-2.789506000
H	4.252787000	-3.633529000	-3.795277000
H	5.630083000	-3.212651000	-2.769242000
C	4.059236000	-4.259239000	-1.703621000
H	4.605868000	-5.208774000	-1.709062000
H	3.000230000	-4.495033000	-1.856920000
C	5.797388000	-4.191409000	0.556387000
H	6.491659000	-4.058664000	-0.281341000
H	6.185586000	-3.562447000	1.364329000
C	5.772832000	-5.654173000	0.983029000
H	5.374978000	-6.302270000	0.194788000
H	5.160225000	-5.805661000	1.876716000
H	6.784240000	-6.007523000	1.214992000
C	2.939732000	-4.426663000	0.879385000
H	3.029967000	-5.473250000	0.564969000
H	3.171554000	-4.400734000	1.947861000
C	1.534477000	-3.907514000	0.623988000
H	0.787937000	-4.509392000	1.152496000
H	1.291721000	-3.939372000	-0.442001000
H	1.439181000	-2.864074000	0.936270000
C	8.850675000	-0.758403000	-1.258458000
H	9.579814000	-1.453224000	-1.691169000
H	9.209086000	-0.490994000	-0.259171000
H	8.863697000	0.147578000	-1.870835000
C	7.465292000	-1.395359000	-1.211178000
H	7.515137000	-2.362748000	-0.702032000
H	7.113796000	-1.608968000	-2.227251000
C	6.360438000	1.220546000	-1.095328000
H	7.400064000	1.530665000	-0.941320000

H	6.221169000	1.110223000	-2.175975000
C	5.405511000	2.271848000	-0.548576000
H	4.385746000	1.881715000	-0.490999000
H	5.394767000	3.166658000	-1.178865000
H	5.695597000	2.587402000	0.456294000
C	6.820197000	-0.259280000	1.345616000
H	7.220098000	-1.238215000	1.638999000
H	7.653993000	0.451310000	1.346598000
C	5.718367000	0.169859000	2.306663000
H	5.460976000	1.221467000	2.150013000
H	6.022311000	0.069985000	3.353598000
C	4.238010000	-2.152610000	3.218460000
H	3.340062000	-2.754878000	3.054622000
H	5.086934000	-2.795076000	2.962781000
C	4.314299000	-1.730534000	4.681347000
H	4.366563000	-2.612598000	5.329656000
H	3.435900000	-1.153905000	4.984771000
H	5.198571000	-1.121477000	4.891408000
C	2.857406000	0.276556000	2.647582000
H	3.195980000	0.656680000	3.617435000
H	2.755475000	1.146743000	1.994116000
C	1.508123000	-0.420446000	2.772441000
H	1.147726000	-0.756005000	1.796162000
H	0.766694000	0.267745000	3.187283000
H	1.552012000	-1.290667000	3.434891000
C	0.095237000	1.351248000	-3.247937000
H	1.061262000	1.369988000	-2.729493000
H	0.279246000	0.959452000	-4.255203000
H	-0.544886000	0.628041000	-2.729259000
C	0.687847000	4.217916000	-3.997409000
H	0.344078000	5.257142000	-4.065883000
H	1.016266000	3.911013000	-4.998861000
H	1.565398000	4.208550000	-3.340345000
C	-2.011829000	3.036647000	-4.661389000
H	-1.604158000	2.636220000	-5.598467000
H	-2.403111000	4.036907000	-4.876259000
H	-2.860415000	2.409695000	-4.372987000
C	-0.951314000	6.097900000	-0.326161000
H	-0.459933000	5.589774000	0.510766000
H	-1.510384000	6.950367000	0.079672000
H	-0.165762000	6.492269000	-0.981305000
C	-2.941215000	5.850827000	-2.610680000
H	-2.225919000	6.234444000	-3.347754000
H	-3.469500000	6.715441000	-2.188950000
H	-3.678573000	5.245445000	-3.149084000
C	-3.409227000	4.396728000	0.018313000
H	-4.253355000	3.895731000	-0.466024000
H	-3.801374000	5.278409000	0.540067000
H	-3.021912000	3.715595000	0.785277000
C	2.457195000	4.349125000	-0.398220000
H	2.584908000	3.315694000	-0.747191000
H	3.349357000	4.902862000	-0.712062000
H	1.599851000	4.781708000	-0.926529000
C	3.792973000	3.834709000	2.307993000
H	3.682675000	3.812461000	3.398415000
H	4.639994000	4.490033000	2.069600000
H	4.047962000	2.823465000	1.984032000
C	2.065830000	6.259333000	1.956681000
H	1.153823000	6.725716000	1.571767000
H	2.921157000	6.823879000	1.563346000
H	2.070819000	6.381835000	3.046552000
C	-0.946323000	5.089872000	3.685216000
H	-0.239619000	5.903986000	3.878580000
H	-1.594948000	4.996789000	4.565897000
H	-1.573669000	5.395662000	2.839536000
C	-1.463186000	2.186679000	3.133696000
H	-2.215778000	2.518271000	2.408534000
H	-1.988261000	2.070312000	4.088969000
H	-1.128364000	1.190038000	2.826881000
C	0.945529000	3.003044000	4.836844000

H	1.498487000	2.068718000	4.690280000
H	0.309045000	2.883515000	5.722777000
H	1.679942000	3.784836000	5.065192000
C	-2.839263000	-0.858139000	-3.805187000
H	-2.607178000	-1.356940000	-4.752331000
H	-2.161972000	-1.250006000	-3.041950000
H	-2.610212000	0.202351000	-3.930436000
C	-4.298482000	-1.064067000	-3.415022000
H	-4.582921000	-2.119398000	-3.487195000
H	-4.960019000	-0.519836000	-4.099680000
C	-4.220038000	1.264135000	-1.736733000
H	-4.315934000	1.622732000	-0.709139000
H	-3.148044000	1.328122000	-1.951883000
C	-5.012847000	2.143884000	-2.692349000
H	-6.056607000	2.245959000	-2.377775000
H	-5.011819000	1.754027000	-3.715399000
H	-4.579114000	3.147044000	-2.728250000
C	-6.517037000	-0.514125000	-1.705053000
H	-6.912827000	0.133450000	-2.494547000
H	-6.835668000	-1.538506000	-1.926346000
C	-6.977752000	-0.099439000	-0.317555000
H	-8.035758000	-0.323127000	-0.146325000
H	-6.852477000	0.981382000	-0.190395000
C	-5.083892000	1.551159000	1.992470000
H	-5.477600000	2.147376000	1.163435000
H	-4.080643000	1.219754000	1.708476000
H	-4.997666000	2.217104000	2.857233000
C	-5.979460000	0.356998000	2.298380000
H	-7.019774000	0.677342000	2.429444000
H	-5.672899000	-0.112028000	3.237655000
C	-7.065845000	-2.226990000	1.623897000
H	-7.333889000	-2.830762000	0.747954000
H	-6.476102000	-2.887642000	2.268542000
C	-8.317030000	-1.762369000	2.360643000
H	-8.964814000	-2.613375000	2.601161000
H	-8.909733000	-1.061753000	1.763121000
H	-8.068486000	-1.264660000	3.302681000
C	-3.854532000	-2.119327000	3.706699000
H	-4.947581000	-2.059769000	3.710665000
H	-3.588787000	-2.944356000	4.378565000
C	-3.238308000	-0.809512000	4.184951000
H	-3.694270000	-0.474865000	5.122800000
H	-3.366643000	-0.016348000	3.445160000
H	-2.164055000	-0.915068000	4.361679000
C	-1.495567000	-2.627387000	2.176745000
H	-1.109884000	-2.953700000	1.206896000
H	-1.171619000	-1.586533000	2.274113000
C	-0.932209000	-3.475194000	3.308252000
H	-1.201979000	-4.531137000	3.202175000
H	-1.285526000	-3.139934000	4.288244000
H	0.161231000	-3.418977000	3.321222000
C	-3.838283000	-4.339423000	1.903866000
H	-3.360292000	-4.940154000	2.685481000
H	-4.918294000	-4.359457000	2.087464000
C	-3.526828000	-4.841873000	0.503075000
H	-4.048923000	-5.776452000	0.273694000
H	-2.453880000	-5.042559000	0.410170000
C	-5.500766000	-4.206355000	-1.507999000
H	-6.214254000	-4.274538000	-0.677720000
H	-5.892572000	-3.429100000	-2.173226000
C	-5.412886000	-5.532339000	-2.255282000
H	-6.409907000	-5.876731000	-2.553565000
H	-4.966945000	-6.322591000	-1.641953000
H	-4.813203000	-5.442907000	-3.165929000
C	-2.699917000	-3.927699000	-2.054425000
H	-2.711961000	-5.009759000	-2.232143000
H	-3.017336000	-3.451412000	-2.986410000
C	-1.298206000	-3.468726000	-1.679062000
H	-0.922587000	-4.023903000	-0.815190000
H	-1.281080000	-2.408233000	-1.411193000

H	-0.596015000	-3.632760000	-2.503673000

1-Ce, S=1/2, large core

Ce	9.394044000	8.083787000	15.827142000
Fe	8.777550000	6.439030000	10.521308000
P	6.508392000	6.246307000	10.677688000
P	8.658782000	4.314766000	9.747141000
P	11.039930000	6.568325000	10.289457000
P	8.743724000	8.013520000	8.917659000
Si	6.998038000	10.681839000	15.492693000
Si	8.865603000	11.149219000	17.844701000
Si	7.254344000	6.424943000	18.429056000
Si	8.919458000	4.665584000	16.634299000
Si	12.887045000	7.579680000	17.144211000
Si	12.434697000	9.657500000	14.995601000
N	9.030455000	7.296217000	13.273181000
N	8.919938000	6.929771000	12.179580000
N	8.342843000	10.159983000	16.484641000
N	8.395129000	6.276154000	17.089938000
N	11.791272000	8.398954000	16.034472000
C	6.369846000	9.164363000	14.518963000
H	7.082893000	8.815669000	13.762160000
H	5.455191000	9.440751000	13.984232000
H	6.117914000	8.329530000	15.182887000
C	7.454733000	11.998021000	14.193083000
H	8.239909000	11.645907000	13.516786000
H	7.813864000	12.916591000	14.667390000
H	6.578766000	12.257233000	13.586180000
C	5.490728000	11.378828000	16.420678000
H	5.706491000	12.353353000	16.870109000
H	5.145891000	10.707299000	17.211654000
H	4.665825000	11.522209000	15.712450000
C	9.349962000	12.917237000	17.324503000
H	8.471337000	13.488850000	17.005241000
H	10.066352000	12.919550000	16.497245000
H	9.801488000	13.456449000	18.165289000
C	7.574747000	11.342915000	19.232618000
H	7.236665000	10.370346000	19.603232000
H	6.692549000	11.910622000	18.926073000
H	8.035821000	11.875628000	20.073307000
C	10.356377000	10.337462000	18.694802000
H	11.157664000	10.080553000	17.997291000
H	10.053577000	9.421882000	19.215861000
H	10.763195000	11.017090000	19.452320000
C	6.038612000	7.852282000	18.142437000
H	5.327029000	7.598756000	17.348625000
H	6.556538000	8.772523000	17.858756000
H	5.461864000	8.048644000	19.053669000
C	6.133194000	4.905401000	18.709494000
H	6.676951000	3.983709000	18.935575000
H	5.489095000	4.718398000	17.844315000
H	5.480049000	5.117025000	19.564732000
C	8.154352000	6.739662000	20.074850000
H	8.725025000	7.673595000	20.047076000
H	8.856959000	5.931022000	20.300487000
H	7.441641000	6.810506000	20.904672000
C	9.626304000	3.611496000	18.054906000
H	8.896147000	3.444921000	18.852525000
H	10.506551000	4.081880000	18.502736000
H	9.928401000	2.628350000	17.674534000
C	10.314721000	4.823534000	15.346332000
H	11.150080000	5.443258000	15.688655000
H	9.952130000	5.227596000	14.395078000
H	10.725016000	3.828320000	15.141102000
C	7.573617000	3.620276000	15.781362000
H	7.096355000	4.192140000	14.978796000
H	6.792924000	3.300694000	16.476413000
H	8.017258000	2.719743000	15.340235000
C	11.910032000	6.814693000	18.582825000
H	10.972743000	6.345942000	18.266702000

H	11.659849000	7.581598000	19.322622000
H	12.511944000	6.050911000	19.088490000
C	14.184194000	8.717438000	17.954949000
H	13.715913000	9.558185000	18.475867000
H	14.905302000	9.119921000	17.236147000
H	14.750311000	8.140353000	18.695960000
C	13.900745000	6.194487000	16.310662000
H	14.508707000	6.593456000	15.492352000
H	13.271196000	5.397966000	15.903237000
H	14.581594000	5.738368000	17.039223000
C	13.963656000	9.128113000	13.988196000
H	13.794487000	8.191943000	13.449380000
H	14.834262000	8.986534000	14.637212000
H	14.223973000	9.902870000	13.257205000
C	11.078121000	10.167554000	13.751358000
H	10.218755000	10.621526000	14.261398000
H	10.721051000	9.343360000	13.123657000
H	11.479723000	10.936959000	13.081863000
C	12.929872000	11.277515000	15.862294000
H	13.783523000	11.144267000	16.532078000
H	12.104943000	11.690464000	16.449365000
H	13.211361000	12.024617000	15.110223000
C	4.474744000	5.314979000	12.564128000
H	4.285213000	4.377054000	12.032698000
H	3.749438000	6.052392000	12.206031000
H	4.257199000	5.133987000	13.622196000
C	5.915720000	5.788833000	12.397816000
H	6.620892000	5.028314000	12.748658000
H	6.106988000	6.671915000	13.016218000
C	5.195916000	7.483388000	10.132457000
H	5.493660000	7.824955000	9.134975000
H	4.264293000	6.921016000	9.997923000
C	4.961847000	8.664922000	11.067329000
H	4.267778000	9.382928000	10.616822000
H	5.887433000	9.196434000	11.301410000
H	4.525833000	8.340661000	12.015963000
C	5.937886000	4.789890000	9.643450000
H	4.900372000	4.527041000	9.876466000
H	5.964441000	5.125928000	8.599713000
C	6.901732000	3.639867000	9.871635000
H	6.748386000	2.818241000	9.163861000
H	6.772452000	3.232151000	10.879006000
C	9.600333000	2.909425000	10.582301000
H	9.392644000	1.973544000	10.052897000
H	10.666849000	3.121559000	10.451002000
C	9.276385000	2.767187000	12.066342000
H	8.275446000	2.354875000	12.226103000
H	9.319861000	3.733573000	12.577138000
H	9.983722000	2.090181000	12.556948000
C	8.965982000	3.939833000	7.910673000
H	8.128179000	4.436441000	7.405934000
H	9.860213000	4.495823000	7.618879000
C	9.077836000	2.487383000	7.452664000
H	9.116212000	2.431941000	6.358665000
H	8.224739000	1.880914000	7.774867000
H	9.985591000	2.009243000	7.832431000
C	12.177873000	6.428579000	11.766977000
H	13.115576000	6.933755000	11.510370000
H	11.711440000	7.011317000	12.566624000
C	12.443785000	5.003772000	12.242733000
H	12.945245000	4.400812000	11.478271000
H	11.517037000	4.496942000	12.520350000
H	13.087798000	5.014170000	13.127344000
C	11.974614000	5.565445000	8.994240000
H	11.795391000	4.509233000	9.217986000
H	11.462090000	5.764200000	8.047075000
C	13.472528000	5.823196000	8.842236000
H	13.887603000	5.185055000	8.054103000
H	14.024894000	5.605418000	9.760462000
H	13.687138000	6.858848000	8.563015000

C	11.409914000	8.306649000	9.697220000
H	11.179654000	8.968017000	10.538518000
H	12.470606000	8.434342000	9.455631000
C	10.501819000	8.592982000	8.508669000
H	10.840335000	8.039460000	7.624701000
H	10.497507000	9.655083000	8.241811000
C	8.077368000	7.674968000	7.179845000
H	8.625273000	6.784543000	6.850701000
H	7.036770000	7.352835000	7.310981000
C	8.163647000	8.775354000	6.123857000
H	7.830890000	8.400861000	5.149011000
H	9.187871000	9.140958000	5.997645000
H	7.532378000	9.633490000	6.371360000
C	7.950388000	9.692740000	9.252130000
H	8.151112000	10.343931000	8.394070000
H	6.867976000	9.539741000	9.287416000
C	8.438871000	10.345440000	10.540294000
H	9.493287000	10.631561000	10.471619000
H	8.338609000	9.667163000	11.392596000
H	7.871898000	11.256419000	10.758919000

1-Sm, S=5/2, large core

Sm	2.442714000	4.753162000	10.787204000
Fe	2.423295000	4.174801000	16.282981000
P	0.177849000	3.778167000	16.176426000
P	1.845722000	6.017721000	17.427901000
P	4.677561000	4.464976000	16.425696000
P	2.793964000	2.418360000	17.660607000
Si	1.666499000	1.313559000	10.694977000
Si	-0.129654000	2.943180000	8.905889000
Si	-0.023044000	7.238120000	10.921700000
Si	2.016256000	7.837364000	8.790475000
Si	5.304508000	3.583958000	8.783902000
Si	5.699477000	5.877472000	10.734313000
N	2.512724000	4.503926000	13.407535000
N	2.484894000	4.328298000	14.552578000
N	1.228947000	2.885948000	10.036076000
N	1.430216000	6.759983000	10.064808000
N	4.643254000	4.687449000	9.995497000
C	2.432328000	0.083418000	9.460116000
H	1.727289000	-0.229657000	8.686449000
H	2.750826000	-0.815444000	10.002139000
H	3.312086000	0.500791000	8.962768000
C	0.204292000	0.402321000	11.511931000
H	-0.284702000	1.030891000	12.262639000
H	0.555142000	-0.510068000	12.008603000
H	-0.553671000	0.105953000	10.779840000
C	2.978708000	1.530311000	12.058555000
H	3.843313000	2.124401000	11.738797000
H	3.365498000	0.540349000	12.327618000
H	2.568617000	1.985901000	12.963369000
C	-1.799958000	3.115913000	9.808147000
H	-1.980288000	2.253494000	10.458426000
H	-2.624384000	3.167419000	9.087276000
H	-1.844414000	4.017368000	10.426506000
C	-0.312559000	1.406235000	7.792260000
H	0.581984000	1.238886000	7.184486000
H	-1.150931000	1.577664000	7.106271000
H	-0.527722000	0.487023000	8.345614000
C	0.057770000	4.395151000	7.701946000
H	0.246165000	5.338243000	8.222654000
H	-0.851941000	4.508762000	7.101157000
H	0.890358000	4.210249000	7.014821000
C	-1.639395000	7.209431000	9.917151000
H	-1.773182000	6.259577000	9.391944000
H	-1.682363000	8.010307000	9.175042000
H	-2.491134000	7.340412000	10.595706000
C	0.082921000	8.959973000	11.728588000
H	0.977219000	9.053196000	12.352850000
H	-0.795083000	9.142362000	12.359648000

H	0.115196000	9.755575000	10.977111000
C	-0.309545000	5.999991000	12.344250000
H	-0.404410000	4.967026000	11.985339000
H	-1.256825000	6.241567000	12.839843000
H	0.482268000	6.041975000	13.098804000
C	0.635213000	8.650889000	7.757242000
H	-0.038509000	7.909051000	7.317939000
H	1.099841000	9.207030000	6.934084000
H	0.034030000	9.363541000	8.330995000
C	3.081336000	6.886448000	7.542379000
H	3.851350000	6.286431000	8.035299000
H	3.576016000	7.586527000	6.859269000
H	2.458299000	6.217349000	6.940209000
C	3.041917000	9.292847000	9.470389000
H	2.445246000	9.912357000	10.147438000
H	3.378408000	9.932033000	8.645387000
H	3.929842000	8.963860000	10.016595000
C	3.951830000	3.065866000	7.559090000
H	3.039773000	2.742123000	8.069400000
H	4.301565000	2.241088000	6.927642000
H	3.695867000	3.903801000	6.902470000
C	6.697756000	4.331944000	7.719842000
H	6.376978000	5.245119000	7.209640000
H	6.976928000	3.602033000	6.950452000
H	7.601461000	4.559604000	8.294245000
C	6.057347000	2.021164000	9.570364000
H	6.860317000	2.285640000	10.266657000
H	6.487037000	1.373209000	8.797342000
H	5.317471000	1.434085000	10.121482000
C	6.401762000	7.218289000	9.580446000
H	5.631095000	7.669200000	8.950253000
H	7.181887000	6.822813000	8.924942000
H	6.851043000	8.016467000	10.183812000
C	7.213956000	5.114462000	11.602691000
H	6.929179000	4.323042000	12.301456000
H	7.770503000	5.879377000	12.157263000
H	7.901875000	4.678033000	10.870601000
C	4.687208000	6.822120000	12.052647000
H	3.804957000	7.316404000	11.626166000
H	5.311092000	7.616384000	12.477341000
H	4.358479000	6.192693000	12.886348000
C	-0.477812000	3.063801000	14.578293000
H	-0.025513000	3.666305000	13.787629000
H	-0.018488000	2.071703000	14.501487000
C	-1.988173000	2.972494000	14.379094000
H	-2.211069000	2.605396000	13.371771000
H	-2.475526000	3.946908000	14.481336000
H	-2.457157000	2.281626000	15.085939000
C	-0.746639000	2.723081000	17.452681000
H	-0.805461000	1.722947000	17.007672000
H	-0.097810000	2.631977000	18.325906000
C	-2.135014000	3.174936000	17.905085000
H	-2.558967000	2.439413000	18.598204000
H	-2.100106000	4.132210000	18.433956000
H	-2.835312000	3.280815000	17.073278000
C	-0.668510000	5.442603000	16.322473000
H	-0.467946000	5.959718000	15.379148000
H	-1.753594000	5.347536000	16.426200000
C	-0.041111000	6.183676000	17.497388000
H	-0.367128000	5.741791000	18.447161000
H	-0.331923000	7.239661000	17.513853000
C	2.265285000	6.266900000	19.255822000
H	1.838431000	5.389455000	19.753004000
H	3.353267000	6.155224000	19.337581000
C	1.806546000	7.545774000	19.954211000
H	0.727830000	7.702876000	19.851872000
H	2.310852000	8.433206000	19.561773000
H	2.026400000	7.497716000	21.026899000
C	2.289960000	7.728359000	16.768167000
H	1.779130000	8.475354000	17.385849000

H	3.363432000	7.864331000	16.931710000
C	1.945047000	7.932122000	15.297502000
H	2.385089000	7.151589000	14.670632000
H	2.311041000	8.901540000	14.942162000
H	0.863193000	7.918089000	15.132189000
C	5.714193000	3.985926000	14.935505000
H	5.350929000	4.602227000	14.108551000
H	6.746194000	4.298417000	15.136509000
C	5.666638000	2.513475000	14.542837000
H	6.166475000	2.358873000	13.581164000
H	6.178672000	1.882530000	15.276261000
H	4.636731000	2.162211000	14.439523000
C	5.566251000	6.065280000	16.887803000
H	5.012994000	6.500647000	17.726993000
H	6.549691000	5.774528000	17.278349000
C	5.736593000	7.082504000	15.762685000
H	6.160393000	8.015953000	16.149150000
H	6.414979000	6.713717000	14.988885000
H	4.788756000	7.323995000	15.275218000
C	5.400620000	3.368736000	17.771700000
H	6.473548000	3.224524000	17.596926000
H	5.295342000	3.924165000	18.711734000
C	4.634309000	2.058508000	17.832734000
H	4.833047000	1.507465000	18.758650000
H	4.908963000	1.408235000	16.998412000
C	2.178394000	0.670045000	17.301654000
H	2.664215000	-0.012575000	18.007788000
H	1.108223000	0.648680000	17.530398000
C	2.422943000	0.219001000	15.866493000
H	2.004486000	0.930610000	15.149607000
H	1.970625000	-0.761931000	15.683880000
H	3.491020000	0.131125000	15.644878000
C	2.376139000	2.564376000	19.511207000
H	3.088222000	3.313463000	19.878536000
H	1.391856000	3.037881000	19.584437000
C	2.423632000	1.313237000	20.385385000
H	1.646979000	0.593553000	20.110394000
H	2.267818000	1.573662000	21.438607000
H	3.388233000	0.798669000	20.321737000

1-Dy, S=5/2, large core

Dy	2.338810000	13.436832000	10.834398000
Fe	1.973273000	14.199570000	16.191852000
P	-0.287447000	14.393135000	16.020532000
P	1.754702000	16.058528000	17.478305000
P	2.014839000	12.312756000	17.407607000
P	4.237266000	14.120885000	16.372930000
Si	1.896286000	10.285682000	9.060513000
Si	-0.309899000	11.203125000	10.884427000
Si	0.072906000	15.284525000	8.693902000
Si	1.820206000	16.853338000	10.587781000
Si	5.526463000	12.166014000	11.142878000
Si	5.360919000	14.311408000	9.021495000
N	2.181369000	13.765738000	13.338660000
N	2.103957000	13.989000000	14.474850000
N	1.266433000	11.509538000	10.173609000
N	1.332338000	15.291456000	9.937283000
N	4.554890000	13.333312000	10.257692000
C	2.756345000	8.849468000	9.970619000
H	3.591518000	9.183969000	10.591351000
H	3.148699000	8.123953000	9.248133000
H	2.050331000	8.321963000	10.620034000
C	3.119588000	11.053918000	7.832627000
H	2.589921000	11.698665000	7.123730000
H	3.622116000	10.267438000	7.257904000
H	3.882261000	11.652968000	8.336872000
C	0.568767000	9.452230000	7.974059000
H	-0.134701000	8.837374000	8.544310000
H	1.075768000	8.789414000	7.262349000
H	-0.004289000	10.182917000	7.395314000

C	-0.424366000	9.543736000	11.812854000
H	-0.382708000	8.692374000	11.125727000
H	-1.372005000	9.475974000	12.360458000
H	0.394336000	9.434754000	12.530739000
C	-0.662455000	12.561153000	12.174864000
H	-0.014323000	12.468688000	13.050804000
H	-1.700873000	12.471814000	12.513202000
H	-0.553514000	13.572114000	11.761678000
C	-1.795309000	11.250575000	9.694411000
H	-1.800774000	12.156346000	9.082167000
H	-2.724238000	11.234946000	10.277543000
H	-1.815388000	10.391125000	9.020080000
C	-1.679655000	15.261474000	9.442890000
H	-1.861584000	14.374370000	10.055753000
H	-2.433391000	15.274328000	8.646714000
H	-1.845734000	16.142113000	10.072028000
C	0.093358000	16.795211000	7.531211000
H	-0.129572000	17.738573000	8.039143000
H	-0.678182000	16.647088000	6.765937000
H	1.053326000	16.900474000	7.016926000
C	0.264030000	13.785601000	7.548679000
H	1.157295000	13.897640000	6.925269000
H	-0.601677000	13.706315000	6.881038000
H	0.353367000	12.851467000	8.109808000
C	0.366134000	17.849207000	11.313808000
H	-0.346059000	18.155525000	10.540816000
H	0.732044000	18.759495000	11.803352000
H	-0.178943000	17.258510000	12.056922000
C	3.051733000	16.600435000	12.019866000
H	2.563397000	16.216882000	12.918724000
H	3.497873000	17.571544000	12.264235000
H	3.881911000	15.929708000	11.767124000
C	2.711992000	18.018225000	9.373271000
H	3.562664000	17.529708000	8.890177000
H	3.095601000	18.883428000	9.927771000
H	2.052465000	18.395794000	8.588536000
C	4.446715000	11.388259000	12.512885000
H	4.143221000	12.110670000	13.275723000
H	5.024612000	10.601413000	13.011750000
H	3.541249000	10.909817000	12.119571000
C	7.048259000	12.937179000	11.993627000
H	7.793288000	13.252222000	11.255489000
H	7.531119000	12.205785000	12.652750000
H	6.782404000	13.813489000	12.591221000
C	6.202493000	10.696127000	10.139661000
H	6.601217000	9.942523000	10.829741000
H	7.015383000	10.993990000	9.472620000
H	5.429915000	10.217278000	9.533259000
C	6.167710000	15.877407000	9.746791000
H	5.436671000	16.551928000	10.200773000
H	6.687972000	16.433716000	8.958100000
H	6.905886000	15.618281000	10.512858000
C	6.763844000	13.407510000	8.099995000
H	7.614936000	13.160778000	8.742369000
H	7.135486000	14.067748000	7.307074000
H	6.413210000	12.485830000	7.625949000
C	4.136020000	14.832374000	7.670729000
H	3.857330000	13.969525000	7.057246000
H	4.595796000	15.577028000	7.010737000
H	3.222436000	15.262524000	8.090322000
C	-2.124857000	12.462431000	17.185569000
H	-2.770893000	11.598820000	16.993604000
H	-2.734658000	13.220080000	17.686393000
H	-1.353930000	12.139639000	17.891623000
C	-1.536590000	12.985330000	15.877213000
H	-2.345406000	13.333594000	15.227140000
H	-1.027492000	12.185464000	15.332109000
C	-2.181930000	16.048576000	14.523681000
H	-2.359724000	16.556786000	13.570069000
H	-2.319193000	16.791374000	15.316127000

H	-2.968878000	15.295523000	14.635925000
C	-0.781688000	15.442160000	14.541314000
H	-0.017806000	16.221920000	14.467806000
H	-0.630677000	14.794497000	13.672803000
C	-0.947995000	15.406768000	17.447841000
H	-2.000173000	15.673493000	17.300892000
H	-0.883988000	14.782706000	18.345479000
C	-0.047005000	16.626128000	17.570345000
H	-0.226799000	17.184118000	18.495284000
H	-0.223326000	17.313266000	16.736974000
C	2.103470000	15.970693000	19.343594000
H	1.318969000	15.303236000	19.719857000
H	3.042960000	15.426479000	19.465655000
C	2.137981000	17.261992000	20.158312000
H	1.236366000	17.868298000	20.020384000
H	2.998540000	17.885433000	19.898532000
H	2.212483000	17.036989000	21.228432000
C	2.584060000	17.694306000	17.032867000
H	2.311813000	18.445969000	17.781353000
H	3.664011000	17.531289000	17.121095000
C	2.246283000	18.190756000	15.631445000
H	2.398627000	17.405665000	14.885578000
H	2.874567000	19.045551000	15.359121000
H	1.205502000	18.521596000	15.556536000
C	1.706138000	10.396925000	15.313454000
H	1.549911000	11.227794000	14.620612000
H	1.170924000	9.523501000	14.926505000
H	2.773362000	10.154369000	15.297397000
C	1.229215000	10.742796000	16.719419000
H	1.445968000	9.919171000	17.408734000
H	0.145059000	10.888820000	16.730121000
C	1.535499000	10.894103000	19.953179000
H	0.851666000	10.144527000	19.544857000
H	1.276959000	11.024546000	21.010272000
H	2.547034000	10.476673000	19.916360000
C	1.439680000	12.224655000	19.208972000
H	2.039463000	12.988427000	19.717064000
H	0.409079000	12.598231000	19.223838000
C	3.800105000	11.700385000	17.613297000
H	4.144465000	12.016838000	18.605055000
H	3.828238000	10.605662000	17.597931000
C	4.678103000	12.305579000	16.525513000
H	4.459414000	11.858579000	15.550918000
H	5.746141000	12.162736000	16.722619000
C	5.325148000	14.663521000	14.952206000
H	6.290598000	14.155735000	15.052961000
H	4.851819000	14.271570000	14.047697000
C	5.514034000	16.172600000	14.829426000
H	6.129268000	16.408405000	13.955301000
H	6.013555000	16.599101000	15.705735000
H	4.557131000	16.683244000	14.700980000
C	5.164177000	14.826506000	17.855240000
H	4.932790000	15.895698000	17.896971000
H	4.691609000	14.379964000	18.736574000
C	6.675633000	14.612453000	17.909991000
H	7.189461000	15.073432000	17.061969000
H	6.941658000	13.551426000	17.922627000
H	7.087953000	15.058529000	18.821929000

1-Ce, S=1/2, large core+disp

Ce	9.280857000	8.085154000	15.776733000
Fe	8.832558000	6.482679000	10.656385000
P	6.614763000	6.356149000	10.691902000
P	8.694247000	4.451326000	9.787519000
P	11.060926000	6.517449000	10.643499000
P	8.978609000	8.039366000	9.116384000
Si	6.608122000	10.263683000	15.551886000
Si	8.852959000	11.215072000	17.460928000
Si	7.501432000	6.468868000	18.613004000
Si	8.754160000	4.738929000	16.498598000

Si	12.790737000	7.532751000	16.699507000
Si	12.171874000	9.761759000	14.740626000
N	8.866568000	7.450113000	13.350763000
N	8.852158000	7.013306000	12.277357000
N	8.147990000	10.101559000	16.328623000
N	8.376511000	6.322370000	17.107036000
N	11.657264000	8.407668000	15.705844000
C	6.095247000	8.517917000	14.974631000
H	6.677889000	8.204322000	14.101686000
H	5.043286000	8.518220000	14.671457000
H	6.207921000	7.763279000	15.761551000
C	6.714491000	11.342607000	13.996303000
H	7.490175000	10.954792000	13.329721000
H	6.987407000	12.370143000	14.258254000
H	5.767709000	11.370326000	13.445558000
C	5.207168000	10.946513000	16.625918000
H	5.344744000	12.013453000	16.823987000
H	5.147276000	10.426625000	17.584916000
H	4.249642000	10.825711000	16.106652000
C	9.539776000	12.760938000	16.605347000
H	8.709868000	13.349538000	16.198352000
H	10.202340000	12.502558000	15.776235000
H	10.094362000	13.400850000	17.300318000
C	7.676804000	11.830471000	18.816144000
H	7.166261000	11.000873000	19.312878000
H	6.915666000	12.510327000	18.424103000
H	8.252125000	12.375890000	19.573156000
C	10.230600000	10.269179000	18.365562000
H	10.961987000	9.823615000	17.682479000
H	9.786603000	9.467802000	18.969498000
H	10.778334000	10.925829000	19.049722000
C	6.513255000	8.080171000	18.674473000
H	5.612821000	8.002202000	18.056850000
H	7.095167000	8.925022000	18.298054000
H	6.199770000	8.297380000	19.701329000
C	6.227836000	5.082716000	18.881622000
H	6.676066000	4.086052000	18.924376000
H	5.484043000	5.085805000	18.078122000
H	5.698606000	5.249360000	19.826672000
C	8.692126000	6.464292000	20.088304000
H	9.368636000	7.323611000	20.039206000
H	9.309728000	5.561271000	20.092299000
H	8.150134000	6.515353000	21.039125000
C	9.484370000	3.556955000	17.790692000
H	8.799611000	3.377987000	18.624225000
H	10.410784000	3.969479000	18.201881000
H	9.717458000	2.587947000	17.334954000
C	10.077142000	4.906890000	15.145080000
H	10.935751000	5.512525000	15.451557000
H	9.676633000	5.322223000	14.216620000
H	10.465074000	3.910028000	14.913837000
C	7.276153000	3.898234000	15.656393000
H	6.846735000	4.583151000	14.918074000
H	6.491967000	3.639041000	16.372522000
H	7.582816000	2.982888000	15.137627000
C	11.864329000	6.755314000	18.158745000
H	10.913975000	6.292618000	17.873100000
H	11.637254000	7.522193000	18.906227000
H	12.475644000	5.987294000	18.644967000
C	14.154092000	8.621196000	17.446570000
H	13.727728000	9.462984000	17.999956000
H	14.825845000	9.022108000	16.680878000
H	14.759857000	8.029586000	18.142275000
C	13.683906000	6.157654000	15.737334000
H	14.078600000	6.547337000	14.793679000
H	13.011305000	5.327457000	15.505850000
H	14.521524000	5.755764000	16.318113000
C	13.564019000	9.351961000	13.515326000
H	13.244075000	8.606999000	12.784076000
H	14.444003000	8.959377000	14.035102000

H	13.871464000	10.253065000	12.972638000
C	10.683956000	10.339557000	13.708681000
H	9.842915000	10.678501000	14.326190000
H	10.324224000	9.541683000	13.051384000
H	10.975098000	11.184908000	13.076210000
C	12.789746000	11.257260000	15.732269000
H	13.803476000	11.092248000	16.106508000
H	12.146557000	11.471846000	16.588245000
H	12.809896000	12.148596000	15.094830000
C	4.515575000	5.388018000	12.451625000
H	4.189568000	4.628775000	11.733677000
H	3.899282000	6.278849000	12.298099000
H	4.293784000	5.006713000	13.453509000
C	6.000165000	5.695212000	12.323074000
H	6.610751000	4.807549000	12.513278000
H	6.312420000	6.426365000	13.073389000
C	5.411958000	7.734710000	10.335228000
H	5.685743000	8.131190000	9.351889000
H	4.415245000	7.292307000	10.227559000
C	5.412047000	8.822393000	11.401653000
H	4.840018000	9.697526000	11.078222000
H	6.423652000	9.154753000	11.642400000
H	4.965454000	8.460628000	12.330053000
C	6.023966000	5.071841000	9.476307000
H	4.959817000	4.853357000	9.610740000
H	6.154045000	5.508692000	8.479605000
C	6.910062000	3.854071000	9.660355000
H	6.806563000	3.127741000	8.848521000
H	6.658813000	3.341705000	10.592986000
C	9.444887000	3.006313000	10.715924000
H	9.218111000	2.070193000	10.196281000
H	10.530736000	3.140407000	10.689815000
C	8.950802000	2.970170000	12.157298000
H	7.914190000	2.625440000	12.219913000
H	8.991406000	3.968302000	12.603603000
H	9.553919000	2.293346000	12.770305000
C	9.203647000	4.118668000	8.002465000
H	8.415158000	4.606553000	7.417181000
H	10.115561000	4.685885000	7.810983000
C	9.378434000	2.667455000	7.573179000
H	9.549924000	2.598400000	6.493504000
H	8.495335000	2.061716000	7.801487000
H	10.234486000	2.203206000	8.071318000
C	12.038404000	6.196799000	12.192332000
H	13.023093000	6.658308000	12.071847000
H	11.540067000	6.723532000	13.012046000
C	12.178363000	4.713708000	12.506154000
H	12.708156000	4.177305000	11.712533000
H	11.202652000	4.247185000	12.639867000
H	12.741809000	4.574490000	13.431592000
C	12.043340000	5.554127000	9.372407000
H	11.793780000	4.497098000	9.503371000
H	11.628528000	5.846844000	8.403200000
C	13.554016000	5.755701000	9.381177000
H	14.023964000	5.159679000	8.591851000
H	14.002280000	5.453223000	10.331026000
H	13.825079000	6.800215000	9.204644000
C	11.537916000	8.260754000	10.179883000
H	11.220840000	8.892606000	11.014345000
H	12.620398000	8.374897000	10.068249000
C	10.773223000	8.600247000	8.906658000
H	11.196676000	8.065661000	8.049027000
H	10.810067000	9.668858000	8.674042000
C	8.467110000	7.727267000	7.340130000
H	9.036290000	6.838287000	7.048626000
H	7.417717000	7.410913000	7.385847000
C	8.656114000	8.850922000	6.327967000
H	8.418870000	8.509054000	5.314692000
H	9.689248000	9.212528000	6.317791000
H	8.007873000	9.704599000	6.543158000

C	8.197373000	9.713549000	9.432848000
H	8.447612000	10.382468000	8.602712000
H	7.113226000	9.582661000	9.426757000
C	8.661721000	10.301046000	10.757473000
H	9.716745000	10.585966000	10.720729000
H	8.550409000	9.576629000	11.568631000
H	8.091429000	11.197144000	11.019023000

1-Sm, S=5/2, large core+disp

Sm	2.431302000	4.764492000	10.886960000
Fe	2.470554000	4.165903000	16.183723000
P	0.290704000	3.710559000	16.007986000
P	1.850968000	5.949381000	17.297426000
P	4.661433000	4.524843000	16.343944000
P	2.844448000	2.471596000	17.549062000
Si	1.607925000	1.375759000	10.863522000
Si	-0.157498000	3.044018000	9.079710000
Si	0.032289000	7.201327000	11.049059000
Si	2.091157000	7.797568000	8.917148000
Si	5.207116000	3.512716000	8.973648000
Si	5.635148000	5.883234000	10.843722000
N	2.600088000	4.554326000	13.350285000
N	2.559671000	4.345298000	14.487944000
N	1.175188000	2.934708000	10.210313000
N	1.474117000	6.761178000	10.186180000
N	4.606782000	4.667760000	10.141678000
C	2.405485000	0.178024000	9.628207000
H	1.732596000	-0.078867000	8.807206000
H	2.676394000	-0.750268000	10.144804000
H	3.316572000	0.599758000	9.197369000
C	0.128910000	0.463076000	11.629968000
H	-0.403642000	1.106499000	12.334491000
H	0.460918000	-0.433118000	12.166234000
H	-0.584554000	0.146220000	10.862928000
C	2.891923000	1.609323000	12.240743000
H	3.738443000	2.233741000	11.933149000
H	3.306552000	0.632106000	12.511729000
H	2.459881000	2.053010000	13.139234000
C	-1.814017000	3.242706000	9.987077000
H	-2.010243000	2.357688000	10.601250000
H	-2.644115000	3.352567000	9.280484000
H	-1.815985000	4.116712000	10.643887000
C	-0.341556000	1.524175000	7.956286000
H	0.564968000	1.366350000	7.364174000
H	-1.171754000	1.690308000	7.260324000
H	-0.553264000	0.604972000	8.510412000
C	0.101490000	4.508615000	7.912503000
H	0.326828000	5.428489000	8.458412000
H	-0.789530000	4.678708000	7.298566000
H	0.939452000	4.296101000	7.240542000
C	-1.568095000	7.175510000	10.033893000
H	-1.675669000	6.230128000	9.495942000
H	-1.602080000	7.984581000	9.300803000
H	-2.432522000	7.286957000	10.698709000
C	0.162622000	8.897419000	11.886060000
H	1.086649000	8.965257000	12.467648000
H	-0.684091000	9.081983000	12.556690000
H	0.177000000	9.698614000	11.140004000
C	-0.232463000	5.911689000	12.424171000
H	-0.302989000	4.891892000	12.025095000
H	-1.185815000	6.110902000	12.924642000
H	0.557149000	5.941594000	13.179689000
C	0.718433000	8.603451000	7.882031000
H	0.040818000	7.850394000	7.469168000
H	1.166072000	9.151162000	7.044997000
H	0.125988000	9.317013000	8.463616000
C	3.143358000	6.784142000	7.715072000
H	3.867179000	6.159243000	8.245067000
H	3.690483000	7.440703000	7.029947000
H	2.500878000	6.131425000	7.116462000

C	3.129999000	9.229401000	9.606605000
H	2.527427000	9.845363000	10.281574000
H	3.487443000	9.871348000	8.793548000
H	4.001828000	8.878902000	10.163364000
C	3.800878000	3.021395000	7.805232000
H	2.888756000	2.767523000	8.353639000
H	4.080209000	2.158988000	7.190577000
H	3.573800000	3.856090000	7.134598000
C	6.599494000	4.200849000	7.883791000
H	6.276399000	5.103015000	7.356113000
H	6.880876000	3.453184000	7.133664000
H	7.496959000	4.443946000	8.461325000
C	5.907151000	1.972912000	9.832912000
H	6.726778000	2.261201000	10.499801000
H	6.300063000	1.254048000	9.105387000
H	5.150173000	1.463072000	10.434082000
C	6.324111000	7.168634000	9.632493000
H	5.540931000	7.588201000	8.997801000
H	7.088498000	6.735474000	8.982673000
H	6.785576000	7.992395000	10.189328000
C	7.137536000	5.136538000	11.731815000
H	6.843235000	4.313838000	12.387138000
H	7.667087000	5.886530000	12.330300000
H	7.846116000	4.738031000	10.997907000
C	4.599524000	6.859525000	12.113433000
H	3.682430000	7.274345000	11.675932000
H	5.184891000	7.711398000	12.474458000
H	4.318344000	6.263445000	12.985186000
C	-0.278280000	2.998255000	14.388486000
H	0.272932000	3.535787000	13.616293000
H	0.110341000	1.974736000	14.376716000
C	-1.770891000	3.018053000	14.088797000
H	-1.955256000	2.668638000	13.068453000
H	-2.183045000	4.028276000	14.159878000
H	-2.334055000	2.369797000	14.765815000
C	-0.622580000	2.610050000	17.235568000
H	-0.636490000	1.616512000	16.774572000
H	0.003536000	2.530438000	18.125431000
C	-2.032544000	3.022785000	17.644885000
H	-2.466360000	2.269561000	18.311255000
H	-2.033046000	3.973456000	18.185876000
H	-2.701200000	3.128539000	16.787917000
C	-0.613561000	5.335459000	16.168958000
H	-0.404588000	5.882171000	15.246323000
H	-1.695823000	5.202648000	16.242631000
C	-0.030321000	6.053522000	17.378176000
H	-0.338255000	5.556409000	18.306218000
H	-0.359676000	7.095794000	17.435448000
C	2.293798000	6.238812000	19.098188000
H	1.856071000	5.386438000	19.626796000
H	3.380080000	6.110659000	19.170479000
C	1.855877000	7.551548000	19.737381000
H	0.778882000	7.712932000	19.626645000
H	2.367750000	8.409591000	19.293632000
H	2.078849000	7.556663000	20.809811000
C	2.235257000	7.636285000	16.572347000
H	1.673390000	8.392477000	17.130671000
H	3.295498000	7.827810000	16.757223000
C	1.925888000	7.723016000	15.084197000
H	2.392697000	6.904624000	14.531210000
H	2.284074000	8.667356000	14.662409000
H	0.850008000	7.674244000	14.895062000
C	5.651779000	4.063763000	14.833780000
H	5.283004000	4.711252000	14.034573000
H	6.699172000	4.334990000	15.008627000
C	5.513321000	2.606938000	14.417716000
H	5.926547000	2.448623000	13.417022000
H	6.046012000	1.940310000	15.102597000
H	4.462038000	2.312338000	14.392133000
C	5.479332000	6.158760000	16.758933000

H	4.917292000	6.593197000	17.591896000
H	6.481807000	5.928389000	17.139192000
C	5.568824000	7.128244000	15.586390000
H	5.916026000	8.112142000	15.918098000
H	6.269375000	6.770001000	14.828236000
H	4.604292000	7.262684000	15.093123000
C	5.417097000	3.466590000	17.691073000
H	6.492908000	3.352057000	17.518489000
H	5.288661000	4.026450000	18.625183000
C	4.680504000	2.141633000	17.754307000
H	4.873781000	1.600124000	18.686101000
H	4.974107000	1.493193000	16.925721000
C	2.269304000	0.725564000	17.171192000
H	2.801185000	0.036631000	17.836039000
H	1.208943000	0.659834000	17.428136000
C	2.490888000	0.360985000	15.710971000
H	2.027364000	1.098315000	15.051985000
H	2.075435000	-0.625051000	15.479351000
H	3.554652000	0.340521000	15.456996000
C	2.378958000	2.640563000	19.370352000
H	3.084046000	3.388816000	19.751026000
H	1.394921000	3.118493000	19.406577000
C	2.399796000	1.386423000	20.234961000
H	1.634693000	0.670081000	19.922394000
H	2.210632000	1.632405000	21.285497000
H	3.367144000	0.875767000	20.190474000

1-Dy, S=5/2, large core+disp

Dy	2.326822000	13.416576000	10.914624000
Fe	1.996123000	14.190170000	16.103316000
P	-0.206143000	14.389244000	15.897993000
P	1.799365000	16.001327000	17.356664000
P	1.996615000	12.360853000	17.308107000
P	4.210838000	14.093735000	16.260858000
Si	1.945747000	10.322121000	9.180592000
Si	-0.301603000	11.261777000	10.960404000
Si	0.040180000	15.231249000	8.877016000
Si	1.843219000	16.767213000	10.763348000
Si	5.467179000	12.193176000	11.246370000
Si	5.264818000	14.364120000	9.143577000
N	2.178783000	13.682848000	13.292846000
N	2.118803000	13.947051000	14.419131000
N	1.274674000	11.519432000	10.268865000
N	1.302013000	15.251241000	10.091683000
N	4.516877000	13.361102000	10.371068000
C	2.803103000	8.919895000	10.129745000
H	3.598770000	9.289274000	10.780741000
H	3.244088000	8.191541000	9.439966000
H	2.077035000	8.393081000	10.757109000
C	3.174036000	11.144677000	8.001706000
H	2.634807000	11.785606000	7.297067000
H	3.725001000	10.394307000	7.424667000
H	3.894180000	11.762452000	8.543920000
C	0.640633000	9.489622000	8.081133000
H	-0.070804000	8.888088000	8.655493000
H	1.140543000	8.820674000	7.371395000
H	0.078298000	10.231106000	7.505911000
C	-0.424928000	9.619661000	11.901600000
H	-0.408606000	8.769419000	11.212157000
H	-1.351705000	9.559816000	12.483288000
H	0.422192000	9.512623000	12.584841000
C	-0.622189000	12.652756000	12.217245000
H	-0.014028000	12.524742000	13.114977000
H	-1.675647000	12.646454000	12.515053000
H	-0.422402000	13.647096000	11.798393000
C	-1.752133000	11.321824000	9.740887000
H	-1.720465000	12.223353000	9.124779000
H	-2.698132000	11.323902000	10.294859000
H	-1.758269000	10.458094000	9.072247000
C	-1.685484000	15.186407000	9.668729000

H	-1.847492000	14.280944000	10.257762000
H	-2.467927000	15.232367000	8.903044000
H	-1.813033000	16.046713000	10.334096000
C	0.052164000	16.753966000	7.743825000
H	-0.162024000	17.681626000	8.283376000
H	-0.717981000	16.633851000	6.973384000
H	1.017209000	16.863894000	7.240705000
C	0.266065000	13.733927000	7.743409000
H	1.148363000	13.881179000	7.112397000
H	-0.601623000	13.603079000	7.087841000
H	0.408256000	12.815266000	8.318631000
C	0.426561000	17.746464000	11.561023000
H	-0.291651000	18.089571000	10.809535000
H	0.801901000	18.627076000	12.094239000
H	-0.108590000	17.113950000	12.274020000
C	3.113838000	16.431215000	12.135887000
H	2.636838000	16.072164000	13.048875000
H	3.627108000	17.370650000	12.367134000
H	3.890942000	15.711951000	11.850595000
C	2.709790000	17.930678000	9.540831000
H	3.509258000	17.418000000	9.000610000
H	3.157634000	18.766898000	10.090382000
H	2.018998000	18.346724000	8.804319000
C	4.371033000	11.419540000	12.601432000
H	4.112783000	12.130634000	13.388328000
H	4.914165000	10.587227000	13.062716000
H	3.435999000	11.002574000	12.206429000
C	6.980335000	12.979746000	12.082508000
H	7.744635000	13.217486000	11.335332000
H	7.432244000	12.302033000	12.815598000
H	6.711468000	13.909961000	12.588423000
C	6.122137000	10.738103000	10.222585000
H	6.541503000	9.976638000	10.890440000
H	6.912280000	11.052273000	9.536556000
H	5.331600000	10.269577000	9.632924000
C	6.039900000	15.927688000	9.889641000
H	5.291690000	16.576108000	10.351027000
H	6.557889000	16.508654000	9.118399000
H	6.772334000	15.657465000	10.657212000
C	6.656522000	13.480342000	8.202585000
H	7.506475000	13.233279000	8.846148000
H	7.025352000	14.134573000	7.404505000
H	6.295376000	12.556795000	7.740748000
C	3.979819000	14.857370000	7.845753000
H	3.718289000	13.989141000	7.233308000
H	4.376342000	15.633092000	7.181761000
H	3.065065000	15.235202000	8.310295000
C	-2.007435000	12.458065000	17.019641000
H	-2.607233000	11.559407000	16.843196000
H	-2.658379000	13.202363000	17.486395000
H	-1.233442000	12.196158000	17.746455000
C	-1.418290000	12.973889000	15.711798000
H	-2.220022000	13.302968000	15.044971000
H	-0.880271000	12.183917000	15.182876000
C	-2.039795000	15.996761000	14.315346000
H	-2.157451000	16.562681000	13.385821000
H	-2.291304000	16.668999000	15.141805000
H	-2.784396000	15.195068000	14.299114000
C	-0.619593000	15.463376000	14.429389000
H	0.108745000	16.277846000	14.464480000
H	-0.353047000	14.867559000	13.553609000
C	-0.896030000	15.379737000	17.319031000
H	-1.944964000	15.647906000	17.158160000
H	-0.841633000	14.744037000	18.208483000
C	0.009656000	16.591012000	17.463653000
H	-0.159744000	17.132902000	18.399183000
H	-0.162284000	17.292012000	16.642338000
C	2.164203000	15.907249000	19.203361000
H	1.336898000	15.304957000	19.596618000
H	3.062139000	15.298036000	19.321341000

C	2.298252000	17.214012000	19.974713000
H	1.434760000	17.870478000	19.824753000
H	3.191024000	17.768624000	19.671806000
H	2.379311000	17.024589000	21.050549000
C	2.636351000	17.602282000	16.852206000
H	2.398011000	18.389903000	17.573676000
H	3.715076000	17.424409000	16.906937000
C	2.245920000	18.016308000	15.439248000
H	2.305314000	17.162905000	14.758453000
H	2.904167000	18.802984000	15.057993000
H	1.222535000	18.401096000	15.397505000
C	1.669378000	10.582447000	15.141693000
H	1.467213000	11.449862000	14.509865000
H	1.158256000	9.716566000	14.710955000
H	2.744136000	10.389146000	15.082534000
C	1.219479000	10.819004000	16.577075000
H	1.476708000	9.964280000	17.211738000
H	0.134584000	10.936719000	16.626290000
C	1.363426000	10.943639000	19.779910000
H	0.645393000	10.258747000	19.320487000
H	1.085169000	11.050554000	20.833949000
H	2.346998000	10.464174000	19.747779000
C	1.376571000	12.296891000	19.079392000
H	2.018724000	13.006591000	19.612516000
H	0.377045000	12.744957000	19.074157000
C	3.761239000	11.723614000	17.551845000
H	4.102094000	12.068145000	18.534881000
H	3.770915000	10.629503000	17.568021000
C	4.646348000	12.288177000	16.450846000
H	4.419663000	11.823913000	15.487591000
H	5.713146000	12.143864000	16.647898000
C	5.244920000	14.612498000	14.808510000
H	6.215602000	14.111647000	14.875524000
H	4.739690000	14.217556000	13.923570000
C	5.402475000	16.121864000	14.688519000
H	5.940042000	16.379778000	13.771420000
H	5.957743000	16.544585000	15.531992000
H	4.427785000	16.609324000	14.643144000
C	5.149561000	14.827233000	17.705846000
H	4.897797000	15.890735000	17.750810000
H	4.709429000	14.371783000	18.598659000
C	6.661300000	14.632777000	17.704594000
H	7.132217000	15.096981000	16.834300000
H	6.935322000	13.574153000	17.704070000
H	7.105998000	15.083098000	18.598229000

References

1 M. Hirano, M. Akita, T. Morikita, H. Kubo, A. Fukuoka and S. Komiya, *J. Chem. Soc. Dalt. Trans.*, 1997, 3453–3458.

2 J. Parry, E. Carmona, S. Coles and M. Hursthouse, *J. Am. Chem. Soc.*, 1995, **117**, 2649–2650.

3 R. A. Andersen, *Inorg. Chem.*, 1979, **18**, 1507–1509.

4 S. M. Mansell, B. F. Perandones and P. L. Arnold, *J. Organomet. Chem.*, 2010, **695**, 2814–2821.

5 S. M. Mansell, N. Kaltsoyannis and P. L. Arnold, *J. Am. Chem. Soc.*, 2011, **133**, 9036–9051.

6 D. C. Bradley, J. S. Ghotra and F. A. Hart, *J. Chem. Soc. Dalt. Trans.*, 1973, 1021.

7 G. Brauer, W. A. Herrmann and F. T. Edelmann, *Synthetic Methods of Organometallic and Inorganic Chemistry*, Georg Thieme Verlag, 1996, **6**, 37.

8 M. Xemard, PhD Thesis, Université Paris-Saclay, 2020.

9 W. J. Evans, D. K. Drummond, H. Zhang and J. L. Atwood, *Inorg. Chem.*, 1988, **27**, 575–579.

10 **CrysAlisPro** (Rigaku, V1.171.43.107a, 2024)

11 Sheldrick, G.M., ShelXT-Integrated space-group and crystal-structure determination, *Acta Cryst.*, (2015), **A71**, 3-8.

12 O.V. Dolomanov and L.J. Bourhis and R.J. Gildea and J.A.K. Howard and H. Puschmann, Olex2: A complete structure solution, refinement and analysis program, *J. Appl. Cryst.*, (2009), **42**, 339-341.

13 Sheldrick, G.M., Crystal structure refinement with ShelXL, *Acta Cryst.*, (2015), **C71**, 3-8.

14 (a) A. D. Becke, J. Chem. Phys. 1993, 98, 5648; (b) K. Burke, J. P. Perdew, W. Yang, in Electronic Density Functional Theory: Recent Progress and New Directions, Eds: J. F. Dobson, G. Vignale, M. P. Das, Plenum, New York, 1998.

15 (a) M. Dolg, U. Wedig, H. Stoll, H. Preuss, J. Chem. Phys. 1987, 86, 866; (b) A. Hollwarth, M. Bohme, S. Dapprich, A.W. Ehlers, A. Gobbi, V. Jonas, K.F. Kohler, R. Stegmann, A. Veldkamp, G. Frenking J. Chem. Phys. 1993, 208, 237; (c) W. Kuechle, M. Dolg, H. Stoll, H. Preuss, J. Chem. Phys. 1994, 100, 7535; (d) X. Cao, M. Dolg, H. Stoll, J. Chem. Phys. 118, 487 (2003) cf. also X. Cao, M. Dolg, J. Molec. Struct. (Theochem) 2004, 673, 203 (e) M. Dolg, H. Stoll, A. Savin, H. Preuss, Theor. Chim. Acta 1989, 75, 173 (f) M. Dolg, H. Stoll, H. Preuss, Theor. Chim. Acta 1993, 85, 441.

16 (a) P. C. Hariharan and J. A. Pople, Theor. Chim. Acta 1973, 28, 213; (b) W. J. Hehre, R. Ditchfield and J. A. Pople, J. Chem. Phys. 1972, 56, 2257.

17 S. Grimme, S. Ehrlich, L. Goerigk, J. Comp. Chem., 2011, 32, 1456.

18 A. V. Marenich, C. J. Cramer, and D. G. Truhlar, J. Phys. Chem. B, 2009, 113 6378.

19 Gaussian 16, Revision B.01, M. J. Frisch, G. W. Trucks, H. B. Schlegel, G. E. Scuseria, M. A. Robb, J. R. Cheeseman, G. Scalmani, V. Barone, G. A. Petersson, H. Nakatsuji, X. Li, M. Caricato, A. V. Marenich, J. Bloino, B. G. Janesko, R. Gomperts, B. Mennucci, H. P. Hratchian, J. V. Ortiz, A. F. Izmaylov, J. L. Sonnenberg, D. Williams-Young, F. Ding, F. Lipparini, F. Egidi, J. Goings, B. Peng, A. Petrone, T. Henderson, D. Ranasinghe, V. G. Zakrzewski, J. Gao, N. Rega, G. Zheng, W. Liang, M. Hada, M. Ehara, K. Toyota, R. Fukuda, J. Hasegawa, M. Ishida, T. Nakajima, Y. Honda, O. Kitao, H. Nakai, T. Vreven, K. Throssell, J. A. Montgomery, Jr., J. E. Peralta, F. Ogliaro, M. J. Bearpark, J. J. Heyd, E. N. Brothers, K. N. Kudin, V. N. Staroverov, T. A. Keith, R. Kobayashi, J. Normand, K. Raghavachari, A. P. Rendell, J. C. Burant, S. S. Iyengar, J. Tomasi, M. Cossi, J. M. Millam, M. Klene, C. Adamo, R. Cammi, J. W. Ochterski, R. L. Martin, K. Morokuma, O. Farkas, J. B. Foresman, and D. J. Fox, Gaussian, Inc., Wallingford CT, 2016.
